# Evidence for a Novel Mechanism of Influenza Virus-Induced Type I Interferon Expression by a Defective RNA-Encoded Protein

**DOI:** 10.1371/journal.ppat.1004924

**Published:** 2015-05-29

**Authors:** Yvonne Boergeling, Timofey S. Rozhdestvensky, Mirco Schmolke, Patricia Resa-Infante, Thomas Robeck, Gerrit Randau, Thorsten Wolff, Gülsah Gabriel, Jürgen Brosius, Stephan Ludwig

**Affiliations:** 1 Institute of Molecular Virology (IMV), Center for Molecular Biology of Inflammation (ZMBE), University of Muenster, Muenster, Germany; 2 Institute of Experimental Pathology, Center for Molecular Biology of Inflammation (ZMBE), University of Muenster, Muenster, Germany; 3 Viral Zoonosis and Adaptation, Heinrich-Pette-Institute, Leibniz Institute for Experimental Virology, Hamburg, Germany; 4 Division of Influenza Viruses and Other Respiratory Viruses, Robert Koch Institute, Berlin, Germany; 5 Institute of Evolutionary and Medical Genomics, Brandenburg Medical School (MHB), Neuruppin, Germany; 6 Interdisciplinary Center of Clinical Research (IZKF), Medical Faculty, University of Muenster, Muenster, Germany; 7 Cells-in-Motion Cluster of Excellence, University of Muenster, Muenster, Germany; University of Pennsylvania, UNITED STATES

## Abstract

Influenza A virus (IAV) defective RNAs are generated as byproducts of error-prone viral RNA replication. They are commonly derived from the larger segments of the viral genome and harbor deletions of various sizes resulting in the generation of replication incompatible viral particles. Furthermore, small subgenomic RNAs are known to be strong inducers of pattern recognition receptor RIG-I-dependent type I interferon (IFN) responses. The present study identifies a novel IAV-induced defective RNA derived from the PB2 segment of A/Thailand/1(KAN-1)/2004 (H5N1). It encodes a 10 kDa protein (PB2_∆_) sharing the N-terminal amino acid sequence of the parental PB2 protein followed by frame shift after internal deletion. PB2_∆_ induces the expression of IFNβ and IFN-stimulated genes by direct interaction with the cellular adapter protein MAVS, thereby reducing viral replication of IFN-sensitive viruses such as IAV or vesicular stomatitis virus. This induction of IFN is completely independent of the defective RNA itself that usually serves as pathogen-associated pattern and thus does not require the cytoplasmic sensor RIG-I. These data suggest that not only defective RNAs, but also some defective RNA-encoded proteins can act immunostimulatory. In this particular case, the KAN-1-induced defective RNA-encoded protein PB2_∆_ enhances the overwhelming immune response characteristic for highly pathogenic H5N1 viruses, leading to a more severe phenotype *in vivo*.

## Author Summary

Error-prone polymerase function of RNA viruses can result in expression of defective RNAs harboring internal deletions of various sizes. Small subgenomic RNAs are strong inducers of the antiviral response by serving as pathogen-associated patterns that are predominantly detected by cellular sensors. Recently, it has been shown that influenza A virus defective RNAs are not only generated upon passages in cell culture, but also in infected humans, indicating that these subgenomic RNAs may also be relevant in infections *in vivo*. Here, we characterize a novel defective RNA derived from the PB2 segment of a highly pathogenic H5N1 influenza A virus. This RNA encodes a 10 kDa peptide (PB2_Δ_) which activates type I interferon (IFN) responses through direct interaction with the adapter protein MAVS, a key component of the RIG-I-dependent IFN induction. This is the first time that such a function was described for a defective RNA-encoded protein, a finding that has several important implications with regard to deciphering viral protein functions and options for immunostimulatory approaches. Furthermore, this is an example of how influenza viruses may acquire novel polypeptides with altered functions from its limited genome.

## Introduction

Influenza A viruses (IAV) belong to the family of *Orthomyxoviridae* and are characterized by a segmented single-stranded RNA genome with negative orientation. These eight segments encode nine structural and up to seven nonstructural proteins [1–5]. For the production of infectious virus particles at least one copy of each of the segments needs to be packaged into the progeny particle during the virion assembly step [6].

Non-infectious particles exhibiting an incomplete viral genome e.g. due to a large internal deletion within at least one segment, are defined as defective interfering particles (DIPs) [7, 8]. These DIPs need the presence of a helper virus for replication of their defective RNA [9]. The generation of IAV DI RNAs has been linked to the ribonucleoprotein tertiary structure that leads to jumping of the viral polymerase making transitions between adjacent regions of the RNA template. Furthermore, there are indications that polymerase skipping is facilitated by short sequence repeats within viral segments [10]. It has been shown that the competition for viral RNA polymerases and preferential packaging of over-abundant DI RNA segments interferes with replication and packaging of full-length segments of replication competent helper virus [7, 8, 11–13].

IAV DI particles have been observed primarily after multiple passages of viruses in cell culture at high multiplicity of infection or in experimentally infected embryonated chicken eggs [10, 14, 15] and therefore were considered as experimental artifacts. However, subgenomic RNAs were isolated recently *in vivo* from naso-pharyngeal swabs of human patients [16]. The same study demonstrated that identical *in vivo*-derived defective RNAs were present in patients linked by direct contact which might suggest efficient co-transmission between them. It is thus reasonable to assume that defective RNAs are common byproducts also in natural infections albeit their function in the biology of influenza viruses and its interactions with the host remains elusive.

Defective RNAs have been shown to act as efficient pathogen-associated molecular patterns (PAMPs) recognized more potently by the sensor molecule retinoic acid-inducible gene-I (RIG-I) than viral genomic segments [17]. This activation of RIG-I leads to the induction of interferon beta (IFNβ) which is one of the key cytokines orchestrating a broadly reactive antiviral program upon infection [18]. Recently, Tapia and colleagues provided evidence that the appearance of defective RNAs coincides with the production of cytokines during IAV infection [19].

It has been shown previously that some subgenomic RNAs harbor open reading frames that might be expressed in the presence of a helper virus or could be *in vitro* translated into polypeptides [20, 21]. But, so far, a putative biological function of these proteins has never been analyzed, although they might share sequence similarities with their parental proteins and might therefore exhibit related or even different functions. In the past years, the discovery of novel IAV-encoded proteins led to new insights into viral pathogenicity [1–4, 22]. Surprisingly, all viral nonstructural proteins discovered since 2001 have been shown to be nonessential for viral replication [1–4] or even possess antiviral activities under certain conditions [23]. However, several analyses demonstrated that changes in expression levels of these proteins were linked to virulence *in vivo* [3, 23, 24]. Therefore, defective RNA-encoded proteins may also contribute to the course of infections *in vivo* and could exemplify a mechanism on how influenza viruses acquire novel polypeptides with altered functions from its limited genome.

The present study describes for the very first time a defective RNA-encoded functional polypeptide, named PB2_∆_. The subgenomic RNA was identified in the H5N1 strain A/Thailand/KAN-1/2004 (KAN-1). It belongs to the group of defective RNAs derived from the PB2 segment and potently restricts viral replication by a mechanism independent of DI RNA-mediated interference. The present study demonstrates that the defective RNA-encoded polypeptide PB2_Δ_ directly interacts with the mitochondrial antiviral signaling protein (MAVS). In contrast to the effects of PB2-MAVS interaction, this leads to the induction of IFNβ expression, thereby diminishing viral replication. Furthermore, the presence of this particular defective RNA-encoded protein in KAN-1 infection leads to higher expression levels of antiviral acting genes also *in vivo*, resulting in a more severe disease phenotype.

## Results

### Characterization of PB2_∆_ defective interfering (DI)-like RNA

In recent years, new influenza encoded non-structural proteins were discovered which could be either linked to viral polymerase activity [25] or host cell response [3, 22, 26, 27] and are therefore determinants of viral virulence.

**Fig 1:**
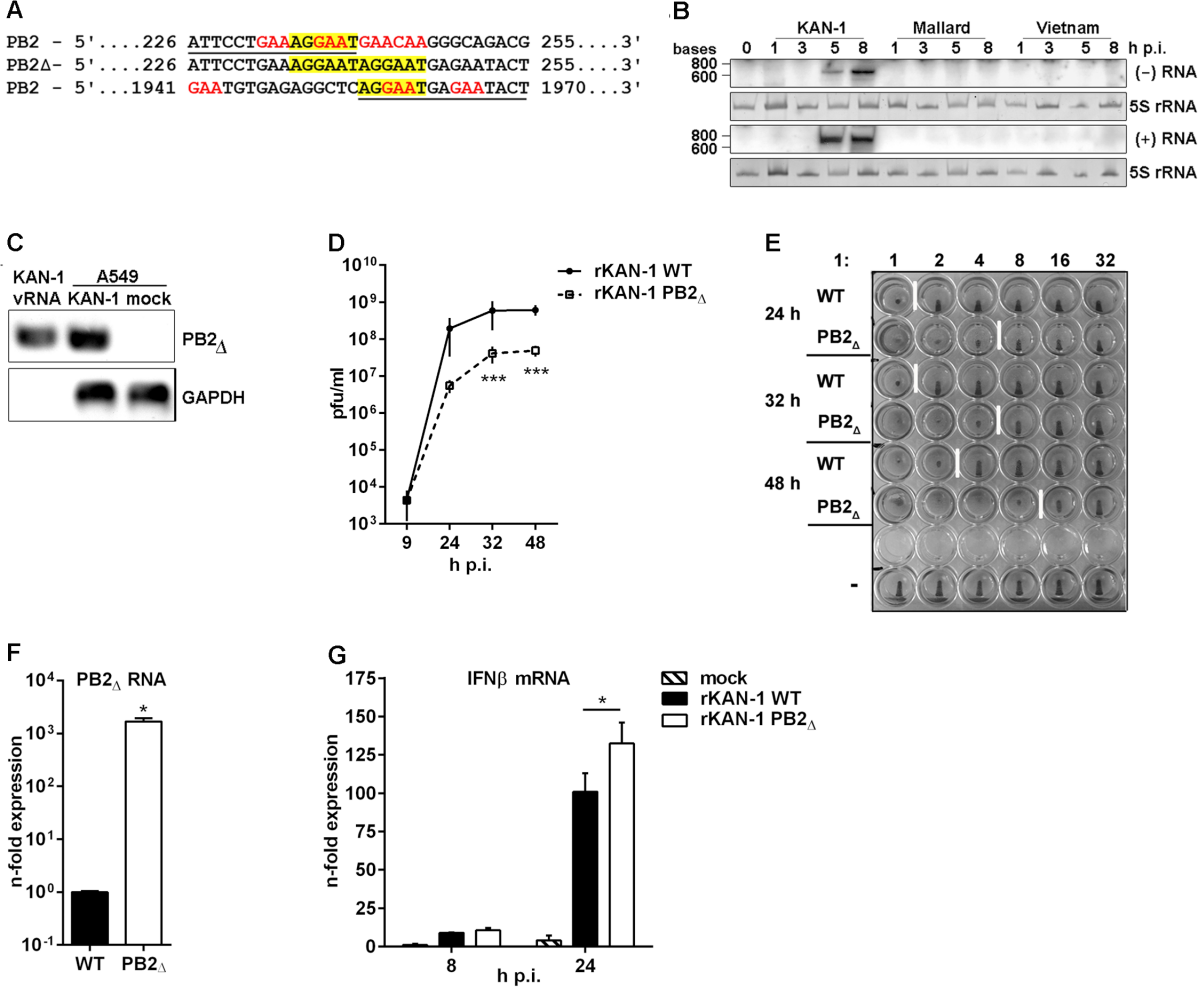
Characterization of the H5N1 KAN-1-expressed PB2_∆_ DI-like RNA. **A)** Sequence analysis of the PB2_**∆**_ DI-like RNA (middle lane) in comparison to the parental PB2 segment (upper and lower lane). Sequence identity is underlined in PB2 sequences and characteristic DI RNA sequence motives are displayed in color. **B)** Northern blot analysis of total RNA isolated from A549 infected with 5 MOI of different H5N1 isolates (KAN-1, Mallard and Vietnam) for the indicated time points. PB2_**∆**_ vRNA and c/mRNA were detected by hybridization probes covering the PB2_**∆**_ junction site. Visualization was achieved by autoradiography. As loading control serves 5S rRNA in ethidiumbromide stained PAA gels. **C)** A549 cells were infected with 5 MOI of KAN-1 for 8 h, subsequently viral particles were purified from cell culture supernatants and total RNA was isolated. Total RNA from uninfected or KAN-1-infected A549 served as controls. Detection of PB2_**∆**_ RNA and GAPDH mRNA was performed by qRT PCR and analyzed by agarose gel. **B, C)** Northern blot and agarose gel are representative of three independent experiments. **D)** A549 cells were infected with 0.01 MOI rKAN-1 WT or PB2_**∆**_ and virus-containing supernatants were harvested at the time points indicated. Infectivity titers were determined by a standard plaque assay and are depicted as mean (±SD) of one representative of three independent experiments. ***p≤0.001; two-way ANOVA, Sidak’s multiple comparisons test. **E)** Analysis of the relative amount of viral particles (infectious and noninfectious) in supernatants from (D) by hemagglutination assay. To exclude differences due to different replication abilities of the two viruses 1x10^6^ infectious particles were used for the assay which is representative for three independent experiments. **F)** A549 cells were infected with 5 MOI of rKAN-1 WT or PB2_**∆**_ and 2 h p.i. total RNA was isolated. Expressional changes of PB2_**∆**_ RNA were detected by qRT-PCR and are depicted as mean *n*-fold (±SD) of one representative out of two independent experiments normalized to rKAN-1 WT. *p≤0.05; unpaired two-tailed Student’s t-test. **G)** A549 cells were infected with 1 MOI of rKAN-1 WT or PB2_**∆**_ and 8 or 24 h p.i. total RNA was isolated. Expressional changes of IFNβ mRNA were detected by qRT-PCR and are depicted as mean *n*-fold (±SD) of one representative out of two independent experiments normalized to control. *≤0.05; two-way ANOVA, Tukey’s multiple comparisons test. Significances between the different time points and to mock infection are not indicated.

During analysis of a cDNA library generated from total RNAs of A549 cells infected with different IAV subtypes, cDNAs representing a 618 nt-long RNA were identified in H5N1 strain A/Thailand/KAN-1/2004-infected cultures. This RNA has sequence identity to the 5’ and 3’ regions of the viral PB2 gene but lacks 1715 nt from the PB2 internal region. Hence, the PB2_Δ_ RNA retains viral promoter and packaging information required for efficient viral replication (Fig 1A) and thus possesses a structure that is typical for viral defective interfering RNAs [7]. The internal deletion junction site of the PB2_∆_ RNA occurs at nucleotide position 240 as observed previously for other defective RNAs with junction sites located around positions 200–300 *in vitro* and *in vivo* [10, 16]. Furthermore, this RNA harbors characteristic defective RNA sequence motifs as described by Jennings and colleagues (1983) such as the frequent occurrence of the sequences 5’..GAA..3’ and 5’..CAA..3’ near the junction site, the number of adenosine residues varying from 2 to 3. In addition, it has been described that identification of the precise junction is frequently not possible due to local sequence similarities [10]. Here, short repeat sequences at the junction site were also observed, although these nucleotides are not deleted but still present at both ends (5’..AGGAAT/AGGAAT..3’) (Fig 1A). Therefore, it can be concluded that the PB2_∆_ RNA structurally belongs to the group of viral defective interfering (DI)-like RNAs.

To test whether PB2_∆_ RNA was expressed also by closely related H5N1 viruses total RNA was isolated from A549 cells infected with A/Thailand/1(KAN-1)/2004, A/Vietnam/1203/2004 or A/Mallard/Bavaria/1/2006. A specific Northern blot probe complementary to the junction site was designed, thus preventing cross-hybridization with the PB2 genomic vRNA under the hybridization conditions applied. The PB2_∆_ RNA was detectable only in KAN-1-infected cells (Fig 1B), although especially the Vietnam strain shows high sequence similarity and sequence repetition at the junction site within the PB2 gene (S1 Table, NCBI’s Influenza Virus Resource [28] KAN-1: CY111595.1 and Vietnam: HM006756.1). Interestingly, sequence alignments showed that this repeat is present in 53% of all known human and 79% of all avian H5N1 strains compared (S1 and S2 Tables), while all other strains show in general only a single nucleotide substitution. To exclude that the generation of the PB2_Δ_ RNA was a KAN-1 stock-specific event, the ability of *de novo* PB2_Δ_ RNA synthesis was examined for the H5N1 strains KAN-1 and Vietnam. Undiluted passages of plaque-purified clones from PB2_Δ_-free virus preparations performed as described by Nayak and colleagues [29] uncovered PB2_Δ_ RNA expression in different passages to various extents for both strains (e.g. KAN-1 clones 8 and 12 as well as Vietnam clones 3 and 7; S3 Table). Although PB2_Δ_ RNA expression is a nonessential event and not specific for KAN-1, this strain seems to exhibit higher PB2_Δ_ expression levels compared to Vietnam virus.

DI dependent interference is characterized by competition for viral polymerases during replication of vRNA into cRNA and preferential packaging of over-abundant DI segments [12, 13]. To test whether PB2_∆_ RNA can be replicated by the viral polymerase complex, Northern blot hybridization was performed with a specific probe complementary to PB2_∆_ (+)RNA. As expected, the presence of PB2_∆_ (+)RNA upon infection with the KAN-1 isolate was confirmed, suggesting that the PB2 DI-like RNA is a substrate for the viral polymerase and is amplified upon viral replication (Fig 1B). This was further verified by strand-specific qRT-PCR, uncovering the existence of all three viral RNA species (S1 Fig).

One further aspect of the interfering activity of defective viral RNAs is the preferential packaging of RNAs which exist at high copy numbers within the cell [12]. The retained 5’ and 3’ regions of the PB2 segment within the PB2_∆_ RNA suggest that packaging of this smaller RNA fragment into virions is possible. The presence of PB2_∆_ vRNA in KAN-1 viral particles was confirmed by qRT-PCR (Fig 1C). Total RNA from KAN-1- and mock-infected A549 cells served as controls, showing higher amounts of PB2_∆_ RNA in infected cells compared to viral particles which might be due to the detection of PB2_∆_ RNA in negative and positive orientation. To exclude contamination of viral RNA with cellular RNA, the presence of GAPDH mRNA was analyzed, showing no PCR product from total RNA of viral particles (Fig 1C).

Hence, specific expression of PB2_∆_ DI-like RNA was confirmed and conversion into (+)RNA was shown, arguing that PB2_∆_ DI-like RNA serves as a substrate for the viral polymerase. Furthermore, it is packaged into viral particles and, thus, possesses all crucial features of defective RNAs owning interfering activity.

To answer the question as to whether the PB2_∆_ DI-like RNA exhibits interfering activity, a recombinant KAN-1 virus was produced by reverse genetics [30] harboring PB2_∆_ DI-like RNA as additional segment. This method has the advantage that the artificially introduced RNA predominantly persists over newly originated defective RNAs which are in an inferior position due to copy numbers [11, 31]. Furthermore, the generation of wild type virus allows the comparative analysis with a PB2_∆_ RNA-free virus.

To investigate whether the PB2_∆_ DI-like RNA exhibits interfering activity, replication of the recombinant PB2_∆_ RNA-expressing KAN-1 (rKAN-1 PB2_∆_) and of wild type virus (rKAN-1 WT) were compared (Fig 1D). Although 9 h p.i. there were no differences in viral replication, infectivity titers were significantly reduced by one log step at all further time points analyzed. Furthermore, rKAN-1 PB2_∆_ indeed induces the production of non-infectious particles as confirmed by hemagglutination assay (Fig 1E). This method allows the determination of the relative amount of particles in a virus suspension with a predefined number of infectious particles due to the same ability of infectious and non-infectious influenza particles to agglutinate avian erythrocytes. Here, an increased amount of virus particles was observed at all time points analyzed which was elevated by a factor of 4 for rKAN-1 PB2_∆_ infection compared to wild type virus (Fig 1E). To evaluate whether these differences are due to non-infectious particles induced by the PB2_∆_ DI-like RNA, whole genomes of both recombinant viruses were sequenced. This analysis revealed no differences in viral segment sequences neither among each other nor to the PB2_Δ_-expressing KAN-1 isolate, whereas a strong expression of PB2_∆_ m/cRNA for rKAN-1 PB2_∆_ was observed (Fig 1F), confirming that the presence of PB2_Δ_ defective RNA indeed induces the production of non-infectious particles.

In recent years, increased attention has been paid to defective RNA-induced interferon expression. The first evidence of an influenza DI RNA inducing IFN expression came from DI 244 which protected wild type mice from lethal infections with heterologous paramyxo- and influenza B virus, but failed in protection of type I IFN receptor-deficient mice [32, 33]. The main PAMP that is sensed by different pattern-recognition receptors (PRRs) to induce the type I IFN response in viral infections is viral RNA. Particularly, detection of the 5’-triphosphate (5’-ppp) structure in viral RNAs by the cytoplasmic helicase RIG-I plays an important role in influenza A virus infection [34]. It has been shown that RIG-I preferentially binds 5’-ppp-containing small genomic segments and subgenomic RNAs [17]. Moreover, defective RNA generation coincides with the production of cytokines during IAV infection [19]. In addition, double-stranded RNAs of more than 30 base pairs in length, often found in snap- or copyback DI RNAs of *Paramyxo-* and *Rhabdoviridae* due to long double-stranded regions, also can trigger IFN responses [11]. To analyze whether PB2_∆_ DI-like RNA affects viral replication via enhancing virus-induced innate immune responses, the expression of IFNβ was determined by qRT-PCR. IFNβ is the most crucial mediator of the innate type I IFN response upon IAV infection [35]. No differences in IFNβ mRNA expression were detectable 8 h p.i. between the two recombinant viruses (Fig 1G), although PB2_∆_ RNA was expressed in high amounts as early as 2 h p.i. (Fig 1F). Interestingly, 24 h p.i. IFNβ mRNA levels were significantly increased upon infection with rKAN-1 PB2_∆_, suggesting that the reduced infectivity titers may be in part due to the induction of a strong host cell response.

### PB2_∆_ protein impairs viral protein expression without affecting the viral polymerase complex

**Fig 2:**
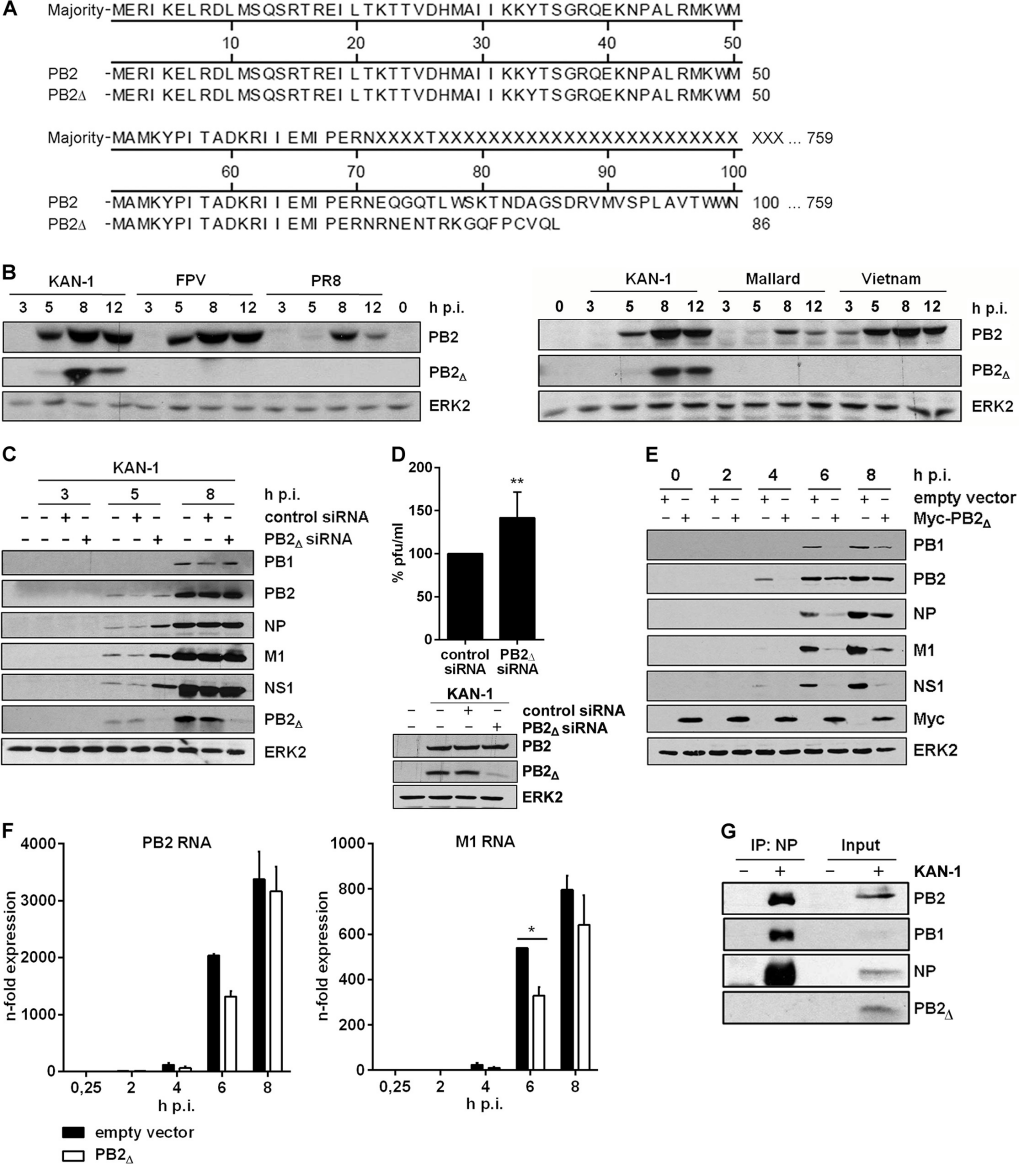
The PB2_∆_ mRNA encodes a 10 kDa protein that impairs viral gene expression. **A)** Sequence alignment of the PB2_**∆**_ protein in comparison to PB2. **B)** Western blot analysis of total lysates of A549 infected with 5 MOI of different influenza viruses of the subtypes H5N1 (KAN-1, Mallard, Vietnam; *right*), H7N7 (FPV) and H1N1 (PR8) (*left*). PB2_**∆**_ and PB2 were detected 3, 5, 8 and 12 h p.i.. Equal loading was verified by the detection of total ERK2. **C, D)** A549 cells were transfected with PB2_**∆**_-specific or scrambled siRNA and subsequently infected with KAN-1 (**C**: MOI 5, **D**: MOI 2) for the indicated time points. **C)** Expression of viral proteins PB1, PB2, NP, M1 and NS1 upon PB2_**∆**_ knockdown was analyzed by Western blot. Knockdown efficiency was verified by using PB2-specific antibodies. ERK2 expression served as loading control. **D)** Changes in infectivity titers upon PB2_**∆**_ knockdown were determined by standard plaque assay and are depicted as mean (±SD) of six independent experiments (*above*). **p≤0.01, unpaired two-tailed Student’s t-test. Efficient knockdown of PB2_**∆**_ was verified by Western blot analysis (*below)*. Efficient infection was confirmed by immunodetection of viral PB2 and equal loading was verified by analysis of total ERK2 expression. **E, F)** A549 cells were transfected with PB2_**∆**_ or empty vector and subsequently infected with 5 MOI KAN-1 for the indicated time points. **E)** Expression of viral proteins PB1, PB2, NP, M1 and NS1 was analyzed by Western blot. Overexpression of PB2_**∆**_ was confirmed by using Myc-specific antibodies. Equal loading was verified by detection of total ERK2. **F)** Expression levels of viral m/cRNAs were detected by qRT-PCR and are depicted as mean *n*-fold (±SD) of one representative out of two independent experiments normalized to 15 min infection of empty vector-transfected cells. *p≤0.05, two-way ANOVA, Sidak’s multiple comparisons test. **G)** A549 cells were infected with 5 MOI KAN-1 for 8 h. Subsequently, co-immunoprecipitation of the viral polymerase complex was performed by using NP-specific antibodies. Efficient immunoprecipitation was confirmed by detection of viral proteins NP, PB1 and PB2. Presence of PB2_**∆**_ was analyzed by PB2-specific antibodies. **B, C, E, G)** Blots are representative of three independent experiments.

Internal deletion of nucleotides within influenza segments during the synthesis of defective RNAs in general results in truncated RNAs possessing all important sequences for transcription and translation. Therefore, the presence of an intact open reading frame (ORF) can result in the expression of polypeptides from subgenomic RNAs, as previously described [20, 21]. However, so far there are no studies delineating a potential function of these polypeptides in IAV infection. The PB2_∆_ RNA comprises the PB2 translational initiation codon followed by a frameshift downstream of the junction site, leading to early termination after 15 additional amino acid codons (Fig 2A). To test whether this potential ORF leads to the generation of a polypeptide from the PB2_∆_ mRNA, viral protein expression was analyzed. Antibodies directed against the PB2 N-terminus were used, a region that would be identical in both proteins. A distinct protein band around 10 kDa emerged 5 h p.i. that was not observed upon infection with other IAV isolates of various subtypes (Fig 2B). Furthermore, the existence of this protein in KAN-1-infected cells was confirmed by mass spectrometry (S2 Fig). To verify that it is encoded by PB2_∆_ mRNA, a specific siRNA was designed covering the conserved regions flanking the junction site (Fig 1A) thus limiting interference with viral PB2 RNA. Viral protein expression was analyzed in the presence or absence of PB2_∆_-specific siRNA (Fig 2C). As expected, the small PB2-like protein encoded by the PB2_∆_ RNA was down-regulated, whereas the expression of PB2 was not reduced. Interestingly, especially 5 h p.i. there was a slight increase in viral protein expression observable when PB2_∆_ was knocked down which might suggest a function of the small protein in viral protein expression. Another explanation would be a simultaneous knockdown of PB2_∆_ v/cRNA leading to a more efficient replication of KAN-1 due to the prevention of non-infectious particles. To analyze whether viral replication is affected by siRNA-mediated knockdown of PB2_∆_ RNA species, A549 cells were infected with KAN-1 for 8 h (Fig 2D, *above*). Although the KAN-1 isolate is well adapted to cell culture and replicates efficiently in A549 to high viral titers, there was a slight increase in viral replication observable when PB2_∆_ was knocked down (Fig 2D, *below*). Furthermore, expression of PB2_Δ_ protein was compared in rKAN-1 WT and rKAN-1 PB2_Δ_ infection. As expected, the PB2_Δ_ protein was only detectable in rKAN-1 PB2_Δ_-infected cells concomitant with decreased expression levels of other viral proteins (S3 Fig).

Thus, the viral PB2_∆_ defective RNA is efficiently transcribed into mRNA that is translated to a truncated 10 kDa protein. The data further suggest that this polypeptide might fulfill an antiviral function in influenza virus replication.

In order to analyze a potential function of the PB2_∆_ protein, an expression vector containing the PB2_∆_ ORF was generated. Initially, viral protein expression was analyzed in the presence or absence of PB2_∆_ in A549 cells infected with KAN-1. Interestingly, overexpression of PB2_∆_ led to a significant reduction in viral protein expression (Fig 2E). This could be attributed at least in part to the fact that the expression of viral mRNAs such as PB2 and M1 was reduced in the presence of PB2_∆_ (Fig 2F), although not significant for PB2 and effects on protein levels were more pronounced (Fig 2E).

Due to its function as part of the viral polymerase complex, the IAV protein PB2 has been linked to viral pathogenicity and host adaptation [36, 37]. There are several mutations known that verifiably affect viral polymerase activity and thus have an impact on viral replication [38, 39]. Comparative analysis of the viral proteins PB2 and PB2_∆_ showed sequence identity within the N-termini (Fig 2A), comprising the binding sites for the viral PB1 protein. Nuclear interaction with PB1 is essential for the generation of an active viral polymerase complex. Therefore, the decreased expression of viral mRNAs might be due to a reduced viral polymerase activity caused by direct interaction of PB2_∆_ with the polymerase complex. To test this hypothesis, co-immunoprecipitation of the viral polymerase complex was performed by using antibodies specific for the viral nucleoprotein (NP). The polymerase subunits PB1 and PB2 were efficiently co-immunoprecipitated, whereas PB2_∆_ was only detectable in the input control (Fig 2G). Therefore, reduced levels of viral mRNA expression in the presence of PB2_∆_ are unlikely to be caused by a modulation of viral polymerase activity induced by direct interaction of PB2_∆_ with the polymerase complex.

### PB2_∆_ interacts with the adapter protein MAVS

Another more indirect mode of interference with viral protein expression would be the enhancement of the IAV-induced antiviral host cell response. One of the most potent mediators of this response is IFNβ. Beside its role in viral replication, PB2 also participates in the evasion of the antiviral immune response mediated by direct interaction with the mitochondrial antiviral signaling protein (MAVS) [40]. The latter is of particular importance in the RIG-I-mediated signal transduction upon detection of viral RNA which results in the expression of type I IFNs [41–43]. It has been shown that the protein-protein interaction of MAVS and PB2 is mediated by N-terminal amino acids 1–37 of the viral PB2 protein [44] which are present in PB2_∆_ (Fig 2A). Therefore, an interaction with the cellular MAVS protein affecting IAV-induced IFNβ expression is likely to occur.

**Fig 3:**
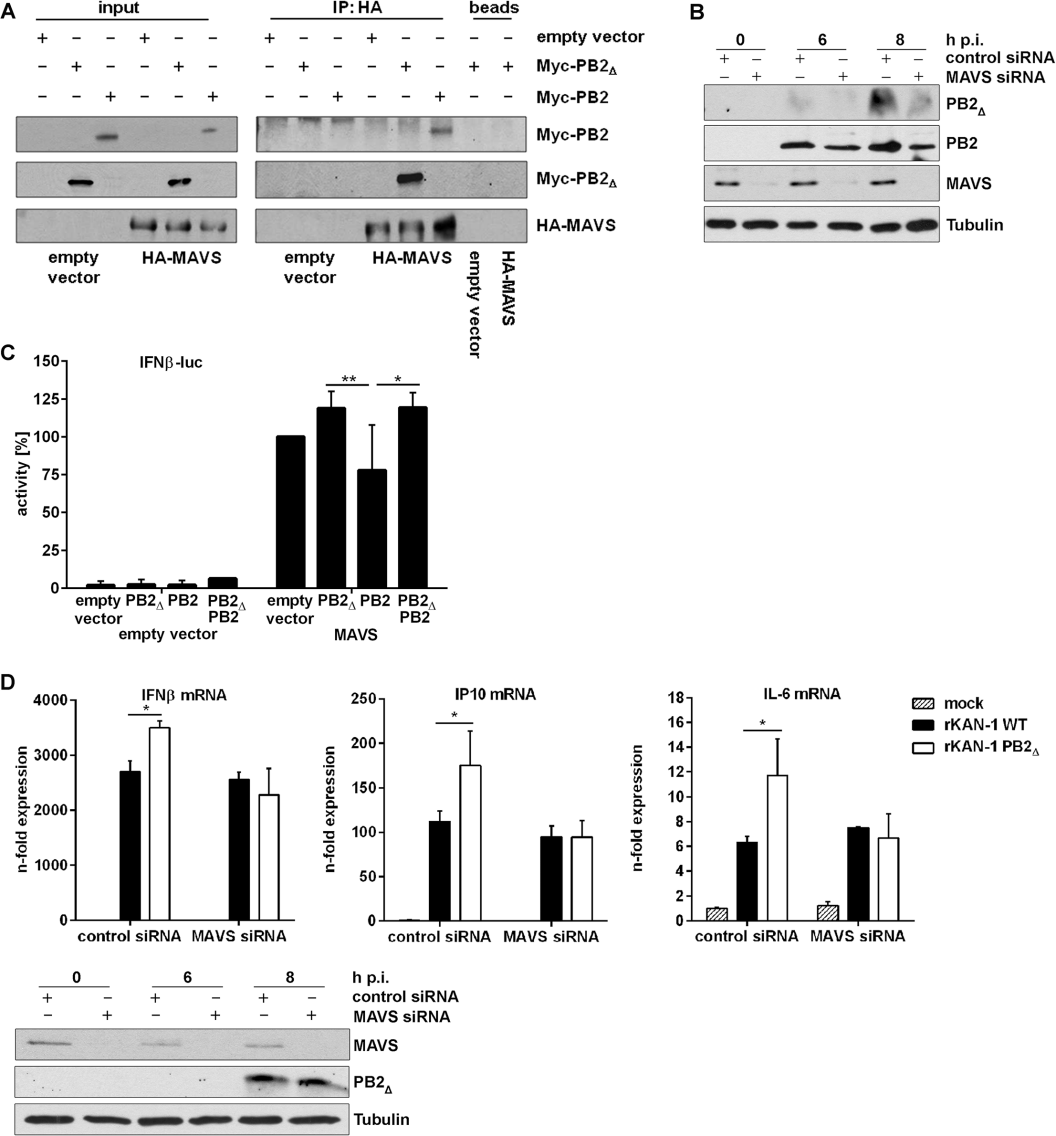
PB2_∆_ interacts with MAVS at mitochondria thereby inducing IFNβ expression. **A)** Hek293 cells were transfected with HA-MAVS or empty vector in combination with Myc-PB2_**∆**_, Myc-PB2 or empty vector. 24 h p.t. co-immunoprecipitation was performed by using HA-specific antibodies. Analysis of the co-immunoprecipitation of PB2 and PB2_**∆**_ was performed by detection of the Myc-tag. Blots are representative of three independent experiments. **B)** A549 cells were transfected with MAVS-specific or scrambled siRNA and subsequently infected with 5 MOI KAN-1. At the indicated time points, mitochondria were isolated and the presence of viral proteins PB2_**Δ**_ and PB2 was analyzed by Western blot. Knockdown efficiency was verified by using MAVS-specific antibodies. Tubulin expression served as loading control. **C)** Hek293 cells were transfected with the IFNβ promoter in combination with HA-MAVS or empty vector as well as with PB2_**∆**_, PB2, PB2_**∆**_/PB2 or empty vector. 24 h p.t. promoter activity was measured by luciferase assay. Depicted are mean percentages (±SD) of four independent experiments. *p≤0.05, **p≤0.01; two-way ANOVA, Tukey’s multiple comparisons test. **D)** A549 cells were transfected with MAVS-specific or scrambled siRNA and subsequently infected with 0.5 MOI rKAN-1 WT or PB2_**Δ**_ for 24 h. Expression levels of cytokines and ISGs were detected by qRT-PCR and are depicted as mean *n*-fold (±SD) of one representative out of three independent experiments normalized to non-infected control cells. *p≤0.05, two-way ANOVA, Sidak’s multiple comparisons test. Knockdown efficiency was verified by Western blot analysis using MAVS-specific antibodies. Presence of PB2_**∆**_ was analyzed by PB2-specific antibodies and Tubulin expression served as loading control. Blots are representative of two independent experiments.

To test this hypothesis, HEK293 cells were transfected with plasmids expressing HA-tagged MAVS in combination with expression plasmids for Myc-tagged PB2_∆_ or Myc-tagged PB2, respectively. 24 h p.t. HA-tagged MAVS was precipitated with HA-specific antibodies and the presence of co-precipitating proteins was analyzed. As expected, co-precipitation with cellular MAVS was confirmed not only for viral PB2 but also for the PB2_∆_ protein (Fig 3A).

It has been shown that the IAV PB2 protein exhibits an N-terminal mitochondrial-targeting signal, leading to the association of PB2 with mitochondria where it can interact with MAVS which needs mitochondrial localization to fulfill its signaling functions [40, 42, 45]. To analyze whether PB2_Δ_ is also associated with mitochondria and whether this localization is dependent on the presence of MAVS, mitochondria were isolated from MAVS siRNA-transfected A549 cells that were subsequently infected with KAN-1. As expected, knockdown of MAVS led to a reduced mitochondrial localization of PB2_Δ_ that was even more pronounced compared to that of viral PB2 protein (Fig 3B).

To analyze whether this interaction might affect MAVS-induced IFNβ expression, reporter gene assays were performed. A construct containing the luciferase gene under control of the entire IFNβ promoter harboring all transcription factor binding sites responsible for the formation of the IFNβ enhanceosome was used. As expected, empty vector-transfected cells exhibited no luciferase activity when viral proteins were co-expressed (Fig 3C). The presence of the MAVS protein led to a strong induction of the IFNβ promoter activity that was even increased when PB2_∆_ was co-expressed. In clear contrast, the full-length PB2 protein resulted in a decreased promoter activity, confirming earlier findings [40]. Interestingly, the presence of PB2_∆_ in PB2/MAVS-expressing cells compensated for the PB2-mediated decrease in IFNβ promoter activity, resulting in the same level of luciferase expression as observed for MAVS/PB2_∆_-expressing cells. This MAVS-mediated mechanism of antiviral gene expression induced by PB2_Δ_ was confirmed on endogenous levels by siRNA-mediated knockdown of MAVS. In this MAVS deficient situation, infection with a PB2_Δ_-positive virus led to the same levels of IFNβ and ISG mRNAs as observed in WT virus infection (Fig 3D). Since MAVS does not only participate in IFN induction but leads to the activation of different transcription factors inducing the expression of pro- and anti-inflammatory cytokines [41–43, 46], the expression of IL-6, a primarily NFκB-dependent cytokine, was analyzed in the presence of PB2_Δ_. As expected, IL-6 mRNA expression was also increased in presence of PB2_Δ_ showing the same dependency on MAVS signaling as observed for IFN and ISGs, suggesting that PB2_Δ_ leads to the activation of the full set of MAVS effector functions.

The results collectively demonstrate that PB2_∆_ can interact with MAVS, forming a functional complex that induces the expression of IFNβ, ISGs and other cytokines.

### PB2_∆_ induces the expression of IFNβ, thereby restricting viral replication

It is well known that influenza virus proteins such as the nonstructural proteins NS1 and PB1-F2 or the components of the heterotrimeric polymerase complex modulate IFNβ induction [26, 27, 47, 48], although in an inhibitory manner.

**Fig 4:**
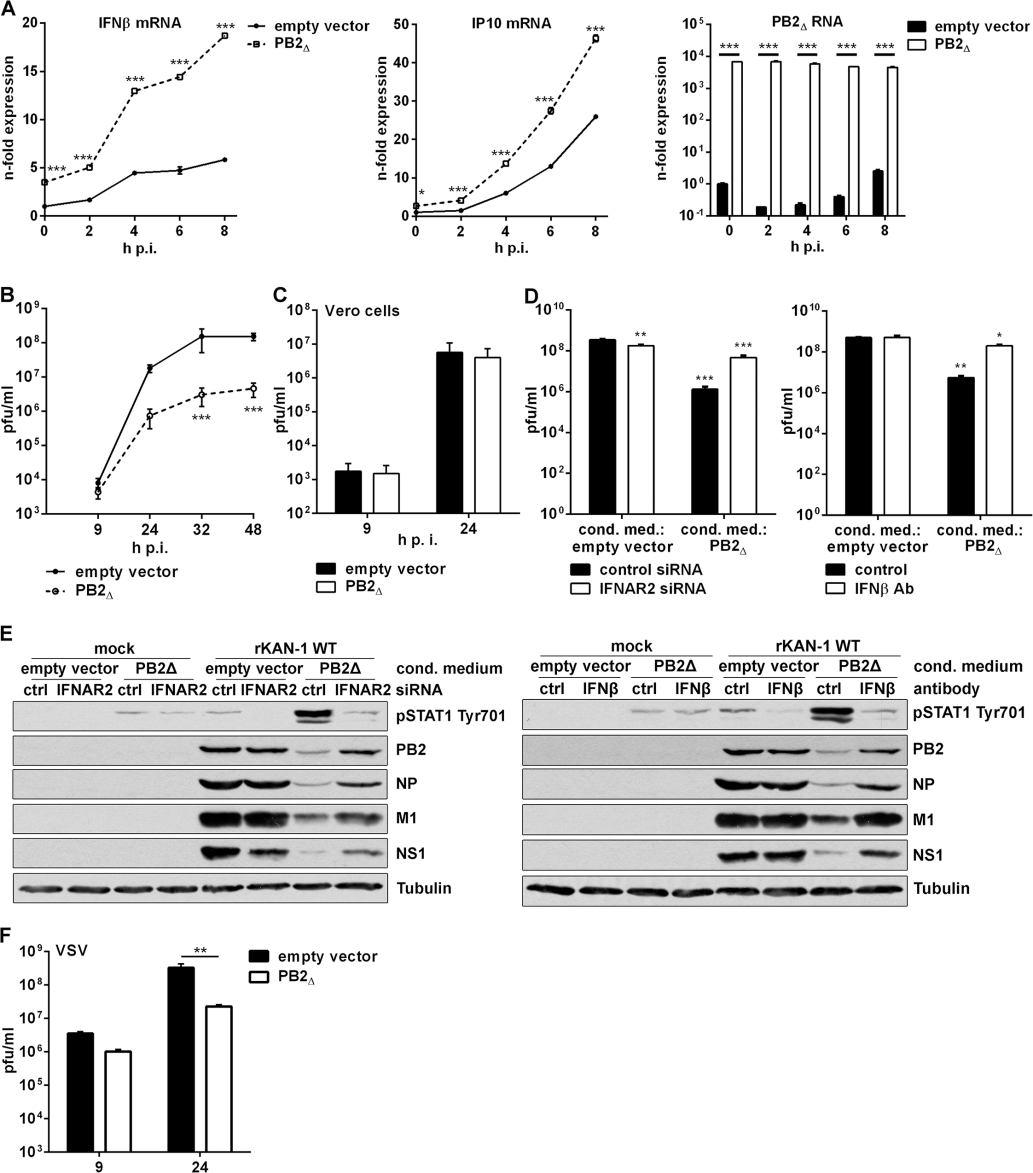
PB2_∆_ increases virus-induced expression of IFNβ, thereby restricting viral replication of IFN-sensitive viruses. **A)** A549 cells were transfected with PB2_**∆**_ or empty vector and subsequently infected with 5 MOI KAN-1. At the time points indicated total RNA was isolated and expressional changes of IFNβ or IP10 mRNAs were analyzed by qRT-PCR and are depicted as mean *n*-fold (±SD) of one representative out of two independent experiments normalized to empty vector-transfected control. Efficient transfection was confirmed by detection of PB2_**∆**_ mRNA. *p≤0.05; ***p≤0.001; two-way ANOVA, Sidak’s multiple comparisons test. **B, F)** A549 or **C)** Vero cells were transfected with PB2_**∆**_ or empty vector and subsequently infected with **B)** 0.01 MOI KAN-1, **C)** 0.001 MOI KAN-1 or **F)** 0.01 MOI VSV. At the time points indicated virus-containing supernatants were harvested and infectivity titers were determined by a standard plaque assay and are depicted as mean (±SD) of three independent experiments. **p≤0.01, ***p≤0.001; two-way ANOVA, Sidak’s multiple comparisons test. **D, E)** A549 cells were transfected with PB2_**Δ**_ or empty vector and 32 h p.t. conditioned media were transferred to IFNAR2 siRNA-transfected A549 cells or were enriched with neutralizing IFNβ antibody and transferred to untreated A549 cells. 16 h p.s. acceptor cells were infected with 0.1 MOI rKAN-1 WT for 24 h. **D)** Infectivity titers were determined by standard plaque assay and are depicted as mean (±SD) of one representative out of three independent experiments. *p≤0.05; **p≤0.01; ***p≤0.001; two-way ANOVA, Dunnett’s multiple comparisons test relating to empty vector-conditioned media-treated control cells, respectively. **E)** Expression of viral proteins PB2, NP, M1 and NS1 in conditioned medium-treated acceptor cells upon IFNAR2 knockdown or IFNβ neutralization was analyzed by Western blot. Knockdown and neutralization efficiencies were verified by using phosphor-STAT1 Tyr701 antibodies. Tubulin expression served as loading control. Blots are representative of three independent experiments.

To analyze whether overexpression of PB2_∆_ protein would interfere with IAV-mediated expression of IFNβ and alter the induction of interferon-stimulated genes (ISGs), KAN-1-induced mRNA levels of different antiviral acting genes were analyzed (Fig 4A). Indeed, overexpression of PB2_∆_ led to a strong interference with IFNβ mRNA induction that was detectable even in mock-infected cells, however, expression of the cytokine was strongly increased rather than inhibited. According to this, the expression of ISGs such as IP10 was also enhanced.

To gain insight into the role of PB2_∆_-induced stimulation of the innate immune response in viral replication, infectivity titers were determined in the presence or absence of PB2_∆_. KAN-1 replication was significantly reduced by more than 10-fold when PB2_∆_ was overexpressed (Fig 4B). Furthermore, this impaired viral propagation was most likely a consequence of the induction of type I IFN as demonstrated by infection of type I IFN-deficient Vero cells, where PB2_∆_ expression did not decrease infectivity titers (Fig 4C). This was confirmed by pretreatment of A549 cells with conditioned media of PB2_Δ_-transfected cells leading to a significant decrease in infectivity titers around 100-fold (Fig 4D). This suppression was recovered by specific knockdown of IFNα/β receptor chain 2 (IFNAR2) in the acceptor cells or enrichment of conditioned media with neutralizing IFNβ antibodies. Efficient interference with PB2_Δ_-induced type I IFN is demonstrated by reduced STAT1 Tyr701 phosphorylation which led to recovery of viral protein expression (Fig 4E). To analyze whether this PB2_∆_-dependent IFN induction is sufficient to inhibit replication of other IFN-sensitive viruses as well, A549 cells were infected with vesicular stomatitis virus (VSV), showing significantly reduced replication in the presence of PB2_∆_ (Fig 4F).

In summary, the defective RNA-encoded protein PB2_∆_ acts antiviral by the induction of type I IFN and ISGs, thereby inhibiting viral propagation of IFN-sensitive viruses.

### Presence of PB2_Δ_ leads to a more severe *in vivo* phenotype by enhancing early innate immune responses

**Fig 5:**
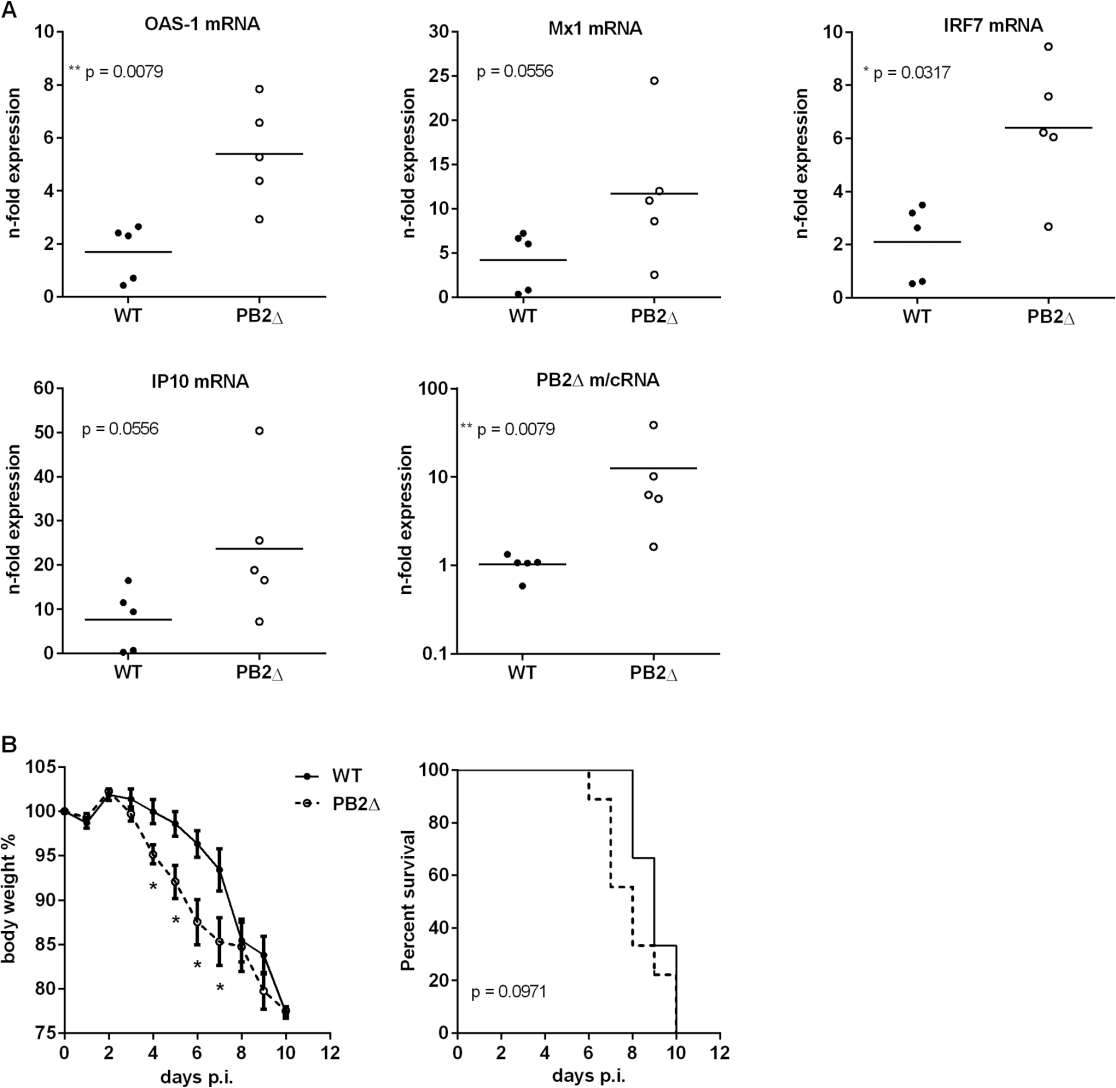
Effects of PB2_Δ_ on viral pathogenesis *in vivo*. BALB/c mice were infected with 50 pfu rKAN-1 wild type or PB2_**Δ**_-expressing virus. **A)** Expression changes of ISG mRNAs in lungs were analyzed two days p.i. by qRT-PCR. N-fold expression in individual animals normalized to uninfected PBS control mice is depicted. *p≤0.05, **p≤0.01: Mann-Whitney test. **B)** Body weight curves; mean % body weight of 1–9 (initial group size) animals normalized to initial weight +/-SEM is depicted. *p-values range between 0.014 and 0.049, Mann-Whitney test. Animals were excluded from the analysis when reaching less than 80% of the initial body weight. Survival curves; %-survival of 1–9 (initial group size) animals is depicted. Statistical significance was analyzed by Gehan-Breslow-Wilcoxon test.

The induced cytokine storm during severe influenza infections leads to major morbidity and mortality. A significant association between excessive early cytokine response, immune cell recruitment and poor outcome has been documented for highly pathogenic avian H5N1 virus infections [49]. To investigate the role of PB2_Δ_ in the induction of the innate immune response *in vivo*, BALB/c mice were infected with 50 pfu rKAN-1 WT in comparison to a PB2_Δ_-positive virus. Two days post infection, mouse lungs were extracted and total RNA was isolated for qRT-PCR. Fig 5A shows lung ISG mRNA levels normalized to PBS-treated control mice. Although differences in IFNβ mRNA levels between PB2_Δ_-negative and—positive KAN-1 viruses were not significant at the time point analyzed (S4A Fig), different ISGs such as OAS-1 and IRF7 showed considerably increased expression levels in presence of PB2_Δ_, while virus replication was not significantly altered at that time (S4B Fig).

Dysregulation of the innate cytokine response indicates disease severity and death during HPAIV infection [49, 50]. To analyze whether PB2_Δ_-induced ISG expression has an impact on disease severity of KAN-1 infection, BALB/c mice were infected with 50 pfu of wild type or PB2_Δ_-expressing virus. While in presence of PB2_Δ_ mice lost body weight as early as 4 days post infection, weight loss in WT-infected mice was delayed and was not detectable until day 6 p.i. (Fig 5B, left). This is supported by the survival curves which show the tendency to earlier death of PB2_Δ_-infected mice, although this result is not significant.

These findings demonstrate that defective RNA-encoded PB2_Δ_ protein-induced amplification of early innate immune responses leads to enhanced disease severity in KAN-1 infection and impressively emphasizes the role defective RNA-encoded proteins could play in viral pathogenicity of IAV.

## Discussion

The generation of IAV defective interfering particles upon serial passages at high multiplicity of infection *in vitro* has been well described and in recent years there is increasing evidence that influenza defective RNAs are naturally occurring also *in vivo* [16]. It is still discussed why non-infectious particles are produced upon infection in many or possibly all animal virus systems and defective viruses have been ignored as possible important determinants in the outcome of natural virus infections for years [9]. Generation of non-infectious particles might be induced by the host, thereby restricting viral replication and boosting innate immune responses. One further possibility is that DI particles have evolved as an adaptive measure of the virus, thereby enabling persistent infections to allow further spread in a population, especially in the case of highly pathogenic viruses whose infections are associated with high mortality rates. Furthermore, the non-infectious particle population contains transcriptionally competent gene segments that can be complemented through coinfection [51]. Natural infection of an animal host involves interplay between physically and genetically heterogeneous virions and a mixed collection of wildly different cell types of varying susceptibility in combination with a dynamic and complex immune response. Therefore, genetic variability induced by error-prone polymerases is an evolutionary advantage that is strongly promoted by non-infectious particles [52]. Recently, Saira and colleagues showed that *in vivo*-derived DI-like RNAs were similar to those generated *in vitro* and that the presence of identical DI-like RNAs in patients linked by direct contact is compatible with transmission between them [16]. Therefore, a functional role of DI-like RNAs in natural infections seems not unlikely. Here, we investigated the molecular mechanisms by which defective particles, harboring a specific PB2-derived subgenomic RNA that encodes a 10kDa protein, inhibit H5N1 KAN-1 viral replication.

Beside DIP-induced interference mediated by competition for viral polymerases and preferential packaging of over-abundant DI segments [12, 13], recent reports show that subgenomic RNAs can act as potent immune stimulators [17, 19, 32, 33]. It has been observed that the cytoplasmic PRR RIG-I preferentially binds small genomic and subgenomic RNAs [17] and there is evidence that the appearance of defective RNAs coincides with the production of cytokines during IAV infection [19]. Here, overexpression of the defective RNA-encoded protein PB2_∆_ *per se* was sufficient to induce transcription of IFNβ mRNA, whereupon subsequent infection led to a further increase in IFNβ expression. In addition, KAN-1-induced expression of IFNβ and subsequently that of ISGs seems to be primarily induced by the PB2_∆_ protein and not by the detection of the defective RNA itself. This was shown by the recombinant PB2_∆_-expressing virus which induced increased IFNβ levels only late upon infection although considerable expression of PB2_∆_ RNA was detectable already 2 h p.i.. These data suggest that not only defective RNA but also some defective RNA-encoded proteins can act immunostimulatory. Furthermore, the PB2_Δ_ protein-mediated IFN induction is completely independent of the detection of subgenomic RNA by the cellular sensor RIG-I but comprises complex formation with the adapter protein MAVS.

MAVS is of primary importance for RIG-I-mediated signal transduction upon sensing of viral PAMPs resulting in the expression of type I IFNs [41–43]. It has been shown that the PB2 protein interferes with IFNβ expression by direct interaction with MAVS via its binding site located within aa 1–37 [44]. It was hypothesized that PB2 binding leads to the inactivation of the MAVS complex by inhibition of intermolecular conformational changes needed for oligomerisation [44]. Here, PB2_∆_ binding of MAVS *per se* activates MAVS-mediated IFN induction. This suggests that not the binding of PB2 itself inhibits MAVS signaling but rather leads to a transition of the cellular protein into the active form, whereas the C terminal part of the viral protein interferes with induction of signal transduction. Thus, it is very likely that the C-terminus of PB2 inhibits MAVS oligomerisation or the interaction with downstream acting kinases such as TBK-1, TAK-1 or IKK or different transcription factors like IRF3 or IRF7 which are normally recruited by the adapter protein [41, 43, 46].

Interestingly, there was no interaction of PB2_∆_ with the viral polymerase complex observable although the binding site within PB2 mediating the interaction with MAVS is also involved in PB1 binding [44, 53]. This might be attributed to the tertiary structure of PB2_∆_. The binding site for MAVS and PB1 consists of 3 α-helices, of which the first establishes the interaction between PB1 and PB2 [53], whereas in the case of MAVS, helix 3 carries this function [44]. Therefore, specific folding of PB2_∆_ or steric inhibition by its C-terminus might lead to masking of helix 1, thereby inducing preferential binding of MAVS or even disabling PB1 binding. One further possibility would be the primary mediation of the PB1-PB2 interaction by an alternative PB1 binding site identified within the C-terminus of the PB2 protein [54]. Therefore, the present study reveals that functional analysis of defective RNA-encoded proteins can help to understand the biological characteristics of the parental viral protein and its interactions with viral and/or host factors.

The interaction of MAVS and PB2_Δ_ at mitochondria increases IAV-induced type I IFN expression leading to reduced infectivity titers in the presence of PB2_∆_. Therefore, the present study links for the first time a biological function to an IAV defective RNA-encoded protein. In addition, PB2_∆_ is an antiviral acting protein whose action is not restricted to influenza A virus infection, but also limits the replication of other IFN-sensitive viruses as shown by VSV infection. Therefore, this peptide or a low-molecular peptidomimetic thereof might be useful as antiviral agent for IFN-sensitive viruses.

Interestingly, sequence alignments of human and avian H5N1 isolates showed that 53% or 79%, respectively, harbor the short sequence repeat at the junction site as observed for PB2_∆_ RNA which could be responsible for polymerase skipping, suggesting that these IAV isolates might also evolve a defective RNA similar to KAN-I PB2_∆_ upon passaging and/or transmission *in vivo*. This was supported by the finding that serial undiluted passaging of PB2_Δ_-free propagations of H5N1 strains KAN-1 and Vietnam led to the *de novo* generation of PB2_Δ_ RNA to various extents. These data highlight different expression patterns of the same RNA within different preparations of the same virus strain and possibly explains different phenotypes of the same virus strain propagated in different laboratories.

Whether there are additional *cis*- or *trans*-mutations needed to facilitate generation of these defective RNAs has to be further studied. The apparent late expression of the PB2_∆_ protein compared to its RNA might suggest that there are other sequences or structures needed that support the translation of PB2_∆_.

In summary, PB2_∆_ is a virulence factor in H5N1 KAN-1 infections that restricts viral replication by modulation of the antiviral host gene response. Infections with highly pathogenic avian influenza viruses are characterized by a hyperactivation of the host immune response leading to an excessive expression of proinflammatory cytokines. This so called ‘cytokine burst’ has been discussed to be decisive for disease outcome [49, 50, 55]. Therefore, although PB2_∆_ is an antiviral acting protein in cell culture, its presence in KAN-1 infections *in vivo* enhances the induction of innate immune responses and thereby is involved in increased pathogenicity. Graef and colleagues hypothesized that the reduced PB2-induced suppression of MAVS-mediated signal transduction upon infection with avian influenza viruses in comparison to human subtypes might play a role in HPAIV-induced dysregulation of cytokine expression [40]. The adapter protein MAVS is not only essential for the induction of IFNβ but plays a role directly and indirectly in the induction of interferon-stimulated response element (ISRE)-regulated and proinflammatory genes by activation of transcription factors IRF3 (interferon regulatory factor 3) and NF-κB (nuclear factor kappa-light-chain-enhancer of activated B-cells) [41–43, 46]. In this context, it has already been shown that the early suppression of cytokine amplification significantly leads to the protection of mice from lethal IAV infection [56, 57]. Therefore, in context of KAN-1 infection, targeting PB2_∆_ might be a promising approach to reduce disease manifestation by the suppression of immunopathology.

Overall, the present study provides evidence for the very first time that polypeptides encoded by influenza virus defective RNAs can fulfill biological functions that are associated but must not coincide with the activities of the parental full-length protein. Furthermore, subgenomic RNA-encoded polypeptides seem to play a prominent role in influenza virus pathogenicity, therefore the analysis of their functions leads to deeper understanding of the biology of influenza virus infections and its interactions with the host.

## Materials and Methods

### Ethics statement

All animal studies were performed in compliance with animal welfare regulations of the German Society for Laboratory Animal Science (GV-SOLAS) and the European Health Law of the Federation of Laboratory Animal Science Associations (FELASA). The protocol was approved by the relevant authorities in Hamburg (Behörde für Gesundheit und Verbraucherschutz) under the license number Az 42/13.

### Viruses and cells

A/Thailand/KAN-1/2004 (H5N1; KAN-1) was used with kind permission from P. Puthavathana (Bangkok, Thailand). Recombinant A/Vietnam/1203/2004 (H5N1; Vietnam) and A/Puerto-Rico/8/34 (H1N1; PR8) were described before [58, 59]. A/FPV/Bratislava/79 (H7N7; fowl plaque virus, FPV) was a kind gift from S. Pleschka (Institute of Virology, Giessen, Germany) and A/Mallard/Bavaria/1/2006 (H5N1, Mallard) was obtained from O. Planz (Interfaculty Institute for Cell Biology, Tübingen, Germany). All experiments and handling of samples containing H5N1 or H7N7 infectious particles were performed in a biological safety level 3 containment. Influenza viruses as well as vesicular stomatitis virus strain Indiana (VSV) were propagated on Madin-Darby canine kidney (MDCKII) cells cultured in minimal essential medium (MEM, PAA Laboratories) containing 10% v/v FCS (Invitrogen) as described elsewhere [25]. Human alveolar epithelial cells (A549), green monkey epithelial cells (Vero) and human embryonic kidney 293 cells (HEK293) were cultured in Dulbecco’s modified eagle medium (DMEM, PAA Laboratories) containing 10% v/v FCS. All cell lines were originally purchased from ATCC and have been passaged in the laboratory. At regular intervals cells are checked for their identity by SNP-profiling (Multiplexion).

### Plasmids

The luciferase reporter construct pTATA-IFNβ-luc containing the whole IFNβ enhanceosome upstream of a luciferase gene was a kind gift from J. Hiscott (Vaccine & Gene Therapy Institute of Florida, Port Saint Lucie, Florida, USA). pHW2000-PB2_∆_ was obtained by reverse transcription of the A/Thailand/KAN-1/2004 PB2_∆_ DI RNA by using influenza A segment one specific universal 12 primers [60] and subsequent PCR amplification. PB2_∆_ DI DNA was then cloned into the bidirectional pHW2000 plasmid [30] by using Eco31I restriction enzyme. pcDNA3-6xMyc-PB2_∆_ was obtained by PCR amplification of the open reading frame of PB2_∆_ that was subsequently cloned into pcDNA3-6xMyc by using EcoRV and XhoI restriction enzymes. Cloning of the PB2 gene into pcDNA3-6xMyc was performed the same way by Ludmilla Wixler (Institute of Molecular Virology, Muenster, Germany). Primer sequences are included in S4 Table. pCAGGS-HA-MAVS was a kind gift from B. Dauber (Robert Koch Institute, Berlin, Germany).

### Generation of recombinant viruses

A set of eight plasmids based on the bidirectional pHW2000 plasmid allowing the rescue of the recombinant wild-type (WT) of influenza A/Thailand/KAN-1/2004 was used with kind permission from J. Stech (Friedrich-Loeffler-Institute, Riems, Germany). The generation of the recombinant viruses was carried out as described elsewhere [25, 30].

### Plaque titration

Plaque forming units of a given virus suspension were determined by a standard plaque assay as described earlier [61].

### Hemagglutination assay

Serial 2-fold dilutions of virus supernatants (1x10^6^ infectious particles, according to 1 HA unit of rKAN-1 WT stock) were prepared in V-bottomed microtiter plates in a total volume of 50 μl PBS, the latter also serving as negative control. 50 μl of solution of 1% chicken erythrocytes (Rockland Immunochemicals, Inc) in PBS were added to the wells and microtiter plates were incubated for 30 min at 4°C. Hemagglutination was monitored by photography.

### Transfection of siRNA and plasmid DNA

PB2_∆_ siRNA (5’-AGGAAUAGGAAUGAGAAUA-3’), MAVS siRNA (5’-GCUGAAGACAAGACCUAUA-3’), IFNAR2 siRNA (5’-GAAGCAUAAACCCGAAAUA-3’) and control siRNA (5’-UUCUCCGAACGUGUCACGU-3’) were synthesized by MWG-Biotech AG. Transfection with PB2_Δ_ siRNA was performed with HiPerFect (Qiagen), MAVS and IFNAR2 siRNAs were transfected by the use of Lipofectamine 2000 (Invitrogen) according to the manufacturer’s protocols. Infections were carried out 16–48 h p.t. and conditioned medium experiments 36 h p.t.. Plasmid DNA was transfected using Lipofectamine 2000 (Invitrogen) consistent with manufacturer’s instructions and infections were carried out 24 h p.t..

### Western blot analysis and antibodies

Cells were lysed in radioimmunoprecipitation assay (RIPA) buffer containing protease and phosphatase inhibitors [61]. Mitochondria were isolated by using the Mitochondria Fractionation Kit from BioVision according to the manufacturer’s protocol. RIPA protein lysates were cleared by centrifugation, lysates were separated by SDS-PAGE and blotted onto nitrocellulose membranes. Antisera directed against ERK2 (C-14) and influenza A PB1 (vK-20) were purchased from Santa Cruz Biotechnology and anti-α-Tubulin from Sigma-Aldrich. Antibody against influenza A PB2 protein was a kind gift of E. Fodor (Sir William Dunn School of Pathology, Oxford, UK [45]. Influenza A M1 and NP antibodies were obtained from AbD Serotec and mouse monoclonal antibody against influenza A NS1 (23–1) was developed at the Institute of Molecular Virology (Muenster, Germany) and can be purchased from Santa Cruz Biotechnology. MAVS (AT107) antibody was obtained from Alexis Biochemicals and anti-Myc (9E10) from ATCC. Antibody against phosphor-STAT1 Tyr701 (clone 14) was purchased from BD Transduction Laboratories. Neutralizing IFNβ antibody was obtained from Abcam and used in a concentration of 4 μg/ml conditioned medium.

### Mass spectrometry

For proteome analyses 100 μg of protein mixtures from KAN-1-infected A549 cells were separated on a one-dimensional gel, the lanes were cut into twelve slices each and in-gel digested by trypsin as described previously [62]. Peptide fractions were collected and desalted separately using C18 StageTips [63]. LC-MS/MS analyses were performed on an EasyLC nano-HPLC (Proxeon Biosystems) coupled to an LTQ Orbitrap Elite mass spectrometer (Thermo Scientific) as described previously [64]. Briefly, the peptide mixtures were injected onto the column in HPLC solvent A (0.5% acetic acid) at a flow rate of 500 nl/min and subsequently eluted with an 87-min segmented gradient of 5–90% HPLC solvent B (80% ACN in 0.5% acetic acid). During peptide elution the flow rate was kept constant at 200 nl/min. For proteome analysis the 20 most intense precursor ions were sequentially fragmented by CID in each scan cycle. Sequenced precursor masses were excluded from further selection for 90 s. The target values for the LTQ were 5000 charges (MS/MS) and 10^6^ charges (MS).

The MS data were processed using default parameters of the MaxQuant software (v1.2.2.9) [65]. Extracted peak lists were submitted to database search using the Andromeda search engine [66] to query a target-decoy databases [67] consisting of the uniprot human proteome database (88,692 protein entries, downloaded on the 25^th^ of February 2014), of H5N1 virus database (13 entries, including the sequence of PB2_Δ_) and 248 commonly observed contaminants. In database search, full tryptic specificity was required and up to two missed cleavages were allowed. Carbamidomethylation of cysteine was set as fixed modification; protein N-terminal acetylation, and oxidation of methionine were set as variable modifications. Mass tolerances were set to 6 ppm at the precursor and 0.5 Da at the fragment ion level, respectively. False discovery rates were set to 5% at peptide, and protein group level and the minimum peptide length was set to five amino acids.

### Co-immunoprecipitation

Cells were lysed with RIPA or Triton lysis (TLB) buffer [68] containing protease and phosphatase inhibitors. Lysates were cleared by centrifugation and supernatants were incubated o/n at 4°C with the antibodies indicated coupled to protein A/G-conjugated agarose (Roche). Complexes were washed three times with lysis buffer (5 min overhead shaking, 4°C) and resolved by SDS-PAGE with subsequent electrotransfer onto nitrocellulose membranes.

### Luciferase reporter gene assays

Transfection of HEK293 with the IFNβ luciferase reporter plasmid (0.25 μg) in combination with the different expression plasmids (1 μg in total) was performed with polyethylenimine (PEI) as described elsewhere [69]. Luciferase assays were carried out 24 h p.t. as previously described [70]. Relative light units were normalized to protein concentrations determined with a standard Bradford assay.

### RNA isolation, cDNA synthesis and qRT-PCR

Total RNA from cells was isolated using the RNeasy Kit (Qiagen) according to the manufacturer’s instructions. Lungs from mice were collected at the time points indicated and total RNA was isolated using TRIzol reagent (Invitrogen). TRIzol lysis was performed according to the manufacturer's protocol, introducing a secondary phase separation step as described previously [57].

Three micrograms of total RNA were reverse transcribed with RevertAid H Minus Reverse Transcriptase (MBI Fermentas) and oligo(dT) (MWG-Biotech AG) or random hexamer (Fermentas) primers according to the manufacturer’s protocol.

Strand-specific qRT PCR for distinguishing influenza v/c/mRNAs was performed as described previously [71]. Briefly, 200 ng of total RNA were reverse transcribed with RevertAid Premium Reverse Transcriptase (MBI Fermentas) and 10 pmol of each primer (specific for v/c/mRNAs and Oligo(dT)) according to the manufacturer’s instructions.

The cDNA was used for qRT-PCR, which was performed using a Roche LightCycler 480 and Brilliant SYBR Green III Mastermix (Agilent) according to the manufacturer’s instructions. Primer sequences are included in S4 Table. Relative changes in expression levels (*n*-fold) were calculated according to the 2^-∆∆CT^ method [72].

### Northern blot analysis

Total RNA (10 μg) was separated on 8% (w/v) polyacrylamide [29:1 acrylamide/bisacrylamide], 7 M urea gels and electro-transferred onto positively charged nylon membranes (Roche). Hybridization probes (50 pmol; PB2_∆_ vRNA: 5’-TGAAAGGAATAGGAATGAGAAT-3’; PB2_∆_ c/mRNA: 5’-ATTCTCATTCCTATTCCTTTCA-3’) were radioactively labeled using [γ-^32^P]-ATP (PerkinElmer) and T4 Polynucleotide Kinase (Fermentas) according to the manufacturer’s instructions. Northern blot analysis was performed as described previously [73].

### Mouse experiments

All animal experiments were performed in the BSL-3 animal facility of the Heinrich-Pette-Institute, Leibniz Institute in Hamburg. BALB/c mice were obtained from the Harlan-Winkelmann animal breeding facilities. Eight-week-old mice were anaesthetized by intraperitoneal injection of 100 mg/kg Ketavet and 10 mg/kg Xylazin. Mice were infected by the intranasal route in a 50 μL volume as indicated. Health status of the animals was monitored daily according to the animal protocols approved by the Hamburg authorities.

### Accession numbers

**Table:** 

GenBank	AAV35117	: polymerase basic protein 2 (PB2)
		: (A/Thailand/1 (KAN-1)/2004
	AAV35116	: polymerase basic protein 1 (PB1)
		: (A/Thailand/1 (KAN-1)/2004)
	AAV35113	: nonstructural protein 1 (NS1) (A/Thailand/1 (KAN-1)/2004)
	AAV35112	: nucleoprotein (NP) (A/Thailand/1 (KAN-1)/2004)
	AAV35110	: matrixprotein 1 (M1) (A/Thailand/1 (KAN-1)/2004)
	ABO21695	: polymerase basic protein 2 (PB2) (A/Vietnam/1203/2004)
	Q7Z434.2	: mitochondrial antiviral-signaling protein (MAVS)
	AAA58459.1	: extracellular signal-regulated kinase 2 (ERK2)
	CAG46616.1	: interferon alpha/beta receptor 2 (IFNAR2)
	ADA59516.1	: signal transducer and activator of transcription 1 (STAT1)
	AAM97604.1	: 2’-5’-oligoadenylate synthetase 1 (OAS-1) (*mus musculus*)
	CAJ18612.1	: Myxovirus resistance protein 1 (Mx1) (*mus musculus*)
	AAI38800.1	: interferon regulatory factor 7 (IRF7) (*mus musculus*)
Swiss-Prot	O95786.2	: retinoic acid-inducible gene I (RIG-I)
	P01574.1	: interferon beta
PRF	225751	: gamma interferon inducible gene IP10

## Supporting Information

S1 Table Multiple sequence alignment of PB2 mRNA fragments from known human H5N1 influenza A viruses. All sequences were obtained from NCBI’s Influenza Virus Resource (http://www.ncbi.nlm.nih.gov/genomes/FLU/FLU.html).
(PDF)

S2 Table Multiple sequence alignment of PB2 mRNA fragments from known avian H5N1 influenza A viruses. All sequences were obtained from NCBI’s Influenza Virus Resource (http://www.ncbi.nlm.nih.gov/genomes/FLU/FLU.html).
(PDF)

S3 Table Expressional changes of PB2_Δ_ RNA in multiple undiluted passages of plaque purified viral clones. MDCKII cells were infected with plaque-purified PB2_Δ_-free rKAN-1 WT or Vietnam clones for 16–24 h. Undiluted supernatants were transferred to untreated MDCKII cells for 5 passages. Total RNA was isolated from every passage and expressional changes of PB2_Δ_ RNA were analyzed by qRT-PCR and are depicted as *n*-fold normalized to first passage.
(PDF)

S4 Table List of primers used in the study. (PDF)

S1 Fig Expressional changes of PB2_Δ_ RNA species. A549 cells were infected with 5 MOI KAN-1 and total RNA was isolated at the time points indicated. Expressional changes of the different PB2_Δ_ RNA species were detected by qRT-PCR and are depicted as mean *n*-fold (±SD) of three independent experiments normalized to the respective RNA expression 2 h p.i..
(PDF)

S2 Fig MS/MS spectrum of the tryptic C-terminal unique peptide KGQFPCVQL of the H5N1 KAN-1 virus protein PB2_Δ_. 100 μg of protein mixtures from KAN-1-infected A549 cells were separated on a one-dimensional gel followed by in-gel digest by trypsin. LC-MS/MS analyses were performed on an EasyLC nano-HPLC (Proxeon Biosystems) coupled to an LTQ Orbitrap Elite mass spectrometer (Thermo Scientific). The MS data were processed using default parameters of the MaxQuant software (v1.2.2.9).
(PDF)

S3 Fig Expressional changes of viral proteins in presence of PB2_Δ_ protein. A549 cells were infected with 5 MOI of rKAN-1 WT or PB2_Δ_ for 2, 4, 6 or 8 h. Expression of viral proteins PB1, PB2, NP, M1 and NS1 was analyzed by Western blot. Presence of PB2_Δ_ was verified by using PB2-specific antibodies. ERK2 expression served as loading control. Blots are representative of three independent experiments.
(PDF)

S4 Fig Effects of PB2_Δ_ protein *in vivo*. BALB/c mice were infected with 50 pfu rKAN-1 wild type or PB2_Δ_-expressing virus. **A)** Expression changes of IFNβ mRNA in lungs were analyzed two days p.i. by qRT-PCR. N-fold expression in individual animals normalized to uninfected PBS control mice is depicted. **B)** Viral lung titers 2 days p.i. of individual animals are depicted. **A, B)** Statistical significance was analyzed by Mann-Whitney test.
(PDF)
