# Supplementary material for: Evidence for a Novel Mechanism of Influenza Virus-Induced Type I Interferon Expression by a Defective RNA-Encoded Protein
Source: PLoS Pathog. 2015 May 29;11(5):e1004924. doi: 10.1371/journal.ppat.1004924 (PMC4449196; doi:10.1371/journal.ppat.1004924)
Supplement: S1 Table — All sequences were obtained from NCBI’s Influenza Virus Resource (http://www.ncbi.nlm.nih.gov/genomes/FLU/FLU.html). (PDF) [file ppat.1004924.s001.pdf]

S1 Table. Multiple sequence alignment of PB2 mRNA fragments from known human H5N1 influenza A viruses.

All sequences were obtained from NCBI's Influenza Virus Resource (http://www.ncbi.nlm.nih.gov/genomes/FLU/FLU.html).

```
               222          235  240            255     1945      1956  1961    1970
                *            *    *              *       *          *    *        *
CY111595  5'-...GATGATTCCTGAAAGGAATGAACAAGGGCAGACG-------GTGAGAGGCTCAGGAATGAGAATACT...-3'
HM172413  5'-...GATGATTCCTGAAAGGAATGAACAAGGGCAGACG-------GTGAGAGGCTCAGGAATGAGAATACT...-3'
EU263988  5'-...GATGATTCCTGAAAGGAATGAACAAGGGCAGACG-------GTGAGAGGCTCAGGAATGAGAATACT...-3'
EU146846  5'-...GATGATTCCTGAAAGGAATGAACAAGGACAGACA-------GTTAGAGGCTCAGGAATGAGAATACT...-3'
HM114566  5'-...AATGATCCCTGAAAGGAATGAACAAGGGCAGACG-------GTGAGAGGCTCAGGAATGAGGATACT...-3'
HM114542  5'-...AATGATCCCTGAAAGGAATGAACAAGGGCAGACG-------GTGAGAGGCTCAGGAATGAGGATACT...-3'
HM114550  5'-...AATGATCCCTGAAAGGAATGAACAAGGGCAGACG-------GTGAGAGGCTCAGGAATGAGGATACT...-3'
HM114558  5'-...AATGATCCCTGAAAGGAATGAACAAGGGCAGACG-------GTGAGAGGCTCAGGAATGAGGATACT...-3'
GQ232441  5'-...AATGATCCCTGAAAGGAATGAACAAGGGCAGACG-------GTGAGAGGCTCAGGAATGAGGATACT...-3'
HM114582  5'-...AATGATCCCTGAAAGGAATGAACAAGGGCAGACG-------GTGAGAGGCTCAGGAATGAGGATACT...-3'
HM114614  5'-...GATGATTCCTGAAAGGAATGAACAAGGGCAGACG-------GTGAGAGGCTCAGGAATGAGAATACT...-3'
HM114606  5'-...AATGATCCCTGAAAGGAATGAACAAGGGCAGACG-------GTGAGAGGCTCAGGAATGAGGATACT...-3'
HM114598  5'-...AATGATCCCTGAAAGGAATGAGCAAGGGCAGACG-------GTGAGAGGCTCAGGAATGAGGATACT...-3'
CY088766  5'-...GATGATTCCTGAAAGGAATGAACAAGGACAGACA-------GTGAGAGGCTCAGGAATGAGAATACT...-3'
EU434701  5'-...GATGATCCCTGAACGGAATGAACAGGGACAAACG-------GTGAGAGGTTCAGGAATGAGAATACT...-3'
EU434693  5'-...GATGATCCCTGAACGGAATGAACAGGGACAAACG-------GTGAGAGGTTCAGGAATGAGAATACT...-3'
AF115291  5'-...GATGATCCCTGAAAGGAATGAGCAAGGTCAAACT-------GTGAGGGGATCAGGAATGAGAATACT...-3'
AF084261  5'-...GATGATCCCTGAAAGGAATGAGCAAGGTCAAACT-------GTGAGGGGATCAGGAATGAGAATACT...-3'
AF084262  5'-...GATGATCCCTGAAAGGAATGAGCAAGGTCAAACT-------GTGAGGGGATCAGGAATGAGAATACT...-3'
AF084263  5'-...GATGATCCCTGAAAGGAAGGAGCAAGGTCAAACT-------GTGAGGGGATCAGGAATGAGAATACT...-3'
AF115290  5'-...GATGATCCCTGAAAGGAATGATCAAGGTCAAACT-------GTGAGGGGATCAGGAATGAGAATACT...-3'
CY098659  5'-...GATGATTCCTGAAAGGAATGAACAAGGACAGACA-------GTGAGAGGCTCAGGAATGAGAATACT...-3'
CY098576  5'-...GATGATTCCTGAAAGGAATGAACAAGGGCAGACG-------GTGAGAGGCTCAGGAATGAGAATACT...-3'
CY098582  5'-...GATGATTCCTGAAAGGAATGAACAAGGGCAGACG-------GTGAGAGGCTCAGGAATGAGAATACT...-3'
CY098571  5'-...GATGATTCCTGAAAGGAATGAACAAGGGCAGACG-------GTGAGAGGCTCAGGAATGAGAATACT...-3'
CY098613  5'-...GATGATTCCTGAAAGGAATGAACAAGGGCAGACG-------GTGAGAGGCTCAGGAATGAGAATACT...-3'
CY098607  5'-...GATGATTCCTGAAAGGAATGAACAAGGGCAGACG-------GTGAGAGGCTCAGGAATGAGAATACT...-3'
CY098638  5'-...GATGATTCCTGAAAGGAATGAACAAGGGCAGACG-------GTGAGAGGCTCAGGAATGAGAATACT...-3'
CY098619  5'-...GATGATTCCTGAAAGGAATGAACAAGGGCAGACG-------GTGAGAGGCTCAGGAATGAGAATACT...-3'
CY098645  5'-...GATGATTCCTGAAAGGAATGAACAAGGGCAGACG-------GTGAGAGGCTCAGGAATGAGAATACT...-3'
CY098652  5'-...GATGATTCCTGAAAGGAATGAACAAGGGCAGACG-------GTGAGAGGCTCAGGAATGAGAATACT...-3'
CY098678  5'-...GATGGTTCCTGAAAGGAATGAACAAGGGCAGACG-------GTGAGAGGCTCAGGAATGAGAATACT...-3'
CY098692  5'-...GATGATCCCTGAAAGGAATGAACAAGGGCAGACG-------GTGAGAGGCTCAGGAATGAGGATACT...-3'
CY098672  5'-...GATGATTCCTGAAAGAAATGAACAAGGGCAGACG-------GTGAGAGGCTCAGGAATGAGAATACT...-3'
CY098706  5'-...GATGATTCCTGAAAGGAATGAACAAGGGCAGACG-------GTGAGAGGCTCAGGGATGAGAATACT...-3'
```

```
                   222            235  240              255      1945        1956  1961     1970
                    *              *    *                *        *           *     *        *
CY098734  5'-...GATGATTCCTGAAAGGAATGAACAGGGGCAGACA-------GTGAGGGGCTCAGGAATGAGAATACT...-3'
CY098748  5'-...GATGATTCCTGAAAGGAATGAACAGGGGCAGACA-------GTGAGGGGCTCAGGAATGAGAATACT...-3'
CY098720  5'-...GATGATTCCCGAAAGGAATGAACAAGGGCAGACA-------GTGAGGGGCTCAGGAATGAGAATACT...-3'
CY098727  5'-...TATGATTCCTGAAAGGAATGAACAAGGGCAGACG-------GTGAGAGGCTCAGGAATGAGAATACT...-3'
CY098755  5'-...TATGATTCCTGAAAGGAATGAACAAGGGCAGACA-------GTGAGGGGCTCAGGAATGAGAATACT...-3'
CY098665  5'-...GATGATCCCTGAACGGAATGAACAGGGACAAACG-------GTGAGAGGTTCAGGAATGAGAATACT...-3'
CY098625  5'-...GATGATCCCTGAACGGAATGAACAGGGACAAACG-------GTGAGAGGTTCAGGAATGAGAATACT...-3'
CY098631  5'-...GATGATCCCTGAACGGAATGAACAGGGACAAACG-------GTGAGAGGTTCAGGAATGAGAATACT...-3'
CY098594  5'-...GATGATCCCTGAACGGAATGAACAGGGACAAACG-------GTGAGAGGTTCAGGAATGAGAATACT...-3'
CY098588  5'-...GATGATCCCTGAACGGAATGAACAGGGACAAACG-------GTGAGAGGTTCAGGAATGAGAATACT...-3'
CY098685  5'-...GATGATCCCTGAACGGAATGAACAGGGACAAACG-------GTGAGAGGTTCAGGAATGAGAATACT...-3'
CY098713  5'-...GATGATCCCTGAACGGAATGAACAGGGACAAACA-------GTGAGAGGTTCAGGAATGAGAATACT...-3'
CY098699  5'-...GATGATCCCTGAACGGAATGAACAGGGACAAACG-------GTGAGAGGTTCAGGAATGAGAATACT...-3'
JN588925  5'-...GATGATCCCTGAAAGGAATGAACAAGGACAGACG-------GTGAGAGGCTCAGGAATGAGAATACT...-3'
JN588929  5'-...GATGATCCCTGAAAGGAATGAACAAGGACAGACG-------GTGAGAGGCTCAGGAATGAGAATACT...-3'
AB598126  5'-...GATGATTCCTGAAAGGAATGAACAAGGGCAGACG-------GTGAGAGGCTCAGGAATGAGAATACT...-3'
JQ714243  5'-...GATGATCCCTGAAAGGAATGAACAAGGACAGACG-------GTGAGAGGCTCAGGAATGAGAATACT...-3'
HQ664943  5'-...GATGATTCCTGAAAGGAATGAACAAGGGCAGACG-------GTGAGAGGCTCAGGAATGAGAATACT...-3'
EU146634  5'-...AATGATTCCTGAAAGGAATGAACAAGGGCAGACA-------GTGAGAGGCTCTGGAATGAGAATACT...-3'
AY651721  5'-...GATGATTCCTGAAAGGAATGAACAGGGGCAGACG-------GTGAGAGGCTCAGGAATGAGAATACT...-3'
JN588926  5'-...GATGATCCCTGAAAGGAATGAACAAGGACAGACG-------GTGAGAGGCTCAGGAATGAGAATACT...-3'
EF456776  5'-...GATGATTCCTGAAAGGAATGAACAAGGGCAGACG-------GTGAGAGGATCAGGAATGAGAATACT...-3'
EF467807  5'-...GATGATTCCTGAAAGGAATGAACAAGGGCAGACG-------GTGAGAGGCTCAGGAATGAGAATACT...-3'
EF467805  5'-...GATGATTCCTGAAAGGAATGAACAAGGGCAGACG-------GTGAGAGGCTCAGGAATGAGAATACT...-3'
EF467806  5'-...GATGATTCCTGAAAGGAATGAACAAGGGCAGACG-------GTGAGAGGCTCAGGAATGAGAATACT...-3'
AY576381  5'-...GATGATTCCTGAAAGGAATGAACAAGGGCAGACG-------GTGAGAGGCTCAGGAATGAGAATACT...-3'
GU052096  5'-...GATGATTCCTGAAAGGAATGAACAAGGGCAGACG-------GTGAGAGGCTCAGGAATGAGAATACT...-3'
GU052104  5'-...GATGATCCCTGAAAGGAATGAGCAAGGTCAAACT-------GTGAGGGGATCAGGAATGAGAATACT...-3'
GU052149  5'-...GATGATCCCTGAAAGGAATGAGCAAGGTCAAACT-------GTGAGGGGATCAGGAATGAGAATACT...-3'
DQ360837  5'-...GATGATTCCTGAAAGGAATGAACAAGGGCAGACG-------GTGAGAGGATCAGGAATGAGAATACT...-3'
JN588924  5'-...GATGATCCCTGAAAGGAATGAACAAGGACAGACG-------GTGAGAGGCTCAGGAATGAGAATACT...-3'
AY576380  5'-...GATGATTCCTGAAAGGAATGAACAAGGGCAGACG-------GTGAGAGGCTCAGGAATGAGAATACT...-3'
JN588923  5'-...GATGATCCCTGAAAGGAATGAACAAGGACAGACG-------GTGAGAGGCTCAGGAATGAGAATACT...-3'
EU146687  5'-...GATGATTCCTGAAAGGAATGAACAAGGGCAGACG-------GTGAGAGGCTCTGGAATGAGAATACT...-3'
JN588928  5'-...GATGATCCCTGAAAGGAATGAACAAGGACAGACG-------GTGAGAGGCTCAGGAATGAGAATACT...-3'
CY098741  5'-...TATGATTCCTGAAAGGAATGAACAAGGGCAGACG-------GTGAGAGGCTCAGGAATGAGAATACT...-3'
JN588927  5'-...GATGATCCCTGAAAGGAATGAACAAGGACAGACG-------GTGAGAGGCTCAGGAATGAGAATACT...-3'
CY116643  5'-...GATGATTCCTGAAAGGAATGAGCAAGGGCAGACG-------GTGAGAGGCTCTGGAATGAGAATGCT...-3'
AB212050  5'-...GATGATTCCTGAAAGGAATGAACAAGGGCAGACG-------GTGAGAGGCTCAGGAATGAGAATACT...-3'
```

```
                      222          235   240              255       1945         1956   1961     1970
                       *            *     *                *         *             *     *         *
AB212051   5'-...GATGATTCCTGAAAGGAATGAACAAGGGCAGACG-------GTGAGAGGCTCAGGAATGAGAATACT...-3'
AY626149   5'-...GATGATTCCTGAAAGGAATGAACAAGGGCAGACG-------GTGAGAGGCTCAGGAATGAGAATACT...-3'
AY627898   5'-...GATGATTCCTGAAAGGAATGAACAAGGGCAGACG-------GTGAGAGGCTCAGGAATGAGAATACT...-3'
EF587274   5'-...GATGGTTCCTGAAAGGAATGAACAAGGGCAGACG-------GTGAGAGGCTCAGGAATGAGAATACT...-3'
GQ466181   5'-...GATGATTCCTGAAAGGAATGAACAAGGGCAGACG-------GTGAGAGGCTCAGGAATGAGAATACT...-3'
AY627892   5'-...GATGATTCCTGAAAGGAATGAACAAGGGCAGACG-------GTGAGAGGCTCAGGAATGAGAATACT...-3'
HM006756   5'-...GATGATTCCTGAAAGGAATGAACAAGGGCAGACG-------GTGAGAGGCTCAGGAATGAGAATACT...-3'
HQ200462   5'-...GATGATTCCTGAAAGGAATGAACAAGGGCAGACA-------GTGAGAGGCTCAGGAATGAGAATACT...-3'
HQ200422   5'-...GATGATTCCTGAAAGGAATGAACAAGGGCAGACG-------GTGAGAGGCTCAGGAATGAGAATACT...-3'
HM172451   5'-...GATGATTCCTGAAAGGAATGAACAAGGGCAGACG-------GTGAGAGGCTCAGGAATGAGAATACT...-3'
CY098600   5'-...GATGATTCCTGAAAGGAATGAACAAGGGCAGACG-------GTGAGAGGCTCAGGAATGAGAATACT...-3'
EF541126   5'-...GATGATCCCTGAACGGAATGAACAGGGACAAACG-------GTGAGAGGTTCAGGAATGAGAATACT...-3'
AB462292   5'-...GATGATCCCTGAACGGAATGAACAGGGACAAACG-------GTGAGAGGTTCAGGAATGAGAATACT...-3'
DQ835310   5'-...GATGATCCCTGAACGGAATGAACAGGGACAAACG-------GTGAGAGGTTCAGGAATGAGAATACT...-3'
AF036363   5'-...GATGATCCCTGAAAGGAATGAGCAAGGTCAAACT-------GTGAGGGGATCAGGAATGAGAATACT...-3'
AF258840   5'-...GATGATCCCTGAAAGGAATGAGCAAGGTCAAACT-------GTGAGGGGATCAGGAATGAGAATACT...-3'
AF258846   5'-...GATGATCCCTGAAAGGAATGAGCAAGGTCAAACT-------GTGAGGGGATCAGGAATGAGAATACT...-3'
AJ404632   5'-...GATGATCCCTGAAAGGAATGAGCAAGGTCAAACT-------GTGAGGGGATCAGGAATGAGAATACT...-3'
AF258839   5'-...GATGATCCCTGAAAGGAATGAGCAAGGTCAAACT-------GTGAGGGGATCAGGAATGAGAATACT...-3'
AF258838   5'-...GATGATCCCTGAAAGGAATGAGCAAGGTCAAACT-------GTGAGGGGATCAGGAATGACAATACT...-3'
AF258845   5'-...GATGATCCCTGAAAGGAATGAGCAAGGTCAAACT-------GTGAGGGGATCAGGAATGAGAATACT...-3'
AF258844   5'-...GATGATCCCTGAAAGGAATGAGCAAGGGCAAACT-------GTGAGGGGATCAGGAATGAGAATACT...-3'
AF258837   5'-...GATGATCCCTGAAAGGAATGATCAAGGTCAAACT-------GTGAGGGGATCAGGAATGAGAATACT...-3'
AF258843   5'-...GATGATCCCTGAAAGGAATGAGCAAAGTCAAACT-------GTGAGGGGATCAGGAATGAGAATACT...-3'
CY017675   5'-...GATGATTCCTGAAAGGAATGAACAAGGGCAGACG-------GTGAGAGGCTCTGGAATGAGAATACT...-3'
CY014167   5'-...GATGATTCCTGAAAGGAATGAGCAAGGGCAGACG-------GTGAGAGGCTCTGGAATGAGAATACT...-3'
CY017659   5'-...GATGATTCCTGAAAGGAATGAACAAGGGCAAACG-------GTGAGAGGCTCTGGAATGAGAATACT...-3'
CY017651   5'-...GATGATTCCTGAAAGGAATGAACAAGGGCAGACG-------GTGAGAGGCTCTGGAATGAGAATACT...-3'
CY017667   5'-...GATGATTCCTGAAAGGAATGAACAAGGGCAGACG-------GTGAGAGGCTCTGGAATGAGAATACT...-3'
CY017685   5'-...GATGATTCCTGAAAGGAATGAACAAGGGCAAACG-------GTGAGAGGCTCTGGAATGAGAATACT...-3'
CY017635   5'-...GATGATTCCTGAAAGGAATGAACAAGGGCAAACG-------GTGAGAGGCTCTGGAATGAGAATACT...-3'
CY017643   5'-...GATGATTCCTGAAAGGAATGAACAAGGGCAAACG-------GTGAGAGGCTCTGGAATGAGAATACT...-3'
CY014367   5'-...GATGATTCCTGAAAGGAATGAACAAGGGCAAACG-------GTGAGAGGCTCTGGAATGAGAATACT...-3'
CY014325   5'-...GATGATTCCTGAAAGGAATGAACAAGGGCAGACG-------GTGAGAGGCTCTGGAATGAGAATACT...-3'
CY014348   5'-...GATGATTCCTGAAAGGAATGAACAAGGGCAGACG-------GTGAGAGGCTCTGGAATGAGAATACT...-3'
DQ535731   5'-...GATGATTCCTGAAAGGAATGAACAAGGGCAGACG-------GTGAGAGGCTCAGGAATGAGAATACT...-3'
CY014322   5'-...GATGATTCCTGAAAGGAATGAACAAGGGCAGACG-------GTAAGAGGCTCTGGAATGAGAATACT...-3'
CY014352   5'-...GATGATTCCTGAAAGGAATGAACAAGGGCAGACG-------GTGAGAGGCTCTGGAATGAGAATACT...-3'
CY014346   5'-...GATGATTCCTGAAAGGAATGAACAAGGGCAGACG-------GTGAGAGGCTCTGGAATGAGAATACT...-3'
```

```
                      222          235  240            255      1945       1956  1961    1970
                       *            *    *              *        *          *     *        *
CY014337  5'-...GATGATTCCTGAAAGGAATGAACAAGGGCAGACG-------GTGAGAGGCTCTGGAATGAGAATACT...-3'
CY014341  5'-...GATGATTCCTGAAAGGAATGAACAAGGGCAGACG-------GTGAGAGGCTCTGGAATGAGAATACT...-3'
CY014329  5'-...GATGATTCCTGAAAGGAATGAACAAGGGCAGACG-------GTGAGAGGCTCTGGAATGAGAATACT...-3'
EU146679  5'-...GATGATTCCTGAAAGGAATGAACAAGGGCAGACG-------GTGAGAGGCTCTGGAATGAGAATACT...-3'
CY014332  5'-...GATGATTCCTGAAAGGAATGAACAAGGGCAGACG-------GTGAGAGGCTCTGGAATGAGAATACT...-3'
EU146647  5'-...GATGATTCCTGAAAGGAATGAACAAGGGCAGACG-------GTAAGAGGCTCTGGAATGAGAATACT...-3'
CY014344  5'-...GATGATTCCTGAAAGGAATGAACAAGGGCAGACG-------GTGAGAGGCTCTGGAATGAGAATACT...-3'
DQ492894  5'-...GATGATTCCTGAAAGGAATGAACAAGGGCAGACG-------GTGAGAGGCTCAGGAATGAGAATACT...-3'
CY014333  5'-...GATGATTCCTGAAAGGAATGAACAAGGGCAGACG-------GTGAGAGGCTCTGGAATGAGAATACT...-3'
CY014359  5'-...GATGATTCCTGAAAGGAATGAACAAGGGCAGACG-------GTGAGAGGCTCTGGAATGAGAATACT...-3'
CY014356  5'-...GATGATTCCTGAAAGGAATGAACAAGGGCAGACG-------GTGAGAGGCTCTGGAATGAGAATACT...-3'
CY014364  5'-...GATGATTCCTGAAAGGAATGAACAAGGGCAGACG-------GTGAGAGGCTCTGGAATGAGAATACT...-3'
DQ492896  5'-...GATGATTCCTGAAAGGAATGAACAAGGGCAGACG-------GTGAGAGGCTCAGGAATGAGAATACT...-3'
CY014534  5'-...GATGATCCCTGAAAGGAATGAACAAGGGCAGACG-------GTGAGAGGCTCTGGAATGAGAATACT...-3'
CY014375  5'-...GATGATTCCTGAAAGGAATGAACAAGGGCAGACG-------GTGAGAGGCTCTGGAATGAGAATACT...-3'
CY014308  5'-...GATGATTCCTGAAAGGAATGAACAAGGGCAGACG-------GTGAGAGGCTCTGGAATGAGAATACT...-3'
CY014383  5'-...GATGATTCCTGAAAGGAATGAACAAGGGCAGACG-------GTGAGAGGCTCTGGAATGAGAATACT...-3'
EU146784  5'-...GATGATTCCTGAAAGGAATGAACAAGGGCAGACG-------GTGAGAGGCTCTGGAATGAGAATACT...-3'
CY014396  5'-...GATGATTCCTGAAAGGAATGAACAAGGGCAGACG-------GTGAGAGGCTCTGGAATGAGAATACT...-3'
EU268216  5'-...GATGATTCCTGAAAGGAATGAACAAGGGCAGACG-------GTGAGAGGCTCAGGAATGAGAATACT...-3'
CY014360  5'-...GATGATTCCTGAAAGGAATGAACAAGGGCAGACG-------GTGAGAGGCTCTGGAATGAGAATACT...-3'
CY014492  5'-...GATGATTCCTGAAAGGAATGAACAAGGGCAGACG-------GTGAGAGGCTCTGGAATGAGAATACT...-3'
CY014484  5'-...GATGATTCCTGAAAGGAATGAACAAGGGCAGACG-------GTGAGAGGCTCTGGAATGAGAATACT...-3'
CY014500  5'-...GATGATTCCTGAAAGGAATGAACAAGGGCAGATG-------GTGAGAGGCTCTGGAATGAGAATACT...-3'
CY014391  5'-...GATGATTCCTGAAAGGAATGAACAAGGGCAGACG-------GTGAGAGGCTCTGGAATGAGAATACT...-3'
DQ138181  5'-...GATGATTCCTGAAAGGAATGAACAAGGGCAGACG-------GTGAGAGGCTCAGGAATGAGAATACT...-3'
CY017683  5'-...GATGATTCCTGAAAGGAATGAACAAGGGCAGACG-------GTGAGAGGCTCTGGAATGAGAATACT...-3'
DQ492900  5'-...GATGATTCCTGAAAGGAATGAACAAGGGCAGACG-------GTGAGAGGCTCAGGAATGAGAATACT...-3'
CY014412  5'-...GATGATTCCTGAAAGGAATGAACAAGGGCAGACG-------GTGAGAGGCTCTGGAATGAGAATACT...-3'
CY014404  5'-...GATGATTCCTGAAAGGAATGAACAAGGGCAGACG-------GTGAGAGGCTCTGGAATGAGAATACT...-3'
AY651718  5'-...GATGATTCCTGAAAGGAATGAACAAGGGCAGACG-------GTGAGAGGCTCAGGAATGAGAATACT...-3'
DQ492902  5'-...GATGATTCCTGAAAGGAATGAACAAGGGCAGACG-------GTGAGAGGCTCAGGAATGAGAATACT...-3'
CY014452  5'-...GATGATTCCTGAAAGGAATGAACAAGGGCAGACG-------GTGAGAGGCTCTGGAATGAGAATACT...-3'
CY014460  5'-...GATGATTCCTGAAAGGAATGAACAAGGGCAGACG-------GTGAGAGGCTCTGGAATGAGAATACT...-3'
CY014420  5'-...GATGATTCCTGAAAGGAATGAACAAGGGCAGACG-------GTGAGAGGCTCTGGAATGAGAATACT...-3'
CY014428  5'-...GATGATTCCTGAAAGGAATGAACAAGGGCAGACG-------GTGAGAGGCTCTGGAATGAGAATACT...-3'
AY651719  5'-...GATGATTCCTGAAAGGAATGAACAAGGGCAGACG-------GTGAGAGGCTCAGGAATGAGAATACT...-3'
CY014444  5'-...GATGATTCCTGAAAGGAATGAACAAGGGCAGACG-------GTGAGAGGCTCTGGAATGAGAATACT...-3'
CY014526  5'-...GATGATTCCTGAAAGGAATGAACAAGGGCAGACG-------GTGAGAGGCTCTGGAATGAGAATACT...-3'
```

```
               222          235  240            255     1945      1956  1961     1970
                *            *    *              *       *          *    *        *
CY014512  5′-...GATGATTCCTGAAAGGAATGAACAAGGGCAGACG-------GTGAGAGGCTCTGGAATGAGAATACT...-3′
AY818126  5′-...GATGATTCCTGAAAGGAATGAACAAGGGCAGACG-------GTGAGAGGCTCAGGAATGAGAATACT...-3′
EU146655  5′-...AATGATTCCTGAAAGGAATGAACAAGGGCAGACA-------GTGAGAGGCTCTGGAATGAGAATACT...-3′
CY014520  5′-...GATGATTCCTGAAAGGAATGAACAAGGGCAGACG-------GTGAGAGGCTCTGGAATGAGAATACT...-3′
CY014320  5′-...AATGATTCCTGAAAGGAATGAACAAGGGCAGACA-------GTGAGAGGCTCTGGAATGAGAATACT...-3′
CY014175  5′-...AATGATTCCTGAAAGGAATGAACAAGGGCAGACA-------GTGAGAGGCTCTGGAATGAGAATACT...-3′
DQ372598  5′-...GATGATTCCTGAAAGGAATGAACAAGGGCAGACG-------GTGAGAGGATCAGGAATGAGAATACT...-3′
JX235396  5′-...AATGATTCCTGAAAGGAATGAACAAGGGCAGACA-------GTGAGAGGCTCTGGAATGAGAATACT...-3′
CY014468  5′-...GATGATTCCCGAAAGGAATGAACAAGGACAGACG-------GTGAGAGGCTCTGGAATGAGAATACT...-3′
EU146776  5′-...GATGATTCCCGAAAGGAATGAACAAGGACAGACG-------GTGAGAGGCTCTGGAATGAGAATACT...-3′
CY014269  5′-...GATGATTCCCGAAAGGAATGAACAAGGACAGACG-------GTGAGAGGCTCTGGAATGAGAATACT...-3′
CY014277  5′-...GATGATTCCCGAAAGGAATGAACAAGGACAGACG-------GTGAGAGGCTCTGGAATGAGAATACT...-3′
CY014285  5′-...GATGATTCCCGAAAGGAATGAACAAGGACAGACG-------GTGAGAGGCTCTGGAATGAGAATACT...-3′
CY014392  5′-...GATGATTCCCGAAAGGAATGAACAAGGACAGACG-------GTGAGAGGCTCTGGAATGAGAATACT...-3′
CY014436  5′-...GATGATTCCCGAAAGGAATGAACAAGGACAGACG-------GTGAGAGGCTCTGGAATGAGAATACT...-3′
EU146800  5′-...GATGATTCCCGAAAGGAATGAACAAGGACAGACG-------GTGAGAGGCTCTGGAATGAGAATACT...-3′
CY014476  5′-...GATGATTCCCGAAAGGAATGAACAAGGACAGACG-------GTGAGAGGCTCTGGAATGAGAATACT...-3′
EU146774  5′-...GATGATTCCCGAAAGGAATGAACAAGGACAGACG-------GTGAGAGGCTCTGGAATGAGAATACT...-3′
HM172438  5′-...GATGATTCCTGAAAGGAATGAACAAGGGCAGACG-------GTGAGAGGCTCAGGAATGAGAATACT...-3′
CY014293  5′-...GATGATTCCCGAAAGGAATGAACAAGGACAGACG-------GTGAGAGGCTCTGGAATGAGAATACT...-3′
```
