# Supplementary material for: Evidence for a Novel Mechanism of Influenza Virus-Induced Type I Interferon Expression by a Defective RNA-Encoded Protein
Source: PLoS Pathog. 2015 May 29;11(5):e1004924. doi: 10.1371/journal.ppat.1004924 (PMC4449196; doi:10.1371/journal.ppat.1004924)
Supplement: S2 Table — All sequences were obtained from NCBI’s Influenza Virus Resource (http://www.ncbi.nlm.nih.gov/genomes/FLU/FLU.html). (PDF) [file ppat.1004924.s002.pdf]

**S2 Table. Multiple sequence alignment of PB2 mRNA fragments from known avian H5N1 influenza A viruses.** All sequences were obtained from NCBI's Influenza Virus Resource (http://www.ncbi.nlm.nih.gov/genomes/FLU/FLU.html).

```
               224        235  240           254 1941           1956 1961    1969
                *          *    *             *   *              *    *       *
CY111595  5′-...TGATTCCTGAAAGGAATGAACAAGGGCAGAC---GAATGTGAGAGGCTCAGGAATGAGAATAC...-3′
DQ840518  5′-...TGATTCCTGAAAGGAATGAACAGGGACAGAC---GAATGTGAGAGGCTCAGGAATGAGAATAC...-3′
EU443540  5′-...TGATTCCTGAAAGGAATGAACAAGGACAGAC---GAATGTGAGAGGCTCAGGAATGAGAATAC...-3′
DQ320823  5′-...TGATCCCTGAACGGAATGAACAGGGACAAAC---GAATGTGAGAGGTTCAGGAATGAGAATAC...-3′
DQ992710  5′-...TAATTCCTGAAAGGAATGAACAAGGGCAGAC---GAATGTGAGAGGCTCAGGAATGAGAATAC...-3′
CY036122  5′-...TGATCCCTGAAAGGAATGAACAAGGGCAGAC---GAATGTGAGAGGCTCAGGAATGAGGATAC...-3′
FJ010991  5′-...TGATTCCTGAGAGAAATGAACAAGGGCAGAC---GAATGTGAGAGGCTCAGGAATGAGAATAC...-3′
DQ997331  5′-...TGGTTCCTGAAAGGAATGAACAAGGGCAGAC---GAATGTGAGAGGCTCAGGGATGAGAATAC...-3′
EU008590  5′-...TGATTCCTGAAAGAAATGAACAAGGGCAGAC---GAATGTGAGAGGCTCAGGAATGAGAATAC...-3′
FJ010983  5′-...TGATTCCTGAGAGAAATGAACAAGGGCAGAC---GAATGTGAGAGGCTCAGGAATGAGAATAC...-3′
FJ010997  5′-...TGATTCCTGAAAGGAATGAACAAGGACAGAC---GAATGTGAGAGGCTCAGGAATGAGAATAC...-3′
DQ320815  5′-...TGATCCCTGAACGGAATGAACAGGGACAAAC---GAATGTGAGAGGTTCAGGAATGAGAATAC...-3′
CY036114  5′-...TGATCCCTGAAAGGAATGAACAAGGGCAGAC---GAATGTGAGAGGCTCAGGAATGAGGATAC...-3′
AY770993  5′-...TGATTCCTGAAAGGAATGAACAAGGGCAGAC---GAATGTGAGAGGCTCAGGAATGAGAATAC...-3′
CY029011  5′-...TGATTCCTGAAAGGAATGAACAAGGGCAGAC---GAATGTGAGAGGCTCAGGAATGAGAATAC...-3′
EU889040  5′-...TGATTCCTGAAAGGAATGAACAGGGACAGAC---GAATGTGAGAGGCTCAGGAATGAGAATAC...-3′
EU889046  5′-...TGATTCCTGAAAGGAATGAACAAGGACAGAC---GAATGTTAGAGGCTCAGGAATGAGAATAC...-3′
EU889037  5′-...TGATTCCTGAAAGGAATGAACAGGGACAGAC---GAATGTGAGAGGCTCAGGAATGAGAATAC...-3′
EU889038  5′-...TGATTCCTGAAAGGAATGAACAGGGACAGAC---GAATGTGAGAGGCTCAGGAATGAGAATAC...-3′
EU889036  5′-...TGATTCCTGAAAGGAATGAACAGGGACAGAC---GAATGTGAGAGGCTCAGGAATGAGAATAC...-3′
EU889045  5′-...TGATTCCTGAAAGGAATGAACAAGGACAGAC---GAATGTTAGAGGCTCAGGAATGAGAATAC...-3′
EU889044  5′-...TGATTCCTGAAAGGAATGAACAGGGACAGAC---GAATGTGAGAGGCTCAGGAATGAGAATAC...-3′
EU889035  5′-...TGATTCCTGAAAGGAATGAACAGGGACAGAC---GAATGTGAGAGGCTCAGGAATGAGAATAC...-3′
EU889039  5′-...TGATTCCTGAAAGGAATGAACAGGGACAGAC---GAATGTGAGAGGCTCAGGAATGAGAATAC...-3′
EU889041  5′-...TGATTCCTGAAAGGAATGAACAGGGACAGAC---GAATGTGAGAGGCTCAGGAATGAGAATAC...-3′
EU889042  5′-...TGATTCCTGAAAGGAATGAACAGGGACAGAC---GAATGTGAGAGGCTCAGGAATGAGAATAC...-3′
DQ992673  5′-...TGGTTCCTGAAAGGAATGAACAAGGGCAGAC---GAATGTGAGAGGCTCAGGAATGAGAATAC...-3′
DQ822557  5′-...TGATTCCTGAAAGGAATGAACAAGGGCAGAC---GAATGTGAGAGGCTCAGGAATGAGAATAC...-3′
DQ822558  5′-...TGATTCCTGAAAGGAATGAACAGGGACAGAC---GAATGTGAGAGGCTCAGGAATGAGAATAC...-3′
DQ822559  5′-...TGATTCCTGAAAGGAATGAACAGGGACAGAC---GAATGTGAGAGGCTCAGGAATGAGAATAC...-3′
JQ936692  5′-...TGATTCCTGAAAGGAATGAACAAGGGCAGAC---GAACGTGAGGGGCTCAGGAATGAGAATAC...-3′
FJ864697  5′-...TGATCCCTGAAAGAAATGAGCAGGGTCAGAC---GAACGTGAGGGGTTCAGGAATGAGAATAC...-3′
FJ864698  5′-...TGATCCCTGAAAGAAATGAGCAGGGTCAGAC---GAACGTGAGGGGTTCAGGAATGAGAATAC...-3′
CY029340  5′-...TGATTCCTGAAAGGAATGAACAAGGGCAGAC---GAATGTGAGAGGCTCAGGAATGAGAATAC...-3′
KF597834  5′-...TGATTCCTGAAAGGAATGAACAAGGACAGAC---GAATGTGAGAGGCTCAGGAATGAGAATAC...-3′
DQ100543  5′-...TGATTCCTGAAAGGAATGAACAAGGACAGAC---GAATGTGAGAGGCTCAGGAATGAGAATAC...-3′
DQ100545  5′-...TGATTCCTGAAAGGAATGAACAAGGACAGAC---GAATGTGAGAGGCTCAGGAATGAGAATAC...-3′
DQ100544  5′-...TGATTCCTGAAAGGAATGAACAAGGACAGAC---GAATGTGAGAGGCTCAGGAATGAGAATAC...-3′
DQ100542  5′-...TGATTCCTGAAAGGAATGAACAAGGACAGAC---GAATGTGAGAGGCTCAGGAATGAGAATAC...-3′
EU008598  5′-...TGGTTCCTGAAAGGAATGAACAAGGGCAGAC---GAATGTGAGAGGCTCAGGAATGAGAATAC...-3′
CY029081  5′-...TGATTCCTGAAAGGAATGAACAAGGGCAGAC---GAATGTGAGAGGCTCAGGAATGAGAATAC...-3′
CY036130  5′-...TGGTTCCTGAAAGGAATGAACAAGGGCAGAC---GAATGTGAGAGGCTCAGGAATGAGAATAC...-3′
CY036138  5′-...TGGTTCCTGAAAGGAATGAACAAGGGCAGAC---GAATGTGAGAGGCTCAGGAATGAGAATAC...-3′
CY029137  5′-...TGATTCCTGAAAGGAATGAACAAGGGCAGAC---GAATGTGAGAGGCTCAGGAATGAGAATAC...-3′
DQ320819  5′-...TGATCCCTGAACGGAATGAACAGGGACAAAC---GAATGTGAGAGGTTCAGGAATGAGAATAC...-3′
CY029074  5′-...TGATTCCTGAAAGGAATGAACAAGGGCAAAC---GAATGTGAGAGGCTCAGGAATGAGAATAC...-3′
DQ992637  5′-...TGATTCCTGAAAGGAATGAACAAGGGCAGAC---GAATGTGAGAGGCTCAGGAATGAGAATAC...-3′
CY036106  5′-...TGATCCCTGAAAGGAATGAACAAGGGCAGAC---GAATGTGAGAGGCTCAGGAATGAGGATAC...-3′
CY029067  5′-...TGATTCCCGAAAGGAATGAACAAGGGCAAAC---GAATGTGAGAGGCTCAGGAATGAGAATAC...-3′
CY029221  5′-...TGATTCCTGAAAGGAATGAACAAGGGCAGAC---GAATGTGAGAGGCTCAGGAATGAGAATAC...-3′
CY029396  5′-...TGATTCCTGAAAGGAATGAACAAGGGCAGAC---GAATGTGAGAGGCTCAGGAATGAGAATAC...-3′
DQ520852  5′-...TGATTCCTGAAAGGAATGAACAAGGGCAGAC---GAATGTGAGAGGCTCAGGAATGAGAATAC...-3′
JN588935  5′-...TGATCCCTGAAAGGAATGAACAAGGACAGAC---GAATGTGAGAGGCTCAGGAATGAGAATAC...-3′
CY028997  5′-...TGATTCCTGAAAGGAATGAACAAGGACAAAC---GAATGTGAGAGGCTCAGGAATGAGAATAC...-3′
CY029165  5′-...TGATCCCTGAACGGAATGAACAGGGACAGAC---GAATGTGAGAGGTTCAGGAATGAGAATAC...-3′
CY029046  5′-...TGATTCCCGAAAGGAATGAACAAGGGCAAAC---GAATGTGAGAGGCTCAGGAATGAGAATAC...-3′
DQ992697  5′-...TGATCCCTGAAAGAAATGAGCAAGGTCAAAC---GAACGTAAGAGGCTCAGGAATGAGAATAC...-3′
HQ200551  5′-...TGATTCCTGAAAGGAATGAACAAGGGCAGAC---GAATGTGAGAGGCTCAGGAATGAGAATAC...-3′
HQ200530  5′-...TGATTCCTGAAAGGAATGAACAAGGGCAGAC---GAATGTGAGAGGCTCAGGAATGAGAATAC...-3′
JQ936716  5′-...TGATTCCTGAAAGGAATGAACAAGGGCAGAC---GAACGTGAGGGGCTCAGGAATGAGAATAC...-3′
JQ936724  5′-...TGATTCCTGAAAGGAATGAACAAGGACAGAC---GAATGTGAGAGGCTCAGGAATGAGAATAC...-3′
EU620659  5′-...TGATTCCTGAAAGGAATGAACAAGGGCAGAC---GAATGTGAGAGGATCAGGAATGAGAATAC...-3′
EU620667  5′-...TGATTCCTGAAAGGAATGAACAAGGGCAGAC---GAATGTGAGAGGATCAGGAATGAGAATAC...-3′
```

| | |
|---|---|
| CY036162 | 5’-...TGATTCCTGAAAGGAATGAACAAGGGCAGAC---GAATGTGAGAGGCTCAGGAATGAGAATAC...-3’ |
| JF510040 | 5’-...TGATTCCTGAAAGGAATGAACAAGGGCAGAC---GAATGTGAGAGGCTCAGGAATGAGAATAC...-3’ |
| CY029172 | 5’-...TGATTCCTGAAAGGAATGAACAAGGGCAGAC---GAATGTGAGAGGCTCAGGAATGAGAATAC...-3’ |
| CY029116 | 5’-...TGATTCCTGAAAGGAATGAACAAGGGCAGAC---GAATGTGAGAGGCTCAGGAATGAGAATAC...-3’ |
| DQ992696 | 5’-...TGATTCCTGAAAGGAATGAACAAGGGCAGAC---GAATGTGAGAGGCTCAGGAATGAGAATAC...-3’ |
| HQ156772 | 5’-...TGATTCCTGAAAGGAATGAACAAGGACAGAC---GAATGTGAGAGGCTCAGGAATGAGAATAC...-3’ |
| DQ992683 | 5’-...TGATCCCTGAAAGAAATGAGCAAGGTCAAAC---GAACGTAAGGGGCTCAGGAATGAGAATAC...-3’ |
| CY048544 | 5’-...TGATTCCTGAAAGGAATGAACAGGGACAAAC---GAATGTGAGAGGCTCAGGAATGAGAATAC...-3’ |
| AY576382 | 5’-...TGATTCCTGAAAGGAATGAACAAGGGCAGAC---GAATGTGAGAGGCTCAGGAATGAGAATAC...-3’ |
| CY048048 | 5’-...TGATTCCTGAAAGGAATGAACAGGGACAGAC---GAATGTGAGAGGCTCAGGAATGAGAATAC...-3’ |
| CY048168 | 5’-...TGATTCCTGAAAGGAATGAACAGGGACAGAC---GAATGTGAGAGGCTCAGGAATGAGAATAC...-3’ |
| FM165519 | 5’-...TGATTCCTGAAAGGAATGAACAAGGACAGAC---GAATGTGAGAGGCTCAGGAATGAGAATAC...-3’ |
| CY048440 | 5’-...TGATTCCTGAAAGGAATGAACAGGGACAGAC---GAATGTGAGAGGCTCCGGAATGAGAATAC...-3’ |
| CY048456 | 5’-...TGATTCCTGAAAGGAATGAACAGGGACAGAC---GAATGTGAGAGGCTCAGGAATGAGAATAC...-3’ |
| CY048336 | 5’-...TGATTCCTGAAAGGAATGAACAGGGACAGAC---GAATGTGAGAGGCTCAGGAATGAGAATAC...-3’ |
| CY048608 | 5’-...TGATTCCTGAAAGGAATGAACAGGGACAAAC---GAATGTGAGAGGCTCAGGAATGAGAATAC...-3’ |
| CY029144 | 5’-...TGATTCCTGAAAGGAATGAACAAGGGCAGAC---GAATGTGAGAGGCTCAGGAATGAGAATAC...-3’ |
| EU233413 | 5’-...TGATTCCTGAAAGGAATGAACAAGGGCAGAC---GAATGTGAGAGGCTCAGGAATGAGAATAC...-3’ |
| CY029270 | 5’-...TGATTCCTGAAAGGAATGAACAAGGGCAGAC---GAATGTGAGAGGCTCTGGAATGAGAATAC...-3’ |
| CY048040 | 5’-...TGATTCCTGAAAGGAATGAACAGGGACAGAC---GAATGTGAGAGGCTCAGGAATGAGAATAC...-3’ |
| DQ992644 | 5’-...TGATTCCTGAAAGGAATGAACAAGGGCAGAC---GAATGTGAGAGGCTCAGGAATGAGAATAC...-3’ |
| DQ992596 | 5’-...TGATTCCTGAAAGGAATGAACAAGGGCAGAC---GAATGTGAGAGGCTCAGGAATGAGAATAC...-3’ |
| DQ992604 | 5’-...TGATTCCTGAAAGGAATGAACAAGGGCAGAC---GAATGTGAGAGGCTCAGGAATGAGAATAC...-3’ |
| DQ992607 | 5’-...TGATTCCTGAAAGGAATGAACAAGGGCAGAC---GAATGTGAGAGGCTCAGGAATGAGAATAC...-3’ |
| DQ992608 | 5’-...TGATTCCTGAAAGGAATGAACAAGGGCAGAC---GAATGTGAGAGGCTCAGGAATGAGAATAC...-3’ |
| CY048216 | 5’-...TGATTCCTGAAAGGAATGAACAGGGACAGAC---GAATGTGAGAGGCTCAGGAATGAGAATAC...-3’ |
| CY048376 | 5’-...TGATTCCTGAAAGGAATGAACAGGGACAGAC---GAATGTGAGAGGCTCAGGAATGAGAATAC...-3’ |
| EU590689 | 5’-...TGATTCCTGAAAGGAATGAACAAGGACAGAC---GAATGTGAGAGGCTCAGGAATGAGAATAC...-3’ |
| CY048384 | 5’-...TGATCCCTGAAAGGAATGAACAGGGACAGAC---GAATGTGAGAGGCTCAGGAATGAGAATAC...-3’ |
| DQ992645 | 5’-...TGGTTCCTGAAAGGAATGAACAAGGGCAGAC---GAATGTGAGAGGCTCAGGAATGAGAATAC...-3’ |
| DQ992590 | 5’-...TGGTTCCTGAAAGGAATGAACAAGGGCAGAC---GAATGTGAGAGGCTCAGGAATGAGAATAC...-3’ |
| DQ992605 | 5’-...TGATTCCTGAAAGGAATGAACAAGGGCAGAC---GAATGTGAGAGGCTCAGGAATGAGAATAC...-3’ |
| DQ992606 | 5’-...TGATTCCTGAAAGGAATGAACAAGGGCAGAC---GAATGTGAGAGGCTCAGGAATGAGAATAC...-3’ |
| DQ992646 | 5’-...TGATTCCTGAAAGGAATGAACAAGGGCAGAC---GAATGTGAGAGGCTCAGGAATGAGAATAC...-3’ |
| CY048432 | 5’-...TGATTCCTGAAAGGAATGAACAGGGACAGAC---GAATGTGAGAGGCTCCGGAATGAGAATAC...-3’ |
| CY048392 | 5’-...TGATTCCTGAAAGGAATGAACAGGGACAGAC---GAATGTGAGAGGCTCAGGAATGAGAATAC...-3’ |
| CY029764 | 5’-...TGATCCCTGAAAGGAATGAACAAGGGCAGAC---GAATGTGAGAGGCTCAGGAATGAGGATAC...-3’ |
| EU616858 | 5’-...TGATTCCTGAAAGGAATGAACAAGGGCAGAC---GAATGTGAGAGGCTCAGGAATGAGAATAC...-3’ |
| CY048640 | 5’-...TGATTCCTGAAAGGAATGAACAGGGACAAAC---GAATGTGAGAGGCTCAGGAATGAGAATAC...-3’ |
| CY048632 | 5’-...TGATTCCTGAAAGGAATGAACAGGGACAGAC---GAATGTGAGAGGCTCCGGAATGAGAATAC...-3’ |
| CY048136 | 5’-...TGATTCCTGAAAGGAATGAACAGGGACAGAC---GAATGTGAGAGGCTCAGGAATGAGAATAC...-3’ |
| HQ156773 | 5’-...TGATTCCTGAAAGGAATGAACAAGGACAGAC---GAATGTGAGAGGCTCAGGAATGAGAATAC...-3’ |
| CY029130 | 5’-...TGATTCCTGAAAGGAATGAACAAGGGCAGAC---GAATGTGAGAGGCTCAGGAATGAGAATAC...-3’ |
| DQ992626 | 5’-...TGATTCCTGAAAGGAATGAACAAGGGCAGAC---GAATGTGAGAGGCTCAGGAATGAGAATAC...-3’ |
| DQ992625 | 5’-...TGATTCCTGAAAGGAATGAACAAGGGCAGAC---GAATGTGAGAGGCTCAGGAATGAGAATAC...-3’ |
| DQ992616 | 5’-...TGATTCCTGAAAGGAATGAACAAGGGCAGAC---GAATGTGAGAGGCTCAGGAATGAGAATAC...-3’ |
| HQ156757 | 5’-...TGATTCCTGAAAGGAATGAACAAGGACAGAC---GAATGTGAGAGGCTCAGGAATGARAATAC...-3’ |
| JQ936708 | 5’-...TGATTCCTGAAAGGAATGAACAAGGGCAGAC---AAATGTGAGAGGCTCAGGAATGAGAATAC...-3’ |
| CY029102 | 5’-...TGATTCCCGAAAGGAATGAACAAGGGCAAAC---GAATGTGAGAGGCTCAGGAATGAGAATAC...-3’ |
| CY029109 | 5’-...TGATTCCCGAAAGGAATGAACAAGGGCAAAC---GAATGTGAGAGGCTCAGGAATGAGAATAC...-3’ |
| CY029095 | 5’-...TGATTCCCGAAAGGAATGAACAAGGGCAAAC---GAATGTGAGAGGCTCAGGAATGAGAATAC...-3’ |
| DQ992649 | 5’-...TGATCCCTGAACGGAATGAACAGGGACAAAC---GAATGTGAGAGGTTCAGGAATGAGAATAC...-3’ |
| DQ992651 | 5’-...TGATCCCTGAACGGAATGAACAGGGACAAAC---GAATGTGAGAGGTTCAGGAATGAGAATAC...-3’ |
| DQ992653 | 5’-...TGATCCCTGAACGGAATGAACAGGGACAAAC---GAATGTGAGAGGTTCAGGAATGAGAATAC...-3’ |
| DQ992652 | 5’-...TGATCCCTGAACGGAATGAACAGGGACAAAC---GAATGTGAGAGGTTCAGGAATGAGAATAC...-3’ |
| DQ992655 | 5’-...TGATCCCTGAACGGAATGAACAGGGACAAAC---GAATGTGAGAGGTTCAGGAATGAGAATAC...-3’ |
| DQ992654 | 5’-...TGATCCCTGAACGGAATGAACAGGGACAAAC---GAATGTGAGAGGTTCAGGAATGAGAATAC...-3’ |
| DQ992658 | 5’-...TGATCCCTGAACGGAATGAACAGGGACAAAC---GAATGTGAGAGGTTCAGGAATGAGAATAC...-3’ |
| DQ992650 | 5’-...TGATCCCTGAACGGAATGAACAGGGACAAAC---GAATGTGAGAGGTTCAGGAATGAGAATAC...-3’ |
| DQ992584 | 5’-...TGATCCCTGAACGGAATGAACAGGGACAAAC---GAATGTGAGAGGTTCAGGAATGAGAATAC...-3’ |
| DQ992648 | 5’-...TGATCCCTGAACGGAATGAACAGGGACAAAC---GAATGTGAGAGGTTCAGGAATGAGAATAC...-3’ |
| DQ992656 | 5’-...TGATCCCTGAACGGAATGAACAGGGACAAAC---GAATGTGAGAGGTTCAGGAATGAGAATAC...-3’ |
| DQ992657 | 5’-...TGATCCCTGAACGGAATGAACAGGGACAAAC---GAATGTGAGAGGTTCAGGAATGAGAATAC...-3’ |
| DQ992587 | 5’-...TGATCCCTGAACGGAATGAACAGGGACAAAC---GAATGTGAGAGGTTCAGGAATGAGAATAC...-3’ |
| DQ992588 | 5’-...TGATCCCTGAACGGAATGAACAGGGACAAAC---GAATGTGAGAGGTTCAGGAATGAGAATAC...-3’ |
| DQ992659 | 5’-...TGATCCCTGAACGGAATGAACAGGGACAAAC---GAATGTGAGAGGTTCAGGAATGAGAATAC...-3’ |
| CY029772 | 5’-...TGATCCCTGAAAGAAATGAGCAAGGTCAGAC---GAACGTAAGGGGTTCAGGAATGAGAATAC...-3’ |
| EU443536 | 5’-...TGATTCCTGAAAGGAATGAACAAGGACAGAC---GAATGTTAGAGGCTCAGGAATGAGAATAC...-3’ |
| EU443535 | 5’-...TGATTCCTGAAAGGAATGAACAAGGACAGAC---GAATGTTAGAGGCTCAGGAATGAGAATAC...-3’ |
| EU443534 | 5’-...TGATTCCTGAAAGGAATGAACAAGGACAGAC---GAATGTTAGAGGCTCAGGAATGAGAATAC...-3’ |
| EU443533 | 5’-...TGATTCCTGAAAGGAATGAACAAGGACAGAC---GAATGTTAGAGGCTCAGGAATGAGAATAC...-3’ |
| EU443537 | 5’-...TGATTCCTGAAAGGAATGAACAGGGACAGAC---GAATGTGAGAGGCTCAGGAATGAGAATAC...-3’ |
| EU443538 | 5’-...TGATTCCTGAAAGGAATGAACAAGGACAGAC---GAATGTGAGAGGCTCAGGAATGAGAATAC...-3’ |

```
               224        235  240           254 1941           1956 1961   1969
                *          *    *             *   *              *    *      *
EU443539  5′-...TGATTCCTGAAAGGAATGAACAAGGACAGAC---GAATGTGAGAGGCTCAGGAATGAGAATAC...-3′
CY048400  5′-...TGATTCCTGAAAGGAATGAACAGGGACAGAC---GAATGTGAGAGGCTCAGGAATGAGAATAC...-3′
FM164849  5′-...TGATTCCTGAAAGGAATGAACAGGGACAGAC---GAATGTGAGAGGCTCCGGAATGAGAATAC...-3′
JN588931  5′-...TGATCCCTGAAAGGAATGAACAAGGACAGAC---GAATGTGAGAGGCTCAGGAATGAGAATAC...-3′
CY048288  5′-...TGATTCCTGAGAGGAATGAACAGGGACAGAC---GAATGTGAGAGGCTCAGGAATGAGAATAC...-3′
HQ200545  5′-...TGATTCCTGAAAGGAATGAACAAGGGCAGAC---GAATGTGAGAGGCTCAGGAATGAGAATAC...-3′
CY029347  5′-...TGATTCCTGAAAGGAATGAACAAGGGCAGAC---GAATGTGAGAGGCTCAGGAATGAGAATAC...-3′
CY029263  5′-...TGATTCCTGAAAGGAATGAACAAGGGCAGAC---GAATGTGAGAGGCTCAGGAATGAGAATAC...-3′
CY029207  5′-...TGATTCCTGAAAGGAATGAACAAGGGCAGAC---GAATGTGAGAGGCTCAGGAATGAGAATAC...-3′
CY029186  5′-...TGATTCCTGAAAGGAATGAACAAGGGCAGAC---GAATGTGAGAGGCTCAGGAATGAGAATAC...-3′
CY029305  5′-...TGATTCCTGAAAGGAATGAACAAGGGCAGAC---GAATGTGAGAGGCTCAGGAATGAGAATAC...-3′
CY029284  5′-...TGATTCCTGAAAGGAATGAACAAGGGCAGAC---GAATGTGAGAGGCTCAGGAATGAGAATAC...-3′
CY029333  5′-...TGATTCCTGAAAGGAATGAACAAGGGCAGAC---GAATGTGAGAGGCTCAGGAATGAGAATAC...-3′
CY029214  5′-...TGATTCCTGAAAGGAATGAACAAGGGCAGAC---GAATGTGAGAGGCTCAGGAATGAGAATAC...-3′
CY029200  5′-...TGATTCCTGAGAGGAATGAACAAGGGCAGAC---GAATGTGAGAGGCTCAGGAATGAGAATAC...-3′
CY029277  5′-...TGATTCCTGAAAGGAATGAACAAGGGCAGAC---GAATGTGAGAGGCTCAGGAATGAGAATAC...-3′
CY029291  5′-...TGATTCCTGAAAGGAATGAACAAGGGCAGAC---GAATGTGAGAGGCTCAGGAATGAGAATAC...-3′
DQ992695  5′-...TGATTCCTGAAAGGAATGAACAAGGGCAGAC---GAATGTGAGAGGCTCAGGAATGAGAATAC...-3′
CY048280  5′-...TGATTCCTGAAAGGAATGAACAGGGACAGAC---GAATGTGAGAGGCTCAGGAATGAGAATAC...-3′
CY029298  5′-...TGATTCCTGAAAGGAACGAACAAGGGCAGAC---GAATGTGAGAGGCTCTGGAATGAGAATAC...-3′
DQ992701  5′-...TGATTCCTGAAAGGAATGAACAAGGGCAGAC---GAATGTGAGAGGCTCAGGAATGAGAATAC...-3′
CY029032  5′-...TGATTCCTGAAAGGAATGAACAAGGGCAGAT---GAATGTGAGAGGCTCAGGAATGAGAATAC...-3′
DQ992713  5′-...TGATTCCTGAAAGGAATGAACAAGGGCAGAC---GAATGTGAGAGGCTCAGGAATGAGAATAC...-3′
DQ992674  5′-...TGATTCCTGAAAGGAATGAACAGGGGCAGAC---GAATGTGAGAGGCTCAGGAATGAGAATAC...-3′
CY048224  5′-...TGATTCCTGAAAGGAATGAACAGGGACAGAC---GAATGTGAGAGGCTCAGGAATGAGAATAC...-3′
CY029060  5′-...TGATTCCTGAAAGGAATGAACAAGGGCAAAC---GAATGTGAGAGGCTCAGGAATGAGAATAC...-3′
AF509154  5′-...TGATTCCTGAAAGGAATGAACAAGGGCAGAC---GAATGTGAGAGGCTCAGGAATGAGAATAC...-3′
CY029151  5′-...TGATTCCTGAAAGGAATGAACAAGGGCAGAC---GAATGTGAGAGGCTCAGGAATGAGAATAC...-3′
CY029158  5′-...TGATTCCTGAAAGGAATGAACAAGGGCAGAC---GAATGTGAGAGGCTCAGGAATGAGAATAC...-3′
CY029361  5′-...TGATTCCTGAAAGGAATGAACAAGGGCAGAC---GAATGTGAGAGGCTCAGGAATGAGAATAC...-3′
CY029382  5′-...TGATTCCTGAAAGGAATGAACAAGGGCAGAC---GAATGTGAGAGGCTCAGGAATGAGAATAC...-3′
CY029389  5′-...TGATTCCTGAAAGGAATGAACAAGGGCAGAC---GAATGTGAGAGGCTCAGGAATGAGAATAC...-3′
CY029375  5′-...TGATTCCTGAAAGGAATGAACAAGGGCAGAC---GAATGTGAGAGGCTCAGGAATGAGAATAC...-3′
CY029368  5′-...TGATTCCTGAAAGGAATGAACAAGGGCAGAC---GAATGTGAGAGGCTCAGGAATGAGAATAC...-3′
DQ992622  5′-...TGATTCCTGAAAGGAATGAACAAGGGCAGAC---GAATGTGAGAGGCTCAGGAATGAGAATAC...-3′
CY029740  5′-...TGATCCCTGAAAGGAATGAACAGGGGCAGAC---GAATGTGAGAGGATCAGGAATGAGGATAC...-3′
CY036154  5′-...TGATCCCTGAAAGAAATGAGCAGGGTCAGAC---GAACGTGAGGGGTTCAGGAATGAGAATAC...-3′
CY103944  5′-...TGATTCCTGAAAGGAATGAACAAGGACAGAC---GAATGTTAGAGGCTCAGGAATGAGAATAC...-3′
AY651730  5′-...TGATTCCCGAAAGGAATGAACAAGGGCAAAC---GAATGTGAGAGGCTCAGGAATGAGAATAC...-3′
EF619975  5′-...TGATTCCTGAAAGGAATGAACAAGGACAGAC---GAATGTTAGAGGCTCAGGAATGAGAATAC...-3′
EU146842  5′-...TGATTCCTGAAAGGAATGAACAAGGACAGAC---GAATGTTAGAGGCTCAGGAATGAGAATAC...-3′
DQ320811  5′-...TGATTCCTGAAAGGAATGAACAAGGGCAGAC---GAATGTGAGAGGCTCTGGAATGAGAATAC...-3′
HQ200434  5′-...TGATTCCTGAAAGGAATGAACAAGGGCAGAC---GAATGTGAGAGGTTCAGGAATGAGAATAC...-3′
HQ200440  5′-...TGATTCCTGAAAGGAATGAACAAGGGCAGAC---GAATGTGAGAGGTTCAGGAATGAGAATAC...-3′
HQ338099  5′-...TGATTCCTGAAAGGAATGAACAAGGGCAGAC---GAATGTGAGAGGCTCAGGAATGAGAATAC...-3′
FM164853  5′-...TGATTCCTGAAAGGAATGAACAGGGACAGAC---GAATGTGAGAGGCTCCGGAATGAGAATAC...-3′
DQ320826  5′-...TGATCCCTGAACGGAATGAACAGGGACAAAC---AAATGTGAGAGGTTCAGGAATGAGAATAC...-3′
AF509148  5′-...TGATTCCTGAAAGGAATGAACAAGGGCAGAC---GAATGTGAGAGGCTCAGGAATGAGAATAC...-3′
FM164852  5′-...TGATTCCTGAAAGGAATGAACAGGGACAGAC---GAATGTGAGAGGCTCCGGAATGAGAATAC...-3′
DQ992689  5′-...TGATTCCTGAAAGGAATGAACAAGGGCAGAC---GAATGTGAGAGGCTCAGGAATGAGAATAC...-3′
DQ992577  5′-...TGATTCCTGAAAGGAATGAACAAGGGCAGAC---GAATGTGAGAGGCTCAGGAATGAGAATAC...-3′
DQ992615  5′-...TGATTCCAGAAAGGAATGAACAAGGGCAGAC---GAATGTGAGAGGCTCAGGAATGAGAATAC...-3′
DQ992612  5′-...TGATTCCTGAAAGGAATGAACAAGGGCAGAC---GAATGTGAGAGGCTCAGGAATGAGAATAC...-3′
CY029612  5′-...TGATCCCTGAAAGGAATGAACAAGGGCAGAC---GAATGTGAGAGGCTCAGGAATGAGAATAC...-3′
CY055188  5′-...TGATTCCGGAGAGGAATGAACAAGGACAGAC---GAATGTTAGAGGCTCAGGAATGAGAATAC...-3′
DQ992663  5′-...TGATCCCTGAACGGAATGAACAGGGACAAAC---GAATGTGAGAGGTTCAGGAATGAGAATAC...-3′
DQ992586  5′-...TGATCCCTGAACGGAATGAACAGGGACAAAC---GAATGTGAGGGGTTCAGGAATGAGAATAC...-3′
AY651731  5′-...TGATCCCTGAAAGAAATGAGCAAGGTCAGAC---GAACGTGAGGGGTTCAGGAATGAGAATAC...-3′
CY048424  5′-...TGATTCCTGAAAGGAATGAACAGGGACAGAC---GAATGTGAGAGGCTCAGGAATGAGAATAC...-3′
CY036050  5′-...TGATCCCTGAAAGAAATGAGCAGGGTCAGAC---GAACGTGAGGGGTTCAGGAATGAGAATAC...-3′
EU233698  5′-...TGATTCCTGAAAGGAATGAACAAGGACAGAC---GAATGTGAGAGGCTCAGGAATGAGAATAC...-3′
CY048120  5′-...TGATTCCTGAAAAGAATGAACAGGGACAGAC---GAATGTGAGAGGCTCCGGAATGAGAATAC...-3′
EU875390  5′-...TGATTCCTGAAAGGAATGAAAAAGGGCAGAC---GAATGTGAGAGGATCAGGAATGAGAATAC...-3′
DQ992702  5′-...TGATTCCTGAAAGGAATGAACAAGGGCAGAC---GAATGTGAGAGGCTCAGGAATGAGAATAC...-3′
DQ992704  5′-...TGATTCCTGAAAGGAATGAACAAGGGCAGAC---GAATGTGAGAGGCTCAGGAATGAGAATAC...-3′
DQ992703  5′-...TAATTCCTGAAAGGAATGAACAAGGGCAGAC---GAATGTGAGAGGCTCAGGAATGAGAATAC...-3′
DQ992694  5′-...TGATTCCTGAAAGGAATGAACAAGGACAGAC---GAATGTGAGAGGCTCAGGAATGAGAATAC...-3′
DQ992687  5′-...TGATTCCTGAAAGGAATGAACAAGGGCAGAC---GAATGTGAGAGGCTCAGGAATGAGAATAC...-3′
CY029692  5′-...TGATCCCTGAAAGGAATGAACAAGGGCAGAC---GAATGTTAGAGGCTCAGGAATGAGGATAC...-3′
DQ992692  5′-...TGATTCCTGAAAGGAATGAACAAGGGCAGAC---GAATGTGAGAGGCTCAGGAATGAGAATAC...-3′
```

```
               224        235  240           254  1941          1956 1961    1969
                *          *    *             *   *              *    *       *
EU850421  5'-...TGATTCCTGAAAGGAATGAACAAGGGCAGAC---GAATGTGAGAGGATCAGGAATGAGAATAC...-3'
EF446769  5'-...TGATTCCTGAAAGGAATGAACAAGGACAGAC---GAATGTTAGAGGCTCAGGAATGAGAATAC...-3'
EF446777  5'-...TGATTCCTGAAAGGAATGAACAAGGACAGAC---GAATGTTAGAGGCTCAGGAATGAGAATAC...-3'
EF441267  5'-...TGATTCCTGAAAGGAATGAACAAGGACAGAC---GAATGTTAGAGGCTCAGGAATGAGAATAC...-3'
DQ992573  5'-...TGATTCCTGAAAGGAATGAACAAGGGCAGAC---GAATGTGAGAGGCTCAGGAATGAGAATAC...-3'
DQ992709  5'-...TGATTCCTGAAAGGAATGAACAAGGGCAGAC---GAATGTGAGGGGCTCAGGAATGAGAATAC...-3'
EU616890  5'-...TGATTCCTGAAAGGAATGAACAAGGGCAGAC---GAATGTGAGAGGCTCAGGAATGAGAATAC...-3'
CY029708  5'-...TGATCCCTGAAAGGAATGAACAAGGGCAGAC---GAATGTGAGAGGCTCAGGAATGAGGATAC...-3'
CY036058  5'-...TGATCCCTGAAAGAAATGAGCAGGGTCAGAC---GAACGTGAGGGGTTCAGGAATGAGAATAC...-3'
AF098582  5'-...TGATCCCTGAAAGGAATGAGCAAGGTCAAAC---GAATGTGAGGGGATCAGGAATGAGAATAC...-3'
AF098584  5'-...TGATCCCTGAAAGGAATGAGCAAGGTCAAAC---GAATGTGAGGGGATCAGGAATGAGAATAC...-3'
AF098581  5'-...TGATCCCTGAAAGGAATGAGCAAGGTCAAAC---GAATGTGAGGGGATCAGGAATGAGAATAC...-3'
AF098583  5'-...TGATCCCTGAAAGGAATGAGCAAGGTCGAAC---GAATGTGAGGGGATCAGGAATGAGAATAC...-3'
AF098577  5'-...TGATCCCTGAAAGGAATGAGCAAGGTCAAAC---GAATGTGAGGGGATCAGGAATGAGAATAC...-3'
AF098579  5'-...TGATCCCTGAAAGGAATGAGCAAGGTCAAAC---GAATGTGAGGGGATCAGGAATGAGAATAC...-3'
AF098580  5'-...TGATCCCTGAAAGGAATGAGCAAGGTCAAAC---GAATGTGAGGGGATCAGGAATGAGAATAC...-3'
CY048080  5'-...TGATTCCTGAAAGGAATGAACAGGGACAGAC---GAATGTGAGAGGCTCAGGAATGAGAATAC...-3'
EU616882  5'-...TGATTCCTGAAAGGAATGAACAAGGGCAGAC---GAATGTGAGAGGCTCAGGAATGAGAATAC...-3'
AY576388  5'-...TGATTCCTGAAAGGAATGAACAAGGGCAAAC---GAATGTGAGAGGCTCAGGAATGAGAATAC...-3'
DQ992711  5'-...TGATTCCTGAAAGGAATGAACAAGGGCAGAC---GAATGTGAGAGGCTCAGGAATGAGAATAC...-3'
EU616874  5'-...TGATTCCTGAAAGGAATGAACAAGGGCAGAC---GAATGTGAGAGGCTCAGGAATGAGAATAC...-3'
CY029256  5'-...TGATTCCTGAAAGGAATGAACAAGGGCAGAC---GAATGTGAGAGGCTCTGGAATGAGAATAC...-3'
AY576386  5'-...TGATTCCTGAAAGGAATGAACAAGGGCAGAC---GAATGTGAGAGGCTCAGGAATGAGAATAC...-3'
DQ992679  5'-...TGATTCCTGAAAGGAATGAACAGGGGCAGAC---GAATGTGAGAGGCTCAGGAATGAGAATAC...-3'
EU676312  5'-...TGATTCCTGAAAGGAATGAAAAAGGGCAGAC---GAATGTGAGAGGATCAGGAATGAGAATAC...-3'
DQ992693  5'-...TGATCCCTGAACGGAATGAACAGGGACAGAC---AAATGTGAGAGGTTCAGGAATGAGAATAC...-3'
DQ095753  5'-...TGATTCCTGAAAGGAATGAACAAGGACAGAC---GAATGTGAGAGGCTCAGGAATGAGAATAC...-3'
CY048352  5'-...TGATTCCTGAAAGGAATGAACAGGGACAAAC---GAATGTGAGAGGCTCAGGAATGAGAATAC...-3'
CY048600  5'-...TGATTCCTGAAAGGAATGAACAGGGACAAAC---GAATGTGAGAGGYTCAGGAATGAGAATAC...-3'
CY048560  5'-...TGATTCCTGAAAGGAATGAACAGGGACAGAC---GAATGTGAGAGGCTCAGGAATGAGAATAC...-3'
CY048096  5'-...TGATTCCTGAAAGGAATGAACAGGGACAGAC---GAATGTGAGAGGCTCCGGAATGAGAATAC...-3'
AY651733  5'-...TGATTCCTGAAAGGAATGAACAAGGGCAGAC---GAACGTAAGGGGTTCAGGAATGAGAATAC...-3'
DQ095752  5'-...TGATTCCTGAAAGGAATGAACAAGGACAGAC---GAATGTGAGAGGCTCAGGAATGAGAATAC...-3'
DQ095757  5'-...TGATTCCTGAAAGGAATGAACAAGGACAGAC---GAATGTGAGAGGCTCAGGAATGAGAATAC...-3'
DQ095760  5'-...TGATTCCTGAAAGGAATGAACAAGGACAGAC---GAATGTGAGAGGCTCAGGAATGAGAATAC...-3'
DQ095758  5'-...TGATTCCTGAAAGGAATGAACAAGGACAGAC---GAATGTGAGAGGCTCAGGAATGAGAATAC...-3'
EU213050  5'-...TGATTCCTGAAAGGAATGAACAAGGACAGAC---GAATGTGAGAGGCTCAGGAATGAGAATAC...-3'
DQ095754  5'-...TGATTCCTGAAAGGAATGAACAAGGACAGAC---GAATGTGAGAGGCTCAGGAATGAGAATAC...-3'
DQ095759  5'-...TGATTCCTGAAAGGAATGAACAAGGACAGAC---GAATGTGAGAGGCTCAGGAATGAGAATAC...-3'
EU616842  5'-...TGATTCCTGAAAGGAATGAACAAGGGCAGAC---GAATGTGAGAGGGTCAGGAATGAGAATAC...-3'
DQ320809  5'-...TGATTCCTGAAAGGAATGAACAAGGACAGAC---GAATGTGAGAGGCTCAGGAATGAGAATAC...-3'
CY029417  5'-...TGATTCCTGAAAGGAATGAACAAGGGCAGAC---GAATGTGAGAGGCTCAGGAATGAGAATAC...-3'
CY029403  5'-...TGATTCCTGAAAGGAATGAACAAGGGCAGAC---GAATGTGAGAGGCTCAGGAATGAGAATAC...-3'
CY029424  5'-...TGATTCCTGAAAGGAATGAACAAGGGCAGAC---GAATGTGAGAGGCTCAGGAATGAGAATAC...-3'
DQ320810  5'-...TGATTCCTGAAAGGAATGAACAAGGACAGAC---GAATGTGAGAGGCTCAGGAATGAGAATAC...-3'
CY029410  5'-...TGATTCCTGAAAGGAATGAACAAGGGCAGAC---GAATGTGAGAGGCTCAGGAATGAGAATAC...-3'
AY651734  5'-...TGATTCCTGAAAGGAATGAACAAGGGCAGAC---GAATGTGAGAGGCTCAGGAATGAGAATAC...-3'
AY585516  5'-...TGATTCCTGAAAGGAATGAACAAGGGCAGAC---GAATGTGAGAGGCTCAGGAATGAGAATAC...-3'
EU195413  5'-...TGGTTCCTGAAAGGAATGAACAAGGGCAGAC---GAATGTGAGAGGCTCAGGAATGAGAATAC...-3'
DQ320849  5'-...TGATTCCTGAAAGGAATGAACAAGGACAGAC---GAATGTGAGAGGCTCAGGAATGAGAATAC...-3'
DQ992681  5'-...TGATTCCTGAAAGGAATGAACAAGGACAGAC---GAATGTGAGAGGCTCAGGAATGAGAATAC...-3'
DQ095763  5'-...TGATTCCTGAAAGGAATGAACAAGGACAGAC---GAATGTGAGAGGCTCAGGAATGAGAATAC...-3'
CY029473  5'-...TGATTCCTGAAAGGAATGAACAAGGGCAGAC---GAATGTGAGAGGCTCAGGGATGAGAATAC...-3'
AY651714  5'-...TGATTCCTGAAAGGAATGAACAAGGGCAGAC---GAATGTGAGAGGCTCAGGAATGAGAATAC...-3'
DQ835803  5'-...TGATTCCTGAAAGGAATGAACAAGGGCAGAC---GAATGTGAGAGGCTCAGGAATGAGAATAC...-3'
AY676022  5'-...TGATTCCTGAAAGGAATGAACAAGGGCAGAC---GAATGTGAGAGGCTCAGGAATGAGAATAC...-3'
FJ868019  5'-...TGATTCCTGAAAGGAATGAACAAGGGCAGAC---GAATGTGAGAGGCTCAGGAATGAGAATAC...-3'
CY029438  5'-...TGATTCCTGAAAGGAATGAACAAGGGCAGAC---GAATGTGAGAGGCTCAGGGATGAGAATAC...-3'
CY029459  5'-...TGATTCCTGAAAGGAATGAACAAGGGCAGAC---GAATGTGAGAGGCTCAGGGATGAGAATAC...-3'
HM172484  5'-...TGATTCCTGAAAGGAATGAACAAGGACAGAC---GAATGTGAGAGGCTCAGGAATGAGAATAC...-3'
CY029354  5'-...TGATTCCTGAAAGGAATGAACAAGGGCAGAC---GAATGTGAGAGGCTCAGGAATGAGAATAC...-3'
AY651729  5'-...TGATTCCTGAAAGGAATGAACAAGGGCAGAC---GAATGTGAGAGGCTCAGGAATGAGAATAC...-3'
DQ095762  5'-...TGATTCCTGAAAGGAATGAACAAGGACAGAC---GAATGTGAGAGGCTCAGGAATGAGAATAC...-3'
AB189050  5'-...TGGTTCCTGAAAGGAATGAACAAGGGCAGAC---GAATGTGAGAGGCTCAGGAATGAGAATAC...-3'
DQ138171  5'-...TGATTCCTGAAAGGAATGAACAAGGGCAGAC---GAATGTGAGAGGCTCAGGAATGAGAATAC...-3'
CY029480  5'-...TGATTCCTGAAAGGAATGAACAAGGGCAGAC---GAATGTGAGAGGCTCAGGGATGAGAATAC...-3'
AY676021  5'-...TGATTCCTGAAAGGAATGAACAAGGGCAGAC---GAATGTGAGAGGCTCAGGAATGAGAATAC...-3'
CY029445  5'-...TGATTCCTGAAAGGAATGAACAAGGGCAGAC---GAATGTGAGAGGCTCAGGGATGAGAATAC...-3'
DQ237956  5'-...TGATTCCTGAAAGGAATGAACAAGGACAGAC---GAATGTGAGAGGCTCAGGAATGAGAATAC...-3'
CY029494  5'-...TGATTCCTGAAAGGAATGAACAAGGGCAGAC---GAATGTGAGAGGCTCAGGGATGAGAATAC...-3'
```

```
                224           235   240              254   1941              1956   1961      1969
                 *             *     *                *     *                 *      *         *
AY676024  5'-...TGGTTCCTGAAAGGAATGAACAAGGGCAGAC---GAATGTGAGAGGCTCAGGAATGAGAATAC...-3'
CY029431  5'-...TGATTCCTGAAAGGAATGAACAAGGGCAGAC---GAATGTGAGAGGCTCAGGGATGAGAATAC...-3'
AB166859  5'-...TGGTTCCTGAAAGAAATGAACAAGGGCAGAC---GAATGTGAGAGGCTCAGGAATGAGAATAC...-3'
AB188821  5'-...TGGTCCCTGAAAGGAATGAACAAGGGCAGAC---GAATGTGAGAGGCTCAGGAATGAGAATAC...-3'
AY585519  5'-...TGATTCCTGAAAGGAATGAACAAGGGCAGAC---GAATGTGAGAGGCTCAGGAATGAGAATAC...-3'
DQ095756  5'-...TGATTCCTGAAAGGAATGAACAAGGACAGAC---GAATGTGAGAGGCTCAGGAATGAGAATAC...-3'
DQ138169  5'-...TGATTCCTGAAAGGAATGAACAAGGGCAGAC---GAATGTGAGAGGCTCAGGAATGAGAATAC...-3'
DQ138166  5'-...TGATTCCTGAAAGGAATGAACAAGGGCAGAC---GAATGTGAGAGGCTCAGGAATGAGAATAC...-3'
AY651722  5'-...TGATTCCTGAAAGGAATGAACAAGGGCAGAC---GAATGTGAGAGGCTCAGGAATGAGAATAC...-3'
AY651727  5'-...TGATTCCTGAAAGGAATGAACAAGGGCAGAC---GAATGTGAGAGGCTCAGGAATGAGAATAC...-3'
DQ138170  5'-...TGATTCCTGAAAGGAATGAACAAGGGCAGAC---GAATGTGAGAGGCTCAGGAATGAGAATAC...-3'
DQ138176  5'-...TGATTCCTGAAAGGAATGAACAAGGGCAGAC---GAATGTGAGAGGCTCAGGAATGAGAATAC...-3'
AY651725  5'-...TGATTCCTGAAAGGAATGAACAAGGGCAGAC---GAATGTGAGAGGCTCAGGAATGAGAATAC...-3'
DQ138173  5'-...TGATTCCTGAAAGGAATGAACAAGGGCAGAC---GAATGTGAGAGGCTCAGGAATGAGAATAC...-3'
DQ138175  5'-...TGATTCCTGAAAGGAATGAACAAGGGCAGAC---GAATGTGAGAGGCTCAGGAATGAGAATAC...-3'
DQ138167  5'-...TGATTCCTGAAAGGAATGAACAAGGGCAGAC---GAATGTGAGAGGCTCAGGAATGAGAATAC...-3'
AB188813  5'-...TGGTTCCTGAAAGGAATGAACAAGGGCAGAC---GAATGTGAGAGGCTCAGGAATGAGAATAC...-3'
DQ138172  5'-...TGATTCCTGAAAGGAATGAACAAGGGCAGAC---GAATGTGAGAGGCTCAGGAATGAGAATAC...-3'
CY029452  5'-...TGATTCCTGAAAGGAATGAACAAGGGCAGAC---GAATGTGAGAGGCTCAGGGATGAGAATAC...-3'
DQ138178  5'-...TGATTCCTGAAAGGAATGAACAAGGGCAGAC---GAATGTGAGAGGCTCAGGAATGAGAATAC...-3'
DQ095761  5'-...TGATTCCTGAAAGGAATGAACAAGGACAGAC---GAATGTGAGAGGCTCAGGAATGAGAATAC...-3'
AY651716  5'-...TGATTCCTGAAAGGAATGAACAAGGGCAGAC---GAATGTGAGAGGCTCAGGAATGAGAATAC...-3'
DQ138180  5'-...TGATTCCTGAAAGGAATGAACAAGGGCAGAC---GAATGTGAGAGGCTCAGGAATGAGAATAC...-3'
DQ320833  5'-...TGATTCCTGAAAGGAATGAACAAGGGCAGAC---GAATGTGAGAGGCTCAGGAATGAGAATAC...-3'
HM172482  5'-...TGATTCCTGAAAGGAATGAACAAGGACAGAC---GAATGTGAGAGGCTCAGGAATGAGAATAC...-3'
DQ138168  5'-...TGATTCCTGAAAGGAATGAACAAGGGCAGAC---GAATGTGAGAGGCTCAGGAATGAGAATAC...-3'
AY651724  5'-...TGATTCCTGAAAGGAATGAACAAGGGCAGAC---GAATGTGAGAGGCTCAGGAATGAGAATAC...-3'
AY651723  5'-...TGATTCCTGAAAGGAATGAACAAGGGCAGAC---GAATGTGAGAGGCTCAGGAATGAGAATAC...-3'
AY651726  5'-...TGATTCCTGAAAGGAATGAACAAGGGCAGAC---GAATGTGAGAGGCTCAGGAATGAGAATAC...-3'
DQ138174  5'-...TGATTCCTGAAAGGAATGAACAAGGGCAGAC---GAATGTGAGAGGCTCAGGAATGAGAATAC...-3'
DQ835800  5'-...TGATTCCTGAAAGGAATGAACAAGGGCAGAC---GAATGTGAGAGGCTCAGGAATGAGAATAC...-3'
AY651715  5'-...TGATTCCTGAAAGGAATGAACAAGGGCAGAC---GAATGTGAGAGGCTCAGGAATGAGAATAC...-3'
CY029179  5'-...TGATTCCTGAAAGGAATGAACAAGGGCAGAC---GAATGTGAGAGGCTCAGGAATGAGAATAC...-3'
AY590581  5'-...TGATTCCTGAAAGGAATGAACAAGGGCAGAC---GAATGTGAGAGGCTCAGGAATGAGAATAC...-3'
DQ320835  5'-...TGATTCCTGAAAGGAATGAACAAGGGCAAAC---GAATGTGAGAGGCTCAGGAATGAGAATAC...-3'
CY029326  5'-...TGATTCCTGAAAGGAATGAACAAGGGCAGAC---GAATGTGAGAGGCTCAGGAATGAGAATAC...-3'
DQ138179  5'-...TGATTCCTGAAAGGAATGAACAAGGGCAGAC---GAATGTGAGAGGCTCAGGAATGAGAATAC...-3'
AB189058  5'-...TGGTTCCTGAAAGGAATGAACAAGGGCAGAC---GAATGTGAGAGGCTCAGGAATGAGAATAC...-3'
JQ794461  5'-...TGATTCCTGAAAGGAATGAACAAGGGCAGAC---GAATGTGAGAGGATCAGGAATGAGAATAC...-3'
JQ794453  5'-...TGATTCCTGAAAGGAATGAACAAGGGCAGAC---GAATGTGAGAGGATCAGGAATGAGAATAC...-3'
AY818128  5'-...TGATTCCTGAAAGGAATGAACAAGGGCAGAC---GAATGTGAGAGGCTCAGGAATGAGAATAC...-3'
AB450511  5'-...TGATTCCTGAAAGGAATGAACAAGGGCAGAC---GAATGTGAGAGGCTCAGGAATGAGAATAC...-3'
CY047480  5'-...TGATTCCTGAAAGGAATGAACAAGGACAAAC---GAATGTGAGAGGCTCAGGAATGAGAATAC...-3'
DQ992707  5'-...TGATTCCTGAAAGGAATGAACAAGGGCAGAC---GAATGTGAGAGGCTCAGGAATGAGAATAC...-3'
DQ992603  5'-...TGATTCCTGAAAGGAATGAACAAGGGCAGAC---GAATGTGAGAGGCTCAGGAATGAGAATAC...-3'
AB450503  5'-...TGATTCCTGAAAGGAATGAACAAGGGCAGAC---GAATGTGAGAGGCTCAGGAATGAGAATAC...-3'
AY818127  5'-...TGATTCCTGAAAGGAATGAACAAGGGCAGAC---GAATGTGAGAGGCTCAGGAATGAGAATAC...-3'
DQ236087  5'-...TGATTCCTGAAAGGAATGAACAAGGGCAGAC---GAATGTGAGAGGATCAGGAATGAGAATAC...-3'
EF523707  5'-...TGATTCCTGAAAGGAATGAACAGGGACAGAC---GAATGTGAGAGGCTCAGGAATGAGAATAC...-3'
EF523711  5'-...TGATTCCTGAAAGGAATGAACAGGGACAGAC---GAATGTGAGAGGCTCAGGAATGAGAATAC...-3'
CY047472  5'-...TGATTCCTGAAAGGAATGAACAAGGACAGAC---GAATGTGAGAGGCTCAGGAATGAGAATAC...-3'
FJ445233  5'-...TGATTCCTGAAAGGAATGAACAGGGACAGAC---GAATGTGAGAGGCTCAGGAATGAGAATAC...-3'
AB450512  5'-...TGATTCCTGAAAGGAATGAACAAGGGCAGAC---GAATGTGAGAGGCTCAGGAATGAGAATAC...-3'
DQ835805  5'-...TGGTTCCTGAAAGGAATGAACAAGGGCAGAC---GAATGTGAGAGGCTCAGGAATGAGAATAC...-3'
DQ320832  5'-...TGATTCCTGAAAGGAATGAACAAGGGCAGAC---GAATGTGAGAGGCTCAGGAATGAGAATAC...-3'
HM590794  5'-...TGATTCCTGAAAGGAATGAACAAGGGCAGAC---GAATGTGAGAGGCTCAGGAATGAGAATAC...-3'
HM590786  5'-...TGATTCCTGAAAGGAATGAACAAGGGCAGAC---GAATGTGAGAGGCTCAGGAATGAGAATAC...-3'
CY029466  5'-...TGATTCCTGAAAGGAATGAACAAGGGCAGAC---GAATGTGAGAGGCTCAGGGATGAGAATAC...-3'
AB450513  5'-...TGATTCCTGAAAGGAATGAACAAGGGCAGAC---GAATGTGAGAGGCTCAGGAATGAGAATAC...-3'
AY651713  5'-...TGATTCCTGAAAGGAATGAACAAGGGCAGAC---GAATGTGAGAGGCTCAGGAATGAGAATAC...-3'
CY029193  5'-...TGATTCCTGAAAGGAATGAACAAGGGCAGAC---GAATGTGAGAGGCTCAGGAATGAGAATAC...-3'
AY676023  5'-...TGGTTCCTGAAAGGAATGAACAAGGGCAGAC---GAATGTGAGAGGCTCTGGAATGAGAATAC...-3'
DQ095755  5'-...TGATTCCTGAAAGGAATGAACAAGGACAGAC---GAATGTGAGAGGCTCAGGAATGAGAATAC...-3'
EU875391  5'-...TGATTCCTGAAAGGAATGAACAAGGGCAGAC---GAATGTGAGAGGCTCAGGAATGAGAATAC...-3'
EF523712  5'-...TGATTCCTGAAAGGAATGAACAGGGACAGAC---GAATGTGAGAGGCTCAGGAATGAGAATAC...-3'
CY048304  5'-...TGATTCCTGAAAGGAATGAACAGGGACAGAC---GAATGTGAGAGGCTCAGGAATGAGAATAC...-3'
EF523713  5'-...TGATTCCTGAAAGGAATGAACAGGGACAGAC---GAATGTGAGAGGCTCAGGAATGAGAATAC...-3'
CY029242  5'-...TGGTTCCTGAAAGGAATGAACAAGGGCAGAC---GAATGTGAGAGGCTCAGGAATGAGAATAC...-3'
AY651748  5'-...TGATTCCTGAAAGGAATGAACAAGGGCAGAC---GAATGTGAGAGGCTCAGGAATGAGAATAC...-3'
EU874902  5'-...TGATTCCTGAAAGGAATGAACAAGGACAGAC---GAATGTGAGAGGCTCAGGAATGAGAATAC...-3'
```

```
                 224         235  240           254  1941           1956  1961     1969
                  *           *    *             *    *              *     *        *
CY048360  5'-...TGATTCCTGAAAGGAATGAACAGGGACAGAC---GAATGTGAGAGGCTCAGGAATGAGAATAC...-3'
AB576199  5'-...TGATTCCTGAAAGGAATGAACAAGGGCAGAC---GAATGTGAGAGGCTCAGGAATGAGAATAC...-3'
AY585515  5'-...TGATTCCTGAAAGGAATGAACAAGGGCAGAC---GAATGTGAGAGGCTCAGGAATGAGAATAC...-3'
HM627942  5'-...TGATTCCTGAAAGGAATGAACAAGGGCAGAC---GAATGTGAGAGGCTCAGGAATGAGAATAC...-3'
DQ992591  5'-...TGATTCCTGAAAGGAATGAACAAGGGCAGAC---GAATGTGAGAGGCTCAGGAATGAGAATAC...-3'
DQ835799  5'-...TGATTCCTGAAAGGAATGAACAAGGGCAGAC---GAATGTGAGAGGCTCAGGAATGAGAATAC...-3'
EU748900  5'-...TGATTCCTGAAAGGAATGAACAAGGACAGAC---GAATGTTAGAGGCTCAGGAATGAGAATAC...-3'
FJ445241  5'-...TGATTCCTGAAAGGAATGAACAAGGACAGAC---GAATGTTAGAGGCTCAGGAATGAGAATAC...-3'
EF523710  5'-...TGATTCCTGAAAGGAATGAACAGGGACAGAC---GAATGTGAGAGGCTCAGGAATGAGAATAC...-3'
DQ992601  5'-...TGATTCCTGAAAGGAATGAACAAGGGCAGAC---GAATGTGAGAGGCTCAGGAATGAGAATAC...-3'
CY034213  5'-...TGATTCCTGAAAGGAATGAACAAGGGCAGAC---GAATGTGAGAGGCTCAGGAATGAGAATAC...-3'
EF523709  5'-...TGATTCCTGAAAGGAATGAACAGGGACAGAC---GAATGTGAGAGGCTCAGGAATGAGAATAC...-3'
DQ992597  5'-...TGATTCCTGAAAGGAATGAACAAGGGCAGAC---GAATGTGAGAGGCTCAGGAATGAGAATAC...-3'
DQ992623  5'-...TGATTCCTGAAAGGAATGAACAAGGGCAGAC---GAATGTGAGAGGCTCAGGAATGAGAATAC...-3'
DQ992708  5'-...TGATTCCTGAAAGGAATGAACAAGGGCAGAC---GAATGTGAGAGGCTCAGGAATGAGAATAC...-3'
EU296249  5'-...TGATTCCTGAAAGGAATGAACAGGGACAGAC---GAATGTGAGAGGCTCAGGAATGAGAATAC...-3'
DQ992611  5'-...TGATTCCTGAAAGGAATGAACAAGGGCAGAC---GAATGTGAGAGGCTCAGGAATGAGAATAC...-3'
EF523714  5'-...TGATTCCTGAAAGGAATGAACAGGGACAGAC---GAATGTGAGAGGCTCAGGAATGAGAATAC...-3'
EU430499  5'-...TGGTTCCTGAAAGGAATGAACAAGGGCAGAC---GAATGTGAGAGGCTCAGGAATGAGAATAC...-3'
CY034773  5'-...TGATTCCTGAAAGGAATGAACAAGGGCAGAC---GAATGTGAGAGGCTCAGGAATGAGAATAC...-3'
CY029312  5'-...TGATTCCTGAAAGGAATGAACAAGGGCAAAC---GAATGTGAGAGGCTCAGGAATGAGAATAC...-3'
EF523708  5'-...TGATTCCTGAAAGGAATGAACAGGGACAGAC---GAATGTGAGAGGCTCAGGAATGAGAATAC...-3'
DQ992571  5'-...TGATTCCTGAAAGGAATGAACAAGGGCAGAC---GAATGTGAGAGGCTCAGGAATGAGAATAC...-3'
DQ138177  5'-...TGATTCCTGAAAGGAATGAACAAGGGCAGAC---GAATGTGAGAGGCTCAGGAATGAGAATAC...-3'
CY029487  5'-...TGATTCCTGAAAGGAATGAACAAGGGCAGAC---GAATGTGAGAGGCTCAGGGATGAGAATAC...-3'
FJ445249  5'-...TGATTCCTGAAAGGAATGAACAAGGACAGAC---GAATGTTAGAGGCTCAGGAATGAGAATAC...-3'
CY048008  5'-...TGATTCCTGAAAGGAATGAACAGGGACAGAC---GAATGTGAGAGGCTCAGGAATGAGAATAC...-3'
EU697231  5'-...TGATTCCTGAAAGGAATGAACAAGGACAGAC---GAATGTTAGAGGCTCAGGAATGAGAATAC...-3'
EF523706  5'-...TGATTCCTGAAAGGAATGAACAGGGACAGAC---GAATGTGAGAGGCTCAGGAATGAGAATAC...-3'
DQ992593  5'-...TGATTCCTGAAAGGAATGAACAAGGGCAGAC---GAATGTGAGAGGCTCAGGAATGAGAATAC...-3'
DQ992602  5'-...TGATTCCTGAAAGGAATGAACAAGGGCAGAC---GAATGTGAGAGGCTCAGGAATGAGAATAC...-3'
DQ992600  5'-...TGATTCCTGAAAGGAATGAACAAGGGCAGAC---GAATGTGAGAGGCACAGGAATGAGAATAC...-3'
CY030918  5'-...TGATTCCTGAAAGGAATGAACAAGGGCAGAC---GAATGTGAGAGGCTCAGGAATGAGAATAC...-3'
CY030910  5'-...TGATTCCTGAAAGGAATGAACAAGGGCAGAC---GAATGTGAGAGGCTCAGGAATGAGAATAC...-3'
CY030878  5'-...TGATTCCTGAAAGGAATGAACAAGGGCAGAC---GAATGTGAGAGGCTCAGGAATGAGAATAC...-3'
CY030926  5'-...TGATTCCTGAAAGGAATGAACAAGGGCAGAC---GAATGTGAGAGGCTCAGGAATGAGAATAC...-3'
DQ992592  5'-...TGATTCCTGAAAGGAATGAACAAGGGCAGAC---GAATGTGAGAGGCTCAGGAATGAGAATAC...-3'
DQ992594  5'-...TGATTCCTGAAAGGAATGAACAAGGGCAGAC---GAATGTGAGAGGCTCAGGAATGAGAATAC...-3'
AB525188  5'-...TGATTCCTGAAAGGAATGAACAAGGACAGAC---GAATGTGAGAGGCTCAGGAATGAGAATAC...-3'
CY029501  5'-...TGATTCCTGAAAGGAATGAACAAGGGCAGAC---GAATGTGAGAGGCTCAGGGATGAGAATAC...-3'
EU429983  5'-...TGATTCCTGAAAGGAATGAACAAGGACAGAC---GAATGTTAGAGGCTCAGGAATGAGAATAC...-3'
EU429985  5'-...TGATTCCTGAAAGGAATGAACAAGGACAGAC---GAATGTTAGAGGCTCAGGAATGAGAATAC...-3'
EU430505  5'-...TGGTTCCTGAAAGGAATGAACAAGGGCAGAC---GAATGTGAGAGGCTCAGGAATGAGAATAC...-3'
CY048272  5'-...TGATTCCTGAAAGGAATGAACAGGGACAGAC---GAATGTGAGAGGCTCAGGAATGAGAATAC...-3'
DQ529292  5'-...TGATTCCTGAAAGGAAYGAACAAGGACAGAC---GAATGTTAGAGGCTCAGGAATGAGAATAC...-3'
AB450507  5'-...TGATTCCTGAAAGGAATGAACAAGGGCAGAC---GAATGTGAGAGGATCAGGAATGAGAATAC...-3'
EU277848  5'-...TGATTCCTGAAAGGAATGAACAAGGACAGAC---GAATGTTAGAGGCTCAGGAATGAGAATAC...-3'
EU233682  5'-...TGATTCCTGAAAGGAATGAACAAGGACAGAC---GAATGTGAGAGGCTCAGGAATGAGAATAC...-3'
EU233690  5'-...TGATTCCTGAAAGGAATGAACAAGGACAGAC---GAATGTGAGAGGCTCAGGAATGAGAATAC...-3'
CY029004  5'-...TGATTCCTGAAAGGAATGAACAAGGGCAGAC---GAATGTGAGAGGCTCAGGAATGAGAATAC...-3'
CY048056  5'-...TGATTCCTGAAAGGAATGAACAGGGACAGAC---GAATGTGAGAGGCTCAGGAATGAGAATAC...-3'
CY048200  5'-...TGATTCCTGAAAGGAATGAACAGGGACAGAC---GAATGTGAGAGGCTCAGGAATGAGAATAC...-3'
DQ992691  5'-...TGATTCCTGAAAGGAATGAACAAGGGCAGAC---GAATGTGAGAGGCTCAGGAATGAGAATAC...-3'
DQ992576  5'-...TGATTCCTGAAAGGAATGAACAAGGGCAGAC---GAATGTGAGAGGCTCAGGAATGAGAATAC...-3'
AB450508  5'-...TGATTCCTGAAAGGAATGAACAAGGGCAGAC---GAATGTGAGAGGATCAGGAATGAGAATAC...-3'
DQ992706  5'-...TGATTCCTGAAAGGAATGAACAAGGGCAGAC---GAATGTGAGAGGCTCAGGAATGAGAATAC...-3'
EU697223  5'-...TGATTCCTGAAAGGAATGAACAAGGACAGAC---GAATGTTAGAGGCTCAGGAATGAGAATAC...-3'
CY048464  5'-...TGATTCCTGAAAGGAATGAACAGGGACAAAC---GAATGTGAGAGGCTCAGGAATGAGAATAC...-3'
EU233714  5'-...TGATTCCTGAAAGGAATGAACAAGGACAGAC---GAATGTGAGAGGCTCAGGAATGAGAATAC...-3'
EU233722  5'-...TGATTCCTGAAAGGAATGAACAAGGACAGAC---GAATGTGAGAGGCTCAGGAATGAGAATAC...-3'
AY059529  5'-...TGATTCCTGAAAGGAATGAACAAGGGCAGAC---GAATGTGAGAGGCTCAGGAATGAGAATAC...-3'
DQ992595  5'-...TGATTCCTGAAAGGAATGAACAAGGGCAGAC---GAATGTGAGAGGCTCAGGAATGAGAATAC...-3'
DQ992598  5'-...TGATTCCTGAAAGGAATGAACAAGGGCAGAC---GAATGTGAGAGGCTCAGGAATGAGAATAC...-3'
DQ992705  5'-...TGATTCCTGAAAGGAATGAACAAGGGCAGAC---GAATGTGAGAGGCTCAGGAATGAGAATAC...-3'
EU429986  5'-...TGATTCCTGAAAGGAATGAACAAGGACAGAC---GAATGTTAGAGGCTCAGGAATGAGAATAC...-3'
AY059525  5'-...TGATTCCTGAAAGGAATGAACAAGGGCAGAC---GAATGTGAGAGGCTCAGGAATGAGAATAC...-3'
AY651751  5'-...TGGTTCCTGAAAGGAATGAACAAGGGCAGAC---GAATGTGAGAGGCTCAGGAATGAGAATAC...-3'
HM172412  5'-...TGATTCCTGAAAGGAATGAACAAGGGCAGAC---GAATGTGAGAGGCTCAGGAATGAGAATAC...-3'
HM172429  5'-...TGATTCCTGAAAGGAATGAACAAGGGCAGAC---GAATTTGAAAGGCTCAGGAATGAAAATAC...-3'
CY048576  5'-...TGATTCCTGAAAGGAATGAACAGGGACAGAC---GAATGTGAGAGGCTCAGGAATGAGAATAT...-3'
```

```
                 224        235  240           254 1941           1956 1961    1969
                 *          *    *             *   *              *    *       *
CY048536   5'-...TGATTCCTGAAAGGAATGAACAAGGACAGAC---GAATGTTAGAGGCTCAGGAATGAGAATAC...-3'
EU233730   5'-...TGATTCCTGAAAGGAATGAACAAGGACAGAC---GAATGTGAGAGGCTCAGGAATGAGAATAC...-3'
HM172437   5'-...TGGTTCCTGAAAGGAATGAACAAGGACAGAC---GAATGTGAGAGGCTCAGGAATGAGAATAC...-3'
AB450514   5'-...TGATTCCTGAAAGGAATGAACAAGGGCAGAC---GAATGTGAGAGGCTCAGGAATGAGAATAC...-3'
AB450517   5'-...TGATTCCTGAAAGGAATGAACAAGGGCAGAC---GAATGTGAGAGGCTCAGGAATGAGAATAC...-3'
AB450515   5'-...TGATTCCTGAAAGGAATGAACAAGGGCAGAC---GAATGTGAGAGGCTCAGGAATGAGAATAC...-3'
DQ992589   5'-...TGGTTCCTGAAAGGAATGAACAAGGGCAGAC---GAATGTGAGAGGCTCAGGAATGAGAATAC...-3'
EU429984   5'-...TGATTCCTGAAAGGAATGAACAAGGACAGAC---GAATGTTAGAGGCTCAGGAATGAGAATAC...-3'
AB478014   5'-...TGATTCCTGAAAGGAATGAACAAGGGCAGAC---GAACGTGAGGGGCTCAGGAATGAGAATAC...-3'
EF178516   5'-...TGATTCCTGAAAGGAATGAACAAGGGCAGAC---GAATGTGAGAGGATCAGGAATGAGAATAC...-3'
AB450516   5'-...TGATTCCTGAAAGGAATGAACAAGGGCAGAC---GAATGTGAGAGGCTCAGGAATGAGAATAC...-3'
EU697216   5'-...TGATTCCTGAAAGGAATGAACAAGGACAGAC---GAATGTTAGAGGCTCAGGAATGAGAATAC...-3'
DQ533586   5'-...TGATTCCTGAAAGGAATGAACAAGGACAGAC---GAATGTGAGAGGCTCAGGAATGAGAATAC...-3'
EU429987   5'-...TGATTCCTGAAAGGAATGAACAAGGACAGAC---GAATGTTAGAGGCTCAGGAATGAGAATAC...-3'
EU233706   5'-...TGATTCCTGAAAGGAATGAACAAGGACAGAC---GAATGTGAGAGGCTCAGGAATGAGAATAC...-3'
CY036066   5'-...TGATCCCTGAAAGGAATGAACAAGGGCAGAC---GAATGTGAGAGGCTCAGGAATGAGGATAC...-3'
CY036098   5'-...TGATCCCTGAAAGGAATGAACAAGGGCAGAC---GAATGTGAGAGGCTCAGGAATGAGGATAC...-3'
JX420195   5'-...TGATTCCTGAAAGGAATGAACAAGGGCAGAC---GAATGTGAGAGGCTCAGGAATGAGAATAC...-3'
AY221587   5'-...TGATTCCTGAAAGGAATGAACAAGGGCAGAC---GAATGTGAGAGGCTCAGGAATGAGAATAC...-3'
CY048208   5'-...TGATTCCTGAAAGGAATGAACAGGGACAGAC---GAATGTGAGAGGCTCAGGAATGAGAATAC...-3'
CY030902   5'-...TGATTCCTGAAAGGAATGAACAAGGGCAGAC---GAATGTGAGAGGCTCAGGAATGAGAATAC...-3'
CY048296   5'-...TGATTCCTGAAAGGAATGAACAGGGACAGAC---GAATGTGAGAGGCTCAGGAATGAGAATAC...-3'
CY029319   5'-...TGGTTCCTGAAAGGAATGAACAAGGGCAGAC---GAATGTGAGAGGCTCAGGAATGAGAATAC...-3'
HM172423   5'-...TGATTCCTGAAAGGAATGAACAAGGACAGAC---GAATGTGAGAGGCTCAGGAATGAGAATAC...-3'
AB450504   5'-...TGATTCCTGAAAGGAATGAACAAGGGCAGAC---GAATGTGAGAGGCTCAGGAATGAGAATAC...-3'
CY048240   5'-...TGATTCCTGAAAGGAATGAACAGGGACAGAC---GAATGTAAGAGGCTCAGGAATGAGAATAC...-3'
CY048232   5'-...TGATTCCTGAAAGGAATGAACAGGGACAGAC---GAATGTAAGAGGCTCAGGAATGAGAATAC...-3'
AB496982   5'-...TGATTCCTGAAAGGAATGAACAAGGACAGAC---GAATGTTAGAGGCTCAGGAATGAGAATAC...-3'
CY036082   5'-...TGATCCCTGAAAGGAATGAACAAGGGCAGAC---GAATGTGAGAGGCTCAGGAATGAGGATAC...-3'
HM172405   5'-...TGATTCCTGAAAGGAATGAACAAGGGCAGAC---GAATGTGAGAGGCTCAGGAATGAGAATAC...-3'
CY029249   5'-...TGATTCCTGAAAGGAATGAACAAGGGCAGAC---GAATGTAAGAGGCTCAGGAATGAGAATAC...-3'
CY029235   5'-...TGATTCCTGAAAGGAATGAACAAGGGCAGAC---GAATGTGAGAGGCTCAGGAATGAGAATAC...-3'
CY048264   5'-...TGATTCCTGAAAGGAATGAACAGGGACAGAC---GAATGTGAGAGGCTCAGGAATGAGAATAC...-3'
DQ334757   5'-...TGATTCCTGAAAGGAATGAACAAGGGCAGAC---GAATGTGAGAGGATCAGGAATGAGAATAC...-3'
AB450509   5'-...TGATTCCTGAAAGGAATGAACAAGGGCAGAC---GAATGTGAGAGGATCAGGAATGAGAATAC...-3'
DQ334773   5'-...TGATTCCTGAAAGGAATGAACAAGGGCAGAC---GAATGTGAGAGGATCAGGAATGAGAATAC...-3'
CY046109   5'-...TGATTCCTGAAAGGAATGAACAAGGACAGAC---GAATGTGAGAGGCTCAGGAATGAGAATAC...-3'
JX420187   5'-...TGATTCCTGAAAGGAATGAACAAGGGCAGAC---GAATGTGAGAGGCTCAGGAATGAGAATAC...-3'
AY585508   5'-...TGATTCCTGAAAGGAATGAACAAGGGCAGAC---GAATGTGAGAGGCTCAGGAATGAGAATAC...-3'
CY048416   5'-...TGATTCCTGAAAGGAATGAACAAGGACAGAC---GAATGTTAGAGGCTCAGGAATGAGAATAC...-3'
DQ890068   5'-...TGATTCCTGCAAGGAATGAACAAGGACAGAC---GAATGTGAGAGGCTCAGGAATGAGAATAC...-3'
AY221584   5'-...TGATTCCTGAAAGGAATGAACAAGGGCAGAC---GAATGTGAGAGGCTCAGGAATGAGAATAC...-3'
CY036074   5'-...TGATCCCTGAAAGGAATGAACAAGGGCAGAC---GAATGTGAGAGGCTCAGGAATGAGGATAC...-3'
AB496981   5'-...TGATTCCTGAAAGGAATGAACAAGGACAGAC---GAATGTTAGAGGCTCAGGAATGAGAATAC...-3'
AY221586   5'-...TGATTCCTGAAAGGAATGAACAAGGGCAGAC---GAATGTGAGAGGCTCAGGAATGAGAATAC...-3'
DQ992572   5'-...TGATTCCTGAAAGGAATGAACAAGGGCAGAC---GAATGTGAGAGGCTCAGGAATGAGAATAC...-3'
DQ992647   5'-...TGATTCCTGAAAGGAATGAACAAGGGCAGAC---GAACGTGAGAGGCTCAGGAATGAGAATAC...-3'
CY036146   5'-...TGATCCCTGAAAGGAATGAACAAGGGCAGAC---GAATGTGAGAGGCTCAGGAATGAGGATAC...-3'
AB496984   5'-...TGATTCCTGAAAGGAATGAGCAAGGACAGAC---GAATGTTAGAGGCTCAGGAATGAGAATAC...-3'
AB496985   5'-...TGATTCCTGAAAGGAATGAGCAAGGACAGAC---GAATGTTAGAGGCTCAGGAATGAGAATAC...-3'
CY030934   5'-...TGATTCCTGAAAGGAATGAACAAGGGCAGAC---GAATGTGAGAGGCTCAGGAATGAGAATAC...-3'
AB496983   5'-...TGATTCCTGAAAGGAATGAACAAGGACAGAC---GAATGTTAGAGGCTCAGGAATGAGAATAC...-3'
JF732743   5'-...TGATTCCTGAAAGGAATGAACAAGGGCAGAC---GAATGTGAGAGGCTCAGGAATGAGAATAC...-3'
AB450505   5'-...TGATTCCTGAAAGGAATGAACAAGGGCAGAC---GAATGTGAGAGGCTCAGGAATGAGAATAC...-3'
DQ835806   5'-...TGATTCCTGAAAGGAATGAACAAGGGCAGAC---GAATGTGAGAGGCTCAGGAATGAGAATAC...-3'
AY221591   5'-...TGATTCCTGAAAGGAATGAACAAGGGCAGAC---GAATGTGAGAGGCTCAGGAATGAGAATAC...-3'
EU700507   5'-...TGATTCCTGAAAGGAATGAACAAGGACAGAC---GAATGTGAGAGGCTCAGGAATGAGAATAC...-3'
FM177116   5'-...TGATTCCTGAAAGGAATGAACAAGGACAGAC---GAATGTGAGAGGCTCAGGAATGAGAATAC...-3'
CY048320   5'-...TGATTCCTGAAAGGAATGAACAGGGACAGAC---GAATGTGAGAGGCTCAGGAATGAGAATAC...-3'
CY040915   5'-...TGATTCCTGAAAGGAATGAACAAGGGCAGAC---GAATGTGAGAGGCTCAGGAATGAGAATAC...-3'
AB465626   5'-...TGATTCCTGAAAGGAATGAGCAAGGACAGAC---GAATGTTAGAGGCTCAGGAATGAGAATAC...-3'
DQ334765   5'-...TGATTCCTGAAAGGAATGAACAAGGGCAGAC---GAATGTGAGAGGATCAGGAATGAGAATAC...-3'
CY034237   5'-...TGATCCCTGAAAGGAATGAACAAGGGCAGAC---GAATGTGAGAGGCTCAGGAATGAGAATAC...-3'
DQ992682   5'-...TGATTCCTGAAAGGAATGAACAAGGGCAGAC---GAATGTGAGAGGCTCAGGAATGAGAATAC...-3'
CY029540   5'-...TGATTCCTGAAAGGAATGAACAAGGGCAGAC---GAATGTGAGAGGCTCAGGAATGAGAATAC...-3'
CY048328   5'-...TGATTCCTGAAAGGAATGAACAGGGACAGAC---GAATGTGAGAGGCTCAGGAATGAGAATAC...-3'
DQ992672   5'-...TGGTTCCTGAAAGGAATGAACAAGGGCAGAC---GAATGTGAGAGGCTCAGGAATGAGAATAC...-3'
DQ320848   5'-...TGATTCCTGAAAGGAATGAACAAGGGCAGAC---GAATGTGAGAGGCTCAGGAATGAGAATAC...-3'
CY030974   5'-...TGATTCCTGAAAGGAATGAACAAGGGCAGAC---GAATGTGAGAGGCTCAGGAATGAGAATAC...-3'
FM174393   5'-...TGATTCCTGAAAGGAATGAACAAGGACAGAC---GAATGTGAGAGGCTCAGGAATGAGAATAC...-3'
```

```
                224        235  240           254  1941          1956 1961    1969
                 *          *    *             *   *              *    *       *
DQ890067   5’-...TGATTCCTGAAAGGAATGAACAAGGACAGAC---GAATGTGAGAGGCTCAGGAATGAGAATAC...-3’
HM172448   5’-...TGGTTCCTGAAAGGAATGAACAAGGGCAGAC---GAATGTGAGAGGCTCAGGAATGAGAATAC...-3’
AB450502   5’-...TGATTCCTGAAAGGAATGAACAAGGGCAGAC---GAATGTGAGAGGCTCAGGAATGAGAATAC...-3’
EF605591   5’-...TGATTCCTGAAAGGAATGAACAAGGACAGAC---GAATGTGAGAGGTTCAGGAATGAGAATAC...-3’
CY048368   5’-...TGATTCCTGAAAGGAATGAACAGGGACAGAC---GAATGTGAGAGGCTCAGGAATGAGAATAC...-3’
FM174392   5’-...TGATTCCTGAAAGGAATGAACAAGGACAGAC---GAATGTGAGAGGCTCAGGAATGAGAATAC...-3’
HM172447   5’-...TGGTTCCTGAAAGGAATGAACAAGGGCAGAC---GAATGTGAGAGGCTCAGGAATGAGAATAC...-3’
CY048312   5’-...TGATTCCTGAAAGGAATGAACAGGGACAGAC---GAATGTGAGAGGCTCAGGAATGAGAATAC...-3’
CY048344   5’-...TGATTCCTGAAAGGAATGAACAGGGACAGAC---GAATGTGAGAGGCTCAGGAATGAGAATAC...-3’
CY048520   5’-...TGATTCCTGAAAGGAATGAACAGGGACAAAC---GAATGTGAGAGGCTCAGGAATGAGAATAC...-3’
AB478013   5’-...TGGTTCCTGAAAGGAATGAACAAGGGCAGAC---GAATGTGAGAGGCTCAGGAATGAGAATAC...-3’
JQ794469   5’-...TGATTCCTGAAAGGAATGAACAAGGGCAGAC---GAATGTGAGAGGCTCAGGAATGAGAATAC...-3’
DQ492835   5’-...TGATTCCTGAAAGGAATGAACAAGGGCAGAC---GAATGTGAGAGGCTCTGGAATGAGAATAC...-3’
DQ492830   5’-...TGATTCCTGAAAGGAATGAACAAGGGCAGAC---GAATGTGAGAGGCTCTGGAATGAGAATAC...-3’
DQ492836   5’-...TGATTCCTGAAAGGAATGAACAAGGGCAGAC---GAATGTGAGAGGCTCTGGAATGAGAATAC...-3’
DQ492833   5’-...TGATTCCTGAAAGGAATGAACAAGGGCAGAC---GAATGTGAGAGGCTCTGGAATGAGAATAC...-3’
DQ351870   5’-...TGATTCCTGAAAGGAATGAACAAGGGCAGAC---GAATGTGAGAGGCTTAGGAATGAGAATAC...-3’
AB450510   5’-...TGATTCCTGAAAGGAATGAACAAGGGCAGAC---GAATGTGAGAGGCTCAGGAATGAGAATAC...-3’
AB450506   5’-...TGATTCCTGAAAGGAATGAACAAGGGCAGAC---GAATGTGAGAGGCTCAGGAATGAGAATAC...-3’
HM172442   5’-...TGGTTCCTGAAAGGAATGAACAAGGGCAGAC---GAATGTGAGAGGCTCAGGAATGAGAATAC...-3’
DQ992643   5’-...TGGTTCCTGAAAGGAATGAACAAGGGCAGAC---GAATGTGAGAGGCTCAGGAATGAGAATAC...-3’
DQ992641   5’-...TGGTTCCTGAAAGGAATGAACAAGGGCAGAC---GAATGTGAGAGGCTCAGGAATGAGAATAC...-3’
CY030536   5’-...TGATTCCTGAAAGGAATGAACAAGGGCAGAC---GAATGTGAGAGGCTCAGGAATGAGAATAC...-3’
CY030725   5’-...TGATTCCTGAAAGGAATGAACAAGGGCAGAC---GAATGTGAGAGGCTCAGGAATGAGAATAC...-3’
CY080392   5’-...TGATTCCTGAAAGGAATGAACAAGGACAGAC---GAATGTGAGAGGCTCAGGAATGAGAATAC...-3’
CY030380   5’-...TGATCCCTGAAAGGAATGAACAAGGGCAGAC---GAATGTGAGAGGCTCAGGAATGAGGATAC...-3’
EU148443   5’-...TGATTCCTGAAAGGAATGAACAGGGACAGAC---GAATGTGAGAGGCTCCGGAATGAGAATAC...-3’
CY046100   5’-...TGATTCCTGAAAGGAATGAACAAGGACAGAC---GAATGTGAGAGGCTCAGGAATGAGAATAC...-3’
GQ122424   5’-...TGATTCCTGAAAGGAATGAACAAGGGCAGAC---GAATGTGAGAGGCTCTGGAATGAGAATAC...-3’
AY221585   5’-...TGATTCCTGAAAGGAATGAACAAGGGCAGAC---GAATGTGAGAGGCTCAGGAATGAGAATAC...-3’
FJ719831   5’-...TGATTCCTGAAAGGAATGAACAAGGACAGAC---GAATGTGAGAGGCTCAGGAATGAGAATAC...-3’
CY030894   5’-...TGATTCCTGAAAGGAATGAACAAGGGCAGAC---GAATGTGAGAGGCTCAGGAATGAGAATAC...-3’
FM174402   5’-...TGATTCCTGAAAGGAATGAACAAGGACAGAC---GAATGTGAGAGGCTCAGGAATGAGAATAC...-3’
HM172483   5’-...TGGTCCCTGAAAGGAATGAACAAGGGCAGAC---GAATGTGAGAGGCTCAGGAATGAAAATAC...-3’
CY030466   5’-...TGATTCCTGAAAGGAATGAACAAGGGCAGAC---GAATGTGAGAGGCTCAGGAATGAGAATAC...-3’
CY030490   5’-...TGATTCCTGAAAGGAATGAACAAGGGCAGAC---GAATGTGAGAGGCTCAGGAATGAGAATAC...-3’
CY048472   5’-...TGATTCCTGAAAGGAATGAACAGGGACAGAC---GAATGTGAGAGGCTCAGGAATGAGAATAC...-3’
CY048504   5’-...TGATTCCTGAAAGGAATGAACAGGGACAGAC---GAATGTGAGAGGCTCAGGAATGAGAATAC...-3’
CY041035   5’-...TGATTCCTGAAAGGAATGAACAAGGGCAGAC---GAATGTGAGAGGATCAGGAATGAGAATAC...-3’
AF509161   5’-...TGATTCCTGAAAGGAATGAACAAGGGCAGAC---GAATGTGAGAGGCTCAGGAATGAGAATAC...-3’
AF509152   5’-...TGATTCCTGAAAGGAATGAACAAGGGCAGAC---GAATGTGAGAGGCTCAGGAATGAGAATAC...-3’
AY585514   5’-...TGATTCCTGAAAGGAATGAACAAGGGCAGAC---GAATGTGAGAGGCTCAGGAATGAGAATAC...-3’
CY030528   5’-...TGATTCCTGAAAGGAATGAACAAGGGCAGAC---GAATGTGAGAGGCTCAGGAATGAGAATAC...-3’
CY029524   5’-...TGATTCCTGAAAGGAATGAACAAGGGCAGAC---GAATGTGAGAGGCTCAGGAATGAGAATAC...-3’
CY048528   5’-...TGATTCCTGAAAGGAATGAACAGGGACAGAC---GAATGTGAGAGGCTCAGGAATGAGAATAC...-3’
CY030723   5’-...TGATTCCTGAAAGGAATGAACAAGGGCAGAC---GAATGTGAGAGGCTCAGGAATGAGAATAC...-3’
CY030458   5’-...TGATTCCTGAAAGGAATGAACAAGGGCAGAC---GAATGTGAGAGGCTCAGGAATGAGAATAC...-3’
DQ320855   5’-...TGGTCCCTGAAAGGAATGAACAAGGGCAGAC---GAATGTGAGAGGCTCAGGAATGAGAATAC...-3’
FM174389   5’-...TGATTCCTGAAAGGAATGAACAAGGACAGAC---GAATGTGAGAGGCTCAGGAATGAGAATAC...-3’
DQ999878   5’-...TGATTCCTGAAAGGAATGAACAAGGGCAGAC---GAATGTGAGAGGCTCAGGAATGAGAATAC...-3’
DQ492831   5’-...TGATTCCTGAAAGGAATGAACAAGGGCAGAC---GAATGTGAGAGGCTCTGGAATGAGAATAC...-3’
FM174396   5’-...TGATTCCTGAAAGGAATGAACAAGGACAGAC---GAATGTGAGAGGCTCAGGAATGAGAATAC...-3’
JX420139   5’-...TGATTCCTGAAAGGAATGAACAAGGGCAGAC---GAATGTGAGAGGCTCAGGAATGAGAATAC...-3’
DQ999884   5’-...TGATTCCTGAAAGGAATGAACAAGGGCAGAC---GAATGTGAGAGGCTCAGGAATGAGAATAC...-3’
HM172445   5’-...TGGTTCCTGAAAGGAATGAACAAGGGCAGAC---GAATGTGAGAGGCTCAGGAATGAGAATAC...-3’
DQ320864   5’-...TGATTCCTGAAAGGAATGAACAAGGGCAGAC---GAATGTGAGAGGCTCTGGAATGAGAATAC...-3’
HM172432   5’-...TGATTCCTGAAAGGAATGAACAAGGGCAGAC---GAATGTAAGAGGCTCTGGAATGAGAATAC...-3’
CY047466   5’-...TGATTCCTGAAAGGAATGAACAAGGACAGAC---GAATGTGAGAGGCTCAGGAATGAGAATAC...-3’
CY030396   5’-...TGATTCCTGAAAGGAATGAACAAGGGCAGAC---GAATGTGAGAGGCTCAGGAATGAGAATAC...-3’
AB465625   5’-...TGATTCCTGAAAGGAATGAACAAGGACAGAC---GAATGTTAGAGGCTCAGGAATGAGAATAC...-3’
HM172446   5’-...TGGTTCCTGAAAGGAATGAACAAGGGCAGAC---GAATGTGAGAGGCTCAGGAATGAGAATAC...-3’
DQ835804   5’-...TGATTCCTGAAAGGAATGAACAAGGGCAGAC---GAATGTGAGAGGCTCAGGAATGAGAATAC...-3’
CY046067   5’-...TGATTCCTGAAAGGAATGAACAAGGACAGAC---GAATGTGAGAGGCTCAGGAATGAGAATAC...-3’
JQ794477   5’-...TGATTCCTGAAAGGAATGAACAAGGGCAGAC---GAATGTGAGAGGCTCAGGAATGAGAATAC...-3’
CY029676   5’-...TGATCCCTGAAAGGAATGAACAAGGGCAGAC---GAATGTGAGAGGCTCAGGAATGAGGATAC...-3’
JX241044   5’-...TGATCCCTGAAAGGAATGAACAAGGGCAGAC---GAATGTGAGAGGCTCAGGAATGAGGATAC...-3’
CY040896   5’-...TGATTCCTGAAAGGAATGAACAAGGGCAGAC---GAATGTGAGAGGCTCAGGAATGAGAATAC...-3’
CY036090   5’-...TGATTCCTGAAAGGAATGAACAAGGGCAGAC---GAATGTGAGAGGCTCAGGAATGAGAATAC...-3’
AB496989   5’-...TGATTCCTGAAAGGAATGAACAAGGACAGAC---GAATGTTAGAGGCTCAGGAATGAGAATAC...-3’
AB496990   5’-...TGATTCCTGAAAGGAATGAACAAGGACAGAC---GAATGTTAGAGGCTCAGGAATGAGAATAC...-3’
```

```
                   224          235  240            254  1941            1956  1961      1969
                    *            *    *              *    *               *     *         *
AB551129  5'-...TGATTCCTGAAAGGAATGAACAAGGACAGAC---GAATGTTAGAGGCTCAGGAATGAGAATAC...-3'
CY111078  5'-...TGATTCCTGAAAGGAATGAACAAGGACAGAC---GAATGTGAGAGGCTCAGGAATGAGAATAC...-3'
DQ320853  5'-...TGGTCCCTGAAAGGAATGAACAAGGGCAGAC---GAATGTGAGAGGCTCAGGAATGAGAATAC...-3'
DQ890070  5'-...TGATTCCTGCAAGGAATGAACAAGGACAGAC---GAATGTGAGAGGCTCAGGAATGAGAATAC...-3'
CY090105  5'-...TGATTCCTGAAAGGAATGAACAAGGACAGAC---GAATGTGAGAGGCTCAGGAATGAGAATAC...-3'
AY221590  5'-...TGATTCCTGAAAGGAATGAACAAGGGCAGAC---GAATGTGAGAGGCTCAGGAATGAGAATAC...-3'
EU616850  5'-...TGATTCCTGAAAGGAATGAACAAGGGCAGAC---GAATGTGAGAGGGTCAGGAATGAGAATAC...-3'
CY048448  5'-...TGATTCCTGAAAGGAATGAACAGGGACAGAC---GAATGTGAGAGGCTCCGGAATGAGAATAC...-3'
GQ122430  5'-...TGATTCCTGAAAGGAATGAACAAGGGCAGAC---GAATGTGAGAGGCTCTGGAATGAGAATAC...-3'
CY034709  5'-...TGATTCCTGAAAGGAATGAACAAGGGCAGAC---GAATGTGAGAGGCTCAGGAATGAGAATAC...-3'
CY030364  5'-...TGATACCTGAAAGGAATGAACAAGGGCAGAC---GAATGTGAGAGGCTCAGGAATGAGAATAC...-3'
CY048480  5'-...TGATTCCTGAAAGGAATGAACAGGGACAGAC---GAATGTGAGAGGCTCAGGAATGAGAATAC...-3'
FM174399  5'-...TGATTCCTGAAAGGAATGAACAAGGACAGAC---GAATGTGAGAGGCTCAGGAATGAGAATAC...-3'
CY030324  5'-...TGATACCTGAAAGGAATGAACAAGGGCAGAC---GAATGTGAGAGGCTCAGGAATGAGAATAC...-3'
CY030356  5'-...TGATACCTGAAAGAAATGAACAAGGGCAGAC---GAATGTGAGAGGCTCAGGAATGAGAATAC...-3'
CY048552  5'-...TGATTCCTGAAAGGAATGAACAGGGACAGAC---GAATGTGAGAGGCTCCGGAATGAGAATAC...-3'
HM172430  5'-...TGATTCCTGAAAGAAATGAACAAGGGCAGAC---GAATGTAAGAGGCTCAGGAATGAGAATAC...-3'
CY048128  5'-...TGATTCCTGAAAGGAATGAACAGGGACAGAC---GAATGTGAGAGGCTCAGGAATGAGAATAC...-3'
HM172428  5'-...TGATTCCTGAAAGGAATGAACAAGGGCAGAC---GAATGTGAGAGGCTCAGGAATGAGAATAC...-3'
DQ351871  5'-...TGGTTCCTGAAAGGAATGAACAAGGGCAAAC---GAATGTGAGAGGCTCAGGAATGAGAATAC...-3'
CY048104  5'-...TGATTCCTGAAAGGAATGAACAGGGACAAAC---GAATGTGAGAGGCTCAGGAATGAGAATAC...-3'
CY030244  5'-...TGATACCTGAAAGGAATGAACAAGGGCAGAC---GAATGTGAGAGGCTCAGGAATGAGAATAC...-3'
AB496988  5'-...TGATTCCTGAAAGGAATGAACAAGGACAGAC---GAATGTTAGAGGCTCAGGAATGAGAATAC...-3'
DQ320844  5'-...TGATTCCTGAAAGGAATGAACAAGGGCAGAC---GAATGTGAGAGGCTCAGGAATGAGAATAC...-3'
DQ992642  5'-...TGGTTCCTGAAAGGAATGAACAAGGGCAGAC---GAATGTGAGAGGCTCAGGAATGAGAATAC...-3'
HM172419  5'-...TGATTCCTGAAAGGAATGAACAGGGACAGAC---GAATGTGAAAGGCTCAGGAATGAAAATAC...-3'
GU356580  5'-...TGATTCCTGAAAGGAATGAACAGGGACAGAC---GAATGTGAGAGGCTCAGGAATGAGAATAC...-3'
AY221588  5'-...TGATTCCTGAAAGGAATGAACAAGGGCAGAC---GAATGTGAGAGGCTCAGGAATGAGAATAC...-3'
AB478015  5'-...TGATTCCTGAAAGGAATGAACAAGGGCAGAC---GAACGTGAGGGGCGCAGGAATGAGAATAC...-3'
CY029548  5'-...TGATTCCTGAAAGGAATGAACAAGGGCAGAC---GAATGTGAGAGGCTCAGGAATGAGAATAC...-3'
DQ999893  5'-...TAATTCCTGAAAGGAATGAACAAGGGCAGAC---GAATGTGAGAGGATCAGGAATGAGAATAC...-3'
CY040939  5'-...TGATTCCTGAAAGGAATGAACAAGGGCAGAC---GAATGTGAGAGGCTCAGGAATGAGAATAC...-3'
CY048496  5'-...TGATTCCTGAAAGGAATGAACAGGGACAGAC---GAATGTGAGAGGCTCAGGAATGAGAATAC...-3'
FM163437  5'-...TGATTCCTGAAAGGAATGAACAAGGACAGAC---GAATGTGAGAGGCTCAGGAATGAGAATAC...-3'
AY221589  5'-...TGATTCCTGAAAGGAATGAACAAGGACAAAC---GAATGTGAGAGGCTCAGGAATGAGAATAC...-3'
CY040955  5'-...TGATTCCTGAAAGGAATGAACAAGGGCAGAC---GAATGTGAGAGGCTCAGGAATGAGAATAC...-3'
CY090121  5'-...TGATTCCTGAAAGGAATGAACAAGGACAGAC---GAATGTGAGAGGCTCAGGAATGAGAATAC...-3'
CY030436  5'-...TGATACCTGAAAGGAATGAACAAGGGCAGAC---GAATGTGAGAGGCTCAGGAATGAGAATAC...-3'
FM163445  5'-...TGATTCCTGAAAGGAATGAACAAGGACAGAC---GAATGTGAGAGGCTCAGGAATGAGAATAC...-3'
AB496987  5'-...TGATTCCTGAAAGGAATGAACAAGGACAGAC---GAATGTTAGAGGCTCAGGAATGAGAATAC...-3'
CY029668  5'-...TGATTCCTGAAAGGAATGAACAAGGGCAGAC---GAATGTGAGAGGCTCAGGAATGAGAATAC...-3'
CY040947  5'-...TGATTCCTGAAAGGAATGAACAAGGGCAGAC---GAATGTGAGAGGCTCAGGAATGAGAATAC...-3'
CY030443  5'-...TGATACCTGAAAGGAATGAACAAGGGCAGAC---GAATGTGAGAGGCTCAGGAATGAGAATAC...-3'
CY030372  5'-...TGATACCTGAAAGGAATGAACAAGGGCAGAC---GAATGTGAGAGGCTCAGGAATGAGAATAC...-3'
CY029516  5'-...TGATTCCTGAAAGGAATGAACAAGGGCAGAC---GAATGTGAGAGGCTCAGGAATGAGAATAC...-3'
CY111110  5'-...TGATTCCTGAAAGGAATGAACAAGGACAGAC---GAATGTGAGAGGCTCAGGAATGAGAATAC...-3'
CY040979  5'-...TGATTCCTGAAAGGAATGAACAAGGGCAGAC---GAATGTGAGAGGCTCAGGAATGAGAATAC...-3'
AB496986  5'-...TGATTCCTGAAAGGAATGAACAAGGACAGAC---GAATGTTAGAGGCTCAGGAATGAGAATAC...-3'
DQ890066  5'-...TGATTCCTGAAAGGAATGAACAAGGACAGAC---GAATGTGAGAGGCTCAGGAATGAGAATAC...-3'
CY029652  5'-...TGATTCCTGAAAGGAATGAACAAGGGCAGAC---GAATGTGAGAGGCTCAGGAATGAGAATAC...-3'
GQ122436  5'-...TGATTCCTGAAAGGAATGAACAAGGGCAGAC---GAATGTGAGAGGCTCTGGAATGAGACTAC...-3'
GQ122454  5'-...TGATTCCTGAAAGGAATGAACAAGGGCAGAC---GAATGTGAGAGGCTCTGGAATGAGACTAC...-3'
EU590695  5'-...TGATTCCTGAAAGGAATGAACAGGGACAGAC---GAATGTGAGAGGATCAGGAATGAGAATAC...-3'
CY048512  5'-...TGATTCCTGAAAGGAATGAACAGGGACAGAC---GAATGTGAGAGGCTCCGGAATGAGAATAC...-3'
CY029228  5'-...TGATTCCTGAAAGGAATGAACAAGGGCAGAC---GAATGTGAGAGGCTCAGGAATGAGAATAC...-3'
CY048192  5'-...TGATTCCTGAAAGGAATGAACAGGGACAGAC---GAATGTGAGAGGCTCAGGAATGAGAATAC...-3'
CY029684  5'-...TGATCCCTGAAAGGAATGAACAAGGGCAGAC---GAATGTGAGAGGCTCAGGAATGAGGATAC...-3'
EU243140  5'-...TGGTTCCTGAAAGGAATGAACAAGGGCAAAC---GAATGTGAGAGGCTCAGGAATGAGAATAC...-3'
HM172427  5'-...TGATTCCTGAAAGGAATGAACAAGGGCAGAC---GAATGTGAGAGGCTCAGGAATGAGAATAC...-3'
JX420171  5'-...TGATTCCTGAAAGGAATGAACAAGGGCAGAC---GAATGTGAGAGGCTCAGGAATGAGAATAC...-3'
CY057200  5'-...TGATTCCTGAAAGGAATGAACAAGGGCAGAC---GAATGTTAGAGGCTCAGGAATGAGAATAC...-3'
JX420179  5'-...TGATCCCTGAAAGGAATGAACAAGGGCAGAC---GAATGTGAGAGGCTCAGGAATGAGGATAC...-3'
CY030498  5'-...TGATTCCTGAAAGGAATGAAAAAGGGCAGAC---GAATGTGAGAGGCTCAGGAATGAGAATAC...-3'
CY029628  5'-...TGATACCTGAAAGGAATGAACAAGGGCAGAC---GAATGTGAGAGGCTCAGGAATGAGAATAC...-3'
CY048408  5'-...TGATTCCTGAAAGGAATGAACAGGGACAGAC---GAATGTGAGAGGTTCAGGAATGAGAATAC...-3'
CY048592  5'-...TGATTCCTGAAAGGAATGAACAGGGACAGAC---GAATGTGAGAGGCTCAGGAATGAGAATAC...-3'
CY040995  5'-...TGATTCCTGAAAGGAATGAACAAGGACAGAC---GAATGTGAGAGGCTCAGGAATGAGAATAC...-3'
AB478016  5'-...TGATTCCTGAAAGGAATGAACAAGGACAGAC---GAATGTGAGAGGCTCAGGAATGAGAATAC...-3'
CY048144  5'-...TGATTCCTGAAAGGAATGAACAGGGACAAAC---GAATGTGAGAGGCTCAGGAATGAGAATAC...-3'
GQ122442  5'-...TGATTCCTGAAAGGAATGAACAAGGGCAGAC---GAATGTGAGAGGCTCTGGAATGAGAATAC...-3'
```

```
               224        235  240           254 1941           1956 1961    1969
                *          *    *             *   *              *    *       *
EU221256  5′-...TGATTCCTGAAAGGAATGAACAAGGGCAGAC---GAATGTGAGAGGCTCAGGAATGAGAATAC...-3′
HM172422  5′-...TGATTCCTGAAAGGAATGAACAAGGGCAGAC---GAATGTGAGAGGCTCAGGAATGAGAATAC...-3′
DQ095769  5′-...TGATTCCTGAAAGGAATGAACAAGGGCAGAC---GAATGTGAGAGGCTCAGGAATGAGAATAC...-3′
CY048152  5′-...TGATTCCTGAAAGGAATGAACAGGGACAGAC---GAATGTGAGAGGCTCCGGAATGAGAATAC...-3′
CY034685  5′-...TGATTCCTGAAAGGAATGAACAAGGGCAGAC---GAATGTGAGAGGCTCAGGAGTGAGAATAC...-3′
DQ992631  5′-...TGATTCCTGAAAGGAATGAACAAGGGCAGAC---GAATGTGAGAGGCTCAGGAATGAGAATAC...-3′
DQ992634  5′-...TGATTCCTGAAAGGAATGAACAAGGGCAGAC---GAATGTGAGAGGCTCAGGAATGAGAATAC...-3′
DQ992630  5′-...TGATTCCTGAAAGGAATGAACAAGGGCAGAC---GAATGTGAGAGGCTCAGGAATGAGAATAC...-3′
DQ992629  5′-...TGATTCCTGAAAGGAATGAACAAGGGCAGAC---GAATGTGAGAGGCTCAGGAATGAGAATAC...-3′
CY044047  5′-...TGATTCCTGAAAGGAATGAACAAGGACAGAC---GAATGTTAGAGGCTCAGGAATGAGAATAC...-3′
DQ890069  5′-...TGATTCCTGAAAGGAATGAACAAGGGCAGAC---GAATGTGAGAGGCTCAGGAATGAGAATAC...-3′
CY036178  5′-...TGATTCCTGAAAGGAATGAACAAGGGCAGAC---GAATGTGAGAGGCTCAGGAATGAGAATAC...-3′
CY048568  5′-...TGATTCCTGAAAGGAATGAACAGGGACAGAC---GAATGTGAGAGGCTCAGGAATGAGAATAC...-3′
DQ492829  5′-...TGATCCCTGAAAGGAATGAACAAGGGCAGAC---GAATGTGAGAGGCTCTGGAATGAGAATAC...-3′
CY048488  5′-...TGATTCCTGAAAGGAATGAACAGGGACAGAC---GAATGTGAGAGGCTCAGGAATGAGAATAC...-3′
CY080360  5′-...TGATTCCTGAAAGGAATGAACAAGGACAGAC---GAATGTGAGAGGCTCAGGAATGAGAATAC...-3′
DQ992599  5′-...TGATTCCTGAAAGGAATGAACAAGGGCAGAC---GAATGTGAGAGGCTCAGGAATGAGAATAC...-3′
CY030450  5′-...TGATTCCTGAAAGGAATGAACAAGGGCAGAC---GAATGTGAGAGGCTCAGGAGTGAGAATAC...-3′
CY030506  5′-...TGATTCCTGAAAGGAATGAAAAAGGGCAGAC---GAATGTGAGAGGCTCAGGAATGAGAATAC...-3′
DQ992627  5′-...TGATTCCTGAAAGGAATGAACAAGGGCAGAC---GAATGTGAGAGGCTCAGGAATGAGAATAC...-3′
DQ992628  5′-...TGATTCCTGAAAGGAATGAACAAGGGCAGAC---GAATGTGAGAGGCTCAGGAATGAGAATAC...-3′
CY034701  5′-...TGATTCCTGAAAGGAATGAACAAGGGCAGAC---GAATGTGAGAGGCTCAGGAATGAGAATAC...-3′
CY063478  5′-...TGATTCCTGAAAGGAATGAACAAGGACAGAC---GAATGTGAGAGGCTCAGGAATGAGAATAC...-3′
DQ520851  5′-...TGATTCCTGAAAGGAATGAACAAGGGCAGAC---GAATGTGAGAGGCTCAGGAATGAGAATAC...-3′
CY036170  5′-...TGATTCCTGAAAGGAATGAACAAGGGCAGAC---GAATGTGAGAGGCTCAGGAATGAGAATAC...-3′
CY048584  5′-...TGATTCCTGAAAGGAATGAACAGGGACAGAC---GAATGTGAGAGGCTCAGGAATGAGAATAC...-3′
CY030268  5′-...TGATCCCTGAAAGGAATGAACAAGGGCAGAC---GAATGTGAGAGGCTCAGGAATGAGAATAC...-3′
CY030292  5′-...TGATCCCTGAAAGGAATGAACAAGGGCAGAC---GAATGTGAGAGGCTCAGGAATGAGAATAC...-3′
CY044039  5′-...TGATTCCTGAAAGAAATGAACAAGGACAGAC---GAATGTTAGAGGCTCAGGAATGAGAATAC...-3′
CY034693  5′-...TGATTCCTGAAAGGAATGAACAAGGGCAGAC---GAATGTGAGAGGCTCAGGAATGAGAATAC...-3′
CY030252  5′-...TGATCCCTGAAAGGAATGAACAAGGGCAGAC---GAATGTGAGAGGCTCAGGAATGAGAATAC...-3′
CY040907  5′-...TGATTCCTGAAAGGAATGAACAAGGGCAGAC---GAATGTGAGAGGCTCAGGAATGAGAATAC...-3′
CY029724  5′-...TGATCCCTGAAAGGAATGAACAAGGGCAGAC---GAATGTGAGAGGCTCAGGAATGAGGATAC...-3′
CY030236  5′-...TGATTCCTGAAAGGAATGAACAAGGGCAGAC---GAATGTGAGAGGCTCAGGAATGAGAATAC...-3′
CY030284  5′-...TGATTCCTGAAAGGAATGAACAAGGGCAGAC---GAATGTGAGAGGCTCAGGAATGAGAATAC...-3′
GU083642  5′-...TGATTCCTGAAAGGAATGAACAAGGACAGAC---GAATGTGAGAGGCTCAGGAATGAGAATAC...-3′
DQ992688  5′-...TGATTCCTGAAAGGAATGAACAAGGGCAGAC---GAATGTGAGAGGCTCAGGAATGAGAATAC...-3′
CY036186  5′-...TGATTCCTGAAAGGAATGAACAAGGGCAGAC---GAATGTGAGAGGCTCAGGAATGAGAATAC...-3′
DQ992638  5′-...TGATTCCTGAAAGGAATGAACAAGGGCAGAC---GAATGTGAGAGGATCAGGAATGAGAATAC...-3′
CY057193  5′-...TGATTCCTGAAAGAAATGAACAAGGACAGAC---GAACGTTAGAGGCTCAGGAATGAGAATAC...-3′
JX420235  5′-...TGATCCCTGAAAGGAATGAACAAGGGCAGAC---GAATGTGAGAGGCTCAGGAATGAGAATAC...-3′
CY048624  5′-...TGATTCCTGAAAGGAATGAACAGGGACAGAC---GAATGTGAGAGGCTCAGGAATGAGAATAC...-3′
EU243132  5′-...TGGTTCCTGAAAGGAATGAACAAGGGCAAAC---GAATGTGAGAGGCTCAGGAATGAGAATAC...-3′
DQ992614  5′-...TGATTCCTGAAAGGAATGAACAAGGGCAGAC---GAATGTGAGAGGCTCAGGAATGAGAATAC...-3′
JX420147  5′-...TGATTCCTGAAAGGAATGAACAAGGGCAGAC---GAATGTGAGAGGCTCAGGAATGAGAATAC...-3′
HM172439  5′-...TGGTTCCTGAAAGGAATGAACAAGGGCAGAC---GAATGTGAGAGGCTCAGGAATGAGAATAC...-3′
JX420163  5′-...TGATTCCTGAAAGGAATGAAAAAGGGCAGAC---GAATGTGAGAGGCTCAGGAATGAGAATAC...-3′
CY030316  5′-...TGATCCCTGAAAGGAATGAACAAGGGCAGAC---GAATGTGAGAGGCTCAGGAATGAGAATAC...-3′
CY030332  5′-...TGATCCCTGAAAGGAATGAACAAGGGCAGAC---GAATGTGAGAGGCTCAGGAATGAGAATAC...-3′
DQ492818  5′-...TGATCCCTGAAAGGAATGAACAAGGGCAGAC---GAATGTGAGAGGCTCTGGAATGAGAATAC...-3′
CY029716  5′-...TGATCCCTGAAAGGAATGAACAAGGGCAGAC---AAATGTGAGAGGCTCAGGAATGAGGATAC...-3′
CY030412  5′-...TGATCCCTGAAAGGAATGAACAAGGGCAGAC---GAATGTGAGAGGCTCAGGAATGAGAATAC...-3′
CY030340  5′-...TGATCCCTGAAAGGAATGAACAAGGGCAGAC---GAATGTGAGAGGCTCAGGAATGAGAATAC...-3′
CY036266  5′-...TGATTCCTGAAAGGAATGAACAAGGGCAGAC---GAATGTGAGAGGCTCAGGAATGAGAATAC...-3′
CY029756  5′-...TGATCCCTGAAAGGAATGAACAAGGGCAGAC---GAATGTGAGAGGCTCAGGAATGAGGATAC...-3′
CY030260  5′-...TGATCCCTGAAAGGAATGAACAAGGGCAGAC---GAATGTGAGAGGCTCAGGAATGAGAATAC...-3′
CY029748  5′-...TGATCCCTGAAAGGAATGAACAAGGGCAGAC---GAATGTGAGAGGCTCAGGAATGAGGATAC...-3′
CY030388  5′-...TGATCCCTGAAAGGAATGAACAAGGGCAGAC---GAATGTGAGAGGCTCAGGAATGAGAATAC...-3′
HM172435  5′-...TGGTTCCTGAAAGGAATGAACAAGGGCAAAC---GAATGTGAGAGGCTCAGGAATGAGAATAC...-3′
CY029123  5′-...TGATTCCTGAAAGGAATGAACAAGGGCAGAC---GAATGTGAGAGGCTCAGGAATGAGAATAC...-3′
GU003873  5′-...TGATTCCTGAAAGGAATGAACAAGGGCAGAC---GAATGTGAGAGGCTCTGGAATGAGAATAC...-3′
CY029508  5′-...TGATTCCTGAAAGGAATGAACAAGGGCAGAC---GAATGTGAGAGGCTCAGGAATGAGAATAC...-3′
AY585506  5′-...TGATTCCTGAAAGGAATGAACAAGGGCAGAC---GAATGTGAGAGGCTCAGGAATGAGAATAC...-3′
AY576389  5′-...TGATTCCTGAAAGGAATGAACAAGGGCAGAC---GAATGTGAGAGGCTCAGGAATGAGAATAC...-3′
DQ992621  5′-...TGATTCCTGAAAGGAATGAACAAGGGCAGAC---GAATGTGAGAGGCTCAGGAATGAGAATAC...-3′
CY048112  5′-...TGATTCCTGAAAGGAATGAACAGGGACAGAC---GAATGTGAGAGGCTCCGGAATGAGAATAC...-3′
CY030420  5′-...TGATCCCTGAAAGGAATGAACAAGGGCAGAC---GAATGTGAGAGGCTCAGGAATGAGAATAC...-3′
KC261449  5′-...TGATTCCTGAAAGGAATGAACAAGGGCAGAC---GAATGTGAGAGGCTCAGGAATGAGAATAC...-3′
CY029700  5′-...TGATTCCTGAAAGGAATGAAAAAGGGCAGAC---GAATGTGAGAGGCTCAGGAATGAGAATAC...-3′
DQ992632  5′-...TGATTCCTGAAAGGAATGAACAAGGGCAGAC---GAATGTGAGAGGCTCAGGAATGAGAATAC...-3′
```

```
               224        235  240           254  1941          1956 1961    1969
                *          *    *             *   *              *    *       *
DQ992639  5′-...TGATTCCTGAAAGGAATGAACAAGGGCAGAC---GAATGTGAGAGGCTCAGGAATGAGAATAC...-3′
DQ992712  5′-...TGATTCCTGAAAGGAATGAACAAGGGCAGAC---GAATGTGAGAGGATCAGGAATGAGAATAC...-3′
CY030886  5′-...TGATTCCTGAAAGGAATGAACAAGGGCAGAC---GAATGTGAGAGGCTCAGGAATGAGAATAC...-3′
KF597841  5′-...TGATTCCTGAAAGGAATGAACAAGGACAGAC---GAATGTAAGAGGCTCAGGAATGAGAATAC...-3′
EU574926  5′-...TGATTCCTGAAAGAAATGAACAAGGACAGAC---GAATGTTAGAGGCTCAGGAATGAGAATAC...-3′
DQ992636  5′-...TGATTCCTGAAAGGAATGAACAAGGGCAGAC---GAATGTGAGAGGCTCAGGAATGAGAATAC...-3′
HM172481  5′-...TGATTCCTGAAAGGAATGAACAAGGACAGAC---GAATGTGAGAGGCTCAGGAATGAGAATAC...-3′
HM172408  5′-...TGATTCCTGAAAGGAATGAACAGGGGCAGAC---GAATGTGAGAGGCTCAGGAATGAGAATAC...-3′
GQ122448  5′-...TGATTCCTGAAAGGAATGAACAAGGGCAGAC---GAATGTGAGAGGCTCTGGAATGAGAATAC...-3′
GQ122460  5′-...TGATTCCTGAAAGGAATGAACAAGGGCAGAC---GAATGTGAGAGGCTCTGGAATGAGAATAC...-3′
DQ992640  5′-...TGATTCCTGAAAGGAATGAACAAGGGCAGAC---GAATGTGAGAGGATCAGGAATGAGAATAC...-3′
HM172421  5′-...TGATTCCTGAAAGAAATGAACAAGGGCAGAC---GAATGTGAGAGGCTCAGGAATGAGAATAC...-3′
JX420131  5′-...TGATTCCTGAAAGGAATGAACAAGGGCAGAC---GAATGTGAGAGGCTCAGGAATGAGAATAC...-3′
CY029556  5′-...TGATCCCTGAAAGGAATGAACAAGGGCAGAC---GAATGTGAGAGGCTCAGGAATGAGAATAC...-3′
CY030348  5′-...TGATCCCTGAAAGGAATGAACAAGGGCAGAC---GAATGTAAGAGGCTCAGGAATGAGAATAC...-3′
EU243139  5′-...TGGTTCCTGAAAGGAATGAACAAGGGCAAAC---GAATGTGAGAGGCTCAGGAATGAGAATAC...-3′
DQ992609  5′-...TGATTCCTGAAAGGAATGAACAAGGGCAGAC---GAATGTGAGAGGCTCAGGAATGAGAATAC...-3′
CY030308  5′-...TGATCCCTGAAAGGAATGAACAAGGGCAGAC---GAATGTGAGAGGCTCAGGAATGAGAATAC...-3′
CY030300  5′-...TGATCCCTGAAAGGAATGAACAAGGGCAGAC---GAATGTGAGAGGCTCAGGAATGAGAATAC...-3′
DQ992620  5′-...TGATTCCTGAAAGGAATGAACAAGGGCAGAC---GAATGTGAGAGGCTCAGGAATGAGAATAC...-3′
CY030276  5′-...TGATCCCTGAAAGGAATGAACAAGGGCAGAC---GAATGTGAGAGGCTCAGGAATGAGAATAC...-3′
DQ992685  5′-...TGATTCCTGAAAGGAATGAACAAGGGCAGAC---GAATGTGAGAGGCTCAGGAATGAGAATAC...-3′
AY221592  5′-...TGATTCCTGAAAGGAATGAACAAGGGCAGAC---GAATGTGAGAGGCTCAGGAATGAGAATAC...-3′
CY041297  5′-...TGATTCCTGAAAGAAATGAACAAGGACAGAC---GAATGTTAGAGGCTCAGGAATGAGAATAC...-3′
CY048616  5′-...TGATTCCTGAAAGGAATGAACAGGGACAGAC---GAATGTGAGAGGCTCCGGAATGAGAATAC...-3′
HM172436  5′-...TGGTTCCTGAAAGGAATGAACAAGGGCAGAC---GAATGTGAGAGGCTCAGGAATGAGAATAC...-3′
CY029660  5′-...TGATTCCTGAAAGGAATGAAAAAGGGCAGAC---GAATGTGAGAGGCTCAGGAATGAGAATAC...-3′
CY036234  5′-...TGATTCCTGAAAGGAATGAACAAGGGCAGAC---GAATGTGAGAGGCTCAGGAATGAGAATAC...-3′
CY034717  5′-...TGATTCCTGAAAGGAATGAGCAAGGGCAGAC---GAATGTGAGAGGCTCAGGAATGAGAATAC...-3′
CY030404  5′-...TGATCCCTGAAAGGAATGAACAAGGGCAGAC---GAATGTGAGAGGCTCAGGAATGAGAATAC...-3′
CY030958  5′-...TGATTCCTGAAAGGAATGAACAAGGGCAGAC---GAATGTGAGAGGCTCAGGAATGAGAATAC...-3′
CY030966  5′-...TGATTCCTGAAAGGAATGAACAAGGGCAGAC---GAATGTGAGAGGCTCAGGAATGAGAATAC...-3′
JX420227  5′-...TGATTCCTGAAAGGAATGAAAAAGGGCAGAC---GAATGTGAGAGGCTCAGGAATGAGAATAC...-3′
DQ992618  5′-...TGATTCCTGAAAGGAATGAACAAGGGCAGAC---GAATGTGAGAGGCTCAGGAATGAGAATAC...-3′
DQ992619  5′-...TGATTCCTGAAAGGAATGAACAAGGGCAGAC---GAATGTGAGAGGCTCAGGAATGAGAATAC...-3′
JX420123  5′-...TGATTCCTGAAAGGAATGAACAAGGGCAGAC---GAATGTGAGAGGCTCAGGAGTGAGAATAC...-3′
GQ917233  5′-...TGATTCCTGAAAGGAATGAACAGGGACAGAC---GAATGTAAGAGGCTCAGGAATGAGAATAC...-3′
CY030428  5′-...TGATHCCTGAAAGGAATGAACAAGGGCAGAC---GAATGTGAGAGGCTCAGGAATGAGAATAC...-3′
DQ992624  5′-...TGATTCCTGAAAGGAATGAACAAGGGCAGAC---GAATGTGAGAGGCTCAGGAATGAGAATAC...-3′
DQ992686  5′-...TGATTCCTGAAAGGAATGAACAAGGGCAGAC---GAATGTGAGAGGCTCAGGAATGAGAATAC...-3′
DQ320831  5′-...TGATTCCTGAAAGGAACGAACAAGGGCAGAA---GAATGTGAGAGGCTCTGGAATGAGAATAC...-3′
CY036202  5′-...TGATTCCTGAAAGGAATGAACAAGGGCAGAC---GAATGTGAGAGGCTCAGGAATGAGAATAC...-3′
CY030942  5′-...TGATTCCTGAAAGGAATGAACAAGGGCAGAC---GAATGTGAGAGGCTCAGGAATGAGAATAC...-3′
CY030950  5′-...TGATTCCTGAAAGGAATGAACAAGGGCAGAC---GAATGTGAGAGGCTCAGGAATGAGAATAC...-3′
EU195405  5′-...TGGTTCCTGAAAGGAATGAACAAGGGCAAAC---GAATGTGAGAGGCTCAGGAATGAGAATAC...-3′
HM172415  5′-...TGATTCCTGAAAGGAATGAACAGGGACAGAC---GAATGTGAGAGGCTCAGGAATGAGAATAC...-3′
DQ992617  5′-...TGATTCCAGAAAGGAATGAACAAGGGCAGAC---GAATGTGAGAGGCTCAGGAATGAGAATAC...-3′
CY029588  5′-...TGATCCCTGAAAGGAATGAACAAGGGCAGAC---GAATGTGAGAGGCTCAGGAATGAGAATAC...-3′
DQ992575  5′-...TGATTCCTGAAAGGAATGAACAAGGGCAGAC---GAACGTGAGAGGCTCAGGAATGAGAATAC...-3′
DQ992613  5′-...TGATTCCTGAAAGGAATGAACAAGGGCAGAC---GAATGTGAGAGGCTCAGGAATGAGAATAC...-3′
CY036210  5′-...TGATTCCTGAAAGGAATGAACAAGGGCAGAC---GAATGTGAGAGGCTCAGGAATGAGAATAC...-3′
CY029636  5′-...TGATCCCTGAAAGGAATGAACAAGGGCAGAC---GAATGTGAGAGGCTCAGGAATGAGAATAC...-3′
CY029644  5′-...TGATCCCTGAAAGGAATGAACAAGGGCAGAC---GAATGTGAGAGGCTCAGGAATGAGAATAC...-3′
CY030982  5′-...TGATTCCTGAAAGGAATGAACAAGGGCAGAC---GAATGTGAGAGGCTCAGGAATGAGAATAC...-3′
DQ992610  5′-...TGATTCCTGAAAGGAATGAACAAGGGCAGAC---GAATGTGAGAGGCTCAGGAATGAGAATAC...-3′
CY029572  5′-...TGATCCCTGAAAGGAATGAACAAGGGCAGAC---GAATGTGAGAGGCTCAGGAATGAGAATAC...-3′
CY040931  5′-...TGATTCCTGAAAGGAATGAGCAAGGGCAGAC---GAATGTGAGAGGCTCAGGAATGAGAATAC...-3′
CY036250  5′-...TGATTCCTGAAAGGAATGAACAAGGGCAGAC---GAATGTGAGAGGCTCAGGAATGAGAATAC...-3′
CY029596  5′-...TGATCCCTGAAAGGAATGAACAAGGGCAGAC---GAATGTGAGAGGCTCAGGAATGAGAATAC...-3′
AY585523  5′-...TGATTCCTGAAAGGAATGAACAAGGGCAAAC---GAATGTGAGAGGCTCAGGAATGAGAATAC...-3′
CY029604  5′-...TGATCCCTGAAAGGAATGAACAAGGGCAGAC---GAATGTGAGAGGCTCAGGAATGAGAATAC...-3′
DQ492828  5′-...TGATTCCTGAAAGGAATGAACAAGGGCAGAC---GAATGTGAGAGGCTCTGGAATGAGAATAC...-3′
CY048184  5′-...TGATTCCTGAAAGGAATGAACAGGGACAGAC---GAATGTGAGAGGCTCCGGAATGAGAATAC...-3′
CY029018  5′-...TGATTCCTGAAAGGAATGAACAAGGGCAGAC---GAATGTGAGAGGCTCAGGAATGAGAATAC...-3′
EU195397  5′-...TGATTCCTGAAAGGAATGAACAAGGGCAGAC---GAATGTGAGAGGCTCAGGAATGAGAATAC...-3′
CY036258  5′-...TGATTCCTGAAAGGAATGAACAAGGGCAAAC---GAATGTGAGAGGCTCAGGAATGAGAATAC...-3′
CY029580  5′-...TGATCCCTGAAAGGAATGAACAAGGGCAGAC---GAATGTGAGAGGCTCAGGAATGAGAATAC...-3′
HM172443  5′-...TGGTTCCTGAAAGGAATGAACAAGGGCAAAC---GAATGTGAGAGGCTCAGGAATGAGAATAC...-3′
GQ122552  5′-...TGATTCCTGAAAGGAATGAACAAGGGCAGAC---GAATGTGAGAGGCTCTGGAATGAGAATAC...-3′
KF735629  5′-...TGATTCCTGAAAGGAATGAACAAGGGCAGAC---GAATGTGAGAGGCTCAGGAATGAGAATAC...-3′
```

```
                         224          235  240            254  1941             1956  1961     1969
                          *            *    *              *    *                *     *        *
DQ992677  5'-...TGATTCCTGAAAGGAATGAACAGGGGCAGAC---GAATGTGAGAGGCTCAGGAATGAGAATAC...-3'
HM172426  5'-...TGATTCCTGAAAGGAATGAACAAGGACAGAC---GAATGTTAGAGGCTCAGGAATGAGAATAC...-3'
CY029732  5'-...TGATCCCTGAAAGGAATGAACAAGGGCAGAC---GAATGTGAGAGGCTCAGGAATGAGGATAC...-3'
AY585511  5'-...TGATTCCTGAAAGGAATGAACGAGGGCAGAC---GAATGTGAGAGGCTCAGGAATGAGAATAC...-3'
CY048176  5'-...TGATTCCTGAAAGGAATGAACAGGGACAGAC---GAATGTGAGAGGCTCCGGAATGAGAATAC...-3'
DQ992678  5'-...TGATTCCTGAAAGGAATGAACAGGGGCAGAC---GAATGTGAGAGGCTCAGGAATGAGAATAC...-3'
DQ992700  5'-...TGATTCCTGAAAGGAATGAACAGGGGCAGAC---GAATGTGAGAGGCTCAGGAATGAGAATAC...-3'
HM172416  5'-...TGATTCCTGAAAGGAATGAACAAGGGCAAAC---GAATGTGAGAGGCTCTGGAATGAGAATAC...-3'
GQ122508  5'-...TGATTCCTGAAAGGAATGAACAAGGGCAGAC---GAATGTGAGAGGCTCTGGAATGAGAATAC...-3'
GQ122466  5'-...TGATTCCTGAAAGGAATGAACAAGGGCAGAC---GAATGTGAGAGGCTCTGGAATGAGAATAC...-3'
GQ122472  5'-...TGATTCCTGAAAGGAATGAACAAGGGCAGAC---GAATGTGAGAGGCTCTGGAATGAGAATAC...-3'
DQ992574  5'-...TGATTCCTGAAAGGAATGAACAAGGGCAGAC---GAATGTGAGAGGCTCAGGAATGAGAATAC...-3'
DQ992680  5'-...TGATTCCTGAAAGGAATGAACAGGGGCAGAC---GAATGTGAGAGGCTCAGGAATGAGAATAC...-3'
GQ122484  5'-...TGATTCCTGAAAGGAATGAACAAGGGCAGAC---GAATGTGAGAGGCTCTGGAATGAGAATAC...-3'
GQ122490  5'-...TGATTCCTGAAAGGAATGAACAAGGGCAGAC---GAATGTGAGAGGCTCTGGAATGAGAATAC...-3'
GQ122478  5'-...TGATTCCTGAAAGGAATGAACAAGGGCAGAC---GAATGTGAGAGGCTCTGGAATGAGAATAC...-3'
HM172431  5'-...TGGTTCCTGAAAGGAATGAACAAGGGCAAAC---GAATGTGAGAGGCTCAGGAATGAGAATAC...-3'
CY029620  5'-...TGATCCCTGAAAGGAATGAACAAGGGCAGAC---GAATGTAAGAGGCTCAGGAATGAGAATAC...-3'
DQ320869  5'-...TGATTCCTGAAAGGAATGAACAAGGGCAGAC---GAATGTGAGAGGCTCTGGAATGAGAATAC...-3'
CY041027  5'-...TGATTCCTGAAAGGAATGAACAAGGGCAGAC---GAATGTGAGAGGCTCAGGGATGAGAATAC...-3'
JX420211  5'-...TGATCCCTGAAAGGAATGAACAAGGGCAGAC---GAATGTGAGAGGCTCAGGAATGAGAATAC...-3'
HM172434  5'-...TGGTTCCTGAAAGGAATGAACAAGGGCAAAC---GAATGTGAAAGGCTCAGGAATGAGAATAC...-3'
DQ992635  5'-...TGATTCCTGAAAGGAATGAACAAGGGCAGAC---GAATATGAGAGGCTCAGGAATGAGAATAC...-3'
DQ492826  5'-...TGATTCCTGAAAGGAATGAACAAGGGCAGAC---GAATGTGAGAGGCTCTGGAATGAGAATAC...-3'
CY036242  5'-...TGATTCCTGAAAGGAATGAACAAGGGCAGAC---GAATGTGAGAGGCTCAGGAATGAGAATAC...-3'
CY029564  5'-...TGATCCCTGAAAGGAATGAACAAGGGCAGAC---GAATGTGAGAGGCTCAGGAATGAGAATAC...-3'
DQ320867  5'-...TGATTCCTGAAAGGAATGAACAAGGGCAGAC---GAATGTGAGAGGCTCTGGAATGAGAATAC...-3'
CY061291  5'-...TGATTCCTGAAAGGAATGAACAAGGACAGAC---GAATGTGAGAGGCTCAGGAATGAGAATAC...-3'
CY090113  5'-...TGATTCCTGAAAGGAATGAACAAGGACAGAC---GAATGTGAGAGGCTCAGGAATGAGAATAC...-3'
JQ936700  5'-...TGATTCCAGAAAGGAATGAACAAGGACAGAC---GAATGTGAGAGGCTCAGGAATGAGAATAC...-3'
DQ492819  5'-...TGATTCCTGAAAGGAATGAACAAGGGCAGAC---GAATGTGAGAGGCTCTGGAATGAGAATAC...-3'
GU182147  5'-...TGATTCCTGAAAGGAATGAACAAGGGCAGAC---GAATGTAAGAGGCTCAGGAATGAGAATAC...-3'
DQ992676  5'-...TGATTCCTGAAAGGAATGAACAGGGGCAGAC---GAATGTGAGAGGCTCAGGAATGAGAATAC...-3'
CY029025  5'-...TGATTCCTGAAAGGAATGAACAAGGGCAGAC---GAATGTGAGAGGCTCAGGAATGAGAATAC...-3'
CY041019  5'-...TGATTCCTGAAAGGAATGAACAAGGGCAGAC---GAATGTGAGAGGCTCAGGGATGAGAATAC...-3'
CY126017  5'-...TGATTCCTGAAAGAAATGAACAAGGACAGAC---GAATGTTAGAGGCTCAGGGATGAGAATAC...-3'
CY048160  5'-...TGATTCCTGAAAAGAATGAACAGGGACAGAC---GAATGTGAGAGGCTCCGGAATGAGAATAC...-3'
HM172433  5'-...TGGTCCCTGAAAGGAATGAACAAGGGCAAAC---GAATGTGAGAGGCTCAGGAATGAGAATAC...-3'
DQ492842  5'-...TGATTCCTGAAAGGAATGAACAAGGGCAGAC---GAATGTGAGAGGCTCTGGAATGAGAATAC...-3'
CY041305  5'-...TGATTCCTGAAAGGAATGAACAAGGGCAGAC---GAATGTTAGAGGCTCAGGAATGAGAATAC...-3'
DQ992675  5'-...TGATTCCTGAAAGGAATGAACAGGGGCAGAC---GAATGTGAGAGGCTCAGGAATGAGAATAC...-3'
JX420219  5'-...TGATCCCTGAAAGGAATGAACAAGGGCAGAC---GAATGTGAGAGGCTCAGGAATGAGAATAC...-3'
CY036194  5'-...TGATTCCTGAAAGGAATGAACAAGGGCAGAC---GAATGTGAGAGGCTCAGGAATGAGAATAC...-3'
DQ492834  5'-...TGATTCCTGAAAGGAATGAACAAGGGCAGAC---GAATGTGAGAGGCTCTGGAATGAGAATAC...-3'
HM172407  5'-...TGATTCCTGAAAGGAATGAACAAGGGCAGAC---GAATGTGAGAGGCTCAGGAATGAGGATAA...-3'
FJ784827  5'-...TGATTCCTGAAAGGAATGAACAAGGGCAGAC---GAATGTGAGAGGCTCAGGAATGAGAATAC...-3'
CY041011  5'-...TGATTCCTGAAAGGAATGAACAAGGGCAGAC---GAATGTGAGAGGCTCAGGGATGAGAATAC...-3'
CY041003  5'-...TGATTCCTGAAAGGAATGAACAAGGGCAGAC---GAATGTGAGAGGCTCAGGGATGAGAATAC...-3'
AB499529  5'-...TGATTCCTGAAAGGAATGAACAAGGGCAGAC---GAATGTGAGAGGCTCAGGGATGAGAATAC...-3'
FR687273  5'-...TGATTCCTGAAAGAAATGAACAAGGACAGAC---GAATGTTAGAGGCTCAGGAATGAGAATAC...-3'
HM172420  5'-...TGGTTCCTGAAAGGAATGAACAAGGGCAAAC---GAATGTGAGAGGCTCAGGAATGAGAATAC...-3'
JQ936684  5'-...TGATTCCAGAAAGGAATGAACAAGGACAGAC---GAATGTGAGAGGCTCAGGAATGAGAATAC...-3'
KC261448  5'-...TGATTCCCGAAAGGAATGAACAAGGGCAGAC---GAATGTAAGAGGCTCAGGAATGAGAATAC...-3'
DQ492827  5'-...TGATTCCTGAAAGGAATGAACAAGGGCAGAC---GAATGTGAGAGGCTCTGGAATGAGAATAC...-3'
CY030520  5'-...TGATACCTGAAAGGAATGAACAAGGGCAGAC---GAATGTGAGAGGCTCAGGAATGAGAATAC...-3'
DQ492825  5'-...TGATTCCTGAAAGGAATGAACAAGGGCAGAC---GAATGTGAGAGGCTCTGGAATGAGAATAC...-3'
DQ492840  5'-...TGATTCCTGAAAGGAATGAACAAGGGCAGAC---GAATGTGAGAGGCTCTGGAATGAGAATAC...-3'
DQ320866  5'-...TGATTCCTGAAAGGAATGAACAAGGGCAGAC---GAATGTGAGAGGCTCTGGAATGAGAATAC...-3'
FR687274  5'-...TGATTCCTGAAAGAAATGAACAAGGACAGAC---GAATGTTAGAGGCTCAGGAATGAGAATAC...-3'
CY040987  5'-...TGATTCCTGAAAGGAATGAACAAGGGCAGAC---GAATGTGAGAGGCTCAGGGATGAGAATAC...-3'
FJ784829  5'-...TGATTCCTGAAAGGAATGAACAAGGGCAGAC---GAATGTGAGAGGCTCAGGAATGAGAATAC...-3'
GQ412036  5'-...TGATTCCTGAAAGGAATGAACAAGGGCAGAC---GAATGTAAGAGGCTCAGGAATGAGAATAC...-3'
GQ412035  5'-...TGATTCCTGAAAGGAATGAACAAGGGCAGAC---GAATGTAAGAGGCTCAGGAATGAGAATAC...-3'
KJ028471  5'-...TGATTCCTGAAAGGAATGAACAAGGGCAGAC---GAATGTGAGAGGCTCAGGAATGAGAATAC...-3'
CY036226  5'-...TGATTCCTGAAAGGAATGAACAAGGGCAGAC---GAATGTGAGAGGCTCAGGAATGAGAATAC...-3'
GQ412037  5'-...TGATTCCTGAAAGGAATGAACAAGGGCAGAC---GAATGTAAGAGGCTCAGGAATGAGAATAC...-3'
CY036218  5'-...TGATTCCTGAAAGGAATGAACAAGGGCAGAC---GAATGTGAGAGGCTCAGGAATGAGAATAC...-3'
GQ412033  5'-...TGATTCCTGAAAGGAATGAACAAGGGCAGAC---GAATGTAAGAGGCTCAGGAATGAGAATAC...-3'
HM172424  5'-...TGATTCCTGAAAGGAATGAACAAGGACAGAC---GAATGTTAGAGGCTCAGGAATGAGAATAC...-3'
DQ492843  5'-...TGATTCCTGAAAGGAATGAACAAGGGCAAAC---GAATGTGAGAGGCTCTGGAATGAGAATAC...-3'
```

```
                  224         235  240           254  1941          1956  1961    1969
                   *           *    *             *    *             *     *       *
DQ492844  5'-...TGATTCCTGAAAGGAATGAACAAGGGCAGAC---GAATGTGAGAGGCTCTGGAATGAGAATAC...-3'
GU477556  5'-...TGATTCCTGAAAGGAATGAACAAGGGCAGAC---GAATGTGAGAGGCTCAGGAATGAGAATAC...-3'
HM172440  5'-...TGGTTCCTGAAAGGAATGAACAAGGGCAAAC---GAATGTGAGAGGCTCAGGAATGAAAATAC...-3'
AB436731  5'-...TGATTCCTGAAAGGAATGAACAAGGGCAGAC---GAATGTAAGAGGCTCAGGAATGAGAATAC...-3'
GQ412038  5'-...TGATTCCTGAAAGGAATGAACAAGGGCAGAC---GAATGTAAGAGGCTCAGGAATGAGAATAC...-3'
DQ492824  5'-...TGATTCCTGAAAGGAATGAACAAGGGCAGAC---GAATGTGAGAGGCTCTGGAATGAGAATAC...-3'
CY126223  5'-...TGATTCCTGAAAGAAATGAACAAGGACAGAC---GAATGTTAGAGGCTCAGGAATGAGAATAC...-3'
DQ492841  5'-...TGATTCCTGAAAGGAATGAACAAGGGCAGAC---GAATGTGAGAGGCTCTGGAATGAGAATAC...-3'
HM172418  5'-...TGGTTCCTGAAAGGAATGAACAAGGGCAAAC---GAATGTGAGAGGCTCAGGAATGAGAATAC...-3'
HM172410  5'-...TGATTCCTGAAAGGAATGAACAAGGGCAGAC---GAATGTGAGAGGCTCAGGAATGAGAATAC...-3'
GU477558  5'-...TGATTCCTGAAAGGAATGAACAAGGGCAGAC---GAATGTGAGAGGCTCAGGAATGAGAATAC...-3'
JF975558  5'-...TGATTCCTGAAAGGAATGAACAAGGGCAGAC---GAATGTGAGAGGCTCAGGAATGAGAATAC...-3'
GQ122544  5'-...TGATTCCTGAAAGGAATGAACAAGGGCAGAC---GAATGTGAGAGGCTCTGGAATGAGAATAC...-3'
GQ122560  5'-...TGATTCCTGAAAGGAATGAACAAGGGCAGAC---GAATGTGAGAGGCTCTGGAATGAGAATAC...-3'
GQ122514  5'-...TGATTCCTGAAAGGAATGAACAAGGGCAGAC---GAATGTGAGAGGCTCTGGAATGAGAATAC...-3'
CY014182  5'-...TGATTCCTGAAAGGAATGAACAAGGGCAGAC---GAATGTGAGAGGCTCTGGAATGAGAATAC...-3'
HM172444  5'-...TGGTTCCTGAAAGGAATGAACAAGGGCAAAC---GAATGTGAGAGGCTCAGGAATGAGAATAC...-3'
GQ412034  5'-...TGATTCCTGAAAGGAATGAACAAGGGCAGAC---GAATGTAAGAGGCTCAGGAATGAGAATAC...-3'
HM172417  5'-...TGGTTCCTGAGAGGAATGAACAAGGGCAAAC---GAATGTGAGGGGCTCAGGAATGAGAATAC...-3'
GQ122520  5'-...TGATTCCTGAAAGGAATGAACAAGGGCAGAC---GAATGTGAGAGGCTCTGGAATGAGAATAC...-3'
CY014190  5'-...TGATTCCTGAAAGGAATGAACAAGGGCAGAC---GAATGTGAGAGGCTCTGGAATGAGAATAC...-3'
GQ122526  5'-...TGATTCCTGAAAGGAATGAACAAGGGCAGAC---GAATGTGAGAGGCTCTGGAATGAGAATAC...-3'
GQ122496  5'-...TGATTCCTGAAAGGAATGAACAAGGGCAGAC---GAATGTGAGAGGCTCTGGAATGAGAATAC...-3'
GQ122502  5'-...TGATTCCTGAAAGGAATGAACAAGGGCAGAC---GAATGTGAGAGGCTCTGGAATGAGAATAC...-3'
CY110851  5'-...TGATTCCTGAAAGGAATGAACAAGGGCAGAC---GAATGTGAGAGGCTCAGGAATGAGAATAC...-3'
GQ122538  5'-...TGATTCCTGAAAGGAATGAACAAGGGCAGAC---GAATGTGAGAGGCTCTGGAATGAGAATAC...-3'
FJ750826  5'-...TGATTCCTGAAAGGAATGAAAAAGGGCAGAC---GAATGTGAGAGGATCAGGAATGAGAATAC...-3'
GU477557  5'-...TGATTCCTGAAAGGAATGAACAAGGGCAGAC---GAATGTGAGAGGCTCAGGAATGAGAATAC...-3'
EU850413  5'-...TGATTCCTGAAAGGAATGAACAAGGGCAGAC---GAATGTGAGAGGATCAGGAATGAGAATAC...-3'
GQ122532  5'-...TGATTCCTGAAAGGAATGAACAAGGGCAGAC---GAATGTGAGAGGCTCTGGAATGAGAATAC...-3'
GQ122568  5'-...TGATTCCTGAAAGGAATGAACAAGGGCAGAC---GAATGTGAGAGGCTCTGGAATGAGAATAC...-3'
JQ638688  5'-...TGATTCCTGAAAGGAATGAACAAGGGCAGAT---GAATGTGAGAGGCTCAGGAATGAGAATAC...-3'
FJ750819  5'-...TGATTCCTGAAAGGAATGAACAAGGGCAGAC---GAATGTGAGAGGATCAGGAATGAGAATAC...-3'
HM172411  5'-...TGGTTCCTGAGAGGAATGAACAAGGGCAAAC---GAATGTGAGAGGCTCAGGAATGAGAATAC...-3'
JQ638687  5'-...TGATTCCTGAAAGGAATGAACAAGGGCAGAC---GAATGTGAGAGGCTCAGGAATGAGAATAC...-3'
JX523356  5'-...TGGTTCCTGAGAGGAATGAACAAGGGCAAAC---GAATGTGAGGGGCTCAGGAATGAGAATAC...-3'
KF735635  5'-...TGATTCCTGAGAGGAATGAACAGGGGCAGAC---GAATGTGAGGGGCTCAGGAATGAGAATAC...-3'
AB684133  5'-...TGATTCCTGAAAGGAATGAACAAGGGCAGAC---GAATGTGAGAGGCTCAGGAATGAGAATAC...-3'
AB684116  5'-...TGATTCCAGAAAGGAATGAACAAGGGCAGAC---GAATGTGAGAGGCTCAGGAATGAGAATAC...-3'
JN807918  5'-...TGATTCCTGAAAGGAATGAACAAGGGCAGAC---GAATGTGAGAGGCTCAGGAATGAGAATAC...-3'
AB684125  5'-...TGATTCCTGAAAGGAATGAACAAGGGCAGAC---GAATGTGAGAGGCTCAGGAATGAGAATAC...-3'
JN202573  5'-...TGATTCCTGAAAGGAATGAACAAGGGCAGAC---GAATGTGAGAGGCTCAGGAATGAGAATAC...-3'
AB684183  5'-...TGATTCCTGAAAGGAATGAACAAGGGCAGAC---GAATGTGAGAGGCTCAGGAATGAGAATAC...-3'
KC261452  5'-...TGATTCCTGAAAGAAATGAACAAGGGCAGAC---GAATGTGAGAGGCTCAGGAATGAGAATAC...-3'
AB684205  5'-...TGATTCCTGAAAGGAATGAACAAGGGCAGAC---GAATGTGAGAGGCTCAGGAATGAGAATAC...-3'
AB684233  5'-...TGATTCCTGAAAGGAATGAACAAGGGCAGAC---GAATGTGAGAGGCTCAGGAATGAGAATAC...-3'
JN807903  5'-...TGATTCCTGAAAGGAATGAACAAGGGCAGAC---GAATGTGAGAGGCTCAGGAATGAGAATAC...-3'
KF735641  5'-...TGATTCCTGAAAGGAATGAACAAGGGCAGAC---GAATGTGAGGGGATCAGGAATGAGAATAC...-3'
JN807900  5'-...TGATTCCTGAAAGGAATGAACAAGGGCAGAC---GAATGTGAGAGGCTCAGGAATGAGAATAC...-3'
JN807901  5'-...TGATTCCTGAAAGGAATGAACAAGGGCAGAC---GAATGTGAGAGGCTCAGGAATGAGAATAC...-3'
AB684226  5'-...TGATTCCTGAAAGGAATGAACAAGGGCAGAC---GAATGTGAGAGGCTCAGGAATGAGAATAC...-3'
AB684102  5'-...TGATTCCTGAAAGGAATGAACAAGGGCAGAC---GAATGTGAGAGGCTCAGGAATGAGAATAC...-3'
KC261451  5'-...TGATTCCTGAAAGAAATGAACAAGGGCAGAC---GAATGTGAGAGGCTCAGGAATGAGAATAC...-3'
DQ992690  5'-...TGATCCCTGAACGGAATGAACAGGGGCAGAC---GAATGTGAGAGGCTCAGGAATGAGAATAC...-3'
CY146689  5'-...TGATTCCTGAAAGGAATGAACAAGGGCAGAC---GAATGTGAGAGGCTCAGGAATGAGAATAC...-3'
AB684169  5'-...TGATTCCTGAAAGGAATGAACAAGGGCAGAC---GAATGTGAGAGGCTCAGGAATGAGAATAC...-3'
JN807902  5'-...TGATTCCTGAAAGGAATGAACAAGGGCAGAC---GAATGTGAGAGGCTCAGGAATGAGAATAC...-3'
JN807911  5'-...TGATTCCTGAAAGGAATGAACAAGGGCAGAC---GAATGTGAGAGGCTCAGGAATGAGAATAC...-3'
JN807904  5'-...TGATTCCTGAAAGGAATGAACAAGGGCAGAC---GAATGTGAGAGGCTCAGGAATGAGAATAC...-3'
AB684219  5'-...TGATTCCTGAAAGGAATGAACAAGGGCAGAC---GAATGTGAGAGGCTCAGGAATGAGAATAC...-3'
AB684095  5'-...TGATTCCTGAAAGGAATGAACAAGGGCAGAC---GAATGTGAGAGGCTCAGGAATGAGAATAC...-3'
JN202572  5'-...TGATTCCTGAAAGGAATGAACAAGGGCAGAC---GAATGTGAGAGGCTCAGGAATGAGAATAC...-3'
AB684088  5'-...TGATTCCAGAAAGGAATGAACAAGGGCAGAC---GAATGTGAGAGGCTCAGGAATGAGAATAC...-3'
JN807912  5'-...TGATTCCTGAAAGGAATGAACAAGGGCAGAC---GAATGTGAGAGGCTCAGGAATGAGAATAC...-3'
HQ695912  5'-...TGATTCCTGAAAGGAATGAACAAGGGCAGAC---GAATGTGAGAGGCTCAGGAATGAGAATAC...-3'
AB684081  5'-...TGATTCCAGAAAGGAATGAACAAGGGCAGAC---GAATGTGAGAGGCTCAGGAATGAGAATAC...-3'
JN807905  5'-...TGATTCCTGAAAGGAATGAACAAGGGCAGAC---GAATGTGAGAGGCTCAGGAATGAGAATAC...-3'
JN807915  5'-...TGATTCCTGAAAGGAATGAACAAGGGCAGAC---GAATGTGAGAGGCTCAGGAATGAGAATAC...-3'
AB684190  5'-...TGATTCCTGAAAGGAATGAACAAGGGCAGAC---GAATGTGAGAGGCTCAGGAATGAGAATAC...-3'
AB684140  5'-...TGATTCCTGAAAGGAATGAACAAGGGCAGAC---GAATGTGAGAGGCTCAGGAATGAGAATAC...-3'
```

```
               224        235  240           254 1941           1956 1961    1969
                *          *    *             *   *              *    *       *
AB684197  5’-...TGATTCCTGAAAGGAATGAACAAGGGCAGAC---GAATGTGAGAGGCTCAGGAATGAGAATAC...-3’
AB684109  5’-...TGATTCCTGAAAGGAATGAACAAGGGCAGAC---GAATGTGAGAGGCTCAGGAATGAGAATAC...-3’
JN807910  5’-...TGATTCCTGAAAGGAATGAACAAGGGCAGAC---GAATGTGAGAGGCTCAGGAATGAGAATAC...-3’
AB684147  5’-...TGATTCCTGAAAGGAATGAACAAGGGCAGAC---GAATGTGAGAGGCTCAGGAATGAGAATAC...-3’
JN807893  5’-...TGATTCCTGAAAGGAATGAACAAGGGCAGAC---GAATGTGAGAGGCTCAGGAATGAGAATAC...-3’
AB684212  5’-...TGATTCCTGAAAGGAATGAACAAGGGCAGAC---GAATGTGAGAGGATCAGGAATGAGAATAC...-3’
JN807894  5’-...TGATTCCTGAAAGGAATGAACAAGGGCAGAC---GAATGTGAGAGGCTCAGGAATGAGAATAC...-3’
JN807917  5’-...TGATTCCTGAAAGGAATGAACAAGGGCAGAC---GAATGTGAGAGGCTCAGGAATGAGAATAC...-3’
HM172409  5’-...TGGTTCCTGAAAGGAATGAACAAGGGCAAAC---GAATGTGAGAGGCTCAGGAATGAGAATAC...-3’
AB684154  5’-...TGATTCCTGAAAGGAATGAACAAGGGCAGAC---GAATGTGAGAGGCTCAGGAATGAGAATAC...-3’
JN807913  5’-...TGATTCCTGAAAGGAATGAACAAGGGCAGAC---GAATGTGAGAGGCTCAGGAATGAGAATAC...-3’
JN807916  5’-...TGATTCCTGAAAGGAATGAACAAGGGCAGAC---GAATGTGAGAGGCTCAGGAATGAGAATAC...-3’
JN807914  5’-...TGATTCCTGAAAGGAATGAACAAGGGCAGAC---GAATGTGAGAGGCTCAGGAATGAGAATAC...-3’
JN807908  5’-...TGATTCCTGAAAGGAATGAACAAGGGCAGAC---GAATGTGAGAGGCTCAGGAATGAGAATAC...-3’
JN807899  5’-...TGATTCCTGAAAGGAATGAACAAGGGCAGAC---GAATGTGAGAGGCTCAGGAATGAGAATAC...-3’
CY146705  5’-...TGATTCCTGAAAGGAATGAACAAGGGCAGAC---GAATGTGAGGGGCTCAGGAATGAGAATAC...-3’
JN807907  5’-...TGATTCCTGAAAGGAATGAACAAGGGCAGAC---GAATGTGAGAGGCTCAGGAATGAGAATAC...-3’
JN807906  5’-...TGATTCCTGAAAGGAATGAACAAGGGCAGAC---GAATGTGAGAGGCTCAGGAATGAGAATAC...-3’
JN807897  5’-...TGATTCCTGAAAGGAATGAACAAGGGCAGAC---GAATGTGAGAGGCTCAGGAATGAGAATAC...-3’
KC261450  5’-...TGATTCCTGAAAGAAATGAACAAGGGCAGAC---GAATGTGAGAGGCTCAGGAATGAGAATAC...-3’
JN807898  5’-...TGATTCCTGAAAGGAATGAACAAGGGCAGAC---GAATGTGAGAGGCTCAGGAATGAGAATAC...-3’
JN807892  5’-...TGATTCCTGAAAGGAATGAACAAGGGCAGAC---GAATGTGAGAGGCTCAGGAATGAGAATAC...-3’
JN807896  5’-...TGATTCCTGAAAGGAATGAACAAGGGCAGAC---GAATGTGAGAGGCTCAGGAATGAGAATAC...-3’
AB684162  5’-...TGATTCCTGAAAGGAATGAACAAGGGCAGAC---GAATGTGAGAGGCTCAGGAATGAGAATAC...-3’
JQ701709  5’-...TGATCCCTGAAAGGAATGAACAAGGACAGAC---GAATGTGAGAGGCTCAGGAATGAGAATAC...-3’
CY089410  5’-...TGATTCCTGAAAGGAATGAACAAGGGCAGAC---GAATGTGAGAGGCTCAGGAATGAGAATAC...-3’
JQ701717  5’-...TGATCCCTGAAAGGAATGAACAAGGACAGAC---GAATGTGAGAGGCTCAGGAATGAGAATAC...-3’
JN807895  5’-...TGATTCCTGAAAGGAATGAACAAGGGCAGAC---GAATGTGAGAGGCTCAGGAATGAGAATAC...-3’
JQ701701  5’-...TGATCCCTGAAAGGAATGAACAAGGACAGAC---GAATGTGAGAGGCTCAGGAATGAGAATAC...-3’
AB684176  5’-...TGATTCCTGAAAGGAATGAACAAGGGCAGAC---GAATGTGAGAGGCTCAGGAATGAGAATAC...-3’
JQ714210  5’-...TGATCCCTGAAAGGAATGAACAAGGACAGAC---GAATGTGAGAGGCTCAGGAATGAGAATAC...-3’
KF182738  5’-...TGATTCCTGAAAGGAATGAACAAGGGCAGAC---GAATGTGAGGGGCTCAGGAATGAGAATAC...-3’
CY146641  5’-...TGATTCCTGAAAGGAATGAGCAAGGGCAGAC---GAATGTGAGGGGCTCAGGAATGAGAATAC...-3’
JX978719  5’-...TGATTCCTGAAAGAAATGAACAAGGACAGAC---GAATGTTAGAGGCTCAGGAATGAGAATAC...-3’
DQ992699  5’-...TGATCCCTGAACGGAATGAACAGGGACAGAC---GAATGTGAGAGGCTCAGGAATGAGAATAC...-3’
JN646698  5’-...TGATTCCTGAAAGGAATGAACAAGGGCAGAC---GAATGTGAGGGGATCAGGAATGAGAATAC...-3’
JN646695  5’-...TGATTCCTGAAAGGAATGAACAAGGGCAGAC---GAATGTGAGGGGATCAGGAATGAGAATAC...-3’
JN646696  5’-...TGATTCCTGAAAGGAATGAACAAGGGCAGAC---GAATGTGAGGGGATCAGGAATGAGAATAC...-3’
JN646693  5’-...TGATTCCTGAAAGGAATGAACAAGGGCAGAC---GAATGTGAGGGGATCAGGAATGAGAATAC...-3’
JN646694  5’-...TGATTCCTGAAAGGAATGAACAAGGGCAGAC---GAATGTGAGGGGATCAGGAATGAGAATAC...-3’
JN807909  5’-...TGATTCCTGAAAGGAATGAACAAGGGCAGAC---GAATGTGAGAGGCTCACGAATGAGAATAC...-3’
KC616590  5’-...TGATTCCTGAAAGGAATGAACAAGGGCAGAC---GAATGTGAGAGGCTCAGGAATGAGAATAC...-3’
KC616605  5’-...TGATTCCTGAAAGGAATGAACAAGGGCAGAC---GAATGTGAGAGGCTCAGGAATGAGAATAC...-3’
KC616589  5’-...TGATTCCTGAAAGGAATGAACAAGGGCAGAC---GAATGTGAGAGGCTCAGGAATGAGAATAC...-3’
AY651732  5’-...TGATTCCTGAAAGGAATGAACAAGGGCAGAC---GAATGTGAGAGGCTCAGGAATGAGAATAC...-3’
KC616594  5’-...TGATTCCTGAAAGGAATGAACAAGGGCAGAC---GAATGTGAGAGGCTCAGGAATGAGAATAC...-3’
AY585512  5’-...TGATTCCTGAAAGGAATGAACAAGGGCAGAC---GAATGTGAGAGGCTCAGGGATGAGAATAC...-3’
KC616602  5’-...TGATTCCTGAAAGGAATGAACAAGGGCAGAC---GAATGTGAGAGGCTCAGGAATGAGAATAC...-3’
HM172406  5’-...TGATTCCTGAAAGAAATGAACAAGGGCAGAC---GAATGTGAGAGGTTCAGGAATGAGAATAC...-3’
KC616604  5’-...TGATTCCTGAAAGGAATGAACAAGGGCAGAC---GAATGTGAGAGGCTCAGGAATGAGAATAC...-3’
KC616591  5’-...TGATTCCTGAAAGGAATGAACAAGGGCAGAC---GAATGTGAGAGGCTCAGGAATGAGAATAC...-3’
KC616592  5’-...TGATTCCTGAAAGGAATGAACAAGGGCAGAC---GAATGTGAGAGGCTCAGGAATGAGAATAC...-3’
KC616598  5’-...TGATTCCTGAAAGGAATGAACAAGGGCAGAC---GAATGTGAGAGGCTCAGGAATGAGAATAC...-3’
CY146697  5’-...TGATTCCTGAAAGGAATGAACAAGGGCAGAC---GAATGTGAGGGGGTCAGGAATGAGAATAC...-3’
KC616600  5’-...TGATTCCTGAAAGGAATGAACAAGGGCAGAC---GAATGTGAGAGGCTCAGGAATGAGAATAC...-3’
KC616593  5’-...TGATTCCTGAAAGGAATGAACAAGGGCAGAC---GAATGTGAGAGGCTCAGGAATGAGAATAC...-3’
KC616603  5’-...TGATTCCTGAAAGGAATGAACAAGGGCAGAC---GAATGTGAGAGGCTCAGGAATGAGAATAC...-3’
KC616599  5’-...TGATTCCTGAAAGGAATGAACAAGGGCAGAC---GAATGTGAGAGGCTCAGGAATGAGAATAC...-3’
DQ320813  5’-...TGATCCCTGAACGGAATGAACAGGGACAAAC---GAATGTGAGAGGCTCTGGAATGAGAATAC...-3’
KC616596  5’-...TGATTCCTGAAAGGAATGAACAAGGGCAGAC---GAATGTGAGAGGCTCAGGAATGAGAATAC...-3’
KC616597  5’-...TGATTCCTGAAAGGAATGAACAAGGGCAGAC---GAATGTGAGAGGCTCAGGAATGAGAATAC...-3’
KC616601  5’-...TGATTCCTGAAAGGAATGAACAAGGGCAGAC---GAATGTGAGAGGCTCAGGAATGAGAATAC...-3’
KC616595  5’-...TGATTCCTGAAAGAAATGAACAAGGGCAGAC---GAATGTGAGAGGCTCAGGAATGAGAATAC...-3’
KC616606  5’-...TGATTCCTGAAAGGAATGAACAAGGGCAGAC---GAATGTGAGAGGCTCAGGACTGAGAATAC...-3’
AY585510  5’-...TGATTCCTGAAAGGAATGAACAAGGACAAAC---GAATGTGGGAGGCTCAGGAATGAGAATAC...-3’
DQ835807  5’-...TGATTCCTGAAAGGAATGAACAAGGGCAGAC---GAATGTGAGAGGCTCAGGAATGAGAATAT...-3’
KF874284  5’-...TGATTCCTGAAAGGAATGAACAAGGGCAGAC---GAATGTGAGAGGCTCAGGAATGAGAATAC...-3’
AY585518  5’-...TGATTCCTGAAAGGAATGAACAGGGACAAAC---GAATGTGAGAGGCTCAGGGATGAGAATAC...-3’
KF638576  5’-...TGGTTCCTGAGAGAAATGAACAAGGGCAAAC---GAATGTGAGAGGCTCAGGAATGAGAATAC...-3’
AY059521  5’-...TGATTCCTGAAAGGAATGAACAAGGACAAAC---GAATGTGAGAGGCTCAGGAATGAGAATAC...-3’
```

```
                 224        235  240          254  1941          1956  1961     1969
                  *          *    *             *   *              *     *        *
AY585513  5'-...TGATTCCTGAAAGGAATGAACAAGGACAAAC---GAATGTGAGAGGCTCAGGAATGAGAATAC...-3'
AY585509  5'-...TGATTCCTGAAAGGAATGAACAAGGACAAAC---GAATGTGAGAGGCTCAGGAATGAGAATAC...-3'
AY059520  5'-...TGATTCCTGAAAGGAATGAACAAGGACAAAC---GAATGTGAGAGGCTCAGGAATGAGAATAC...-3'
AY585507  5'-...TGATTCCCGAAAGGAATGAGCAAGGGCAGAC---GAATGTGAGAGGATCAGGAATGAGAATAC...-3'
AY651747  5'-...TGATCCCTGAACGGAATGAACAGGGACAGAT---GAATGTGAGAGGTTCAGGAATGAGAATAC...-3'
GU721163  5'-...TGATTCCTGAAAGGAATGAACAAGGGCAAAC---GAATGTGAGAGGCTCAGGAATGAGAATAC...-3'
AY585517  5'-...TGATTCCTGAAAGGAATGAACAAGGGCAAAC---GAATGTGAGAGGCTCAGGAATGAGAATAC...-3'
AY585522  5'-...TGATTCCTGAAAGGAATGAACAAGGGCAAAC---GAATGTGAGAGGCTCAGGAATGAGAATAC...-3'
DQ351872  5'-...TGATTCCTGAAAGGAATGAACAAGGGCAAAC---GAATGTGAGAGGCTCAGGAATGAGAATAC...-3'
AY585521  5'-...TGATTCCTGAAAGGAATGAACAAGGGCAAAC---GAATGTGAGAGGCTCAGGAATGAGAATAC...-3'
AY585504  5'-...TGATTCCTGAAAGGAATGAACAAGGGCAAAC---GAATGTGAGAGGCTCAGGAATGAGAATAC...-3'
GU721160  5'-...TGATTCCTGAAAGGAATGAACAAGGGCAAAC---GAATGTGAGAGGCTCAGGAATGAGAATAC...-3'
AY585524  5'-...TGATCCCTGAACGGAATGAACAGGGACAGAC---GAATGTGAGAGGTTCAGGAATGAGAATAC...-3'
AY585505  5'-...TGATTCCTGAAAGGAATGAACAAGGGCAAAC---GAATGTGAGAGGCTCAGGAATGAGAATAC...-3'
CY029039  5'-...TGATTCCCGAAAGGAATGAACAAGGGCAAAC---GAATGTGAGAGGCTCAGGAATGAGAATAC...-3'
CY029088  5'-...TGATTCCCGAAAGGAATGAACAAGGGCAAAC---GAATGTGAGAGGCTCAGGAATGAGAATAC...-3'
CY029053  5'-...TGATTCCCGAAAGGAATGAACAAGGGCAAAC---GAATGTGAGAGGCTCAGGAATGAGAATAC...-3'
DQ992568  5'-...TGATCCCTGAACGGAATGAACAGGGACAAAC---GAATGTGAGAGGTTCAGGAATGAGAATAC...-3'
DQ992566  5'-...TGATCCCTGAACGGAATGAACAGGGACAAAC---GAATGTGAGAGGTTCAGGAATGAGAATAC...-3'
DQ992567  5'-...TGATCCCTGAACGGAATGAACAGGGACAAAC---GAATGTGAGAGGTTCAGGAATGAGAATAC...-3'
DQ992570  5'-...TGATCCCTGAACGGAATGAACAGGGACAAAC---GAATGTGAGAGGTTCAGGAATGAGAATAC...-3'
DQ992562  5'-...TGATCCCTGAACGGAATGAACAGGGACAAAC---GAATGTGAGAGGTTCAGGAATGAGAATAC...-3'
DQ992565  5'-...TGATCCCTGAACGGAATGAACAGGGACAAAC---GAATGTGAGAGGTTCAGGAATGAGAATAC...-3'
HM172441  5'-...TGATCCCTGAACGGAATGAACAGGGACAAAC---GAATGTGAGAGGTTCAGGAATGAGAATAC...-3'
DQ320845  5'-...TGATCCCTGAACGGAATGAACAGGGACAAAC---GAATGTGAGAGGTTCAGGAATGAGAATAC...-3'
DQ320834  5'-...TGGTCCCTGAACGGAATGAACAGGGACAAAC---GAATGTGAGAGGTTCAGGAATGAGAATAC...-3'
AY585520  5'-...TGATTCCCGAAAGGAATGAGCAAGGGCAGAC---GAATGTGAGAGGCTCGGGAATGAGAATAC...-3'
DQ320847  5'-...TGATCCCTGAACGGAATGAACAGGGACAAAC---GAATGTGAGAGGTTCAGGAATGAGAATAC...-3'
DQ992578  5'-...TGATCCCTGAACGGAATGAACAGGGACAGAC---AAATGTGAGAGGTTCAGGAATGAGAATAC...-3'
DQ992665  5'-...TGATCCCTGAACGGAATGAACAGGGACAAAC---GAATGTGAGAGGTTCAGGAATGAGAATAC...-3'
DQ992666  5'-...TGATCCCTGAACGGAATGAACAGGGACAAAC---GAATGTGAGAGGTTCAGGAATGAGAATAC...-3'
DQ320816  5'-...TGATCCCTGAACGGAATGAACAGGGACAAAC---GAATGTGAGAGGTTCAGGAATGAGAATAC...-3'
DQ320846  5'-...TGATCCCTGAACGGAATGAACAGGGACAAAC---GAATGTGAGAGGTTCAGGAATGAGAATAC...-3'
DQ320821  5'-...TGATCCCTGAACGGAATGAACAGGGACAAAC---GAATGTGAGAGGTTCAGGAATGAGAATAC...-3'
DQ320822  5'-...TGATCCCTGAACGGAATGAACAGGGACAAAC---GAATGTGAGAGGTTCAGGAATGAGAATAC...-3'
DQ992564  5'-...TGATCCCTGAACGGAATGAACAGGGACAAAC---GAATGTGAGAGGTTCAGGAATGAGAATAC...-3'
DQ992664  5'-...TGATCCCTGAACGGAATGAACAGGGACAAAC---GAATGTGAGAGGTTCAGGAATGAGAATAC...-3'
DQ320830  5'-...TGATCCCTGAACGGAATGAACAGGGACAAAC---GAATGTGAGAGGTTCAGGAATGAGAATAC...-3'
FJ784839  5'-...TGATCCCTGAACGGAATGAACAGGGACAAAC---GAATGTGAGAGGTTCAGGAATGAGAATAC...-3'
DQ992660  5'-...TGATCCCTGAACGGAATGAACAGGGACAAAC---GAATGTGAGAGGTTCAGGAATGAGAATAC...-3'
DQ992582  5'-...TGATCCCTGAACGGAATGAACAGGGACAAAC---GAATGTGAGAGGTTCAGGAATGAGAATAC...-3'
DQ992583  5'-...TGATCCCTGAACGGAATGAACAGGGACAAAC---GAATGTGAGAGGTTCAGGAATGAGAATAC...-3'
FJ784832  5'-...TGATCCCTGAACGGAATGAACAGGGACAAAC---GAATGTGAGAGGTTCAGGAATGAGAATAC...-3'
DQ320814  5'-...TGATCCCTGAACGGAATGAACAGGGACAAAC---GAATGTGAGAGGTTCAGGAATGAGAATAC...-3'
DQ992563  5'-...TGATCCCTGAACGGAATGAACAGGGACAAAC---GAATGTGAGAGGTTCAGGAATGAGAATAC...-3'
DQ992668  5'-...TGATCCCTGAACGGAATGAACAGGGACAAAC---GAATGTGAGAGGTTCAGGAATGAGAATAC...-3'
DQ320820  5'-...TGATCCCTGAACGGAATGAACAGGGACAAAC---GAATGTGAGAGGTTCAGGAATGAGAATAC...-3'
FJ784840  5'-...TGATCCCTGAACGGAATGAACAGGGACAAAC---GAATGTGAGAGGTTCAGGAATGAGAATAC...-3'
DQ320818  5'-...TGATCCCTGAACGGAATGAACAGGGACAAAC---GAATGTGAGAGGTTCAGGAATGAGAATAC...-3'
DQ992698  5'-...TGATCCCTGAACGGAATGAACAGGGACAGAC---AAATGTGAGAGGTTCAGGAATGAGAATAC...-3'
DQ992667  5'-...TGATCCCTGAACGGAATGAACAGGGACAAAC---GAATGTGAGAGGTTCAGGAATGAGAATAC...-3'
DQ992662  5'-...TGATCCCTGAACGGAATGAACAGGGACAAAC---GAATGTGAGAGGTTCAGGAATGAGAATAC...-3'
DQ992585  5'-...TGATCCCTGAACGGAATGAACAGGGACAAAC---GAATGTGAGGGGTTCAGGAATGAGAATAC...-3'
DQ992661  5'-...TGATCCCTGAACGGAATGAACAGGGACAAAC---GAATGTGAGAGGTTCAGGAATGAGAATAC...-3'
GQ202214  5'-...TGATCCCTGAACGGAATGAACAGGGACAAAC---GAATGTGAGAGGTTCAGGAATGAGAATAC...-3'
DQ992580  5'-...TGATCCCTGAACGGAATGAACAGGGACAGAC---AAATGTGAGAGGTTCAGGAATGAGAATAC...-3'
DQ992671  5'-...TGATCCCTGAACGGAATGAACAGGGACAAAC---GAATGTGAGAGGTTCAGGAATGAGAATAC...-3'
DQ992670  5'-...TGATCCCTGAACGGAATGAACAGGGACAAAC---GAATGTGAGAGGTTCAGGAATGAGAATAC...-3'
HM172425  5'-...TGATCCCTGAACGGAATGAACAGGGACAAAC---GAATGTGAGAGGTTCAGGAATGAGAATAC...-3'
DQ320825  5'-...TGATCCCTGAACGGAATGAACAGGGACAAAC---AAATGTGAGAGGTTCAGGAATGAGAATAC...-3'
FJ784834  5'-...TGATCCCTGAACGGAATGAACAGGGACAAAC---GAATGTGAGAGGTTCAGGAATGAGAATAC...-3'
DQ992579  5'-...TGATCCCTGAACGGAATGAACAGGGACAGAC---AAATGTGAGAGGTTCAGGAATGAGAATAC...-3'
DQ320817  5'-...TGATCCCTGAACGGAATGAACAGGGACAAAC---GAATGTGAGAGGTTCAGGAATGAGAATAC...-3'
DQ320824  5'-...TGATCCCTGAACGGAATGAACAGGGACAAAC---GAATGTGAGAGGTTCAGGAATGAGAATAC...-3'
DQ992669  5'-...TGATCCCTGAACGGAATGAACAGGGACAAAC---GAATGTGAGAGGTTCAGGAATGAGAATAC...-3'
DQ992581  5'-...TGATCCCTGAACGGAATGAACAGGGACAAAC---GAATGTGAGAGGTTCAGGAATGAGAATAC...-3'
CY029532  5'-...TGATCCCTGAACGGAATGAACAGGGACAAAC---GAATGTGAGAGGTTCAGGAATGAGAATAC...-3'
DQ073402  5'-...TGATCCCTGAAAGGAATGAACAAGGGCAAAC---GAATGTGAGAGGCTCAGGAATGAGAATAT...-3'
CY036042  5'-...TGATCCCTGAACGGAATGAACAGGGACAAAC---GAATGTGAGAGGTTCAGGAATGAGAATAC...-3'
HM172414  5'-...TGATCCCTGAACGGAATGAACAGGGACAAAC---GAATGTGAGAGGTTCAAGAATGAGAATAC...-3'
```

```
                224          235  240           254  1941           1956  1961     1969
                 *             *    *             *    *               *     *        *
JN646692   5'-...TGATCCCTGAACGAAATGAACAGGGACAGAC---GAACGTGAGAGGCTCAGGAATGAGAATAC...-3'
DQ992569   5'-...TGATCCCTGAACGGAATGAACAGGGACAAAC---GAATGTGAGAGGTTCAGGAATGAGAATAC...-3'
JN646697   5'-...TGATCCCTGAACGAAATGAACAGGGGCAGAC---GAACGTGAGAGGTTCAGGAATGAGAATAC...-3'
DQ073399   5'-...TGATTCCTGAAAGGAATGAACAAGGGCAGAT---GAATGTAAGAGGGTCAGGGATGAGAATAC...-3'
DQ073400   5'-...TGATCCCTGAAAGGAATGAACAAGGGCAGAC---GAATGTGAGGGGCTCAGGAATGAGAATAC...-3'
FJ784826   5'-...TGATCCCTGAAAGAAATGAGCAAGGTCAGAC---GAACGTAAGAGGCTCAGGAATGAGAATAC...-3'
DQ073401   5'-...TGATCCCTGAAAGAAATGAGCAAGGTCAAAC---GAATGTGAGGGGCTCAGGAATGAGAATAC...-3'
FJ784833   5'-...TGATCCCTGAAAGAAATGAGCAAGGTCAGAC---AAACGTGAGGGGTTCAGGAATGAGAATAC...-3'
FJ784838   5'-...TGATCCCTGAAAGAAATGAGCAGGGTCAGAC---GAACGTGAGGGGGTTCAGGAATGAGAATAC...-3'
KC815848   5'-...TGATCCCTGAAAGGAATGAACAAGGTCAAAC---AAACGTAAGGGGTTCAGGAATGAGGATAC...-3'
KC815856   5'-...TGATCCCTGAAAGGAATGAACAAGGTCAAAC---AAACGTAAGGGGTTCAGGAATGAGGATAC...-3'
KC815872   5'-...TGATCCCTGAAAGGAATGAACAAGGTCAAAC---AAACGTAAGGGGTTCAGGAATGAGGATAC...-3'
GU220792   5'-...TGATCCCTGAAAGAAATGAGCAGGGTCAGAC---GAACGTGAGGGGTTCAGGAATGAGAATAC...-3'
FJ784837   5'-...TGATCCCTGAAAGAAATGAGCAGGGTCAGAC---GAACGTGAGGGGTTCAGGAATGAGAATAC...-3'
FJ784825   5'-...TGATCCCCGAAAGAAATGAGCAGGGTCAGAC---GAACGTGAGGGGTTCAGGAATGAGAATAC...-3'
DQ992684   5'-...TGATCCCTGAAAGAAATGAGCAAGGTCAAAC---GAACGTAAGGGGCTCAGGAATGAGAATAC...-3'
FJ784836   5'-...TGATCCCTGAAAGAAATGAGCAGGGTCAGAC---GAACGTGGGGGGTTCAGGAATGAGAATAC...-3'
FJ784835   5'-...TGATCCCTGAAAGAAATGAGCAGGGTCAGAC---GAACGTAAGGGGTTCAGGAATGAGAATAC...-3'
CY046179   5'-...TGATCCCTGAAAGAAATGAGCAAGGTCAGAC---GAACGTAAGGGGTTCAGGAATGAGAATAC...-3'
FJ784830   5'-...TGATCCCTGAAAGAAATGAGCAGGGTCAGAC---GAACGTGAGGGGTTCAGGAATGAGAATAC...-3'
FJ784828   5'-...TGATCCCTGAAAGAAATGAGCAGGGTCAGAC---GAACGTGAGGGGTTCAGGAATGAGAATAC...-3'
AB212277   5'-...TAATCCCTGAAAGAAATGAGCAAGGTCAGAC---GAACGTAAGGGGCTCAGGAATGAGAATAC...-3'
KC815864   5'-...TGATCCCTGAAAGGAATGAACAAGGTCAAAC---AAACGTAAGGGGTTCAGGAATGAGGATAC...-3'
EU195389   5'-...TGATCCCGGAAAGAAATGAGCAAGGTCAAAC---GAACGTGAGGGGGTTCAGGAATGAGAATAC...-3'
CY040971   5'-...TGATCCCGGAAAGAAATGAGCAAGGTCAGAC---GAACGTGAGGGGTTCAGGAATGAGAATAC...-3'
CY040923   5'-...TGATCCCGGAAAGAAATGAGCGAGGTCAGAC---GAACGTGAGGGGTTCAGGAATGAGAATAC...-3'
CY030998   5'-...TGATCCCTGAAAGAAATGAGCAAGGTCAAAC---GAACGTGAGGGGTTCAGGAATGAGAATAC...-3'
JF758818   5'-...TGATTCCTGARAGRAATGARCARGGACAAAC---GAATGTAAGGGGATCAGGAATGAGAATAC...-3'
JF758815   5'-...TGATTCCTGAAAGAAATGAACAGGGACAAAC---GAATGTAAGGGGATCAGGAATGAGAATAC...-3'
CY036702   5'-...TGATTCCTGAAAGGAATGAACAAGGGCAGAC---GAATGTGAGAGGCTCAGGAATGAGAATAC...-3'
EU148387   5'-...TGATTCCTGAAAGGAATGAACAGGGACAGAC---GAATGTGAGAGGCTCAGGAATGAGAATAC...-3'
CY094806   5'-...TGATTCCTGAAAGGAATGAACAAGGGCAGAC---GAATGTGAGAGGCTCAGGAATGAGAATAC...-3'
DQ990000   5'-...TGATTCCTGAAAGGAATGAACAAGGGCAGAC---GAATGTGAGAGGCTCAGGAATGAGAATAC...-3'
EU616866   5'-...TGATTCCTGAAAGGAATGAACAAGGGCAGAC---GAATGTGAGAGGCTCAGGAATGAGAATAC...-3'
CY045406   5'-...TGATTCCTGAAAGAAATGAACAGGGACAAAC---GAATGTAAGGGGATCAGGAATGAGAATAC...-3'
CY033176   5'-...TGATTCCTGAAAGGAATGAACAAGGGCAGAC---GAATGTGAGAGGCTCAGGAATGAGAATAC...-3'
CY034205   5'-...TGATTCCTGAAAGGAATGAACAAGGGCAGAC---GAATGTGAGAGGCTCAGGAATGAGAATAC...-3'
CY020644   5'-...TGATTCCTGAAAGGAATGAACAAGGACAGAC---GAATGTGAGAGGCTCAGGAATGAGAATAC...-3'
CY017034   5'-...TGATTCCTGAAAGGAATGAACAGGGACAGAC---GAATGTGAGAGGCTCAGGAATGAGAATAC...-3'
CY036718   5'-...TGATTCCTGAAAGGAATGAACAAGGGCAGAC---GAATGTGAGAGGCTCAGGAATGAGAATAC...-3'
EU148451   5'-...TGATTCCTGAAAGGAATGAACAGGGACAGAC---GAATGTGAGAGGCTCAGGAATGAGAATAC...-3'
CY016834   5'-...TGATTCCTGAAAGGAATGAACAAGGGCAGAC---GAATGTGAGAGGCTCAGGAATGAGAATAC...-3'
CY016874   5'-...TGATCCCTGAACGGAATGAACAGGGACAAAC---GAATGTGAGAGGTTCAGGAATGAGAATAC...-3'
CY017058   5'-...TGATCCCTGAACGGAATGAACAGGGACAAAC---GAATGTGAGAGGTTCAGGAATGAGAATAC...-3'
CY043935   5'-...TGATTCCTGAAAGAAATGAACAGGGACAAAC---GAATGTAAGGGGATCAGGAATGAGAATAC...-3'
CY031179   5'-...TGATTCCTGAAAGGAATGAACAAGGGCAGAC---GAATGTGAGAGGCTCAGGAATGAGAATAC...-3'
EF112210   5'-...TGATTCCTGAAAGGAATGAACAAGGGCAGAC---GAATGTGAGAGGCTCAGGAATGAGAATAC...-3'
AY038798   5'-...TGATTCCTGAAAGGAATGAACAAGGACAAAC---GAATGTGAGAGGCTCAGGAATGAGAATAC...-3'
HM627910   5'-...TGATTCCTGAAAGGAATGAACAAGGGCAGAC---GAATGTGAGAGGCTCAGGAATGAGAATAC...-3'
AB593444   5'-...TGATCCCTGAAAGGAATGAACAAGGGCAGAC---GAATGTGAGAGGCTCAGGAATGAGGATAC...-3'
CY016946   5'-...TGATTCCTGAAAGGAATGAACAGGGACAGAC---GAATGTGAGAGGCTCAGGAATGAGAATAC...-3'
EU148403   5'-...TGATTCCTGAAAGGAATGAACAGGGACAGAC---GAATGTGAGAGGCTCAGGAATGAGAATAC...-3'
EF112213   5'-...TGATTCCTGAAAGGAATGAACAAGGGCAGAC---GAATGTGAGAGGCTCAGGAATGAGAATAC...-3'
CY020668   5'-...TGATTCCTGAAAGGAATGAACAAGGACAGAC---GAATGTTAGAGGCTCAGGAATGAGAATAC...-3'
EF112218   5'-...TGATTCCTGAAAGGAATGAACAAGGGCAGAC---GAATGTGAGAGGCTCAGGAATGAGAATAC...-3'
EF112206   5'-...TGATTCCTGAAAGGAATGAACAAGGGCAGAC---GAATGTGAGAGGCTCAGGAATGAGAATAC...-3'
CY016954   5'-...TGATTCCTGAAAGGAATGAACAGGGACAGAC---GAATGTGAGAGGCTCAGGAATGAGAATAC...-3'
CY029944   5'-...TGATTCCTGAAAGGAATGAACAAGGGCAGAC---GAATGTGAGAGGCTCAGGAATGAGAATAC...-3'
CY028723   5'-...TGATTCCTGAAAGGAATGAACAAGGGCAGAC---GAATGTGAGAGGCTCAGGAATGAGAATAC...-3'
CY036710   5'-...TGATTCCTGAAAGGAATGAACAAGGGCAGAC---GAATGTGAGAGGCTCAGGAATGAGAATAC...-3'
JQ714224   5'-...TGATCCCTGAAAGGAATGAACAAGGACAGAC---GAATGTGAGAGGCTCAGGAATGAGAATAC...-3'
JQ714232   5'-...TGATCCCTGAAAGGAATGAACAAGGACAGAC---GAATGTGAGAGGCTCAGGAATGAGAATAC...-3'
CY053332   5'-...TGATTCCTGAAAGAAATGAACAGGGACAAAC---GAATGTAAGGGGATCAGGAATGAGAATAC...-3'
AY651728   5'-...TGATTCCTGAAAGGAATGAACAAGGGCAGAC---GAATGTGAGAGGCTCAGGAATGAGAATAC...-3'
CY020356   5'-...TGATTCCTGAAAGGAATGAACAAGGACAGAC---GAATGTTAGAGGCTCAGGAATGAGAATAC...-3'
DQ323675   5'-...TGATTCCTGAAAGGAATGAACAAGGACAGAC---GAATGTTAGAGGCTCAGGAATGAGAATAC...-3'
EU148395   5'-...TGATTCCTGAAAGGAATGAACAGGGACAAAC---GAATGTGAGAGGCTCAGGAATGAGAATAC...-3'
DQ989984   5'-...TGATTCCTGAAAGGAATGAACAAGGGCAGAC---GAATGTGAGAGGCTCAGGAATGAGAATAC...-3'
EF112221   5'-...TGATTCCTGAAAGGAATGAACAAGGGCAGAC---GAATGTGAGAGGCTCAGGAATGAGAATAC...-3'
CY053884   5'-...TGATTCCTGAAAGAAATGAACAGGGACAAAC---GAATGTAAGGGGATCAGGAATGAGAATAC...-3'
```

```
               224        235  240          254 1941           1956 1961    1969
                *          *    *             *   *              *    *       *
AM503073  5’-...TGATTCCTGAAAGGAATGAACAGGGACAGAC---GAATGTGAGAGGCTCAGGAATGAGAATAC...-3’
AM503068  5’-...TGATTCCTGAAAGGAATGAACAGGGACAGAC---GAATGTGAGAGGCTCAGGAATGAGAATAC...-3’
HM627926  5’-...TGATTCCTGAAAGGAATGAACAAGGGCAGAC---GAATGTGAGAGGCTCAGGAATGAGAATAC...-3’
AM503071  5’-...TGATTCCTGAAAGGAATGAACAGGGACAAAC---GAATGTGAGAGGCTCAGGAATGAGAATAC...-3’
AM503072  5’-...TGATTCCTGAAAGGAATGAACAGGGACAAAC---GAATGTGAGAGGCTCAGGAATGAGAATAC...-3’
AM503070  5’-...TGATTCCTGAAAGGAATGAACAGGGACAGAC---GAATGTGAGAGGCTCAGGAATGAGAATAC...-3’
AM503069  5’-...TGATTCCTGAAAGGAATGAACAGGGACAGAC---GAATGTGAGAGGCTCAGGAATGAGAATAC...-3’
AM503066  5’-...TGATTCCTGAAAGGAATGAACAAGGACAGAC---GAATGTTAGAGGCTCAGGAATGAGAATAC...-3’
AM503067  5’-...TGATTCCTGAAAGGAATGAACAAGGACAGAC---GAATGTTAGAGGCTCAGGAATGAGAATAC...-3’
AM503074  5’-...TGATTCCTGAAAGGAATGAACAGGGACAGAC---GAATGTGAGAGGCTCAGGAATGAGAATAC...-3’
EU277840  5’-...TGATTCCTGAAAGGAATGAACAAGGACAGAC---GAATGTTAGAGGCTCAGGAATGAGAATAC...-3’
EF112204  5’-...TGATTCCTGAAAGGAATGAACAAGGGCAGAC---GAATGTGAGAGGCTCAGGAATGAGAATAC...-3’
EF112216  5’-...TGATTCCTGAAAGGAATGAACAAGGGCAGAC---GAATGTGAGAGGCTCAGGAATGAGAATAC...-3’
EF112215  5’-...TGATTCCTGAAAGGAATGAACAAGGGCAGAC---GAATGTGAGAGGCTCAGGAATGAGAATAC...-3’
DQ989992  5’-...TGATTCCTGAAAGGAATGAACAAGGGCAGAC---GAATGTGAGAGGCTCAGGAATGAGAATAC...-3’
EF112208  5’-...TGATTCCTGAAAGGAATGAACAAGGGCAGAC---GAATGTGAGAGGCTCAGGAATGAGAATAC...-3’
CY028715  5’-...TGATTCCTGAAAGGAATGAACAAGGGCAGAC---GAATGTGAGAGGCTCAGGAATGAGAATAC...-3’
CY028707  5’-...TGATTCCTGAAAGGAATGAACAAGGGCAGAC---GAATGTGAGAGGCTCAGGAATGAGAATAC...-3’
CY016802  5’-...TGATTCCTGAAAGGAATGAACAAGGACAGAC---GAATGTTAGAGGCTCAGGAATGAGAATAC...-3’
DQ989966  5’-...TGATTCCTGAAAGGAATGAACAAGGGCAGAC---GAATGTGAGAGGCTCAGGAATGAGAATAC...-3’
EF112220  5’-...TGATTCCTGAAAGGAATGAACAAGGGCAGAC---GAATGTGAGAGGCTCAGGAATGAGAATAC...-3’
EF112219  5’-...TGATTCCTGAAAGGAATGAACAAGGGCAGAC---GAATGTGAGAGGCTCAGGAATGAGAATAC...-3’
EF112201  5’-...TGATTCCTGAAAGGAATGAACAAGGGCAGAC---GAATGTGAGAGGCTCAGGAATGAGAATAC...-3’
EU669195  5’-...TGATTCCTGAAAGGAATGAACAAGGGCAGAC---GAATGTGAGAGGATCAGGAATGAGAATAC...-3’
CY092133  5’-...TGATTCCTGAAAGGAATGAACATGGGCAGAC---GAATGTGAGAGGCTCAGGAATGAGAATAC...-3’
CY017066  5’-...TGATCCCTGAACGGAATGAACAGGGACAAAC---GAATGTGAGAGGTTCAGGAATGAGAATAC...-3’
CY017186  5’-...TGATTCCTGAAAGGAATGAACAGGGACAGAC---GAATGTGAGAGGCTCAGGAATGAGAATAC...-3’
CY016826  5’-...TGATTCCTGAAAGGAATGAACAGGGACAGAC---GAATGTGAGAGGCTCAGGAATGAGAATAC...-3’
CY016914  5’-...TGATTCCTGAAAGGAATGAACAGGGACAGAC---GAATGTGAGAGGCTCAGGAATGAGAATAC...-3’
CY021380  5’-...TGATTCCTGAAAGGAATGAACAAGGACAGAC---GAATGTGAGAGGCTCAGGAATGAGAATAC...-3’
CY020636  5’-...TGATTCCTGAAAGGAATGAACAAGGACAGAC---GAATGTGAGAGGCTCAGGAATGAGAATAC...-3’
CY016794  5’-...TGATTCCTGAGAGGAATGAACAAGGACAGAC---GAATGTGAGAGGCTCAGGAATGAGAATAC...-3’
CY016922  5’-...TGATTCCTGAAAGGAATGAACAGGGACAGAC---GAATGTGAGAGGCTCAGGAATGAGAATAC...-3’
CY016938  5’-...TGATTCCTGAAAGGAATGAACAGGGACAGAC---GAATGTGAGAGGCTCAGGAATGAGAATAC...-3’
CY016786  5’-...TGATTCCTGAAAGGAATGAACAAGGACAGAC---GAATGTGAGAGGCTCAGGAATGAGAATAC...-3’
CY016291  5’-...TGATTCCTGAAAGGAATGAACAGGGACAGAC---GAATGTGAGAGGCTCAGGAATGAGAATAC...-3’
CY020628  5’-...TGATTCCTGAAAGGAATGAACAAGGACAGAC---GAATGTGAGAGGCTCAGGAATGAGAATAC...-3’
CY020652  5’-...TGATTCCTGAAAGGAATGAACAAGGACAGAC---GAATGTTAGAGGCTCAGGAATGAGAATAC...-3’
CY016930  5’-...TGATTCCTGAAAGGAATGAACAGGGACAGAC---GAATGTGAGAGGCTCAGGAATGAGAATAC...-3’
CY034229  5’-...TGATTCCTGAAAGGAATGAACAAGGGCAGAC---GAATGTGAGAGGCTCAGGAATGAGAATAC...-3’
CY094854  5’-...TGATTCCTGAAAGGAATGAACAAGGGCAGAC---GAATGTGAGAGGCTCAGGAATGAGAATAC...-3’
CY031634  5’-...TGATTCCTGAAAGGAATGAACAAGGGCAGAC---GAATGTGAGAGGCTCAGGAATGAGAATAC...-3’
CY036726  5’-...TGATTCCTGAAAGGAATGAACAAGGGCAGAC---GAATGTGAGAGGCTCAGGAATGAGAATAC...-3’
CY017050  5’-...TGATTCCTGAAAGGAATGAACAAGGACAGAC---GAATGTTAGAGGCTCAGGAATGAGAATAC...-3’
EU148371  5’-...TGATTCCTGAAAGGAATGAACAGGGACAAAC---GAATGTGAGAGGCTCAGGAATGAGAATAC...-3’
CY035905  5’-...TGATTCCTGAAAGGAATGAACAAGGGCAGAC---GAATGTGAGAGGCTCAGGAATGAGAATAC...-3’
CY016283  5’-...TGATTCCTGAAAGGAATGAACAAGGACAGAC---GAATGTTAGAGGCTCAGGAATGAGAATAC...-3’
CY016882  5’-...TGATTCCTGAAAGGAATGAACAAGGGCAGAC---GAATGTGAGAGGCTCAGGAATGAGAATAC...-3’
CY016810  5’-...TGATTCCTGAAAGGAATGAACAAGGACAGAC---GAATGTTAGAGGCTCAGGAATGAGAATAC...-3’
CY021524  5’-...TGATTCCTGAAAGGAATGAACAAGGACAGAC---GAATGTTAGAGGCTCAGGAATGAGAATAC...-3’
CY016818  5’-...TGATTCCTGAAAGGAATGAACAAGGACAGAC---GAATGTTAGAGGCTCAGGAATGAGAATAC...-3’
EU574919  5’-...TGATTCCTGAAAGGAATGAACAAGGACAGAC---GAATGTTAGAGGCTCAGGAATGAGAATAC...-3’
CY017042  5’-...TGATTCCTGAAAGGAATGAACAAGGACAGAC---GAATGTGAGAGGCTCAGGAATGAGAATAC...-3’
CY094838  5’-...TGATTCCTGAAAGGAATGAACAAGGGCAGAC---GAATGTGAGAGGCTCAGGAATGAGAATAC...-3’
CY034221  5’-...TGATTCCTGAAAGGAATGAACAAGGGCAGAC---GAATGTGAGAGGCTCAGGAATGAGAATAC...-3’
CY094814  5’-...TGATTCCTGAAAGGAATGAACAAGGGCAGAC---GAATGTGAGAGGCTCAGGAATGAGAATAC...-3’
CY016906  5’-...TGATTCCTGAAAGGAATGAACAAGGACAGAC---GAATGTTAGAGGCTCAGGAATGAGAATAC...-3’
EU148379  5’-...TGATTCCTGAAAGGAATGAACAGGGACAGAC---GAATGTGAGAGGCTCAGGAATGAGAATAC...-3’
CY031626  5’-...TGATTCCTGAAAGGAATGAACAAGGGCAGAC---GAATGTGAGAGGCTCAGGAATGAGAATAC...-3’
CY031187  5’-...TGATTCCTGAAAGGAATGAACAAGGGCAGAC---GAATGTGAGAGGCTCAGGAATGAGAATAC...-3’
CY033184  5’-...TGATTCCTGAAAGGAATGAACAAGGGCAGAC---GAATGTGAGAGGCTCAGGAATGAGAATAC...-3’
CY017194  5’-...TGATTCCTGAAAGGAATGAACAAGGGCAGAC---GAATGTGAGAGGCTCAGGAATGAGAATAC...-3’
CY016898  5’-...TGATTCCTGAAAGGAATGAACAAGGGCAGAC---GAATGTGAGAGGCTCAGGAATGAGAATAC...-3’
CY094830  5’-...TGATTCCTGAAAGGAATGAACAAGGGCAGAC---GAATGTGAGAGGCTCAGGAATGAGAATAC...-3’
FN186014  5’-...TGATTCCTGAAAGGAATGAACAGGGACAGAC---GAATGTAAGGGGCTCAGGAATGAGAATAC...-3’
EU148411  5’-...TGATTCCTGAAAGGAATGAACAGGGACAGAC---GAATGTGAGAGGCTCAGGAATGAGAATAC...-3’
EU148363  5’-...TGATTCCTGAAAGGAATGAACAGGGACAGAC---GAATGTGAGAGGCTCAGGAATGAGAATAC...-3’
CY021396  5’-...TGATTCCTGAAAGGAATGAACAAGGACAGAC---GAATGTTAGAGGCTCAGGAATGAGAATAC...-3’
EU148435  5’-...TGATTCCTGAAAGGAATGAACAGGGACAGAC---GAATGTGAGAGGCTCAGGAATGAGAATAC...-3’
CY094846  5’-...TGATTCCTGAAAGGAATGAACAAGGGCAGAC---GAATGTGAGAGGCTCAGGAATGAGAATAC...-3’
```

```
                224        235  240           254 1941           1956 1961    1969
                 *          *    *             *   *              *    *       *
CY017074   5′-...TGATTCCTGAAAGGAATGAACAAGGGCAGAC---GAATGTGAGAGGCTCAGGAATGAGAATAC...-3′
EU148427   5′-...TGATTCCTGAAAGGAATGAACAGGGACAGAC---GAATGTGAGAGGCTCAGGAATGAGAATAC...-3′
CY020684   5′-...TGATTCCTGAAAGGAATGAACAAGGACAGAC---GAATGTTAGAGGCTCAGGAATGAGAATAC...-3′
CY034781   5′-...TGATTCCTGAAAGGAATGAACAAGGGCAGAC---GAATGTGAGAGGCTCAGGAATGAGAATAC...-3′
EF112217   5′-...TGATTCCTGAAAGGAATGAACAAGGGCAGAC---GAATGTGAGAGGCTCAGGAATGAGAATAC...-3′
CY016299   5′-...TGATTCCTGAAAGGAATGAACAAGGACAGAC---GAATGTTAGAGGCTCAGGAATGAGAATAC...-3′
CY020716   5′-...TGATTCCTGAAAGGAATGAACAAGGACAGAC---GAATGTTAGAGGCTCAGGAATGAGAATAC...-3′
CY020700   5′-...TGATTCCTGAAAGGAATGAACAAGGACAGAC---GAATGTTAGAGGCTCAGGAATGAGAATAC...-3′
CY020708   5′-...TGATTCCTGAAAGGAATGAACAAGGACAGAC---GAATGTTAGAGGCTCAGGAATGAGAATAC...-3′
DQ650670   5′-...TGATTCCTGAAAGGAATGAACAAGGACAGAC---GAATGTAAGGGGCTCAGGAATGAGAATAC...-3′
CY016307   5′-...TGATTCCTGAAAGGAATGAACAAGGACAGAC---GAATGTTAGAGGCTCAGGAATGAGAATAC...-3′
EU148419   5′-...TGATTCCTGAAAGGAATGAACAGGGACAGAC---GAATGTGAGAGGCTCAGGAATGAGAATAC...-3′
CY020676   5′-...TGATTCCTGAAAGGAATGAACAAGGACAGAC---GAATGTTAGAGGCTCAGGAATGAGAATAC...-3′
AY651707   5′-...TGATTCCTGAAAGGAATGAACAAGGGCAGAC---GAATGTGAGAGGCTCTGGAATGAGAATAC...-3′
AY651705   5′-...TGATTCCTGAAAGGAATGAACAAGGGCAGAC---GAATGTGAGAGGCTCTGGAATGAGAATAC...-3′
CY033192   5′-...TGATTCCTGAAAGGAATGAACGAGGGCAGAC---GAATGTGAGAGGCTCAGGAATGAGAATAC...-3′
CY016850   5′-...TGATTCCTGAAAGGAATGAACGAGGGCAGAC---GAATGTGAGAGGCTCAGGAATGAGAATAC...-3′
CY016858   5′-...TGATTCCTGAAAGGAATGAACGAGGGCAGAC---GAATGTGAGAGGCTCAGGAATGAGAATAC...-3′
CY016866   5′-...TGATTCCTGAAAGGAATGAACGAGGGCAGAC---GAATGTGAGAGGCTCAGGAATGAGAATAC...-3′
GQ337909   5′-...TGATTCCTGAAAGGAATGAACAAGGACAGAC---GAATGTGAGAGGCTCAGGAATGAGAATAC...-3′
CY016842   5′-...TGATTCCTGAAAGGAATGAACAAGGGCAGAC---GAATGTGAGAGGCTCAGGAATGAGAATAC...-3′
EF112203   5′-...TGATTCCTGAAAGGAATGAACAAGGGCAGAC---GAATGTGAGAGGCTCAGGAATGAGAATAC...-3′
FM164848   5′-...TGATTCCTGAAAGGAATGAACAGGGACAGAC---GAATGTGAGAGGCTCCGGAATGAGAATAC...-3′
AY651706   5′-...TGATTCCTGAAAGGAATGAACAAGGGCAGAC---GAATGTGAGAGGCTCTGGAATGAGAATAC...-3′
CY016890   5′-...TGATCCCTGAACGGAATGAACAGGGACAAAC---GAATGTGAGAGGTTCAGGAATGAGAATAC...-3′
CY021404   5′-...TGATCCCTGAAAGGAATGAACAAGGTCAAAC---AAACGTAAGGGGTTCAGGAATGAGGATAC...-3′
EU930963   5′-...TGATTCCTGAAAGGAATGAACAAGGGCAGAC---GAATGTGAGAGGCTCAGGAATGAGAATAC...-3′
EU930971   5′-...TGATTCCTGAAAGGAATGAACAAGGGCAGAC---GAATGTGAGAGGCTCAGGAATGAGAATAC...-3′
EU930979   5′-...TGATTCCTGAAAGGAATGAACAAGGGCAGAC---GAATGTGAGAGGCTCAGGAATGAGAATAC...-3′
EU930987   5′-...TGATTCCTGAAAGGAATGAACAAGGGCAGAC---GAATGTGAGAGGCTCAGGAATGAGAATAC...-3′
EU930955   5′-...TGATTCCTGAAAGGAATGAACAAGGGCAGAC---GAATGTGAGAGGCTCAGGAATGAGAATAC...-3′
EU931027   5′-...TGATTCCTGAAAGGAATGAACAAGGGCAGAC---GAATGTGAGAGGCTCAGGAATGAGAATAC...-3′
EU931019   5′-...TGATTCCTGAAAGGAATGAACAAGGGCAGAC---GAATGTGAGAGGCTCAGGAATGAGAATAC...-3′
EU931011   5′-...TGATTCCTGAAAGGAATGAACAAGGGCAGAC---GAATGTGAGAGGCTCAGGAATGAGAATAC...-3′
EU931003   5′-...TGATTCCTGAAAGGAATGAACAAGGGCAGAC---GAATGTGAGAGGCTCAGGAATGAGAATAC...-3′
EU930947   5′-...TGATTCCTGAAAGGAATGAACAAGGGCAGAC---GAATGTGAGAGGCTCAGGAATGAGAATAC...-3′
EU930899   5′-...TGATTCCTGAAAGGAATGAACAAGGGCAGAC---GAATGTGAGAGGCTCAGGAATGAGAATAC...-3′
EU930995   5′-...TGATTCCTGAAAGGAATGAACAAGGGCAGAC---GAATGTGAGAGGCTCAGGAATGAGAATAC...-3′
CY020660   5′-...TGATTCCTGAAAGGAATGAACAAGGACAGAC---GAATGTTAGAGGCTCAGGAATGAGAATAC...-3′
EU930883   5′-...TGATTCCTGAAAGGAATGAACAAGGGCAGAC---GAATGTGAGAGGCTCAGGAATGAGAATAC...-3′
EF112209   5′-...TGATTCCTGAAAGGAATGAACAAGGGCAGAC---GAATGTGAGAGGCTCAGGAATGAGAATAC...-3′
EU930923   5′-...TGATTCCTGAACGGAATGAACAGGGACAAAC---GAATGTGAGAGGCTCAGGAATGAGAATAC...-3′
AF468840   5′-...TGATTCCTGAAAGGAATGAACAAGGGCAAAC---GAATGTGAGAGGCTCAGGAATGAGAATAC...-3′
EU930891   5′-...TGATCCCTGAACGGAATGAACAGGGACAAAC---GAATGTGAGAGGTTCAGGAATGAGAATAC...-3′
EU930915   5′-...TGATCCCTGAACGGAATGAACAGGGACAAAC---GAATGTGAGAGGTTCAGGAATGAGAATAC...-3′
EU930907   5′-...TGATCCCTGAACGGAATGAACAGGGACAAAC---GAATGTGAGAGGTTCAGGAATGAGAATAC...-3′
CY095584   5′-...TGATCCCTGAAAGGAATGAACAAGGTCAAAC---AAACGTAAGGGGTTCAGGAATGAGGATAC...-3′
CY046134   5′-...TGATTCCTGAAAGAAATGAGCAAGGTCAGAC---GAATGTAAGGGGTTCAGGAATGAGAATAC...-3′
CY095489   5′-...TGATTCCTGAAAGGAACGAACAAGGACAAAC---AAATGTGAGAGGATCAGGAATGAGAATAC...-3′
CY095314   5′-...TGATTCCTGAAAGAAATGAACAGGGACAAAC---GAATGTAAGGGGATCAGGAATGAGAATAC...-3′
CY077117   5′-...TGATTCCTGAAAGAAATGAACAGGGACAAAC---GAATGTAAGGGGATCAGGAATGAGAATAC...-3′
CY133964   5′-...TGATCCCTGAAAGGAATGAACAAGGGCAAAC---GAATGTGAGGGGATCAGGAATGAGAATAC...-3′
CY133996   5′-...TGATCCCTGAAAGGAATGAACAAGGGCAAAC---GAATGTGAGAGGATCAGGAATGAGAATAC...-3′
CY133948   5′-...TGATCCCTGAAAGGAATGAACAAGGGCAAAC---GAATGTGAGAGGATCAGGAATGAGAATAC...-3′
CY134012   5′-...TGATCCCTGAAAGGAATGAACAAGGGCAAAC---GAATGTGAGAGGATCAGGAATGAGAATAC...-3′
CY133956   5′-...TGATCCCTGAAAGGAATGAACAAGGGCAAAC---GAATGTGAGAGGATCAGGAATGAGAATAC...-3′
CY133988   5′-...TGATCCCTGAAAGGAATGAACAAGGGCAAAC---GAATGTGAGAGGATCAGGAATGAGAATAC...-3′
CY134004   5′-...TGATCCCTGAAAGGAATGAACAAGGGCAAAC---GAATGTGAGAGGATCAGGAATGAGAATAC...-3′
CY134028   5′-...TGATCCCTGAAAGGAATGAACAAGGGCAAAC---GAATGTGAGAGGATCAGGAATGAGAATAC...-3′
AF046086   5′-...TGATCCCTGAAAGGAATGAGCAAGGTCAAAC---GAATGTGAGGGGATCAGGAATGAGAATAC...-3′
AB264769   5′-...TGATTCCTGAAAGGAATGAACAAGGACAGAC---GAATGTGAGAGGCTCAGGAATGAGAATAC...-3′
HM627934   5′-...TGATTCCTGAAAGGAATGAACAAGGGCAGAC---GAATGTGAGAGGCTCAGGAATGAGAATAC...-3′
AB284321   5′-...TGATTCCTGAAAGGAATGAACAAGGACAGAC---GAATGTGAGAGGCTCAGGAATGAGAATAC...-3′
EF112207   5′-...TGATTCCTGAAAGGAATGAACAAGGGCAGAC---GAATGTGAGAGGCTCAGGAATGAGAATAC...-3′
AB436547   5′-...TGATTCCTGAAAGGAATGAACAAGGGCAGAC---GAATGTAAGAGGCTCAGGAATGAGAATAC...-3′
AB523756   5′-...TGATTCCTGAAAGGAATGAACAAGGGCAGAC---GAATGTGAGAGGCTCAGGAATGAGAATAC...-3′
AB520705   5′-...TGATTCCTGAAAGGAATGAACAAGGGCAGAC---GAATGTGAGAGGCTCAGGAATGAGAATAC...-3′
AB523764   5′-...TGATTCCTGAAAGGAATGAACAAGGGCAGAC---GAATGTGAGAGGCTCAGGAATGAGAATAC...-3′
AB523772   5′-...TGATTCCTGAAAGGAATGAACAAGGGCAGAC---GAATGTGAGAGGCTCAGGAATGAGAATAC...-3′
AB458244   5′-...TGATTCCTGAAAGGAATGAACAAGGGCAGAC---GAATGTAAGAGGCTCAGGAATGAGAATAC...-3′
```

```
               224        235  240           254 1941           1956 1961    1969
                *          *    *             *   *              *    *       *
AB458236  5′-...TGATTCCTGAAAGGAATGAACAAGGGCAGAC---GAATGTAAGAGGCTCAGGAATGAGAATAC...-3′
AB569345  5′-...TGATTCCTGAAAGGAATGAACAAGGGCAGAC---GAATGTGAGAGGCTCAGGAATGAGAATAC...-3′
HQ131681  5′-...TGATTCCTGAAAGGAATGAACAAGGGCAGAC---GAATGTGAGAGGCTCAGGAATGAGAATAC...-3′
AB636521  5′-...TGATCCCTGAAAGGAATGAACAAGGACAGAC---GAATGTGAGAGGCTCAGGAATGAGAATAC...-3′
JQ710454  5′-...TGATTCCTGAAAGGAATGAACAAGGGCAGAC---GAATGTGAGAGGCTCAGGAATGAGAATAC...-3′
AB617576  5′-...TGATTCCTGAAAGGAATGAACAAGGGCAGAC---GAATGTGAGAGGCTCAGGAATGAGAATAC...-3′
JQ710446  5′-...TGATTCCTGGAAGGAATGAACAAGGGCAGAC---GAATGTGAGAGGCTCAGGAATGAGAATAC...-3′
AB741555  5′-...TGATTCCTGAGAGGAATGAACAAGGGCAGAC---GAATGTGAGGGGCTCAGGAATGAGAATAC...-3′
AB629710  5′-...TGATTCCTGAAAGGAATGAACAAGGGCAGAC---GAATGTGAGAGGCTCAGGAATGAGAATAC...-3′
AB760260  5′-...TGATTCCTGAAAGGAATGAACAAGGGCAGAC---GAATGTGAGAGGCTCAGGAATGAGAATAC...-3′
AB760268  5′-...TGATTCCTGAAAGGAATGAACAAGGGCAGAC---GAATGTGAGAGGCTCAGGAATGAGAATAC...-3′
AB760252  5′-...TGATTCCTGAAAGGAATGAACAAGGGCAGAC---GAATGTGAGAGGCTCAGGAATGAGAATAC...-3′
AB760236  5′-...TGATTCCTGAAAGGAATGAACAAGGGCAGAC---GAATGTGAGAGGCTCAGGAATGAGAATAC...-3′
AB615234  5′-...TGATTCCTGAAAGGAATGAACAAGGGCAGAC---GAATGTGAGAGGCTCAGGAATGAGAATAC...-3′
AB742252  5′-...TGATTCCTGAAAGGAATGAACAAGGGCAGAC---GAATGTGAGGGGCTCAGGAATGAGAATAC...-3′
AB742260  5′-...TGATTCCTGAAAGGAATGAACAAGGGCAGAC---GAATGTGAGGGGCTCAGGAATGAGAATAC...-3′
AB742268  5′-...TGATTCCTGAAAGGAATGAACAAGGGCAGAC---GAATGTGAGGGGCTCAGGAATGAGAATAC...-3′
AB530989  5′-...TGATTCCTGAAAGGAATGAACAAGGGCAGAC---GAATGTGAGAGGCTCAGGAATGAGAATAC...-3′
AB612898  5′-...TGATTCCTGAAAGGAACGAACAAGGGCAGAC---GAATGTGAGAGGCTCAGGAATGAGAGTAC...-3′
AB612906  5′-...TGATTCCTGAAAGGAACGAACAAGGGCAGAC---GAATGTGAGAGGCTCAGGAATGAGAGTAC...-3′
AB742276  5′-...TGATTCCTGAAAGGAATGAACAAGGGCAGAC---GAATGTGAGGGGCTCAGGAATGAGAATAC...-3′
CY110735  5′-...TGATTCCTGAAAGGAATGAACAAGGGCAGAC---GAATGTGAGAGGCTCAGGAATGAGAATAC...-3′
AB742292  5′-...TGATTCCTGAAAGGAATGAACAAGGGCAGAC---GAATGTGAGGGGCTCAGGAATGAGAATAC...-3′
AB760244  5′-...TGATTCCTGAAAGGAATGAACAAGGGCAGAC---GAATGTGAGAGGCTCAGGAATGAGAATAC...-3′
AB742284  5′-...TGATTCCTGAAAGGAATGAACAAGGGCAGAC---GAATGTGAGGGGCTCAGGAATGAGAATAC...-3′
AB847562  5′-...TGATTCCTGAAAGGAATGAACAAGGGCAGAC---GAATGTGAGAGGCTCAGGAATGAGAATAC...-3′
AB610969  5′-...TGATTCCTGAAAGGAATGAACAAGGGCAGAC---GAATGTGAGAGGCTCAGGAATGAGAATAC...-3′
AB787206  5′-...TGATTCCTGAAAGGAATGAACAAGGGCAGAC---GAATGTGAGGGGCTCAGGAATGAGAATAC...-3′
AB787198  5′-...TGATTCCTGAAAGGAATGAACAAGGGCAGAC---GAATGTGAGGGGCTCAGGAATGAGAATAC...-3′
AB760276  5′-...TGATTCCTGAAAGGAATGAACAAGGGCAGAC---GAATGTGAGAGGCTCAGGAATGAGAATAC...-3′
AB819034  5′-...TGATACCTGAAAGGAATGAACAAGGACAGAC---GAATGTGAGAGGCTCAGGAATGAGAATAC...-3′
AB741547  5′-...TGATTCCTGAAAGGAATGAACAAGGGCAGAC---GAATGTGAGGGGCTCGGGAATGAGAATAC...-3′
AB786697  5′-...TGATTCCTGAAAGGAATGAACAAGGGCAGAC---GAATGTGAGGGGCTCAGGAATGAGAATAC...-3′
AB786673  5′-...TGATTCCTGAAAGGAATGAACAAGGGCAGAC---GAATGTGAGGGGCTCAGGAATGAGAATAC...-3′
AB786681  5′-...TGATTCCTGAAAGGAATGAACAAGGGCAGAC---GAATGTGAGGGGCTCAGGAATGAGAATAC...-3′
AB828357  5′-...TGATTCCTGAAAGGAATGAACAAGGGCAGAC---GAATGTGAGGGGCTCAGGAATGAGAATAC...-3′
AB786689  5′-...TGATTCCTGAAAGGAATGAACAAGGGCAGAC---GAATGTGAGGGGCTCAGGAATGAGAATAC...-3′
AB807877  5′-...TGATTCCTGAAAGGAATGAACAAGGGCAGAC---GAATGTGAGGGGCTCAGGAATGAGAATAC...-3′
AB824268  5′-...TGATTCCTGAAAGGAATGAACAAGGGCAGAC---GAATGTGAGGGGCTCAGGAATGAGAATAC...-3′
AB824260  5′-...TGATTCCTGAAAGGAATGAACAAGGGCAGAC---GAATGTGAGGGGCTCAGGAATGAGAATAC...-3′
AB807750  5′-...TGATTCCTGAAAGGAATGAACAAGGGCAGAC---GAATGTGAGGGGCTCAGGAATGAGAATAC...-3′
AB741563  5′-...TGATTCCTGAAAGGAATGAACAAGGGCAGAC---GAATGTGAGGGGATCAGGAATGAGAATAT...-3′
AB823752  5′-...TGATCCCTGAAAGGAATGAACAAGGACAGAC---GAATGTGAGGGGCTCAGGAATGAGAATAC...-3′
AB822987  5′-...TGATCCCTGAAAGGAATGAACAAGGACAGAC---GAATGTGAGGGGCTCAGGAATGAGAATAC...-3′
AB823744  5′-...TGATCCCTGAAAGGAATGAACAAGGACAGAC---GAATGTGAGGGGCTCAGGAATGAGAATAC...-3′
AB829734  5′-...TGATTCCTGAAAGGAATGAACAAGGGCAGAC---GAATGTGAGGGGCTCAGGAATGAGAATAC...-3′
AB829742  5′-...TGATTCCTGAAAGGAATGAACAAGGGCAGAC---GAATGTGAGGGGCTCAGGAATGAGAATAC...-3′
AB818502  5′-...TGATCCCTGAAAGGAATGAACAAGGACAGAC---GAATGTGAGGGGCTCAGGAATGAGAATAC...-3′
AB818494  5′-...TGATCCCTGAAAGGAATGAACAAGGACAGAC---GAATGTGAGGGGCTCAGGAATGAGAATAC...-3′
AB849457  5′-...TGATTCCTGAAAGGAATGAACAAGGGCAGAC---AAATGTGAGGGGCTCAGGAATGAGAATAC...-3′
AB824276  5′-...TGATTCCTGAAAGGAATGAACAAGGGCAGAC---GAATGTGAGGGGGTCAGGAATGAGAATAC...-3′
AB828586  5′-...TGATTCCTGAAAGGAATGAACAAGGGCAGAC---GAATGTGAGGGGGTCAGGAATGAGAATAC...-3′
AB828690  5′-...TGATTCCTGAAAGGAATGAACAAGGGCAGAC---GAATGTGAGGGGGTCAGGAATGAGAATAC...-3′
AB828094  5′-...TGATTCCTGAAAGGAATGAACAAGGGCAGAC---GAATGTGAGAGGCTCAGGAATGAGAATAC...-3′
AB827988  5′-...TGATTCCTGAAAGGAATGAACAAGGGCAGAC---GAATGTGAGAGGCTCAGGAATGAGAATAC...-3′
DQ989958  5′-...TGATTCCTGAAAGGAATGAACAAGGGCAGAC---GAATGTGAGAGGCTCAGGAATGAGAATAC...-3′
EF112200  5′-...TGATTCCTGAAAGGAATGAACAAGGGCAGAC---GAATGTGAGAGGCTCAGGAATGAGAATAC...-3′
EF112199  5′-...TGATTCCTGAAAGGAATGAACAAGGGCAGAC---GAATGTGAGAGGCTCAGGAATGAGAATAC...-3′
HQ025946  5′-...TGATTCCTGAAAGGAATGAACAAGGGCAGAC---GAATGTGAGAGGCTCAGGAATGAGAATAC...-3′
EF112211  5′-...TGATTCCTGAAAGGAATGAACAAGGGCAGAC---GAATGTGAGAGGCTCAGGAATGAGAATAC...-3′
CY029953  5′-...TGATTCCTGAAAGGAATGAACAAGGACAGAC---GAATGTGAGAGGCTCAGGAATGAGAATAC...-3′
FM164850  5′-...TGATTCCTGAAAGGAATGAACAGGGACAGAC---GAATGTGAGAGGCTCAGGAATGAGAATAC...-3′
EF112222  5′-...TGATTCCTGAAAGGAATGAACAAGGGCAGAC---GAATGTGAGAGGCTCAGGAATGAGAATAC...-3′
GU052501  5′-...TGATTCCTGAAAGGAATGAACAAGGGCAGAC---GAATGTGAGAGGCTCAGGAATGAGAATAC...-3′
GU052464  5′-...TGATTCCTGAAAGGAATGAACAAGGGCAGAC---GAATGTGAGAGGCTCAGGAATGAGAATAC...-3′
EU147290  5′-...TGATTCCTGCAAGGAATGAACAAGGACAGAC---GAATGTGAGAGGCTCAGGAATGAGAATAC...-3′
DQ232608  5′-...TGATTCCTGCAAGGAATGAACAAGGACAGAC---GAATGTGAGAGGCTCAGGAATGAGAATAC...-3′
GU186715  5′-...TGGTTCCTGAAAGAAATGAACAAGGGCAGAC---GAATGTGAGAGGCTCAGGAATGAGAATAC...-3′
GU186655  5′-...TGATTCCTGAAAGGAATGAACAAGGGCAGAC---GAATGTGAGAGGCTCAGGAATGAGAATAC...-3′
DQ358750  5′-...TGATTCCTGAAAGGAATGAACAGGGACAGAC---GAATGTGAGAGGCTCAGGAATGAGAATAC...-3′
```

```
                   224        235  240           254  1941          1956  1961     1969
                    *          *    *             *    *              *     *        *
DQ852607  5'-...TGATTCCTGAAAGGAATGAACAAGGACAGAC---GAATGTGAGAGGCTCAGGAATGAGAATAC...-3'
DQ863510  5'-...TGATTCCTGAAAGGAATGAACAAGGACAGAC---GAATGTGAGAGGCTCAGGAATGAGAATAC...-3'
GU186707  5'-...TGATTCCTGAAAGGAATGAACAAGGACAGAC---GAATGTTAGAGGCTCAGGAATGAGAATAT...-3'
DQ449647  5'-...TGATTCCTGAAAGGAATGAACAAGGACAGAC---GAATGTAAGGGGCTCAGGAATGAGAATAC...-3'
DQ914807  5'-...TGATTCCTGAAAGGAATGAACAAGGACAGAC---GAATGTAAGAGGCTCAGGAATGAGAATAC...-3'
GU186739  5'-...TGATTCCTGAAAGGAATGAACAAGGGCAGAC---GAATGTGAGAGGCTCAGGAATGAGAATAC...-3'
GU052509  5'-...TGATTCCTGAAAGGAATGAACAAGGGCAGAC---GAATGTGAGAGGCTCAGGAATGAGAATAC...-3'
EU930939  5'-...TGATTCCTGAAAGGAATGAACAAGGGCAGAC---GAATGTGAGAGGCTCAGGAATGAGAATAC...-3'
DQ989974  5'-...TGATTCCTGAAAGGAATGAACAAGGGCAGAC---GAATGTGAGAGGCTCAGGAATGAGAATAC...-3'
GU052112  5'-...TGATTCCTGAAAGGAATGAACAAGGGCAGAC---GAATGTGAGAGGCTCAGGAATGAGAATAC...-3'
GU052120  5'-...TGATTCCTGAAAGGAATGAACAAGGGCAGAC---GAATGTGAGAGGCTCAGGAATGAGAATAC...-3'
GU052072  5'-...TGATTCCTGAAAGGAATGAACAAGGGCAGAC---GAATGTGAGAGGCTCAGGAATGAGAATAC...-3'
GU052080  5'-...TGATTCCTGAAAGGAATGAACAAGGGCAGAC---GAATGTGAGAGGCTCAGGAATGAGAATAC...-3'
GU186699  5'-...TGATTCCTGAAAGGAATGAACAAGGGCAGAC---GAATGTGAGAGGCTCAGGAATGAGAATAC...-3'
GU186691  5'-...TGATTCCTGAAAGGAATGAACAAGGGCAGAC---GAATGTGAGAGGCTCAGGAATGAGAATAC...-3'
GU052493  5'-...TGATTCCTGAAAGGAATGAACAAGGGCAGAC---GAATGTGAGAGGCTCAGGAATGAGAATAC...-3'
DQ399543  5'-...TGATTCCTGAAAGGAATGAACAGGGACAGAC---GAATGTAAGGGGCTCAGGAATGAGAATAC...-3'
DQ386305  5'-...TGATTCCTGAAAGGAATGAACAGGGACAGAC---GAATGTAAGGGGCTCAGGAATGAGAATAC...-3'
DQ363916  5'-...TGATTCCTGAAAGGAATGAACAGGGACAGAC---GAATGTAAGGGGCTCAGGAATGAGAATAC...-3'
GU052472  5'-...TGATTCCTGAAAGGAATGAACAAGGGCAGAC---GAATGTGAGAGGCTCAGGAATGAGAATAC...-3'
DQ232607  5'-...TGATTCCTGAAAGGAATGAACAAGGACAGAC---GAATGTGAGAGGCTCAGGAATGAGAATAC...-3'
DQ365001  5'-...TGATTCCTGAAAGGAATGAACAGGGACAGAC---GAATGTAAGGGGCTCAGGAATGAGAATAC...-3'
DQ365011  5'-...TGATTCCTGAAAGGAATGAACAGGGACAGAC---GAATGTAAGGGGCTCAGGAATGAGAATAC...-3'
DQ343506  5'-...TGATTCCTGAAAGGAATGAACAGGGACAGAC---GAATGTAAGGGGCTCAGGAATGAGAATAC...-3'
DQ434890  5'-...TGATTCCTGAAAGGAATGAACAGGGACAGAC---GAATGTAAGGGGCTCAGGAATGAGAATAC...-3'
DQ389161  5'-...TGATTCCTGAAAGGAATGAACAGGGACAGAC---GAATGTAAGGGGCTCAGGAATGAGAATAC...-3'
DQ363921  5'-...TGATTCCTGAAAGGAATGAACAGGGACAGAC---GAATGTAAGGGGCTCAGGAATGAGAATAC...-3'
GU050332  5'-...TGATCCCTGAAAGGAATGAACAAGGGCAGAC---GAATGTGAGAGGCTCAGGAATGAGGATAC...-3'
DQ449639  5'-...TGATTCCTGAAAGGAATGAACAAGGACAGAC---GAATGTAAGGGGCTCAGGAATGAGAATAC...-3'
GU050324  5'-...TGATCCCTGAAAGGAATGAACAAGGGCAGAC---GAATGTGAGAGGCTCAGGAATGAGGATAC...-3'
GU186663  5'-...TGATTCCTGAAAGGAATGAACAAGGGCAGAC---GAATGTGAGAGGCTCAGGAATGAGAATAC...-3'
GU050348  5'-...TGATCCCTGAAAGGAATGAACAAGGGCAGAC---GAATGTGAGAGGCTCAGGAATGAGGATAC...-3'
GU050340  5'-...TGATCCCTGAAAGGAATGAACAAGGGCAGAC---GAATGTGAGAGGCTCAGGAATGAGGATAC...-3'
GU050380  5'-...TGATTCCTGAAAGGAATGAACAAGGGCAGAC---GAATGTGAGAGGCTCAGGAATGAGAATAC...-3'
GU052417  5'-...TGATTCCTGAAAGGAATGAACAAGGGCAGAC---GAATGTGAGAGGCTCTGGAATGAGAATAC...-3'
EU163436  5'-...TGATTCCTGAAAGGAATGAACAAGGACAGAC---GAATGTGAGAGGCTCAGGAATGAGAATAC...-3'
GU050435  5'-...TGATCCCTGAAAGGAATGAACAAGGGCAGAC---GAATGTGAGAGGCTCAGGAATGAGGATAC...-3'
GU052432  5'-...TGATTCCTGAAAGGAATGAACAAGGGCAGAC---GAATGTGAGAGGCTCTGGAATGAGAATAC...-3'
EF474443  5'-...TGATTCCTGAAAGGAATGAACAAGGACAGAC---GAATGTGAGAGGCTCAGGAATGAGAATAC...-3'
GU050506  5'-...TGATCCCTGAAAGGAATGAACAAGGGCAGAC---GAATGTGAGAGGCTCAGGAATGAGGATAC...-3'
GU050458  5'-...TGATCCCTGAAAGGAATGAACAAGGGCAGAC---GAATGTGAGAGGCTCAGGAATGAGGATAC...-3'
GU050474  5'-...TGATCCCTGAAAGGAATGAACAAGGGCAGAC---GAATGTGAGAGGCTCAGGAATGAGGATAC...-3'
EU257707  5'-...TGATTCCTGAAAGGAATGAACAAGGACAGAC---GAATGTGAGAGGCTCAGGAATGAGAATAC...-3'
GU050388  5'-...TGATTCCTGAAAGGAATGAACAAGGGCAGAC---GAATGTGAGAGGCTCAGGAATGAGAATAC...-3'
GU050546  5'-...TGATCCCTGAAAGGAATGAACAAGGGCAGAC---GAATGTGAGAGGCTCAGGAATGAGGATAC...-3'
GU052402  5'-...TGATTCCTGAAAGGAATGAACAAGGGCAGAC---GAATGTGAGAGGCTCAGGAATGAGAATAC...-3'
GU050404  5'-...TGATTCCTGAAAGGAATGAACAAGGGCAGAC---GAATGTGAGAGGCTCAGGAATGAGAATAC...-3'
GU052540  5'-...TGATCCCTGAAAGGAATGAACAAGGGCAGAC---GAATGTGAGAGGCTCAGGAATGAGGATAC...-3'
GU050442  5'-...TGATCCCTGAAAGGAATGAACAAGGGCAGAC---GAATGTGAGAGGCTCAGGAATGAGGATAC...-3'
GU050372  5'-...TGATTCCTGAAAGGAATGAACAAGGGCAGAC---GAATGTGAGAGGCTCAGGAATGAGAATAC...-3'
GU186776  5'-...TGATACCTGAAAGGAATGAACAAGGGCAGAC---GAATGTGAGAGGCTCAGGAATGAGAATAC...-3'
GU050420  5'-...TGATCCCTGAAAGGAATGAACAAGGGCAGAC---GAATGTGAGAGGCTCAGGAATGAGGATAC...-3'
GU050530  5'-...TGATCCCTGAAAGGAATGAACAAGGGCAGAC---GAATGTGAGAGGCTCAGGAATGAGGATAC...-3'
GU050538  5'-...TGATCCCTGAAAGGAATGAACAAGGGCAGAC---GAATGTGAGAGGCTCAGGAATGAGGATAC...-3'
GU050466  5'-...TGATCCCTGAAAGGAATGAACAAGGGCAGAC---AAATGTGAGAGGCTCAGGAATGAGGATAC...-3'
GU050498  5'-...TGATCCCTGAAAGGAATGAACACGGGCAGAC---GAATGTGAGAGGCTCAGGAATGAGGATAC...-3'
GU050356  5'-...TGATTCCTGAAAGGAATGAAAAAGGGCAGAC---GAATGTGAGAGGCTCAGGAATGAGAATAC...-3'
EU486848  5'-...TGATTCCTGAAAGGAATGAACGAGGACAGAC---GAATGTGAGAGGCTCAGGAATGAGAATAC...-3'
EU441922  5'-...TGATTCCTGAAAGGAATGAACGAGGACAGAC---GAATGTGAGAGGCTCAGGAATGAGAATAC...-3'
EU441930  5'-...TGATTCCTGAAAGGAATGAACGAGGACAGAC---GAATGTGAGAGGCTCAGGAATGAGAATAC...-3'
GU050490  5'-...TGATCCCTGAAAGGAATGAACAAGGGCAGAC---GAATGTGAGAGGCTCAGGAATGAGGATAC...-3'
GU186723  5'-...TGATTCCTGAAAGGAATGAACAAGGGCAGAC---GAATGTGAGAGGCTCAGGAATGAGAATAC...-3'
EU814510  5'-...TGATTCCTGAAAGGAATGAACGAGGACAGAC---GAATGTGAGAGGCTCAGGAATGAGAATAC...-3'
EU414265  5'-...TGATTCCTGAAAGGAATGAACGAGGACAGAC---GAATGTGAGAGGCTCAGGAATGAGAATAC...-3'
GU050514  5'-...TGATCCCTGAAAGGAATGAACAAGGGCAGAC---AAATGTGAGAGGCTCAGGAATGAGGATAC...-3'
GU050522  5'-...TGATCCCTGAAAGGAATGAACAAGGGCAGAC---AAATGTGAGAGGCTCAGGAATGAGGATAC...-3'
GU050427  5'-...TGATCCCTGAAAGGAATGAACAAGGGCAGAC---GAATGTGAGAGGCTCAGGAATGAGGATAC...-3'
GU050482  5'-...TGATCCCTGAAAGGAATGAACAAGGGCAGAC---GAATGTAAGAGGCTCAGGAATGAGGATAC...-3'
GU050396  5'-...TGATTCCTGAAAGGAATGAAAAAGGGCAGAC---GAATGTGAGAGGCTCAGGAATGAGAATAC...-3'
GU186744  5'-...TGATTCCTGAAAGGAATGAACAAGGGCAGAC---GAATGTGAGAGGCTCTGGAATGAGAATAC...-3'
```

```
               224        235  240           254 1941           1956 1961    1969
                *          *    *             *   *              *    *       *
GU052165  5’-...TGATTCCTGAAAGGAATGAACAAGGGCAGAC---GAATGTGAGAGGCTCTGGAATGAGAATAC...-3’
GU052157  5’-...TGATTCCTGAAAGGAATGAACAAGGGCAGAC---GAATGTGAGAGGCTCTGGAATGAGAATAC...-3’
GU052448  5’-...TGATTCCTGAAAGGAATGAACAAGGGCAGAC---GAATGTGAGAGGCTCAGGAATGAGAATAC...-3’
GU186679  5’-...TGATCCCTGAAAGGAATGAACAAGGGCAGAC---GAATGTGAGAGGCTCAGGAATGAGAATAC...-3’
GU186752  5’-...TGATCCCTGAAAGGAATGAACAAGGGCAGAC---GAATGTGAGAGGCTCAGGAATGAGAATAC...-3’
GU050412  5’-...TGATCCCTGAAAGGAATGAACAAGGGCAGAC---AAATGTGAGAGGCTCAGGAATGAGGATAC...-3’
GU050364  5’-...TGATCCCTGAAAGGAATGAACAAGGGCAGAC---GAATGTGAGAGGCTCAGGAATGAGAATAC...-3’
CY126199  5’-...TGATTCCTGAAAGAAATGAACAAGGACAGAC---GAATGTTAGAGGCTCAGGAATGAGAATAC...-3’
GU052532  5’-...TGATCCCTGAAAGGAATGAACAAGGGCAGAC---GAATGTGAGAGGCTCAGGAATGAGAATAC...-3’
GU186768  5’-...TGATCCCTGAAAGGAATGAACAAGGGCAGAC---GAATGTGAGAGGCTCAGGAATGAGAATAC...-3’
GU186760  5’-...TGATCCCTGAAAGGAATGAACAAGGGCAGAC---GAATGTGAGAGGCTCAGGAATGAGAATAC...-3’
CY126159  5’-...TGATTCCTGAAAGAAATGAACAAGGACAGAC---GAATGTTAGAGGCTCAGGAATGAGAATAC...-3’
EF112205  5’-...TGATTCCTGAAAGGAATGAACAAGGGCAGAC---GAATGTGAGAGGCTCAGGAATGAGAATAC...-3’
CY126183  5’-...TGATTCCTGAAAGAAATGAACAAGGACAGAC---GAATGTTAGAGGCTCAGGAATGAGAATAC...-3’
CY126263  5’-...TGATTCCTGAAAGAAATGAACAAGGACAGAC---GAATGTTAGAGGCTCAGGAATGAGAATAC...-3’
CY126072  5’-...TGATTCCTGAAAGAAATGAACAAGGACAGAC---GAATGTTAGAGGCTCAGGAATGAGAATAC...-3’
CY126135  5’-...TGATTCCTGAAAGAAATGAACAAGGACAGAC---GAATGTTAGAGGCTCAGGAATGAGAATAC...-3’
DQ840532  5’-...TGATTCCTGAAAGGAATGAACAGGGACAGAC---GAATGTAAGGGGCTCAGGAATGAGAATAC...-3’
CY126207  5’-...TGATTCCTGAAAGAAATGAACAAGGACAGAC---GAATGTTAGAGGCTCAGGAATGAGAATAC...-3’
CY126143  5’-...TGATTCCTGAAAGAAATGAACAAGGACAGAC---GAATGTTAGAGGCTCAGGAATGAGAATAC...-3’
CY126167  5’-...TGATTCCTGAAAGAAATGAACAAGGACAGAC---GAATGTTAGAGGCTCAGGAATGAGAATAC...-3’
CY126191  5’-...TGATTCCTGAAAGAAATGAACAAGGACAGAC---GAATGTTAGAGGCTCAGGAATGAGAATAC...-3’
CY126033  5’-...TGATTCCTGAAAGAAATGAACAAGGACAGAC---GAATGTTAGAGGCTCAGGAATGAGAATAC...-3’
CY126111  5’-...TGATTCCTGAAAGAAATGAACAAGGACAGAC---GAATGTTAGAGGCTCAGGAATGAGAATAC...-3’
CY126080  5’-...TGATTCCTGAAAGAAATGAACAAGGACAGAC---GAATGTTAGAGGCTCAGGGATGAGAATAC...-3’
CY126025  5’-...TGATTCCTGAAAGAAATGAACAAGGACAGAC---GAATGTTAGAGGCTCAGGGATGAGAATAC...-3’
CY126175  5’-...TGATTCCTGAAAGAAATGAACAAGGACAGAC---GAATGTTAGAGGCTCAGGGATGAGAATAC...-3’
CY126119  5’-...TGATTCCTGAAAGAAATGAACAAGGACAGAC---GAATGTTAGAGGCTCAGGGATGAGAATAC...-3’
CY126215  5’-...TGATTCCTGAAAGAAATGAACAAGGACAGAC---GAATGTTAGAGGCTCAGGAATGAGAATAC...-3’
CY126231  5’-...TGATTCCTGAAAGAAATGAACAAGGACAGAC---GAATGTTAGAGGCTCAGGAATGAGAATAC...-3’
CY125992  5’-...TGATTCCTGAAAGAAATGAACAAGGACAGAC---GAATGTTAGAGGCTCAGGAATGAGAATAC...-3’
CY126247  5’-...TGATTCCTGAAAGAAATGAACAAGGACAAAC---GAATGTTAGAGGCTCAGGAATGAGAATAC...-3’
EU672455  5’-...TGATTCCTGAAAGGAATGAACAAGGGCAGAC---GAATGTAAGAGGCTCAGGAATGAGAATAC...-3’
CY126151  5’-...TGATTCCTGAAAGAAATGAACAAGGACAAAC---GAATGTTAGAGGCTCAGGAATGAGAATAC...-3’
CY126271  5’-...TGATTCCTGAAAGAAATGAACAAGGACAAAC---GAATGTTAGAGGCTCAGGAATGAGAATAC...-3’
CY126127  5’-...TGATTCCTGAAAGAAATGAACAAGGACAGAC---GAATGTTAGAGGCTCAGGAATGAGAATAC...-3’
CY126255  5’-...TGATTCCTGAAAGAAATGAACAAGGACAAAC---GAATGTTAGAGGCTCAGGAATGAGAATAC...-3’
GQ386154  5’-...TGATTCCTGAAAGAAATGAACAAGGGCAGAC---GAATGTGAGAGGCTCAGGAATGAGAATAC...-3’
GQ386146  5’-...TGATTCCTGAAAGGAATGAACAAGGGCAGAC---GAATGTGAGAGGCTCAGGAATGAGAATAC...-3’
HQ630841  5’-...TGATTCCTGAAAGGAATGAACAAGGGCAGAC---GAATGTGAGAGGCTCAGGAATGAGAATAC...-3’
CY125975  5’-...TGATTCCTGAAAGAAATGAACAAGGACAGAC---GAATGTTAGAGGCTCAGGAATGAGAATAC...-3’
CY126103  5’-...TGATTCCTGAAAGAAATGAACAAGGACAGAC---GAATGTTAGAGGCTCAGGAATGAGAATAC...-3’
CY125968  5’-...TGATTCCTGAAAGAAATGAACAAGGACAGAC---GAATGTTAGAGGCTCAGGAATGAGAATAC...-3’
FJ434380  5’-...TGATTCCTGAAAGGAATGAACAAGGGCAGAC---GAATGTGAGAGGCTCAGGAATGAGAATAC...-3’
GU052026  5’-...TGATTCCTGAAAGGAATGAACAAGGACAAAC---GAATGTGAGAGGCTCAGGAATGAGAATAC...-3’
GU052056  5’-...TGATTCCTGAAAGGAATGAACAAGGACAAAC---GAATGTGAGAGGCTCAGGAATGAGAATAC...-3’
GU052064  5’-...TGATTCCTGAAAGGAATGAACAAGGACAAAC---GAATGTGAGAGGCTCAGGAATGAGAATAC...-3’
GU052034  5’-...TGATTCCTGAAAGGAATGAACAAGGACAAAC---GAATGTGAGAGGCTCAGGAATGAGAATAC...-3’
GU052049  5’-...TGATTCCTGAAAGGAATGAACAAGGACAAAC---GAATGTGAGAGGCTCAGGAATGAGAATAC...-3’
GU052525  5’-...TGATTCCTGAAAGAAATGAACAAGGGCAGGC---GAATGTAAGAGGCTCAGGAATGAGAATAC...-3’
GU050450  5’-...TGATCCCTGAACGGAATGAACAGGGACAAAC---GAATGTGAGAGGTTCAGGAATGAGAATAC...-3’
EU930931  5’-...TGATCCCTGAACGGAATGAACAGGGACAAAC---GAATGTGAGAGGTTCAGGAATGAGAATAC...-3’
GU186731  5’-...TGATCCCTGAACGGAATGAACAGGGACAAAC---GAATGTGAGAGGTTCAGGAATGAGAATAC...-3’
GU052479  5’-...TGATTCCTGAAAGAAATGAGCAAGGCCAAAC---GAATGTGAGGGGCTCAGGAATGAGAATAC...-3’
EU636689  5’-...TGATCCCTGAAAGAAATGAGCAAGGTCAAAC---GAACGTGAGGGGTTCAGGAATGAGAATAC...-3’
GU052126  5’-...TGATCCCTGAAAGGAATGAGCAAGGTCAAAC---GAATGTGAGGGGATCAGGAATGAGAATAC...-3’
GU052141  5’-...TGATCCCTGAAAGGAATGAGCAAGGTCAAAC---GAATGTGAGGGGATCAGGAATGAGAATAC...-3’
GU052517  5’-...TGATCCCTGAAAGAAATGAGCAAGGTCAAAC---GAACGTGAGGGGTTCAGGAATGAGAATAC...-3’
GU052010  5’-...TGATTCCTGAGAGGAATGAACAAGGACAAAC---GAATGTAAGAGGATCAGGAATGAGGATAC...-3’
GU186792  5’-...TGATTCCTGAAAGAAATGAACAGGGACAAAC---GAATGTAAGGGGATCAGGAATGAGAATAC...-3’
GU186538  5’-...TGATTCCTGAAAGAAATGAACAGGGACAAAC---GAATGTAAGGGGATCAGGAATGAGAATAC...-3’
GU186516  5’-...TGATTCCTGAAAGAAATGAACAGGGACAAAC---AAATGTAAGGGGATCAGGAATGAGAATAC...-3’
CY097357  5’-...TGATTCCTGAAAGGAATGAACAGGGACAAAC---AAATGTGAGAGGATCAGGAATGAGAATAC...-3’
GU051963  5’-...TGATTCCTGAAAGAAATGAACAGGGACAAAC---AAATGTAAGGGGATCAGGAATGAGAATAC...-3’
GU186524  5’-...TGATTCCTGAAAGAAATGAACAGGGACAAAC---GAATGTAAGGGGATCAGGAATGAGAATAC...-3’
CY144746  5’-...TGATTCCTGAAAGAAATGAACAGGGACAAAC---GAATGTAAGGGGATCAGGAATGAGAATAC...-3’
CY132452  5’-...TGATTCCTGAAAGGAATGAACAGGGACAGAC---GAATGTGAGGGGGTCAGGAATGAGAATAC...-3’
CY144754  5’-...TGATTCCTGAAAGAAATGAACAGGGACAAAC---GAATGTAAGGGGATCAGGAATGAGAATAC...-3’
GU051308  5’-...TGATTCCTGAAAGGAATGAACAGGGACAAAC---GAATGTGAGAGGATCAGGAATGAGAATAC...-3’
GU186530  5’-...TGATTCCAGAAAGAAATGAACAGGGACAAAC---GAATGTAAGGGGGTCAGGAATGAGAATAA...-3’
```

```
               224        235  240          254  1941          1956  1961    1969
                *          *    *             *   *              *    *       *
CY133084  5′-...TGATCCCTGAAAGGAATGAGCAAGGGCAAAC---GAATGTGAGAGGATCAGGAATGAGAATAC...-3′
EU716168  5′-...TGATTCCTGAAAGGAATGAACAAGGGCAGAC---GAATGTGAGAGGCTCAGGAATGAGAATAC...-3′
EF112214  5′-...TGATTCCTGAAAGGAATGAACAAGGGCAGAC---GAATGTGAGAGGCTCAGGAATGAGAATAC...-3′
EF112212  5′-...TGATTCCTGAAAGGAATGAACAAGGGCAGAC---GAATGTGAGAGGCTCAGGAATGAGAATAC...-3′
EU676314  5′-...TGATTCCTGAAAGGAATGAACAAGGGCAGAC---GAATGTGAGAGGCTCAGGAATGAGAATAC...-3′
EU676322  5′-...TGATTCCTGAAAGGAATGAACAAGGGCAGAC---GAATGTGAGAGGCTCAGGAATGAGAATAC...-3′
CY066009  5′-...TGATTCCTGAAAGGAATGAACAAGGACAGAC---GAATGTGAGAGGCTCAGGAATGAGAATAC...-3′
CY066001  5′-...TGATTCCTGAAAGGAATGAACAAGGACAGAC---GAATGTGAGAGGCTCAGGAATGAGAATAC...-3′
CY061299  5′-...TGATTCCTGAAAGGAATGAACAAGGACAGAC---GAATGTGAGAGGCTCAGGAATGAGAATAC...-3′
JN588936  5′-...TGATCCCTGAAAGGAATGAACAAGGACAGAC---GAATGTGAGAGGCTCAGGAATGAGAATAC...-3′
DQ407243  5′-...TGATTCCTGAAAGGAATGAACAAGGGCAGAC---GAATGTGAGAGGCTCAGGAATGAGAATAC...-3′
CY080352  5′-...TGATTCCTGAAAGGAATGAACAGGGACAGAC---GAATGTAAGAGGCTCAGGAATGAGAATAC...-3′
CY037759  5′-...TGATTCCTGAAAGGAATGAACAAGGACAGAC---GAATGTGAGAGGCTCAGGAATGAGAATAC...-3′
GU083606  5′-...TGATTCCTGAAAGGAATGAACAAGGACAGAC---GAATGTGAGAGGCTCAGGAATGAGAATAC...-3′
CY111118  5′-...TGATTCCTGAAAGGAATGAACAAGGACAGAC---GAATGTGAGAGGCTCAGGAATGAGAATAC...-3′
GU354056  5′-...TGATTCCTGAAAGGAATGAACAAGGACAGAC---GAATGTGAGAGGCTCAGGAATGAGAATAC...-3′
FJ784831  5′-...TGATCCCTGAACGGAATGAACAGGGACAAAC---GAATGTGAGAGGTTCAGGAATGAGAATAC...-3′
CY029977  5′-...TGATTCCTGAAAGGAATGAACAAGGACAGAC---GAATGTGAGAGGCTCAGGAATGAGAATAC...-3′
GU724148  5′-...TGATTCCTGAAAGGAATGAACAAGGACAGAC---GAATGTGAGAGGCTCAGGAATGAGAATAC...-3′
CY094822  5′-...TGATTCCTGAAAGGAATGAACAAGGGCAGAC---GAATGTGAGAGGCTCAGGAATGAGAATAC...-3′
CY029961  5′-...TGATTCCTGAAAGGAATGAACAAGGACAGAC---GAATGTGAGAGGCTCAGGAATGAGAATAC...-3′
CY029985  5′-...TGATTCCTGAAAGGAATGAACAAGGACAGAC---GAATGTGAGAGGCTCAGGAATGAGAATAC...-3′
FR687275  5′-...TGATTCCTGAAAGGAATGAACAAGGACAGAC---GAATGTTAGAGGCTCAGGAATGAGAATAC...-3′
CY029969  5′-...TGATTCCTGAAAGGAATGAACAAGGACAGAC---GAATGTGAGAGGCTCAGGAATGAGAATAC...-3′
CY029945  5′-...TGATTCCTGAAAGGAATGAACAAGGACAGAC---GAATGTGAGAGGCTCAGGAATGAGAATAC...-3′
CY029993  5′-...TGATTCCTGAAAGGAATGAACAAGGACAGAC---GAATGTGAGAGGCTCAGGAATGAGAATAC...-3′
GU083650  5′-...TGATTCCTGAAAGGAATGAACAGGGACAGAC---GAATGTGAGAGGCTCAGGAATGAGAATAC...-3′
GU083620  5′-...TGATTCCTGAAAGGAATGAACAAGGACAGAC---GAATGTGAGAGGCTCAGGAATGAGAATAC...-3′
KF881629  5′-...TGATTCCTGAAAGAAATGAACAAGGACAGAC---GAATGTTAGAGGCTCAGGAATGAGAATAC...-3′
KF881528  5′-...TGATTCCTGAAAGAAATGAACAAGGACAGAC---GAATGTTAGAGGCTCAGGAATGAGAATAC...-3′
CY080376  5′-...TGATTCCTGAAAGGAATGAACAAGGACAGAC---GAATGTGAGAGGCTCAGGAATGAGAATAC...-3′
GU252813  5′-...TGATTCCTGAAAGGAATGAACAAGGACAGAC---GAATGTGAGAGGCTCAGGAATGAGAATAC...-3′
KF881435  5′-...TGATTCCTGAAAGAAATGAACAAGGACAGAC---GAATGTTAGAGGCTCAGGAATGAGAATAC...-3′
KF881579  5′-...TGATTCCTGAAAGAAATGAACAAGGACAGAC---GAATGTTAGAGGCTCAGGAATGAGAATAC...-3′
AM262530  5′-...TGATTCCTGAAAGGAATGAACAGGGACAGAC---GAATGTGAGAGGCTCAGGAATGAGAATAC...-3′
AM262533  5′-...TGATTCCTGAAAGGAATGAACAAGGACAGAC---GAATGTTAGAGGCTCAGGAATGAGAATAC...-3′
AM262531  5′-...TGATTCCTGAAAGGAATGAACAAGGACAGAC---GAATGTTAGAGGCTCAGGAATGAGAATAC...-3′
AM262532  5′-...TGATTCCTGAAAGGAATGAACAAGGACAGAC---GAATGTTAGAGGCTCAGGAATGAGAATAC...-3′
AM262534  5′-...TGATTCCTGAAAGGAATGAACAAGGACAGAC---GAATGTTAGAGGCTCAGGAATGAGAATAC...-3′
FM165527  5′-...TGATTCCTGAAAGGAATGAACAAGGACAGAC---GAATGTGAGAGGCTCAGGAATGAGAATAC...-3′
HM627918  5′-...TGATTCCTGAAAGGAATGAACAAGGGCAGAC---GAATGTGAGAGGCTCAGGAATGAGAATAC...-3′
CY063857  5′-...TGATTCCTGAAAGGAATGAACAGGGACAGAC---GAATGTAAGGGGCTCAGGAATGAGAATAC...-3′
EF178510  5′-...TGATTCCTGAAAGGAATGAACAAGGGCAGAC---GAATGTGAGAGGCTCAGGAATGAGAATAC...-3′
JN588933  5′-...TGATCCCTGAAAGGAATGAACAAGGACAGAC---GAATGTGAGAGGCTCAGGAATGAGAATAC...-3′
JN588937  5′-...TGATCCCTGAAAGGAATGAACAAGGACAGAC---GAATGTGAGAGGCTCAGGAATGAGAATAC...-3′
AY950283  5′-...TGATTCCTGAAAGGAATGAACAAGGGCAGAC---GAATGTGAGAGGCTCAGGAATGAGAATAC...-3′
AY950279  5′-...TGATTCCTGAAAGGAATGAACAAGGGCAGAC---GAATGTGAGAGGCTCAGGAATGAGAATAC...-3′
AY950285  5′-...TGATTCCTGAAAGGAATGAACAAGGGCAGAC---GAATGTGAGAGGCTCAGGAATGAGAATAC...-3′
AY770084  5′-...TGATTCCTGAAAGGAATGAACAAGGGCAGAC---GAATGTGAGAGGCTCAGGAATGAGAATAC...-3′
AY950281  5′-...TGATTCCTGAAAGGAATGAACAAGGGCAGAC---GAATGTGAGAGGCTCAGGAATGAGAATAC...-3′
DQ997181  5′-...TGATTCCTGAAAGGAATGAACAAGGGCAGAC---GAATGTGAGAGGCTCAGGAATGAGAATAC...-3′
AY684703  5′-...TGATTCCTGAAAGGAATGAACAAGGGCAGAC---GAATGTGAGAGGCTCAGGAATGAGAATAC...-3′
AY950282  5′-...TGATTCCTGAAAGGAATGAACAAGGGCAGAC---GAATGTGAGAGGCTCAGGAATGAGAATAC...-3′
EF205204  5′-...TGATTCCTGAAAGGAATGAACAAGGACAGAC---GAATGTGAGAGGCTCAGGAATGAGAATAC...-3′
EF205206  5′-...TGATTCCTGAAAGGAATGAACAGGGACAGAC---GAATGTGAGAGGCTCAGGAATGAGAATAC...-3′
JX013487  5′-...TGATTCCTGAAAGGAATGAACAAGGGCAGAC---GAATGTGAGGGGCTCAGGAATGAGAATAC...-3′
DQ997169  5′-...TGGTTCCTGCAAGGAATGTACAAGGGCAGAC---GAATGTGAGAGGCTCAGGAATGAGAATAC...-3′
EF205207  5′-...TGATTCCTGAAAGGAATGAACAAGGACAGAC---GAATGTGAGAGGCTCAGGAATGAGAATAC...-3′
EF205203  5′-...TGATTCCTGAAAGGAATGAACAAGGACAGAC---GAATGTGAGAGGCTCAGGAATGAGAATAC...-3′
DQ864713  5′-...TGATTCCTGAAAGGAATGAACAAGGACAGAC---GAATGTGAGAGGCTCAGGAATGAGAATAC...-3′
EF205205  5′-...TGATTCCTGAAAGAAATGAACAAGGACAGAC---GAATGTGAGAGGCTCAGGAATGAGAATAC...-3′
DQ997178  5′-...TGGTTCCTGAAAGGAATGAACAAGGGCAGAC---GAATGTGAGAGGCTCAGGAATGAGAATAC...-3′
DQ997282  5′-...TGGTTCCTGAAAGGAATGAACAAGGGCAGAT---GAATGTGAGAGGCTCAGGAATGAGAATAC...-3′
DQ997289  5′-...TGGTTCCTGAAAGGAATGAACAAGGGCAGAC---GAATGTGAGAGGCTCAGGAATGAGAATAC...-3′
AY653193  5′-...TGGTTCCTGAAAGGAATGAACAAGGGCAGAC---GAATGTGAGAGGCTCAGGAATGAGAATAC...-3′
EF205209  5′-...TGATTCCTGAAAGGAATGAACAAGGACAGAC---GAATGTGAGAGGCTCAGGAATGAGAATAC...-3′
DQ997416  5′-...TGATTCCTGAAAGGAATGAACAAGGGCAGAC---GAATGTGAGAGGCTCAGGAATGAGAATAC...-3′
DQ997530  5′-...TGATTCCTGAAAGGAATGAACAAGGGCAGAC---GAATGTGAGAGGCTCAGGAATGAGAATAC...-3′
EF362433  5′-...TGATTCCTGAAAGGAATGAACAAGGACAGAC---GAATGTGAGAGGCTCAGGAATGAGAATAC...-3′
EF362425  5′-...TGATTCCTGAAAGGAATGAACAAGGACAGAC---GAATGTGAGAGGCTCAGGAATGAGAATAC...-3′
```

```
                 224          235  240           254  1941            1956  1961     1969
                  *            *    *             *    *               *    *        *
CY037767   5'-...TGATTCCTGAAAGGAATGAACAAGGACAGAC---GAATGTGAGAGGCTCAGGAATGAGAATAC...-3'
HM208709   5'-...TGATTCCTGAAAGGAATGAACAAGGACAGAC---GAATGTGAGAGGCTCAGGAATGAGAATAC...-3'
DQ464357   5'-...TGATTCCTGAAAGGAATGAACAGGGACAGAC---GAATGTGAGAGGCTCAGGAATGAGAATAC...-3'
EF593099   5'-...TGATTCCTGAAAGGAATGAACAAGGGCAGAC---GAATGTGAGAGGCTCAGGAATGAGAATAC...-3'
EF605604   5'-...TGATTCCTGAAAGGAATGAACAAGGACAGAC---GAATGTGAGAGGTTCAGGAATGAGAATAC...-3'
DQ997519   5'-...TGATTCCTGAAAGGAATGAACAAGGGCAGAC---GAATGTGAGAGGCTCAGGAATGAGAATAC...-3'
GU454578   5'-...TGATTCCTGAAAGGAATGAACAAGGACAGAC---GAATGTGAGAGGCTCAGGAATGAGAATAC...-3'
GU354062   5'-...TGATTCCTGAAAGGAATGAACAAGGACAGAC---GAATGTGAGAGGCTCAGGAATGAGAATAC...-3'
EU329189   5'-...TGATTCCTGAAAGGAATGAACAAGGGCAGAC---GAATGTGAGAGGCTCAGGAATGAGAATAC...-3'
EF205208   5'-...TGATTCCTGAAAGGAATGAACAAGGACAGAC---GAATGTGAGAGGCTCAGGAATGAGAATAC...-3'
AY518367   5'-...TGATTCCTGAAAGGAATGAACAAGGGCAGAC---GAATGTGAGAGGCTCTGGAATGAGAATAC...-3'
AY609309   5'-...TGGTTCCTGAAAGGAATGAACAAGGGCAGAC---GAATGTGAGAGGCTCAGGAATGAGAATAC...-3'
AY737301   5'-...TGATTCCTGAAAGGAATGAACAAGGGCAGAC---GAATGTGAGAGGCTCAGGAATGAGAATAC...-3'
DQ997298   5'-...TGGTTCCTGAAAGGAATGAACAAGGGCAGAC---GAATGTGAGAGGCTCAGGAATGAGAATAC...-3'
HM006748   5'-...TGATTCCTGAAAGGAATGAACAAGGGCAGAC---GAATGTGAGAGGCTCAGGAATGAGAATAC...-3'
FM177124   5'-...TGATTCCTGAAAGGAATGAACAAGGACAGAC---GAATGTGAGAGGCTCAGGAATGAGAATAC...-3'
HQ200559   5'-...TGATTCCTGAAAGGAATGAACAAGGGCAGAC---GAATGTGAGAGGCTCAGGAATGAGAATAC...-3'
HQ338098   5'-...TGATTCCTGAAAGGAATGAACAAGGGCAGAC---GAATGTGAGAGGCTCAGGAATGAGAATAC...-3'
FM177132   5'-...TGATTCCTGAAAGGAATGAACAAGGACAGAC---GAATGTGAGAGGCTCAGGAATGAGAATAC...-3'
AB239300   5'-...TGATTCCTGAAAGGAATGAACAAGGACAGAC---GAATGTTAGAGGCTCAGGAATGAGAATAC...-3'
EU402405   5'-...TGATTCCTGAAAGGAATGAACAAGGACAGAC---GAATGTGAGAGGCTCAGGAATGAGAATAC...-3'
GU083628   5'-...TGATTCCTGAAAGGAATGAACAAGGACAGAC---GAATGTGAGAGGCTCAGGAATGAGAATAC...-3'
GU083612   5'-...TGATTCCTGAAAGGAATGAACAAGGACAGAC---GAATGTGAGAGGCTCAGGAATGAGAATAC...-3'
DQ997162   5'-...TGATTCCTGAAAGGAATGAACAAGGGCAGAC---GAATGTGAGAGGCTCAGGAATGAGAATAC...-3'
GU252819   5'-...TGATTCCTGAAAGGAATGAACAAGGACAGAC---GAATGTGAGAGGCTCAGGAATGAGAATAC...-3'
AY737286   5'-...TGATTCCTGAAAGGAATGAACAAGGGCAAAC---GAATGTGAGAGGCTCAGGAATGAGAATAC...-3'
GU252827   5'-...TGATTCCTGAAAGGAATGAACAAGGACAGAC---GAATGTGAGAGGCTCAGGAATGAGAATAC...-3'
CY111102   5'-...TGATTCCTGAAAGGAATGAACAAGGACAGAC---GAATGTGAGAGGCTCAGGAATGAGAATAC...-3'
CY080384   5'-...TGATTCCTGAAAGGAATGAACAAGGACAGAC---GAATGTGAGAGGCTCAGGAATGAGAATAC...-3'
HQ200457   5'-...TGATTCCTGAAAGGAATGAACAAGGGCAGAC---GAATGTGAGAGGCTCAGGAATGAGAATAC...-3'
GU083634   5'-...TGATTCCTGAAAGGAATGAACAAGGACAGAC---GAATGTGAGAGGCTCAGGAATGAGAATAC...-3'
DQ997544   5'-...TGATTCCTGAAAGGAATGAACAAGGGCAGAC---GAATGTGAGAGGCTCAGGAATGAGAATAC...-3'
GU272003   5'-...TGATTCCTGAAAGGAATGAACAGGGACAGAC---GAATGTGAGAGGCTCAGGAATGAGAATAC...-3'
GU083658   5'-...TGATTCCTGAAAGGAATGAACAGGGACAGAC---GAATGTGAGAGGCTCAGGAATGAGAATAC...-3'
DQ997383   5'-...TGGTTCCTGAAAGGAATGAACAAGGGCAGAC---GAATGTGAGAGGCTCAGGAATGAGAATAC...-3'
CY111086   5'-...TGATTCCTGAAAGGAATGAACAAGGACAGAC---GAATGTGAGAGGCTCAGGAATGAGAATAC...-3'
DQ997085   5'-...TGATTCCTGAAAGGAATGAACAAGGGCAGAC---GAATGTGAGAGGATCAGGAATGAGAATAC...-3'
CY111054   5'-...TGATTCCTGAAAGGAATGAACAAGGACAGAC---GAATGTGAGAGGCTCAGGAATGAGAATAC...-3'
GU354078   5'-...TGATTCCTGAAAGGAATGAACAGGGACAGAC---GAATGTAAGAGGCTCAGGAATGAGAATAC...-3'
CY080368   5'-...TGATTCCTGAAAGGAATGAACAGGGACAGAC---GAATGTGAGAGGCTCAGGAATGAGAATAC...-3'
AY737293   5'-...TGATTCCTGAAAGGAATGAACAAGGGCAGAC---GAATGTGAGAGGCTCTGGAATGAGAATAC...-3'
CY035238   5'-...TGATTCCTGAAAGGAATGAACAGGGACAGAC---GAATGTGAGAGGATCAGGAATGAGAATAC...-3'
HM172450   5'-...TGATTCCTGAAAGGAATGAACAAGGGCAGAC---GAATGTGAGAGGCTCAGGAATGAGAATAC...-3'
HQ200585   5'-...TGATTCCTGAAAGGAATGAACAAGGGCAGAC---GAATGTGAGAGGCTCAGGAATGAGAATAC...-3'
FJ966210   5'-...TGATTCCTGAAAGGAATGAACAAGGACAGAC---GAATGTGAGAGGCTCAGGAATGAGAATAC...-3'
CY111070   5'-...TGATTCCTGAAAGGAATGAACAGGGACAGAC---GAATGTGAGAGGCTCAGGAATGAGAATAC...-3'
EU329174   5'-...TGATTCCTGAAAGGAATGAACAAGGGCAGAC---GAATGTGAGAGGCTCAGGAATGAGAATAC...-3'
GU066387   5'-...TGGTTCCTGAAAGGAATGAACAAGGACAGAC---GAATGTGAGAGGCTCAGGAATGAGAATAC...-3'
GU271995   5'-...TGATTCCTGAAAGGAATGAACAAGGACAGAC---GAATGTGAGAGGCTCAGGAATGAGAATAC...-3'
EU594346   5'-...TGATTCCTGAAAGGAATGAACAAGGGCAGGC---GAATGTGAGAGGCTCAGGAATGAGAATAC...-3'
HM172449   5'-...TGATTCCTGAAAGGAATGAACAAGGGCAGAC---GAATGTGAGAGGCTCAGGAATGAGAATAC...-3'
CY111094   5'-...TGATTCCTGAAAGGAATGAACAAGGACAGAC---GAATGTGAGAGGCTCAGGAATGAGAATAC...-3'
GU354070   5'-...TGATTCCTGAAAGGAATGAACAAGGACAGAC---GAATGTGAGAGGCTCAGGAATGAGAATAC...-3'
JX420155   5'-...TGATCCCTGAAAGGAATGAACAAGGGCAGAC---GAATGTGAGAGGCTCAGGAATGAGAACAC...-3'
DQ997270   5'-...TGATTCCTGAAAGGAATGAACAAGGGCAGAC---GAATGTGAGAGGCTCAGGAATGAGAATAC...-3'
CY063470   5'-...TGATTCCTGAAAGGAATGAACAAGGACAGAC---GAATGTGAGAGGCTCAGGAATGAGAATAC...-3'
CY111062   5'-...TGATTCCTGAAAGGAATGAACAAGGACAGAC---GAATGTGAGAGGCTCAGGAATGAGAATAC...-3'
HM172452   5'-...TGATTCCTGAAAGGAATGAACAAGGGCAGAC---GAATGTGAGAGGCTCAGGAATGAGAATAC...-3'
JX420203   5'-...TGATCCCTGAAAGGAATGAACAAGGGCAGAC---GAATGTGAGAGGCTCAGGAATGAGAATAC...-3'
DQ997306   5'-...TGATTCCTGAAAGGAATGAACAAGGGCAGAC---GAATGTGAGAGGCTCAGGAATGAGAATAC...-3'
DQ997404   5'-...TGATTCCTGAAAGGAATGAACAAGGGCAGAC---GAATGTGAGAGGCTCAGGAATGAGAATAC...-3'
DQ997363   5'-...TGGTTCCTGAAAGGAATGAACAAGGGCAGAC---GAATGTGAGAGGATCAGGAATGAGAATAC...-3'
DQ914812   5'-...TGGTTCCTGAAAGGAATGAACAAGGGCAAAC---GAATGTGAGAGGCTCAGGAATGAGAATAC...-3'
FJ654292   5'-...TGATTCCTGAAAGGAATGAACAAGGGCAGAC---GAATGTAAGAGGCTCAGGAATGAGAATAC...-3'
KF881464   5'-...TGATTCCTGAAAGAAATGAACAAGGACAGAC---GAATGTTAGAGGCTCAGGAATGAGAATAC...-3'
GU182155   5'-...TGATTCCTGAAAGGAATGAACAAGGGCAGAC---TAATGTAAGAGGCTCAGGAATGAGAATAC...-3'
KF881359   5'-...TGATTCCTGAAAGAAATGAACAAGGACAGAC---GAATGTTAGAGGCTCAGGAATGAGAATAC...-3'
GU182139   5'-...TGATTCCTGAAAGGAATGAACAAGGGCAGAC---GAATGTAAGAGGCTCAGGAATGAGAATAC...-3'
DQ997372   5'-...TGGTTCCTGAAAGGAATGAACAAGGGCAGAT---GAATGTAAGAGGGTCAGGGATGAGAATAC...-3'
CY098330   5'-...TGATTCCTGAGAGGAATGAACAGGGGCAGAC---GAATGTGAGGGGCTCAGGAATGAGAATAC...-3'
```

```
                   224          235  240          254  1941          1956  1961      1969
                    *            *    *            *    *             *     *         *
JQ277222  5'-...TGGTTCCTGAAAGGAATGAACAAGGGCAAAC---GAATGTGAGAGGCTCAGGAATGAGAATAC...-3'
KF881687  5'-...TGATTCCTGAAAGAAATGAACAAGGACAGAC---GAATGTTAGAGGCTCAGGAATGAGAATAC...-3'
GU182163  5'-...TGATTCCTGAAAGGAATGAACAAGGGCAGAC---GAATGTGAGAGGCTCAGGAATGAGAATAC...-3'
KF881399  5'-...TGATTCCTGAAAGAAATGAACAAGGACAGAC---GAATGTTAGAGGCTCAGGAATGAGAATAC...-3'
KF881413  5'-...TGATTCCTGAAAGAAATGAACAAGGACAGAC---GAATGTTAGAGGCTCAGGAATGAGAATAC...-3'
DQ997546  5'-...TGATTCCTGAAAGGAATGAACAAGGGCAGAC---GAATGTGAGAGGATCAGGAATGAGAATAC...-3'
KF881716  5'-...TGATTCCTGAAAGAAATGAACAAGGACAGAC---GAATGTTAGAGGCTCAGGAATGAGAATAC...-3'
CY098349  5'-...TGATTCCTGAAAGGAATGAACAAGGGCAGAC---GAATGTGAGAGGCTCAGGAATGAGAATAC...-3'
CY098342  5'-...TGATTCCTGAAAGGAATGAACAAGGGCAGAC---GAATGTGAGAGGCTCAGGAATGAGAATAC...-3'
DQ997339  5'-...TGGTTCCTGAAAGGAATGAACAAGGGCAGAT---GAATGTGAGAGGCTCAGGAATGAGAATAC...-3'
KF881488  5'-...TGATTCCTGAAAGAAATGAACAAGGACAGAC---GAATGTTAGAGGCTCAGGAATGAGAATAC...-3'
CY098322  5'-...TGATTCCTGAAAGGAATGAACAAGGGCAGAC---GAATGTGAGAGGCTCAGGAATGAGAATAC...-3'
CY098306  5'-...TGATTCCTGAAAGGAATGAACAAGGGCAGAC---GAATGTGAGAGGCTCAGGAATGAGAATAC...-3'
CY098357  5'-...TGATTCCTGAGAGGAATGAACAGGGGCAGAC---GAATGTGAGGGGCTCAGGAATGAGAATAC...-3'
CY098294  5'-...TGATTCCTGAGAGGAATGAACAGGGGCAGAC---GAATGTGAGGGGCTCAGGAATGAGAATAC...-3'
CY098403  5'-...TGATTCCTGAAAGGAATGAACAAGGGCAGAC---GAATGTGAGAGGCTCAGGAATGAGAATAC...-3'
CY098409  5'-...TGATTCCTGAAAGGAATGAACAAGGGCAGAC---GAATGTGAGAGGCTCAGGAATGAGAATAC...-3'
KF881708  5'-...TGATTCCTGAAAGAAATGAACAAGGACAGAC---GAATGTTAGAGGCTCAGGAATGAGAATAC...-3'
JN588932  5'-...TGATCCCTGAAAGGAATGAACAAGGACAGAC---GAATGTGAGAGGCTCAGGAATGAGAATAC...-3'
CY098433  5'-...TGATTCCTGAAAGGAATGAACAAGGGCAGAC---GAATGTGAGAGGCTCAGGAATGAGAATAC...-3'
CY098387  5'-...TGATTCCTGAAAGGAATGAACAAGGGCAGAC---GAATGTGAGAGGCTCAGGAATGAGAATAC...-3'
CY098371  5'-...TGATTCCTGAAAGGAATGAACAAGGGCAGAC---GAATGTGAGAGGCTCAGGAATGAGAATAC...-3'
JN588934  5'-...TGATCCCTGAAAGGAATGAACAAGGACAGAC---GAATGTGAGAGGCTCAGGAATGAGAATAC...-3'
CY098441  5'-...TGATTCCTGAAAGGAATGAACAAGGGCAGAC---GAATGTGAGAGGCTCAGGAATGAGAATAC...-3'
CY098363  5'-...TGATTCCTGAAAGGAATGAACAAGGGCAGAC---GAATGTGAGAGGCTCAGGAATGAGAATAC...-3'
DQ997093  5'-...TGATTCCTGAAAGGAATGAACAAGGGCAGAC---GAATGTGAGAGGCTCAGGAATGAGAATAC...-3'
AY950284  5'-...TGATTCCTGAAAGGAATGAACAAGGGCAGAC---GAATGTGAGAGGCTCAGGAATGAGAATAC...-3'
DQ997217  5'-...TGATTCCTGAAAGGAATGAACAAGGGCAGAC---GAATGTGAGAGGCTCAGGAATGAGAATAC...-3'
CY098449  5'-...TGATTCCTGAAAGGAATGAACAAGGGCAGAC---GAATGTGAGAGGCTCAGGAATGAGAATAC...-3'
CY063315  5'-...TGATTCCTGAAAGGAATGAACAAGGGCAGAC---GAATGTGAGAGGCTCAGGAATGAGAATAC...-3'
KF881652  5'-...TGATCCCTGAAAGAAATGAACAAGGACAGAC---GAATGTTAGAGGCTCAGGAATGAGAATAC...-3'
FJ868030  5'-...TGATTCCTGAAAGGAATGAACAAGGGCAGAC---GAATGTGAGAGGATCAGGAATGAGAATAC...-3'
KF881421  5'-...TGATCCCTGAAAGAAATGAACAAGGACAGAC---GAATGTTAGAGGCTCAGGAATGAGAATAC...-3'
KF881695  5'-...TGATCCCTGAAAGAAATGAACAAGGACAGAC---GAATGTTAGAGGCTCAGGAATGAGAATAC...-3'
JN588930  5'-...TGATCCCTGAAAGGAATGAACAAGGACAGAC---GAATGTGAGAGGCTCAGGAATGAGAATAC...-3'
KF881660  5'-...TGATCCCTGAAAGAAATGAACAAGGACAGAC---GAATGTTAGAGGCTCAGGAATGAGAATAC...-3'
KF881536  5'-...TGATCCCTGAAAGAAATGAACAAGGACAGAC---GAATGTTAGAGGCTCAGGAATGAGAATAC...-3'
KF881310  5'-...TGATCCCTGAAAGAAATGAACAAGGACAGAC---GAATGTTAGAGGCTCAGGAATGAGAATAC...-3'
KF881496  5'-...TGATCCCTGAAAGAAATGAACAAGGACAGAC---GAATGTTAGAGGCTCAGGAATGAGAATAC...-3'
JX534554  5'-...TGATTCCTGAAAGGAATGAACAAGGGCAGAC---GAATGTGAGAGGCTCAGGAATGAGAATAC...-3'
KF881353  5'-...TGATCCCTGAAAGAAATGAACAAGGACAGAC---GAATGTTAGAGGCTCAGGAATGAGAATAC...-3'
DQ997314  5'-...TGGTTCCTGAAAGGAATGAACAAGGGCAGAC---GAATGTAAGAGGGTCAGGGATGAGAATAC...-3'
KF881522  5'-...TGATCCCTGAAAGAAATGAACAAGGACAGAC---GAATGTTAGAGGCTCAGGAATGAGAATAC...-3'
KF881600  5'-...TGATCCCTGAAAGAAATGAACAAGGACAGAC---GAATGTTAGAGGCTCAGGAATGAGAATAC...-3'
CY098336  5'-...TGATTCCTGAGAGGAATGAACAGGGGCAGAC---GAATGTGAGGGGCTCAGGAATGAGAATAC...-3'
JX534578  5'-...TGATTCCTGAAAGGAATGAACAAGGGCAGAC---AAATGTGAGAGGCTCAGGAATGAGAATAC...-3'
CY098314  5'-...TGATTCCTGAAAGGAATGAACAAGGGCAGAC---GAATGTGAGAGGCTCAGGAATGAGAATAC...-3'
KF881558  5'-...TGATCCCTGAAAGAAATGAACAAGGACAGAC---GAATGTTAGAGGCTCAGGAATGAGAATAC...-3'
CY089418  5'-...TGATTCCTGAAAGGAATGAACAAGGGCAGAC---GAATGTGAGAGGCTCAGGAATGAGAATAC...-3'
JX497773  5'-...TGATTCCTGAAAGGAATGAACAAGGGCAGAC---GAATGTGAGAGGCTCAGGAATGAGAATAC...-3'
JX497765  5'-...TGATTCCTGAAAGGAATGAACAAGGGCAGAC---GAATGTGAGAGGCTCAGGAATGAGAATAC...-3'
JX534594  5'-...TGATTCCTGAAAGGAATGAACAAGGGCAGAC---GAATGTGAGGGGATCAGGAATGAGAATAT...-3'
KF881443  5'-...TGATCCCTGAAAGAAATGAACAAGGACAGAC---GAATGTTAGAGGCTCAGGAATGAGAATAC...-3'
KF881516  5'-...TGATCCCTGAAAGAAATGAACAAGGACAGAC---GAATGTTAGAGGCTCAGGAATGAGAATAC...-3'
CY098379  5'-...TGATTCCTGAAAGGAATGAACAAGGGCAGAC---GAATGTGAGAGGCTCAGGAATGAGAATAC...-3'
DQ997528  5'-...TGATTCCTGAAAGGAATGAACAAGGGCAGAC---GAATGTGAGAGGCTCAGGAATGAGAATAC...-3'
KF881608  5'-...TGATCCCTGAAAGAAATGAACAAGGACAGAC---GAATGTTAGAGGCTCAGGAATGAGAATAC...-3'
KC690153  5'-...TGATCCCTGAAAGGAATGAACAAGGGCAGAC---GAATGTGAGAGGCTCAGGAATGAGAATAC...-3'
JX534570  5'-...TGATTCCTGAAAGGAACGAACAAGGGCAGAC---GAATGTGAGGGGCTCAGGAATGAGAATAC...-3'
KF881543  5'-...TGATCCCTGAAAGAAATGAACAAGGACAGAC---GAATGTTAGAGGCTCAGGAATGAGAATAC...-3'
CY098457  5'-...TGATTCCTGAAAGGAATGAACAAGGGCAGAC---GAATGTGAGAGGCTCAGGAATGAGAATAC...-3'
JX294924  5'-...TGATTCCTGAAAGGAATGAACATGGGCAGAC---GAATGTGAGAGGCTCAGGAATGAGAATAC...-3'
CY092139  5'-...TGATTCCTGAAAGGAATGAACATGGGCAGAC---GAATGTGAGAGGCTCAGGAATGAGAATAC...-3'
KF881428  5'-...TGATCCCTGAAAGAAATGAACAAGGACAGAC---GAATGTTAGAGGCTCAGGAATGAGAATAC...-3'
KC535016  5'-...TGATTCCTGAAAGGAATGAACAAGGGCAGAC---GAATGTGAGGGGCTCAGGAATGAGAATAC...-3'
KF881405  5'-...TGATCCCTGAAAGAAATGAACAAGGACAGAC---GAATGTTAGAGGCTCAGGAATGAGAATAC...-3'
KF881587  5'-...TGATCCCTGAAAGAAATGAACAAGGACAGAC---GAATGTTAGAGGCTCAGGAATGAGAATAC...-3'
CY092121  5'-...TGATTCCTGAAAGGAATGAACATGGGCAGAC---GAATGTGAGAGGCTCAGGAATGAGAATAC...-3'
DQ997323  5'-...TGATTCCTGAAAGGAATGAACAAGGGCAGAC---GAATGTGAGAGGCTCAGGGATGAGAATAC...-3'
DQ997317  5'-...TGATTCCTGAAAGGAATGAACAAGGGCAGAC---GAATGTAAGAGGCTCAGGGATGAGAATAC...-3'
```

```
                224        235  240           254 1941           1956 1961    1969
                 *          *    *             *   *              *    *       *
CY092115   5′-...TGATTCCTGAAAGGAATGAACATGGGCAGAC---GAATGTGAGAGGCTCAGGAATGAGAATAC...-3′
JX534586   5′-...TGATTCCTGAAAGGAATGAACAAGGGCAGAC---GAATGTGAGGGGATCAGGAATGAGAATAT...-3′
CY092127   5′-...TGATTCCTGAAAGGAATGAACATGGGCAGAC---GAATGTGAGAGGCTCAGGAATGAGAATAC...-3′
KF881593   5′-...TGATCCCTGAAAGAAATGAACAAGGACAGAC---GAATGTTAGAGGCTCAGGAATGAGAATAC...-3′
KF881564   5′-...TGATCCCTGAAAGAAATGAACAAGGACAGAC---GAATGTTAGAGGCTCAGGAATGAGAATAC...-3′
KF881346   5′-...TGATCCCTGAAAGAAATGAACAAGGACAGAC---GAATGTTAGAGGCTCAGGAATGAGAATAC...-3′
KF881667   5′-...TGATCCCTGAAAGAAATGAACAAGGACAGAC---GAATGTTAGAGGTTCAGGAATGAGAATAC...-3′
DQ997354   5′-...TGGTTCCTGAAAGGAATGAACAAGGGCAGAT---GAATGTGAGAGGCTCAGGAATGAGAATAC...-3′
KF881622   5′-...TGATCCCTGAAAGAAATGAACAAGGACAGAC---GAATGTTAGAGGTTCAGGAATGAGAATAC...-3′
KF881324   5′-...TGATCCCTGAAAGAAATGAACAAGGACAGAC---GAATGTTAGAGGCTCAGGAATGAGAATAC...-3′
DQ997142   5′-...TGATTCCTGAAAGGAATGAACCAGGGCAGAC---GAATGTGAGAGGCTCAGGAATGAGAATAC...-3′
KF881317   5′-...TGATCCCTGAAAGAAATGAACAAGGACAGAC---GAATGTTAGAGGCTCAGGAATGAGAATAC...-3′
KF881701   5′-...TGATCCCTGAAAGAAATGAACAAGGACAGAC---GAATGTTAGAGGCTCAGGAATGAGAATAC...-3′
DQ997225   5′-...TGGTTCCTGAAAGGAATGAACAAGGGCAGAC---GAATGTGAGAGGCTCAGGAATGAGAATAC...-3′
AY950280   5′-...TGGTTCCTGAAAGGAATGAACAAGGGCAGAC---GAATGTGAGAGGCTCAGGAATGAGAATAC...-3′
GU727666   5′-...TGATTCCTGAAAGGAATGAACAAGGGCAGAC---GAATGTGAGAGGCTCGGGAATGAGAATAC...-3′
JX534546   5′-...TTGTTCCTGAGAGAAATGAACAAGGGCAAAC---GAATGTGAGAGGCTCAGGAATGAGAATAC...-3′
GU727674   5′-...TGATTCCCGAAAGGAATGAACAAGGGCAGAC---GAATGTGAGAGGCTCGGGAATGAGAATAC...-3′
DQ997149   5′-...TGATTCCCGAAAGGAATGAGCAAGGGCAGAC---GAATGTGAGAGGATCAGGAATGAGAATAC...-3′
FR687271   5′-...TGATTCCTGAAAGAAATGAACAAGGACAGAC---GAATGTGAGGGGATCAGGAATGAGAATAC...-3′
JX534562   5′-...TGATTCCTGAAAGAAATGAACAAGGCCAGAC---GAATGTGAGAGGCTCGGGAATGAGAATAC...-3′
AF144300   5′-...TGATTCCTGAAAGGAATGAGCAAGGACAAAC---GAATGTGAGAGGCTCAGGAATGAGAATAC...-3′
EU263350   5′-...TGATTCCTGAAAGGAATGAACAAGGGCAAAC---GAATGTGAGAGGCTCAGGAATGAGAATAC...-3′
EU263342   5′-...TGATTCCTGAAAGGAATGAACAAGGGCAAAC---GAATGTGAGAGGCTCAGGAATGAGAATAC...-3′
DQ366311   5′-...TGATCCCTGAAAGGAATGAGCAAGGTCAAAC---GAATGTGAGGGGATCAGGAATGAGAATAC...-3′
CY015088   5′-...TGATTCCTGAAAGAAATGAACAAGGGCAGGC---GAATGTAAGAGGCTCAGGAATGAGAATAC...-3′
DQ997101   5′-...TGATTCCCGAAAGGAATGAGCAAGGGCAGAC---GAATGTGAGGGGCTCAGGGATGAGAATAC...-3′
DQ997121   5′-...TGATTCCTGAAAGAAATGAACAAGGGCAGAC---GAATGTAAGAGGGTCAGGGATGAGAATAC...-3′
EU871817   5′-...TGATTCCCGAGAGGAATGAACAAGGGCAAAC---GAATGTAAGAGGATCAGGAATGAGGATAC...-3′
FR687270   5′-...TGATTCCGGAGAGAAATGAACAAGGACAAAC---GAATGTGAGAGGATCAGGATTGAGGATAC...-3′
DQ366335   5′-...TGATCCCTGAACGGAATGAACAGGGACAGAC---AAATGTGAGAGGTTCAGGAATGAGAATAC...-3′
DQ366327   5′-...TGATCCCTGAACGGAATGAACAGGGACAGAC---AAATGTGAGAGGTTCAGGAATGAGAATAC...-3′
DQ997110   5′-...TGATCCCTGAAAGAAATGAACAAGGTCAAAC---GAATGTGAGGGGCTCAGGAATGAGAATAC...-3′
DQ997132   5′-...TGATCCCTGAAAGAAATGAGCAAGGTCAAAC---GAATGTGAGGGGCTCAGGAATGAGAATAC...-3′
DQ366303   5′-...TGATCCCTGAAAGGAATGAGCAAGGTCAAAC---GAATGTGAGGGGATCAGGAATGAGAATAC...-3′
DQ366319   5′-...TGATCCCTGAAAGGAATGAGCAAGGTCAAAC---GAATGTGAGGGGATCAGGAATGAGAATAC...-3′
DQ454106   5′-...TGATTCCTGAAAGGAATGAACAAGGGCAGAC---GAATGTGAGAGGATCAGGAATGAGAATAC...-3′
CY061882   5′-...TGATCCCTGAAAGAAATGAGCAAGGTCAGAC---GAACGTAAGGGGTTCAGGAATGAGAATAC...-3′
CY015126   5′-...TGATCCCTGAAAGAAATGAGCAAGGTCAAAC---GAACGTGAGGGGTTCAGGAATGAGAATAC...-3′
GU596981   5′-...TGGTCCCGGAAAGGAATGAGCAAGGTCAGAC---GAACGTGAGGGGTTCAGGAATGAGAATAC...-3′
FJ031998   5′-...TGATTCCTGAAAGAAATGAACAGGGACAAAC---GAATGTAAGGGGATCAGGAATGAGAATAC...-3′
CY014732   5′-...TGATTCCTGAGAGGAATGAACAAGGACAAAC---GAATGTGAGAGGGTCAGGAATGAGGATAC...-3′
EF392844   5′-...TGATTCCTGAAAGAAATGAACAGGGACAAAC---GAATGTAAGGGGATCAGGAATGAGAATAC...-3′
EF210572   5′-...TGATTCCTGAAAGAAATGAACAGGGACAAAC---GAATGTAAGGGGGTCAGGAATGAGAATAC...-3′
EU871943   5′-...TGATTCCTGAAAGGAATGAACAAGGACAGAC---GAATGTGAGAGGCTCAGGAATGAGAATAC...-3′
FJ868022   5′-...TGATTCCTGAAAGGAATGAAAAAGGGCAGAC---GAATGTGAGAGGATCAGGAATGAGAATAC...-3′
EF112202   5′-...TGATTCCTGAAAGGAATGAACAAGGGCAGAC---GAATGTGAGAGGCTCAGGAATGAGAATAC...-3′
AY856861   5′-...TGATTCCTGAAAGGAATGAACAAGGGCAAAC---GAATGTGAGAGGCTCAGGATTGAGAATAC...-3′
```
