# Supplementary material for: Evidence for a Novel Mechanism of Influenza Virus-Induced Type I Interferon Expression by a Defective RNA-Encoded Protein
Source: PLoS Pathog. 2015 May 29;11(5):e1004924. doi: 10.1371/journal.ppat.1004924 (PMC4449196; doi:10.1371/journal.ppat.1004924)
Supplement: S3 Table — MDCKII cells were infected with plaque-purified PB2Δ-free rKAN-1 WT or Vietnam clones for 16–24 h. Undiluted supernatants were transferred to untreated MDCKII cells for 5 passages. Total RNA was isolated from every passage and expressional changes of PB2Δ RNA were analyzed by qRT-PCR and are depicted as n-fold normalized to first passage. (PDF) [file ppat.1004924.s003.pdf]

S3 Table. Expressional changes of PB2$_{\Delta}$ RNA in multiple undiluted passages of plaque purified viral clones. MDCKII cells were infected with plaque-purified PB2$_{\Delta}$-free rKAN-1 WT or Vietnam clones for 16-24 h. Undiluted supernatants were transferred to untreated MDCKII cells for 5 passages. Total RNA was isolated from every passage and expressional changes of PB2$_{\Delta}$ RNA were analyzed by qRT-PCR and are depicted as *n*-fold normalized to first passage.

virus	clone	passage number				
		1	2	3	4	5
rKAN-1 WT	1	1,000	1,409	0,722	0,084	0,036
	2	1,000	10,666	2,049	0,106	0,166
	3	1,000	5,736	2,151	0,401	0,034
	4	1,000	5,502	12,510	0,300	0,516
	5	1,000	3,283	5,560	0,169	0,007
	6	1,000	4,184	1,454	0,094	0,406
	7	1,000	12,338	5,011	0,325	0,082
	8	1,000	67,415	49,522	32,900	1,602
	9	1,000	1,333	5,598	0,690	0,223
	10	1,000	0,771	0,024	0,001	0,000
	11	1,000	5,796	2,049	0,294	0,012
	12	1,000	13,595	2,809	1,214	0,149
Vietnam	1	1,000	0,790	1,444	0,488	0,737
	2	1,000	1,419	0,176	0,008	0,001
	3	1,000	3,618	1,490	0,040	0,104
	4	1,000	0,283	0,042	0,003	0,001
	5	1,000	1,165	0,798	0,040	0,047
	6	1,000	1,510	0,455	0,049	0,087
	7	1,000	2,558	3,238	0,613	2,676
	8	1,000	0,871	0,249	0,059	0,003
	9	1,000	0,529	0,557	0,041	0,005
	10	1,000	0,333	0,083	0,005	0,001
	11	1,000	0,470	0,085	0,003	0,001
	12	1,000	1,693	0,238	0,020	0,010
