# Supplementary material for: Evidence for a Novel Mechanism of Influenza Virus-Induced Type I Interferon Expression by a Defective RNA-Encoded Protein
Source: PLoS Pathog. 2015 May 29;11(5):e1004924. doi: 10.1371/journal.ppat.1004924 (PMC4449196; doi:10.1371/journal.ppat.1004924)
Supplement: S4 Table — (PDF) [file ppat.1004924.s004.pdf]

**S4 Table. List of primers used in the study.**

Primers:

| Gene | Forward 5‘-3‘ | Reverse 5‘-3‘ |
|---|---|---|
| PB2$_{\Delta}$_BsaI | GCATGGTCTCGGGGAGCGAAAGCAGGTCAAATATATTCAATATG | GCACGGTCTCGTATTAGTAGAAACAAGGTCGTTTTTAAATAATTCGACACTA |
| Myc_PB2_ EcoRV/XhoI | CAGTGCAGGATATCATGGAGAGAATAAAAGAATTACG | GCGACTCGAGTCAAACTTCTGACTCAATTGTTCTCG |
| Myc_PB2$_{\Delta}$_ EcoRV/XhoI | CAGTGCAGGATATCATGGAGAGAATAAAAGAATTACG | GCGACTCGAGTTATAGTTGAACACAGGGGAATTG |

Primers used for strand-specific reverse transcription:

| Gene | Forward 5‘-3‘ | Reverse 5‘-3‘ |
|---|---|---|
| PB2$_{\Delta}$_vRNA | GGCCGTCATGGTGGCGAATGAGATGATTCCTGAAAGGAATAGG | |
| PB2$_{\Delta}$_cRNA | | GCTAGCTTCAGCTAGGCATCAGTAGAAACAAGGTCGTTTTTAAAT |
| PB2$_{\Delta}$_mRNA | | CCAGATCGTTCGAGTCGTTTTTTTTTTTTTTTTTAAATAATTCGA |

Primers used for quantitative real-time RT-PCR:

| Gene | Forward 5‘-3‘ | Reverse 5‘-3‘ |
|---|---|---|
| *human:* | | |
| GAPDH | GCAAATTCCATGGCACCGT | GCCCCACTTGATTTTGGAGG |
| IFNβ | TCTGGCACAACAGGTAGTAGGC | GAGAAGCACAACAGGAG |
| IP10 | GGAACCTCCAGTCTCAGCACCA | AGACATCTCTTCTCACCCTTC |
| IL-6 | TGAGATCTACTCGGCAAACCTAGTG | CTTCGTAGAGAACAACATAAGTCAGATACC |
| *murine:* | | |
| GAPDH | GTCCACCAGCCTGTTGCTGTAG | CCCACTCTTCCACCTTCGATG |
| IFNβ | CGCCTGGATGGTGGTC | AGTCCGCCTCTGATGCTTA |
| IRF7 | GAGACTGGCTATTGGGGGAG | GACCGAAATGCTTCCAGGG |
| Mx1 | GATCCGACTTCACTTCCAGATGG | CATCTCAGTGGTAGTCAACCC |
| OAS-1 | GTCAATGTCGTGTGTGATTTCCT | CTCCCCGTCGGTTTAACTGA |
| *canine:* | | |
| GAPDH | GCCAACATCAAATGGGGTGATGC | AGAGATGATGACCCTCTTGG |
| *influenza A virus:* | | |
| M1 | AGATGAGTCTTCTAACCGAGGTCG | TGCAAAAACATCTTCAAGTCTCTG |
| PB2 | ACCGGAGCAGAGCAGAATG | CTGTTAATGCACCTGCGTCCT |
| PB2$_{\Delta}$ | GGACCATATGGCCATAATCAAG | GAGTATTCTCATTCCTATTCCTTTCAG |
| PB2$_{\Delta}$_vRNA | GGCCGTCATGGTGGCGAAT | GTCCTTTCCAAGAACGGTAAG |
| PB2$_{\Delta}$_cRNA | GATGATTCCTGAAAGGAATAGG | GCTAGCTTCAGCTAGGCATC |
| PB2$_{\Delta}$_mRNA | GATGATTCCTGAAAGGAATAGG | CCAGATCGTTCGAGTCGT |
