# Supplementary material for: Evidence for a Novel Mechanism of Influenza Virus-Induced Type I Interferon Expression by a Defective RNA-Encoded Protein
Source: PLoS Pathog. 2015 May 29;11(5):e1004924. doi: 10.1371/journal.ppat.1004924 (PMC4449196; doi:10.1371/journal.ppat.1004924)
Supplement: S1 Fig — A549 cells were infected with 5 MOI KAN-1 and total RNA was isolated at the time points indicated. Expressional changes of the different PB2Δ RNA species were detected by qRT-PCR and are depicted as mean n-fold (±SD) of three independent experiments normalized to the respective RNA expression 2 h p.i.. (PDF) [file ppat.1004924.s005.pdf]

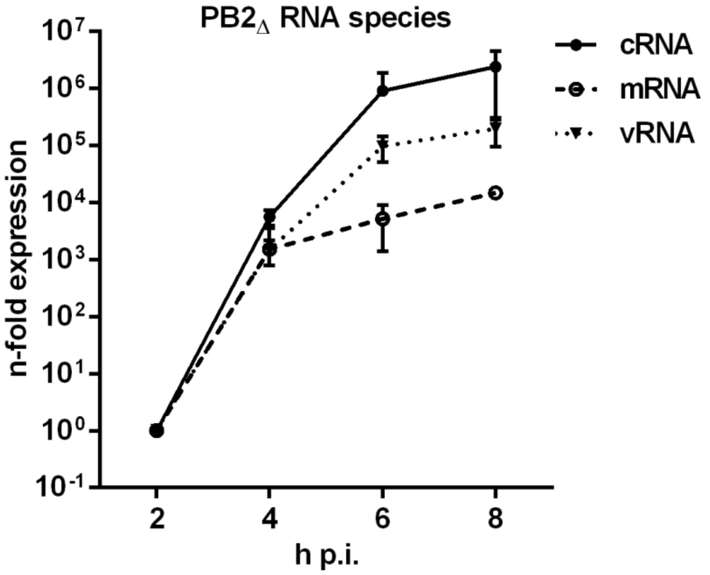

S1 Fig. Expressional changes of $PB2_{\Delta}$ RNA species. A549 cells were infected with 5 MOI KAN-1 and total RNA was isolated at the time points indicated. Expressional changes of the different $PB2_{\Delta}$ RNA species were detected by qRT-PCR and are depicted as mean *n*-fold (±SD) of three independent experiments normalized to the respective RNA expression 2 h p.i..
