# Supplementary material for: Evidence for a Novel Mechanism of Influenza Virus-Induced Type I Interferon Expression by a Defective RNA-Encoded Protein
Source: PLoS Pathog. 2015 May 29;11(5):e1004924. doi: 10.1371/journal.ppat.1004924 (PMC4449196; doi:10.1371/journal.ppat.1004924)
Supplement: S2 Fig — 100 μg of protein mixtures from KAN-1-infected A549 cells were separated on a one-dimensional gel followed by in-gel digest by trypsin. LC-MS/MS analyses were performed on an EasyLC nano-HPLC (Proxeon Biosystems) coupled to an LTQ Orbitrap Elite mass spectrometer (Thermo Scientific). The MS data were processed using default parameters of the MaxQuant software (v1.2.2.9). (PDF) [file ppat.1004924.s006.pdf]

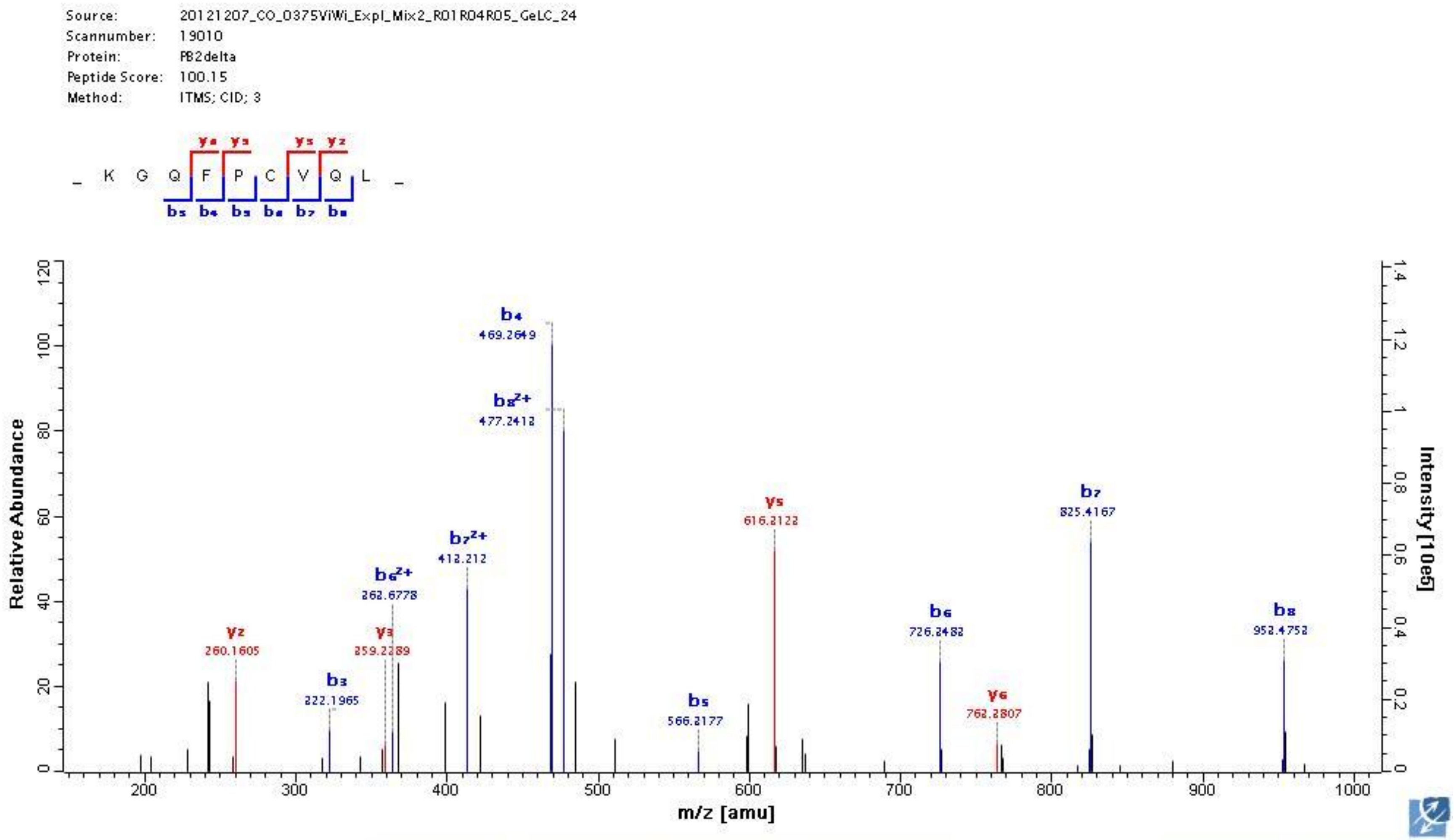


**S2 Fig. MS/MS spectrum of the tryptic C-terminal unique peptide KGQFPCVQL of the H5N1 KAN-1 virus protein PB2$_{\Delta}$.** 100 µg of protein mixtures from KAN-1-infected A549 cells were separated on a one-dimensional gel followed by in-gel digest by trypsin. LC-MS/MS analyses were performed on an EasyLC nano-HPLC (Proxeon Biosystems) coupled to an LTQ Orbitrap Elite mass spectrometer (Thermo Scientific). The MS data were processed using default parameters of the MaxQuant software (v1.2.2.9).
