# Supplementary material for: Evidence for a Novel Mechanism of Influenza Virus-Induced Type I Interferon Expression by a Defective RNA-Encoded Protein
Source: PLoS Pathog. 2015 May 29;11(5):e1004924. doi: 10.1371/journal.ppat.1004924 (PMC4449196; doi:10.1371/journal.ppat.1004924)
Supplement: S3 Fig — A549 cells were infected with 5 MOI of rKAN-1 WT or PB2Δ for 2, 4, 6 or 8 h. Expression of viral proteins PB1, PB2, NP, M1 and NS1 was analyzed by Western blot. Presence of PB2Δ was verified by using PB2-specific antibodies. ERK2 expression served as loading control. Blots are representative of three independent experiments. (PDF) [file ppat.1004924.s007.pdf]

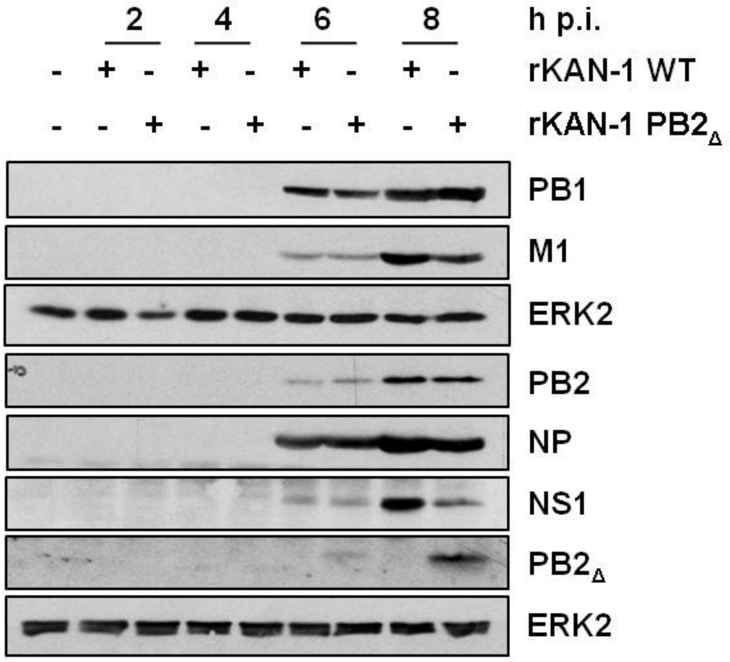

S3 Fig. Expressional changes of viral proteins in presence of PB2Δ protein. A549 cells were infected with 5 MOI of rKAN-1 WT or PB2Δ for 2, 4, 6 or 8 h. Expression of viral proteins PB1, PB2, NP, M1 and NS1 was analyzed by Western blot. Presence of PB2Δ was verified by using PB2-specific antibodies. ERK2 expression served as loading control. Blots are representative of three independent experiments.
