# Supplementary material for: Evidence for a Novel Mechanism of Influenza Virus-Induced Type I Interferon Expression by a Defective RNA-Encoded Protein
Source: PLoS Pathog. 2015 May 29;11(5):e1004924. doi: 10.1371/journal.ppat.1004924 (PMC4449196; doi:10.1371/journal.ppat.1004924)
Supplement: S4 Fig — BALB/c mice were infected with 50 pfu rKAN-1 wild type or PB2Δ-expressing virus. A) Expression changes of IFNβ mRNA in lungs were analyzed two days p.i. by qRT-PCR. N-fold expression in individual animals normalized to uninfected PBS control mice is depicted. B) Viral lung titers 2 days p.i. of individual animals are depicted. A, B) Statistical significance was analyzed by Mann-Whitney test. (PDF) [file ppat.1004924.s008.pdf]

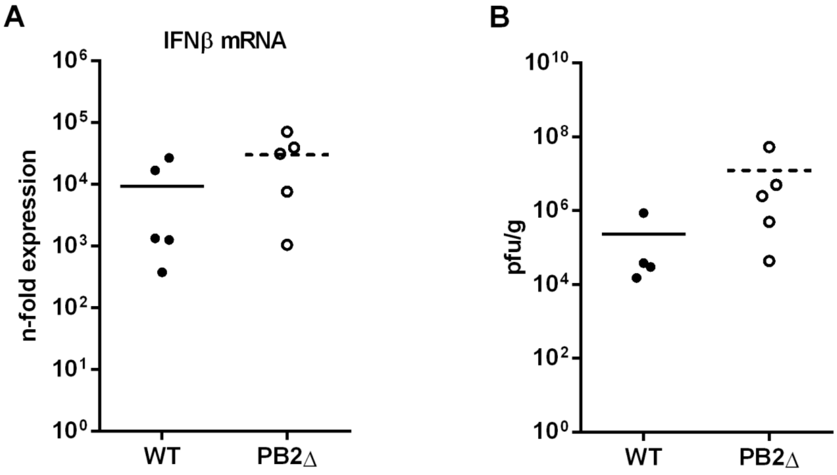

S4 Fig. Effects of PB2$_{\Delta}$ protein *in vivo*. BALB/c mice were infected with 50 pfu rKAN-1 wild type or PB2$_{\Delta}$-expressing virus. **A)** Expression changes of IFNβ mRNA in lungs were analyzed two days p.i. by qRT-PCR. N-fold expression in individual animals normalized to uninfected PBS control mice is depicted. **B)** Viral lung titers were analyzed two days p.i. by standard plaque assay and are depicted for individual animals. **A, B)** Statistical significance was analyzed by Mann-Whitney test.
